# Observations on heterodonty within the dentition of the Atlantic Sharpnose Shark, *Rhizoprionodon terraenovae* (Richardson, 1836), from the north-central Gulf of Mexico, USA, with implications on the fossil record

**DOI:** 10.7717/peerj.15142

**Published:** 2023-04-12

**Authors:** Jun A. Ebersole, Abigail T. Kelosky, Bryan L. Huerta-Beltrán, David J. Cicimurri, J. Marcus Drymon

**Affiliations:** 1 Collections Department, McWane Science Center, Birmingham, Alabama, United States; 2 School of Biological, Environmental, and Earth Sciences, The University of Southern Mississippi, Hattiesburg, Mississippi, United States; 3 Natural History Department, South Carolina State Museum, Columbia, South Carolina, United States; 4 Coastal Research and Extension Center, Mississippi State University, Biloxi, Mississippi, United States; 5 Mississippi-Alabama Sea Grant Consortium, Ocean Springs, Mississippi, United States

**Keywords:** Gynandric heterodonty, Teeth, Monognathic heterodonty, Dignathic heterodonty, Ontogenetic heterodonty, Intraspecific variation

## Abstract

The Atlantic Sharpnose Shark, *Rhizoprionodon terraenovae* (Richardson, 1836), is the most common small coastal requiem shark in the north-central Gulf of Mexico, USA. Despite this fact, little is known about the dental variation within this taxon. To help rectify this shortcoming, we examined 126 male and female *R. terraenovae* jaws sets across all maturity stages to document the various types of heterodonty occurring in the dentition of this taxon. Quantitative data gathered from a subset of our sample allowed for us to place teeth within the dentition of *R. terraenovae* into standardized upper and lower parasymphyseal/symphyseal, anterior lateral, and posterior tooth groups. As with all carcharhinid sharks, the dentition of *R. terraenovae* exhibits monognathic and dignathic heterodonty. We also observed significant ontogenetic heterodonty in the species, as the teeth and dentition progress through five generalized developmental stages as the shark matures. The ontogenetic development of serrations on the teeth appears to be closely related to documented dietary changes as the shark matures. Initial diets are comprised of high percentages of invertebrate prey like shrimp, crabs, and squid, but this transitions through ontogeny to a diet that is more reliant on fishes. We also provide the first documentation of gynandric heterodonty in mature male *R. terraenovae*, with development of these seasonal teeth likely enabling a male to grasp female sharks during copulation. Our analysis revealed a tremendous amount of variation in the dentition of *R. terraenovae*, which has direct implications on the taxonomy of fossil *Rhizoprionodon*. A comparison of the jaws in our sample to those of the extant species of *Rhizoprionodon* and the morphologically similar *Loxodon*, *Scoliodon*, and *Sphyrna* allowed us to formulate a list of generic-level characteristics to assist with the identification of isolated teeth. When applied to the fossil record, it is shown that some species previously assigned to *Rhizoprionodon* likely belong to one of the other aforementioned genera. The earliest occurrence of unequivocal *Rhizoprionodon* teeth in the fossil record are those of the Eocene †*R. ganntourensis* (Arambourg, 1952), the oldest records of which occur in early Ypresian deposits in Alabama and Mississippi, USA. The early Eocene occurrence of unequivocal fossil *Rhizoprionodon* teeth in Alabama predates the first occurrence of *Negaprion*, *Galeocerdo*, and *Carcharhinus* teeth in the state, supporting published molecular and morphological phylogenies positing a basal position for *Rhizoprionodon* within the Carcharhinidae.

## Introduction

The Atlantic Sharpnose Shark, *Rhizoprionodon terraenovae* (Richardson, 1836), is the most common small coastal requiem shark in the north-central Gulf of Mexico, USA (Drymon, Powers & Carmichael, 2012). Consequently, much is known about the distribution (Drymon et al., 2010; Bethea et al., 2014), movement (Gurshin & Szedlmayer, 2004; Carlson et al., 2008), age and growth (Carlson & Baremore, 2003), reproduction (Parsons, 1983; Hoffmayer et al., 2013), sexual segregation (Drymon et al., 2020), and population structure (Davis, Suárez-Moo & Daly-Engel, 2019) of this species. The dietary habits of Atlantic Sharpnose Sharks have been particularly well studied and demonstrate that the trophic niche of this species varies spatially (Drymon, Powers & Carmichael, 2012; Plumlee & Wells, 2016; Seubert et al., 2019) and ontogenetically (Bethea, Buckel & Carlson, 2004; Bethea et al., 2006; Harrington et al., 2016). Atlantic Sharpnose Sharks of the north-central Gulf of Mexico shift from an invertebrate-rich diet as juveniles to one consisting primarily of fish as adults, with a concurrent increase in prey size as individual body size increases.

The genus *Rhizoprionodon* is relatively diverse, with seven recognized extant species that includes *R. acutus* (Rüppell, 1837), *R. lalandii* (Valenciennes in Müller & Henle, 1839), *R. longurio* (Jordan & Gilbert, 1882), *R. oligolinx* Springer, 1964, *R. porosus* (Poey, 1861), *R. taylori* (Ogilby, 1915), and *R. terraenovae*. Although *Rhizoprionodon* is nearly circumglobally distributed, only *R. porosus* and *R. terraenovae* have been reported in the Gulf of Mexico (Davis, Suárez-Moo & Daly-Engel, 2019). With respect to the fossil record of the genus, Cappetta (2012) recognized three species of fossil *Rhizoprionodon*, including the lower-to-middle Eocene †*R. ganntourensis* (Arambourg, 1952), late Eocene †*R. bisulcatus* Li, 1997, and Oligocene to Pliocene †*R. ficheuri* (Joleaud, 1912). However, it is possible that one or more fossil *Scoliodon* or *Sphyrna* species may also belong to this genus (or vice versa) (Cappetta, 2012). Fossil *Rhizoprionodon* species are known exclusively by their teeth, and the Paleogene representatives are herein considered to be ancestral to the extant taxa. Of the fossil species, only †*R. ganntourensis* has been previously reported from the Gulf Coastal Plain of the USA (Ebersole, Cicimurri & Stringer, 2019).

Despite a relatively comprehensive understanding of the ecology and diet of extant *Rhizoprionodon* species, few detailed studies have been conducted that describe the tooth morphology and development of heterodonty within the dentitions of those species. Both Springer (1964) and Compagno (1984, 1988) noted the presence or absence of serrations on the teeth of certain species (alluding to ontogenetic heterodonty in some taxa), and they mentioned the development of gynandric heterodonty (dental sexual dimorphism) in the various species. Springer (1964) went so far as to propose two *Rhizoprionodon* subgenera, *R*. (*Protozygaena*) and *R*. (*Rhizoprionodon*), based in part on the presence or absence, respectively, of gynandric heterodonty within the various taxa. Based on this characteristic, Springer (1964) placed two species that exhibited gynandric heterodonty, *R. lalandii*, and *R. oligolinx*, in *R*. (*Protozygaena*), and *R. acutus*, *R. longurio*, *R. porosus*, and *R. terraenovae* were placed in *R*. (*Rhizoprionodon*) because gynandric heterodonty was thought to be absent. Springer (1964) and Compagno (1984) lacked the specimens necessary to evaluate the development of gynandric heterodonty in *R. taylori*, but the former included this taxon within his *R*. (*Protozygaena*) subgenus based on other criteria. Unfortunately, neither Springer (1964) nor Compagno (1984, 1988) provided detailed tooth descriptions for any *Rhizoprionodon* species. However, Springer (1964) included line drawings of dentitions of most of the extant taxa, Compagno (1984) illustrated representative upper and lower teeth of the various species, and Compagno (1988) figured the upper and lower dentition of *R. longurio*. Garry (2003, 2004) utilized geometric morphometrics to quantify shape differences between five extant *Rhizoprionodon* species, representatives of the closely related genera *Loxodon*, *Eusphyra*, and *Sphyrna*, and fossil *Rhizoprionodon* teeth. He concluded that although geometric morphometrics could be used to differentiate between the teeth of the genera he included in his analyses, the various extant and fossil *Rhizoprionodon* species could not be sufficiently differentiated with this technique. Nevertheless, Garry’s (2003, 2004) shape analysis showed quantifiable differences between the teeth in the Meckel’s cartilage and palatoquadrate of extant *Rhizoprionodon* jaws, as well as differences between some of the tooth files within the jaws (indications of both monognathic and dignathic heterodonty within the genus). Schwartz & Hurst (1996) calculated tooth surface areas for newborn and mature male and female specimens of *R. terraenovae* and concluded that the upper teeth had a greater surface area than those in the lower files (an indication of dignathic heterodonty in the taxon).

The purpose of this study is to describe the dentition and document the various types of heterodonty in *Rhizoprionodon terraenovae* specimens from the north-central Gulf of Mexico, USA. As has been done with several other taxa (*i.e*., Powter, Gladstone & Platell, 2010; Cullen & Marshall, 2019; Türtscher et al., 2022), we also discuss the various types of dental variation observed with respect to gynandric heterodonty, ontogeny, dietary shifts, and life history of the species. Our study has relevance to the fossil record because a thorough understanding of heterodonty within extant shark taxa is necessary to accurately interpret an assortment of isolated teeth recovered from any particular geologic unit. For instance, does an assortment of five isolated teeth represent five biological species (intraspecific variation) or heterodonty (intraspecific variation) within a single biological species? This is important when one interprets the paleobiodiversity, paleoecology, and paleobiogeographic distributions of extinct taxa. Specific to *Rhizoprionodon*, we review the valid extinct species and provide insights into the origin, evolutionary history, and paleobiodiversity of the genus. Additionally, we discuss the morphological features that can be used to distinguish isolated fossil teeth of *Rhizoprionodon* from those of morphologically similar *Loxodon*, *Scoliodon*, and Sphyrnidae, and we utilize these criteria to report new fossil *Rhizoprionodon* material from the northern Gulf Coastal Plain of the USA.

The larger intent of this study is to fill a need for both chondrichthyan neontologists and paleontologists alike, as detailed studies on morphological tooth variation are lacking for most chondrichthyan taxa (Guinot et al., 2018; Mollen, 2019). Gaining a broader understanding of the various forms of heterodonty within the dentitions of extant taxa like *R. terraenovae* allows paleontologists to identify isolated teeth more precisely in the fossil record. Thus, studies such as this one (as well as those on other taxa) provide a means for paleontologists to fill in gaps in the fossil record and help them to better interpret the evolutionary history and past diversity and distribution of these taxa. This, in turn, provides neontologists with more accurate node age estimates to calibrate molecular clocks while also shedding light onto chondrichthyan responses to past environmental, climatic, and anthropogenetic events. This latter point helps to increase our understanding of similar trends observed amongst extant chondrichthyan populations, ultimately providing critical insights necessary for the successful management and conservation of extant populations (Guinot et al., 2018; Paillard, Shimada & Pimiento, 2020).

## Materials and Methods

### Sample collection, extraction, cleaning, and data repositories

**Figure 1:**
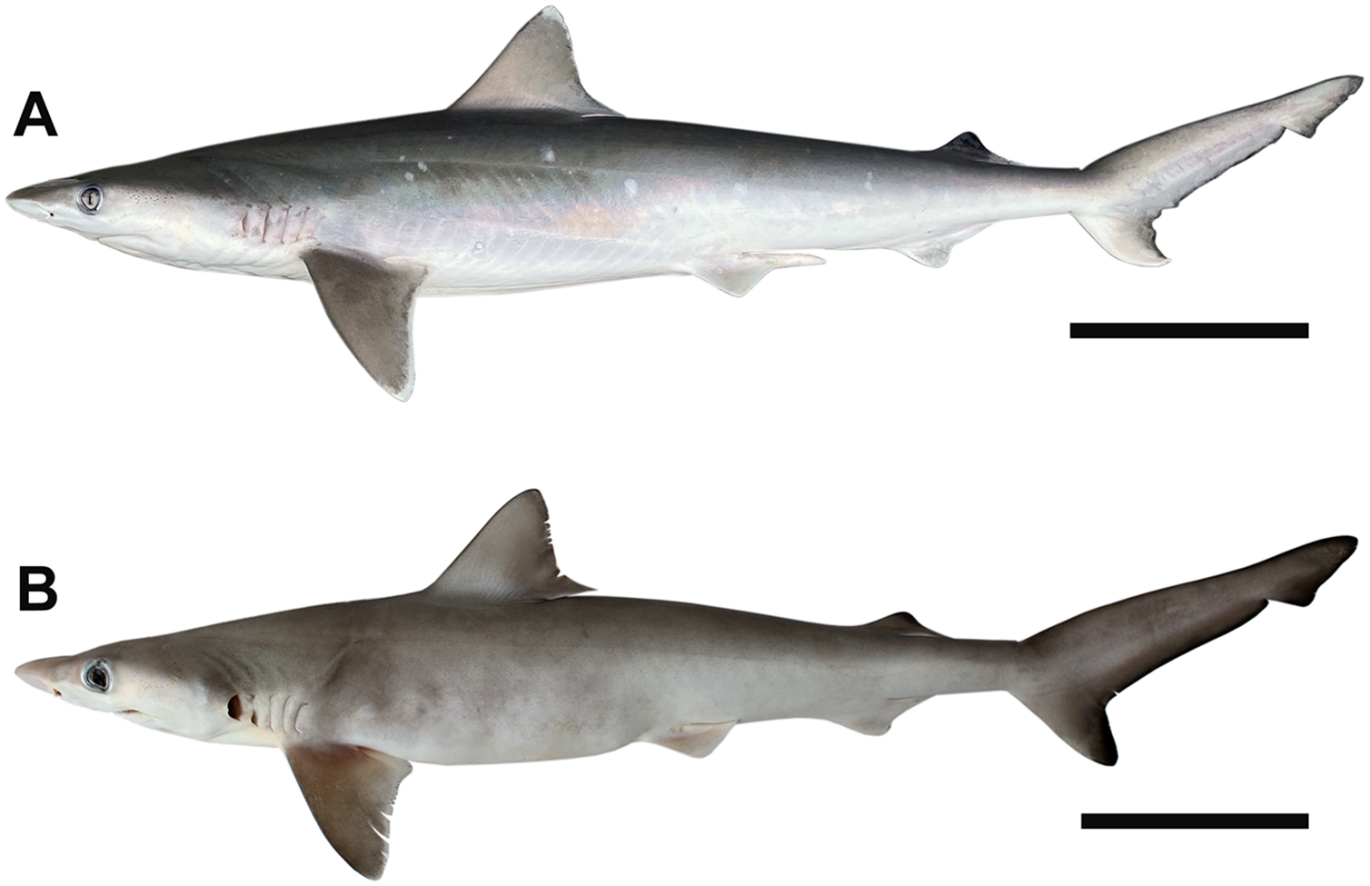
Photographs of *Rhizoprionodon terraenovae* specimens from the northern Gulf of Mexico, USA. (A) Male, STL 820 mm, scale bar = 150 mm. (B) Female *in utero* pup, STL = 180 mm, scale bar = 30 mm. Photographs by Bryan L. Huerta-Beltrán.

We examined a total of 126 *Rhizoprionodon terraenovae* jaw sets, including both males and females, across various size and maturity classes, including pups, neonates, immature, transitional, and mature individuals (see Appendix 1.1). All specimens were captured in the northern Gulf of Mexico off the coast of Alabama between 2018 and 2022 as part of an ongoing shark population monitoring program conducted by the Mississippi State University Coastal Research and Extension Center in Biloxi, MS, USA. For complete methodological sampling details, see Drymon et al. (2020), but in short, Atlantic Sharpnose Sharks were caught on commercial-style bottom longline gear (~2 km in length), set with 100 gangions. Each gangion consisted of a longline swivel and a 15/0 circle hook baited with Atlantic Mackerel (*Scomber scombrus* Linnaeus, 1758). Bottom longline sets were soaked for 1 h; once retrieved, sharks were removed from the mainline, unhooked, and identified to species following Springer (1964) and Castro (2010) (Fig. 1). Notably, the Caribbean Sharpnose Shark (*R. porosus*) can only be differentiated from the Atlantic Sharpnose Shark based on precaudal vertebral counts (Springer, 1964) and molecular diagnostic techniques (Mendonça et al., 2011). However, all individuals sampled for this project were assumed to be Atlantic Sharpnose Sharks because their catch location was far removed from the known reported range of *R. porosus* (Davis, Suárez-Moo & Daly-Engel, 2019; Carlson et al., 2021). For each individual, length (*i.e*., precaudal (PCL), fork (FL), and stretched total (STL) in mm), weight (in kg), sex, and maturity stage (when possible) were recorded. Maturity in males was based on the extent of calcification of the myxopterygia following Clark & von Schmidt (1965). Males were considered mature if the myxopterygia were (1) fully calcified, (2) able to rotate 180° anteriorly, and (3) possessed a flared rhipidion. Males that met none of the aforementioned criteria were defined as juvenile, whereas individuals that met some (but not all) of the criteria were considered in transition between juvenile and adult. Females were classified as mature based on the presence of pups *in utero*.

Given logistical challenges associated with sampling during the winter months, most of the sampling effort for this monitoring program took place in meteorological spring, summer, and fall (*i.e*., March–November). Therefore, no Atlantic Sharpnose Shark jaws were collected during December, January, or February. All individuals were collected under NOAA Fisheries HMS permits issued to one of the authors (JMD). Specimens were humanely euthanized through emersion in an ice bath in accordance with Mississippi State University’s Institutional Animal Care and Use Committee (IACUC) protocol 17-620.

Jaws were manually extracted with dissection tools by the current authors, members of the Mississippi State University Marine Fisheries Ecology Program, or by undergraduate students enrolled in the Shark and Ray Biology summer field course at the Dauphin Island Sea Lab, AL, USA. Extracted jaws were individually bagged and labeled with a unique Fish ID (when available), STL, sex, and maturity stage. Whenever pups were present, they were removed from the pregnant female, and all members of the same litter were individually labeled and bagged together with the adult’s jaws. All specimens were frozen and transported to McWane Science Center (MSC) in Birmingham, AL, USA for cleaning and analysis. The jaws were cleaned by three of the authors (ATK, BLH, JAE) using dissection tools. Pups were individually sexed and measured (*i.e*., STL) in the laboratory before their jaws were extracted. All flesh and connective tissue were removed from both labial and lingual sides of the jaws to expose all tooth files and rows. The jaws were then soaked in a 10% hydrogen peroxide solution, serving as a final cleaning and bleaching agent. Afterward, all jaws were posed in an open position so that all teeth could be easily observed, then dried in a fume hood. Each specimen was assigned a unique MSC catalog number that was recorded in a Microsoft Excel 365 spreadsheet along with all other relevant specimen information (see below). Pup jaws were assigned the same MSC number as those of the adult female shark from which they were removed but were given itemized sub-numbers (for example, MSC 44471.2). This numbering convention was utilized to retain the association of all pups in a litter with their adult female parent. All jaws are permanently accessioned into the scientific collections at MSC, and complete field data for each specimen that was assigned a Fish ID is archived at the Mississippi State University Coastal Research and Extension Center in Biloxi.

### Analyses, tooth extraction, and figures

Both quantitative and qualitative analyses were performed in this study. The first quantitative analysis included the calculation of dental formulae for each jaw to evaluate whether the number of functional teeth within the dentitions of *R. terraenovae* is variable. Dental formulae were calculated by counting the number of functional teeth on the left and right sides of the symphysis on both the Meckel’s cartilage and palatoquadrate. For each jaw, tooth files were counted starting at the symphysis and moving sequentially towards the commissure on both the right and left sides of the jaw, and each tooth file present was marked on the datasheet. For example, the dental formula for the palatoquadrate of specimen MSC 43586 was calculated to be 13-1-12, with a symphyseal file (middle number) separating the 13 files in the left jaw from the 12 files in the right jaw. This same convention was applied to the Meckel’s cartilage, but with the middle numeral representing the number of parasymphyseal teeth in the functional row (*i.e*., those occurring to the left and right of the symphysis, but not within the left or right dental hollows). Dental formulae were not calculated for the four smallest pup specimens in our sample (MSC 42685.2, MSC 42685.3, MSC 42685.4, MSC 42685.6) because the individual teeth, tooth rows, and tooth files were still developing in these jaw sets. These small jaws were analyzed separately because they provided us with unique insights regarding the ontogenetic development of *R. terraenovae* teeth and dentitions.

A second quantitative analysis involved taking a series of measurements of teeth from the functional rows of numerous jaw sets to test whether the teeth could be quantitatively placed within specific tooth groups. To conduct this latter analysis, all teeth were removed from one side of Meckel’s cartilage and palatoquadrate of 20 select specimens in our sample. To account for morphological differences resulting from gynandric heterodonty and/or ontogeny, tooth measurements from 10 female and 10 male jaws (*n* = 20) with nearly identical STLs were taken across various size classes. These ten size classes included female and male pairs with STLs of 600 mm (MSC 42649 and MSC 42662), 610 mm (MSC 42645 and MSC 42663), 615 mm (MSC 43583 and MSC 44474), 635 mm (MSC 43585 and MSC 42657), 675 mm (MSC 42638 and MSC 42651), 895 mm (MSC 42685.1 and MSC 42656), 935/940 mm (MSC 44482.1 and MSC 44454), 980 mm (MSC 44481.1 and MSC 44480), 1,000/1,001 mm (MSC 42670 and MSC 42671), and 1,030/1,033 mm (MSC 42676 and MSC 44456).

All teeth from either the left or right functional rows of both the Meckel’s cartilage and palatoquadrate were extracted by soaking the jaws in warm water to the point where all the individual teeth could be easily removed with forceps and a scalpel. Soaking times varied depending on the jaw size, but generally averaged between 30 to 60 min. Individual teeth were removed one at a time, beginning at the symphysis, then sequentially along the functional tooth row towards the commissure. The extracted teeth were placed individually into gelatin capsules, labeled by jaw and specific tooth file, and stored with the jaw from which they were extracted. Due to the small size of the teeth, all were examined under an AmScope FMA050 microscope and measured digitally in Toupview v64 software. Although the Toupview software measures in pixels (px), pixel measurements could be converted to millimeters (mm) by photographing specimens on top of a mm plastic scale bar that was overlain with a horizontal 1,000 px digital scale bar. The focal length of the microscope was then adjusted so the edges of the 1,000 px digital scale bar aligned precisely with 5.0 mm on the plastic scale bar (therefore 1,000 px = 5 mm). Once aligned, any digital px measurements could be converted to mm by using the following formula: px measurement ÷ 2 ÷ 100. Millimeter conversions were rounded to the nearest hundredth (with 0.005 rounded up) and all converted measurements were recorded in a Microsoft Excel 365 spreadsheet.

**Figure 2:**
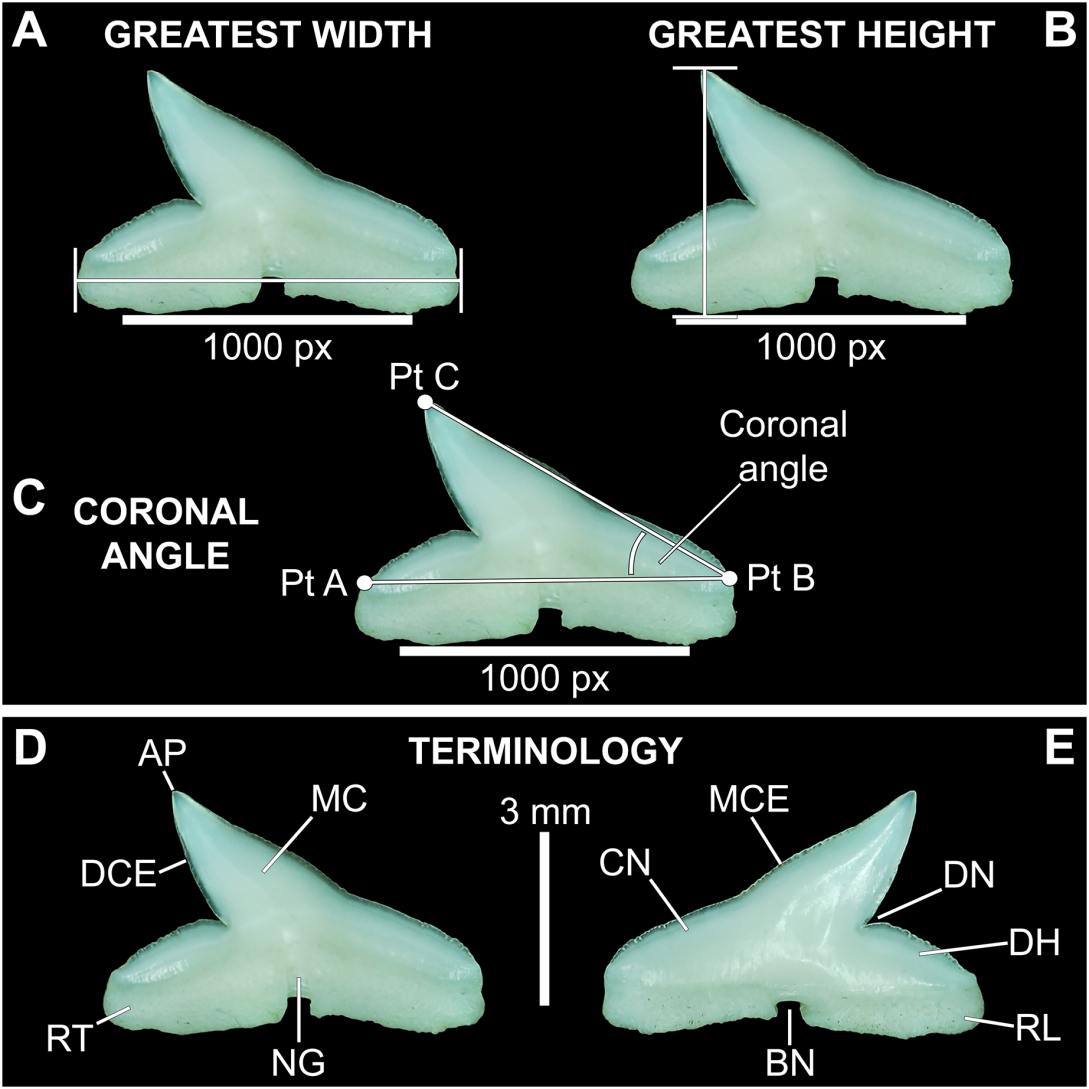
Standard tooth measurements and morphological terms. (A–E) MSC 44461.1, lower left lateral tooth, mature female, STL 991 mm. (A–D) Lingual view; (E) labial view. Abbreviations: AP, apex; BN, basal notch; DCE, distal cutting edge; DH, distal heel; CN, crown; DN, distal notch; MC, main cusp; MCE, mesial cutting edge; NG, nutritive groove; RL, root lobe; RT, root. Scale bar for A–C = 1,000 px/5 mm; D–E = 3 mm.

Three digital measurements were taken for each extracted tooth (*n* = 466), including the greatest mesiodistal width (Fig. 2A), greatest apicobasal height (Fig. 2B), and coronal angle (Fig. 2C), and height/width (H/W) ratios were calculated for each tooth by dividing the height value by the width value. Before digital measurements were taken, isolated teeth were first aligned in a standard position under the microscope. To do so, teeth were placed on their labial face (*i.e*., lingual side facing up) and rotated so the basal-most edges of the mesial and distal root lobes rested precisely upon the upper margin of a horizontal 1,000 px digital scale bar (Figs. 2A–2C). Once the teeth were aligned, the greatest mesiodistal width measurements (Fig. 2A) were taken using the Toupview “horizontal measuring tool.” This tool utilizes a horizontal line to measure the distance between two points along a horizontal axis (rather than an angular line between two points), and in this case, it was used to measure the distance between the mesial-most and distal-most points of the tooth. The greatest apicobasal height (Fig. 2B) was measured in a similar fashion by utilizing the Toupview “vertical measuring tool,” which was used to measure the distance between the apical-most and basal-most points of the tooth. The coronal angle (Fig. 2C) was measured using the Toupview “three point angle tool,” which measures the angle between three marked points. On each tooth, a point (Pt. A) was placed along the distal edge of the crown base, precisely where the crown base intersects the root. The second point (Pt. B) was placed at the corresponding crown base/root intersection on the mesial edge, and the third point (Pt. C) was placed at the apex of the main cusp. On teeth where the crown apex was blunt, Pt. C was placed in the middle of the rounded apex. In this study, the coronal angle represents the angle between the lines connecting Pts. A and B and Pts. B and C (Fig. 2C).

All digital measurements and angles were tabulated in Microsoft Excel 365 and organized according to upper or lower jaw (*i.e*., palatoquadrate or Meckel’s cartilage), ascending order of STL, and descending order of tooth file. The data was also separated by sex. The mean and standard error were calculated for all measurements and angles across the various tooth positions, and the full suite of measurements and angles taken for each tooth is provided in Appendices 1.10–1.18. Data marked in Appendices 1.10–1.18 as “NA” refers to missing data resulting from teeth that were too small to be removed from the jaw (which was the case with several of the posterior-most teeth on some of the smaller jaws), teeth from which a complete set of measurements could not be taken (due to damage), or in some instances the main cusp was not developed on the posterior-most teeth so the coronal angle could not be measured.

Our study also included two qualitative analyses. The first involved a detailed examination of the labial aspect of each *in situ* tooth within the functional rows of the Meckel’s cartilage and palatoquadrate of 122 jaw sets in our sample (the four smallest jaws were excluded from this analysis because their dentitions were not fully developed). In this study, the functional rows are defined as the labial-most rows in the jaw that were actively used for feeding. Within the functional rows of *R. terraenovae* jaws, all tooth apices are erect and angled toward the mouth opening, whereas the apices of the teeth in the replacement rows are downturned and face away from the mouth opening.

Morphological data was captured from three regions of the tooth crown for each functional tooth, including the distal heel and the mesial and distal cutting edges of the main cusp (Figs. 2D, 2E). However, because the tooth rows in the right and left halves of the Meckel’s cartilage and palatoquadrate developed synchronously (*i.e*., the morphological changes observed on the right side were essentially the same as those on the left side), detailed morphological data from only one side of the jaw was recorded (*i.e*., the side with the most complete functional row in both the upper and lower jaws). On the individual teeth, particular attention was paid to the shape of the cutting edge on the distal heel, which was recorded as rounded, triangular, and/or bifurcated (see Results). A rounded distal heel is herein defined as being roughly evenly convex, whereas a triangular heel has straight mesial and distal cutting edges and a pointed apex. A bifurcated distal heel has one or more notable crenulations on the cutting edge, with the crenulations being larger, more irregular, and deeper than what we consider as serrations (see below). See the Results section for figured examples of rounded, triangular, and bifurcated distal heels.

The nature of the mesial and distal cutting edges of the main cusp and on the distal heel was also evaluated and classified as smooth, irregular, or serrated. A smooth cutting edge, as defined by us, is one that lacks any signs of irregularity, including crenulations or serrations. Our concept of a serrated cutting edge is one where all or a portion of the cutting edge is subdivided by two or more fully developed saw-like denticles/serrae. To be considered fully developed, individual serrae have well-defined and convex mesial and distal edges, with each of the serrae being separated from each other by a distinct gap. In contrast, cutting edges identified as irregular exhibit a morphology intermediate between smooth and serrated. This is generally expressed as a crenulated (weakly scalloped) edge and/or incompletely developed serrae, meaning either or both the mesial and distal edges of the serrae were not well delineated from the remainder of the cutting edge. Although some serrated cutting edges also exhibited characteristics of irregular edges, the edge was classified as being serrated if two or more completely developed serrae were present. It is also important to note that serrations and crenulations are often not developed along the entire length of a cutting edge, and they may be located apically, medially, or basally on the edge. Examples of what we define as smooth, irregular, and serrated cutting edges are illustrated in the Results section. Finally, it should also be noted that the tooth root morphology was excluded from our observational analyses because the teeth were observed *in situ* within the jaws, and much of each tooth’s root was obscured due to the imbricated nature of the tooth files (see Results) and/or by the presence of connective gum tissue. Although this particular analysis was largely limited to observations based on the labial face of the tooth crowns, we provide detailed morphological descriptions and figures of the root and lingual face of teeth from across the tooth rows of both the Meckel’s cartilage and palatoquadrate. These descriptions were based on teeth (*n* = 466) that were removed from select jaw sets as part of the aforementioned quantitative analyses, and the described root characteristics can be viewed in the illustrated representative female and male dentitions.

The jaw and tooth data from this qualitative analysis were captured in standardized data sheets created in Microsoft Excel 365. Each data sheet (*n* = 126) included all the morphological information recorded for a single jaw, including the dental formula and descriptive data like MSC number, Fish ID number, sex, maturity stage (if applicable), and date the specimen was caught. All data sheets were digitized into Microsoft Excel 365 so that the data could be sorted by sex and ascending order of STL. Once sorted, specific characteristics (for example, bifurcated distal heels) were color coded. Because it was observed that the morphological changes occurring on the individual teeth did not happen uniformly across the functional tooth row, nor between individuals or sexes, organizing the data in this fashion allowed us to visually present generalized morphological trends for each sex through ontogeny. This method of data analysis also enabled us to observe specific morphological events in the development of the teeth, like the acquisition of serrations on the teeth within a specific size class of each sex, and ultimately allowed us to formulate generalized ontogenetic/developmental stages for *R. terraenovae* jaws/teeth. The entirety of our collected descriptive and morphological data is presented in Appendices 1.19–1.34.

To quantify our morphological observations, specific tooth characteristics for each jaw (triangular distal heel, irregular mesial edge, *etc*.) were tabulated in Microsoft Excel 365 and sorted by sex, ascending order of STL, and by tooth position beginning at the symphysis and moving sequentially towards the commissure. Our tabulated data for each morphological characteristic was then plotted in Microsoft Excel 365 with a polynomial trend. This analysis allowed us to elucidate ontogenetic and sexual dimorphic trends within our dataset. Our polynomial trend plots are presented herein, and the entirety of our raw data is available in Appendices 1.19–1.34.

A second qualitative analysis was performed to test whether sexual dental dimorphism was present within *R. terraenovae*. To do so, and to account for morphological differences resulting from ontogeny, a total of 10 female and 10 male jaws (*n* = 20) with nearly identical STLs were compared across various size classes (see specimen list above). For each size class, the functional rows on both right and left sides of the Meckel’s cartilage and palatoquadrate were compared side-by-side under magnification. The functional tooth rows on each pair of jaws were evaluated for morphological differences across several criteria, including degree of upturn of the apex, mesiodistal width and height of the main cusp, convexity of the labial crown face, and morphology of the distal heel.

The teeth are described herein according to numerical position within the dentitions (see Table 1). Symphyseal and parasymphyseal teeth (*i.e*., those that occur on or just adjacent to the symphysis, respectively) were not assigned a position number. They were instead designated with an “Sy” or a “Pa.” The remaining teeth (*i.e*., those located within the right or left dental hollow of the Meckel’s cartilage or palatoquadrate) were assigned sequential numerical positions beginning with the first, anterior-most tooth, and ending with the posterior-most tooth in the row (*i.e*., the tooth located closest to the commissure).

**Table 1:** Mean values and tooth groups for female and male Rhizoprionodon terraenovae teeth through ontogeny on the palatoquadrate and Meckel’s cartilage.

Palatoquadrate																	
	Mean width				Mean height				Mean H/W ratio				Mean coronal angle				
Position	Female	N#	Male	N#	Female	N#	Male	N#	Female	N#	Male	N#	Female	N#	Male	N#	Group
Sy	2.96 (0.18)	9	2.89 (0.21)	10	2.76 (0.18)	8	2.54 (0.15)	9	0.92 (0.04)	9	0.91 (0.05)	9	49.63 (2.07)	8	48.77 (3.12)	10	Symphyseal
1	3.47 (0.26)	10	3.49 (0.22)	10	3.12 (0.29)	10	3.12 (0.22)	10	0.87 (0.04)	10	0.91 (0.02)	10	43.59 (1.96)	10	43.00 (1.20)	10	Anterior
2	4.05 (0.29)	10	3.91 (0.28)	10	3.31 (0.25)	10	3.27 (0.24)	10	0.83 (0.03)	10	0.84 (0.02)	10	37.34 (0.91)	10	36.62 (1.08)	10	Anterior
3	4.55 (0.31)	10	4.51 (0.31)	10	3.59 (0.25)	10	3.46 (0.24)	10	0.79 (0.03)	10	0.78 (0.02)	10	36.95 (1.26)	10	36.62 (0.54)	10	Anterior
4	5.28 (0.44)	10	5.36 (0.41)	10	3.60 (0.30)	10	3.60 (0.26)	10	0.68 (0.02)	10	0.68 (0.02)	10	33.15 (1.28)	10	31.78 (0.63)	10	Lateral
5	5.64 (0.43)	10	5.67 (0.43)	10	3.38 (0.29)	10	3.41 (0.27)	10	0.60 (0.02)	10	0.59 (0.02)	10	29.28 (0.67)	10	28.85 (0.69)	10	Lateral
6	5.59 (0.44)	10	5.64 (0.39)	10	3.26 (0.29)	10	3.35 (0.24)	9	0.58 (0.01)	9	0.58 (0.02)	9	28.25 (0.79)	10	28.50 (0.83)	9	Lateral
7	5.48 (0.38)	10	5.29 (0.38)	9	3.05 (0.30)	10	3.07 (0.26)	10	0.54 (0.02)	10	0.59 (0.02)	9	27.10 (0.87)	10	27.85 (0.97)	9	Lateral
8	5.42 (0.43)	10	5.31 (0.37)	10	2.71 (0.30)	10	2.81 (0.19)	10	0.49 (0.02)	10	0.53 (0.02)	10	24.14 (0.99)	10	24.86 (0.90)	10	Lateral
9	4.87 (0.49)	10	5.23 (0.38)	9	2.30 (0.30)	10	2.49 (0.19)	9	0.46 (0.02)	10	0.47 (0.02)	9	21.53 (0.96)	9	22.42 (0.71)	9	Lateral
10	4.55 (0.50)	10	4.75 (0.35)	10	1.91 (0.25)	9	2.04 (0.17)	10	0.41 (0.01)	9	0.42 (0.01)	10	18.22 (0.81)	9	19.54 (0.95)	10	Posterior
11	4.58 (0.41)	10	4.48 (0.32)	10	1.68 (0.19)	8	1.77 (0.11)	10	0.37 (0.01)	8	0.38 (0.01)	10	16.49 (0.70)	9	17.23 (0.54)	10	Posterior
12	5.39 (0.13)	2	3.89 (0.23)	6	1.81 (0.00)	2	1.39 (0.08)	7	0.33 (0.01)	2	0.39 (0.02)	6	14.40 (1.31)	2	17.15 (1.40)	7	Posterior

Meckel's Cartilage																	
	Mean width				Mean height				Mean H/W ratio				Mean coronal angle				
Position	Female	N#	Male	N#	Female	N#	Male	N#	Female	N#	Male	N#	Female	N#	Male	N#	Group
Pa	3.05 (0.23)	10	2.97 (0.18)	10	2.39 (0.19)	10	2.44 (0.15)	10	0.80 (0.01)	10	0.81 (0.04)	10	44.34 (1.28)	8	47.65 (1.88)	10	Parasymphyseal
1	4.24 (0.32)	10	4.40 (0.26)	10	3.00 (0.23)	10	2.91 (0.18)	10	0.68 (0.01)	10	0.66 (0.03)	10	37.07 (1.00)	10	34.34 (0.78)	10	Anterior
2	4.80 (0.33)	10	4.98 (0.30)	10	3.15 (0.25)	10	3.06 (0.21)	10	0.63 (0.01)	10	0.61 (0.02)	10	31.18 (1.14)	10	31.17 (1.06)	10	Anterior
3	5.08 (0.39)	10	5.26 (0.40)	10	2.98 (0.25)	9	3.04 (0.23)	10	0.59 (0.01)	9	0.57 (0.02)	10	28.17 (0.86)	10	28.90 (0.81)	10	Lateral
4	5.37 (0.40)	10	5.31 (0.37)	10	2.96 (0.25)	10	2.82 (0.21)	10	0.54 (0.01)	10	0.53 (0.01)	10	26.67 (0.82)	10	25.49 (0.80)	10	Lateral
5	5.41 (0.43)	10	5.38 (0.40)	10	2.78 (0.26)	10	2.60 (0.18)	9	0.52 (0.01)	9	0.48 (0.02)	9	24.96 (1.07)	10	23.70 (0.71)	9	Lateral
6	5.51 (0.41)	10	5.28 (0.38)	10	2.75 (0.25)	9	2.49 (0.18)	10	0.50 (0.01)	10	0.47 (0.02)	10	24.00 (0.80)	10	22.05 (0.83)	10	Lateral
7	5.25 (0.38)	9	4.99 (0.34)	10	2.33 (0.24)	10	2.39 (0.18)	10	0.48 (0.01)	9	0.46 (0.02)	10	21.33 (0.79)	10	21.75 (0.83)	10	Lateral
8	5.01 (0.41)	10	4.83 (0.35)	10	2.20 (0.21)	10	2.08 (0.19)	10	0.45 (0.01)	10	0.43 (0.02)	10	20.05 (0.71)	10	18.77 (1.14)	10	Lateral
9	4.45 (0.42)	10	4.47 (0.39)	10	1.79 (0.22)	9	1.84 (0.20)	10	0.43 (0.02)	9	0.39 (0.02)	10	16.82 (0.90)	9	16.68 (1.22)	10	Lateral
10	4.45 (0.33)	8	3.92 (0.36)	9	1.44 (0.17)	8	1.47 (0.15)	9	0.35 (0.02)	8	0.36 (0.01)	9	14.28 (1.06)	9	14.39 (0.84)	9	Posterior
11	4.36 (0.31)	5	3.81 (0.27)	5	1.30 (0.09)	5	1.31 (0.09)	5	0.34 (0.01)	5	0.33 (0.02)	5	13.40 (0.38)	8	15.09 (1.22)	5	Posterior

**Note:**

Numbers refer to specific tooth files that are numbered consecutively from the symphysis to the commissure. Abbreviations: Pa = parasymphyseal tooth. Sy = symphyseal tooth. H/W Ratio = height/width ratio. N# = Number of specimens measured. Numerals in parentheses represent standard errors values.

In addition to documenting the various morphological trends we observed within *R. terraenovae* dentitions, we compared our findings to data from local ecological studies, which ultimately allowed us to make inferences on the driving forces behind these various morphological changes. Our data was compared to those presented in recently published studies of *R. terraenovae* in the northern Gulf of Mexico, including those documenting growth curves (*e.g*., Carlson & Baremore, 2003), ontogenetic dietary shifts (*e.g*., Drymon, Powers & Carmichael, 2012; Hoffmayer et al., 2013; Appendices 1.35–1.36), and reproductive cycles (*e.g*., Hoffmayer et al., 2013). Data across these various studies were compared using stretched total length (STL). For studies that utilized lengths other than STL (*i.e*., precaudal length or fork length), those lengths were converted to STL using length-length regressions from Hoffmayer et al. (2013). As prior studies have shown that the maturity rates and diet of *R. terraenovae* varies by region (*e.g*., Plumlee & Wells, 2016; Seubert et al., 2019), our data was only compared to studies that utilized specimens captured from the northern Gulf of Mexico.

Finally, to help define both interspecific and intergeneric characteristics that can be used to better identify both *Rhizoprionodon* and similar teeth in the fossil record, our sample of *R. terraenovae* jaws were visually compared to the dentitions of extant species of *Eusphyra*, *Loxodon*, *Rhizoprionodon*, *Scoliodon*, and *Sphyrna*. Dentitions of these taxa were derived from three sources, including extant jaw sets housed in the collections at McWane Science Center in Birmingham, USA (MSC) and the South Carolina State Museum in Columbia, USA (SC), and dentitions published in the literature (*i.e*., Springer, 1964; Gilbert, 1967; Compagno, 1984, 1988; Ebert & Dando, 2021). Catalog numbers for comparative jaw sets are cited throughout the text, and a complete list of comparative specimens we examined is listed in Appendix 1.2. The graphs presented herein were generated in Microsoft Excel 365 and imported into Adobe Photoshop 2022 so they could be reformatted/recreated for publication. Figured specimens were photographed with a Nikon D80 camera with Tamron macro lens. Specimens smaller than 2 mm in greatest dimension were photographed with an AmScope FMA050 digital microscope and Toupview v64 software. Specimen photographs were rendered in Adobe Photoshop 2022 as part of the production of the presented figures.

## Results

### The dentition of *Rhizoprionodon terraenovae*

**Figure 3:**
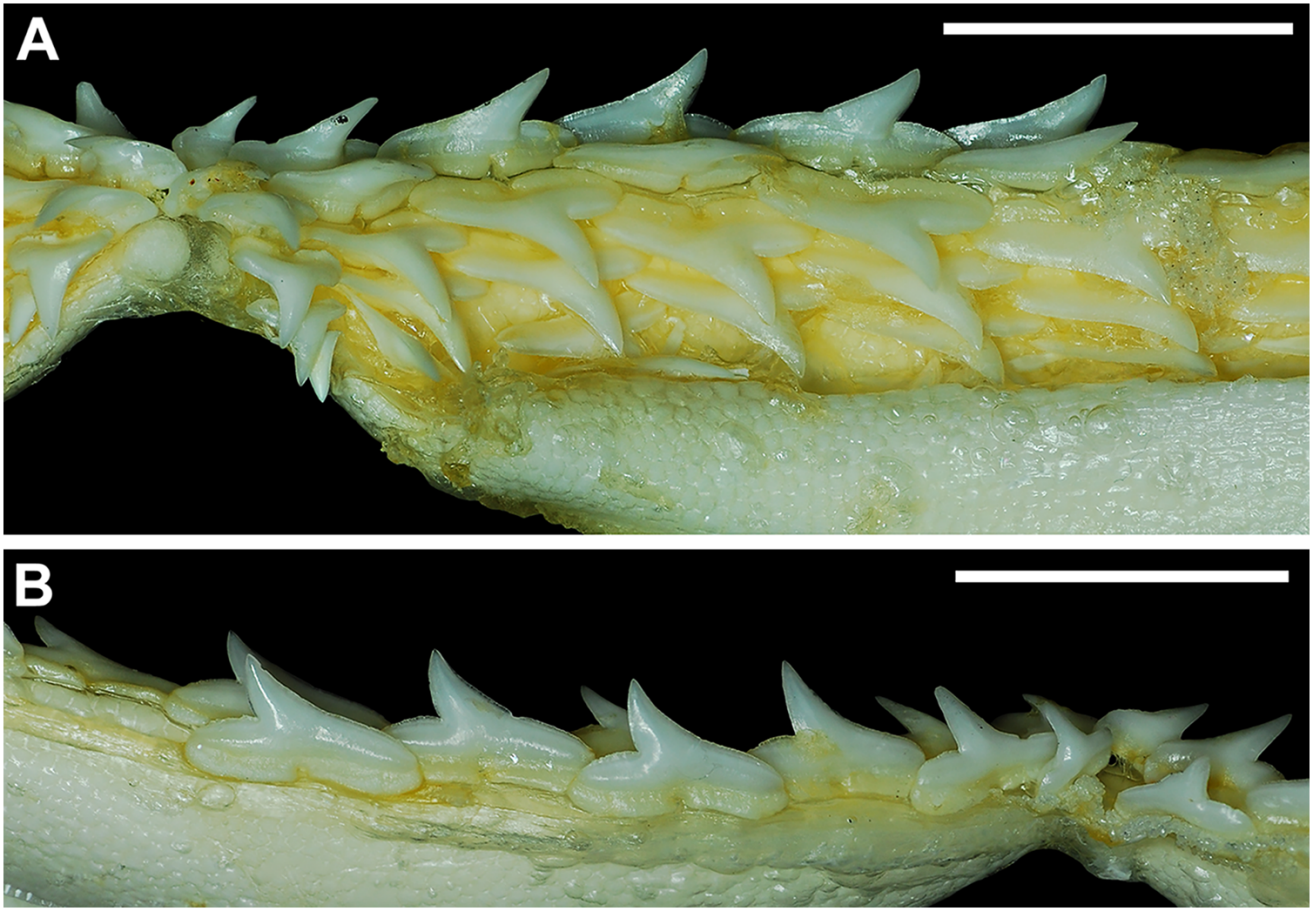
Functional and replacement tooth rows and files and alternate imbricate dentition of *Rhizoprionodon terraenovae*. (A and B) MSC 44461.1, mature female, STL 991 mm. (A) Lingual view of lower right Meckel’s cartilage showing functional and replacement tooth rows. (B) Labial view of lower right Meckel’s cartilage showing alternate imbricated functional tooth row. Scale bars = 2 cm.

Our overall analysis included the detailed examination of 126 *R. terraenovae* jaws that were removed from individuals sampled in the north-central Gulf of Mexico, USA. Of the 122 specimens with completely developed dentitions, the jaws consisted of five rows of teeth arranged in tightly packed tooth files. Each tooth file is comprised of one to two functional teeth and three to four replacement teeth (Fig. 3A). The functional teeth in each file, meaning those that are actively used to acquire and process prey, rotate labially towards the gum line as they develop, and the crown apex is vertical. In living *R. terraenovae* specimens, only the apices of the functional teeth are visible in the mouth, whereas the base of the crown and entirety of the root is obscured by tissue. In contrast, the apices on the replacement teeth point away from the mouth opening, and in live specimens, the entirety of the tooth is obscured by tissue.

The dentition of *R. terraenovae* is imbricated, meaning the mesial and distal edges of the teeth overlap one another as opposed to being separated by a gap. The *R. terraenovae* dentition is classified as alternate-imbricate (*sensu* Cappetta, 2012), where the functional row consists of alternating teeth from the first and second tooth rows. In the functional row, the mesial edge of any given tooth in the first row overlaps the distal edge of the mesially adjacent tooth in the second row, and the distal edge overlaps the mesial edge of the distally adjacent tooth in the second row (Fig. 3B). This arrangement results in the development of essentially two functional rows, although it may appear upon viewing that there is a single sinuous row. This arrangement also indicates that the teeth in the functional row(s) shed at different times, with those within the first row shedding before those in the second row, and so on.

### Tooth file counts for the Meckel’s cartilage and palatoquadrate

**Figure 4:**
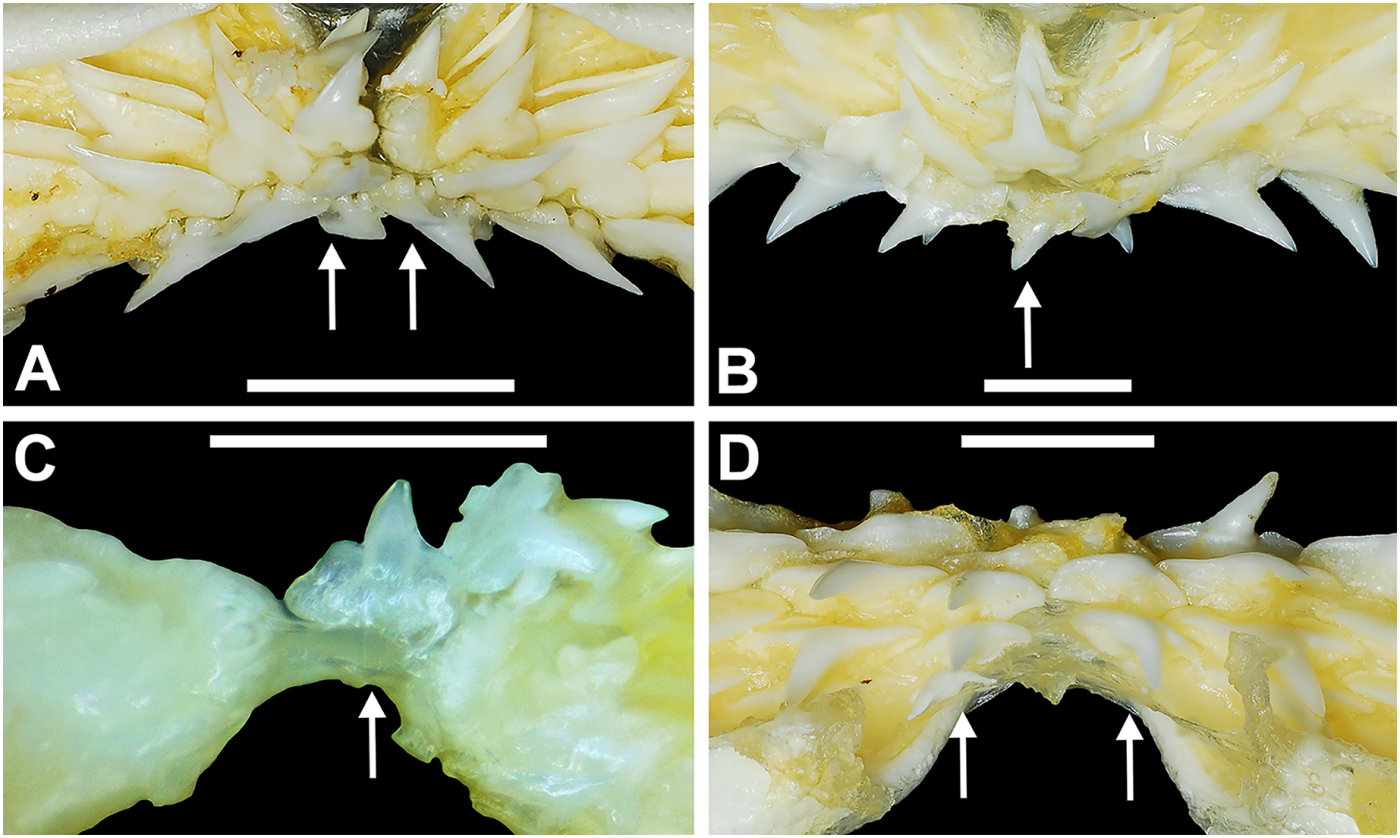
Variation in the number of parasymphyseal and symphyseal files in *Rhizoprionodon terraenovae*. (A) MSC 42650, immature male, STL 645 mm, lingual view of the palatoquadrate showing two files of symphyseal teeth (denoted by arrows), scale bar = 5 mm. (B) MSC 44479, mature male, STL 870 mm, lingual view of the palatoquadrate showing a single file of symphyseal teeth (denoted by an arrow), scale bar = 5 mm. (C) MSC 44482.5, male pup, STL 290 mm, lingual view of the Meckel’s cartilage showing a single parasymphyseal file (denoted by an arrow), scale bar = 2 mm. (D) MSC 44479, mature male, STL 870 mm, lingual view of the Meckel’s cartilage showing two files of parasymphyseal teeth (denoted by arrows), scale bar = 5 mm.

Springer (1964) and Compagno (1984) observed that the total number of teeth within the functional rows on the upper and lower jaws in *R. terraenovae* is variable. Springer (1964) noted that 18 of 20 (90%) specimens in his sample exhibited a total of 25 teeth in the functional row of the palatoquadrate and 24 in the functional row of the Meckel’s cartilage. Compagno (1984) reported similar numbers and noted that there are “usually” 25 teeth in the upper functional row and 24 in the lower. This variability in functional row tooth counts, or number of upper and lower tooth files was also observed in our study sample. Of the 122 specimens examined (the four smallest pups were excluded because they had yet to develop complete dentitions), 97 (79%) showed a total of 25 tooth files in the palatoquadrate and 24 in the Meckel’s cartilage. Of the remaining 25 (21%) jaw sets, the total number of upper and lower tooth files was variable, with specimens exhibiting between 23 and 27 files in either jaw. All but three of the 122 jaws examined exhibited a single upper symphyseal file (Fig. 4B) and two lower parasymphyseal files (one on each side of the symphysis; Fig. 4D). Of the exceptions, specimen MSC 42650 had two upper symphyseal files (Fig. 4A), and specimens MSC 44482.5 and MSC 42654 exhibited a single lower parasymphyseal file (Fig. 4C). A total of nine specimens (7%) in our sample exhibited dental asymmetry, where the total number of tooth files in the right and left halves of the Meckel’s cartilage and/or palatoquadrate was unequal (see Appendix 1.1). Of these, two specimens (MSC 43586 and MSC 44477) showed dental asymmetry in both the Meckel’s cartilage and palatoquadrate, whereas seven exhibited this phenomenon in either the upper or lower jaw (MSC 42658, MSC 42675, MSC 42679, MSC 42683, MSC 44471.2, MSC 44484.2, and MSC 46738). Dental asymmetry and variable dental formulae were observed in both male and female specimens and across all size classes, including *in utero* pups. This indicates that the observed dental asymmetry and variable dental formulae is a result of intraspecific variation as opposed to being related to ontogeny and/or gynandric heterodonty.

Our data on the number of upper and lower tooth files corroborates the observations of Springer (1964) and Compagno (1984), who both noted variability in the dental formula of *R. terraenovae*. Although the number of variable specimens in our sample is more than double (21%) that observed by Springer (1964) (10%), most specimens in both samples (115 of 142; 81%) had an upper dental formula of 12-1-12 and a lower formula of 11-2-11. In our sample, other observed dental formulae for the palatoquadrate included 11-1-12 (*n* = 1), 13-1-13 (*n* = 9), 12-2-12 (*n* = 1), and 12-1-13 (*n* = 4), and others for the Meckel’s cartilage included 10-2-10 (*n* = 1), 11-1-11 (*n* = 2), 11-2-10 (*n* = 3), 11-2-12 (*n* = 3), 12-1-12 (*n* = 1), 12-2-12 (*n* = 3), and 8-2-11 (*n* = 1) (see Appendix 1.1).

### Tooth width, height, H/W ratio, and coronal angle

As part of this study, we attempted to discern if the teeth within *R. terraenovae* dentitions could be quantitatively divided into standardized tooth groups. To do so, we took a series of digital measurements from 466 teeth that were extracted from the Meckel’s cartilage and palatoquadrate of 20 *R. terraenovae* specimens from our sample. To account for potential variation resulting from ontogeny and/or sexual dimorphism, teeth were extracted from 10 pairs of male and female *R. terraenovae* jaws with corresponding STL’s ranging between STL 600 to 1,033 mm (see Material and Methods). Three measurements were taken from the lingual face of each tooth, including the greatest mesiodistal width, greatest apicobasal height, and coronal angle. Values from all measured teeth are presented in Appendices 1.3–1.18.

**Figure 5:**
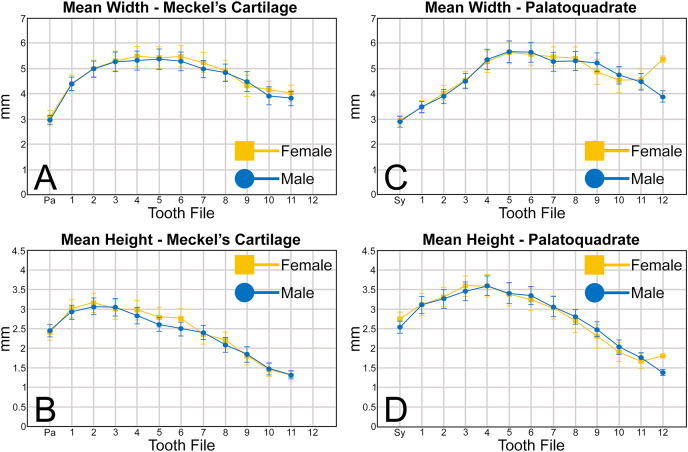
(A–D) Mean greatest width and height of female and male *Rhizoprionodon terraenovae* teeth through ontogeny on the Meckel’s cartilage and palatoquadrate. Abbreviations: Pa, parasymphyseal file; Sy, symphyseal file. Lines above and below plotted values indicate the standard error.

An examination of the mean width of the teeth measured in the Meckel’s cartilage (Fig. 5A) shows that the parasymphyseal tooth is on average the mesiodistally narrowest tooth in the row of both female (mean = 3.05 mm) and male (mean = 2.97 mm) dentitions (Table 1). Moving sequentially towards the commissure, the teeth in both female and male dentitions tend to gradually increase in mean width from positions 1 to 6, with those in positions 3 to 6 generally being the widest teeth in the row (mean = 5.08 to 5.51 mm, Table 1). Beginning with position 7 the teeth tend to gradually decrease in mean width, with those in positions 10 and 11 often being the narrowest non-parasymphyseal teeth in the row (mean = 3.81 to 4.45 mm). The widest tooth measured (7.20 mm) in the Meckel’s cartilage occurred in a large female (MSC 42676) specimen with a length of STL 1,030 mm (Appendix 1.4).

Regarding the mean height of teeth in the Meckel’s cartilage, the shortest teeth generally occur in positions 7 to 12 (mean = 1.30 to 2.39 mm; Fig. 5B, Table 1). The next shortest teeth in the row are those in the parasymphyseal files (mean = 2.39 to 2.44 mm), and the tallest teeth occur in positions 1 and 2 (mean = 2.91 to 3.15 mm). Beginning with position 2, which is generally the tallest tooth in the row, the teeth tend to gradually decrease in mean height the closer they are positioned to the commissure. The tallest tooth measured within the Meckel’s cartilage (4.32 mm) occurred in a large male specimen (MSC 44465) with a length of STL 1,033 mm.

Similar to the Meckel’s cartilage, the mesiodistally narrowest teeth on the palatoquadrate are those in the symphyseal file (mean = 2.89 to 2.96 mm; Fig. 5C, Table 1). Beginning at the symphysis, the teeth gradually increase in mean width to position 5, with the widest teeth generally occurring in positions 4 to 8 (mean = 5.28 to 5.64 mm). Beginning with position 8, the teeth gradually begin to decrease in mean width towards the commissure. The widest tooth measured (7.32 mm) occurred on a large male specimen (MSC 42671) with a length of STL 1,000 mm. One outlier that can be seen in Fig. 5C is the large mean width of the female teeth in position 12 (5.39 mm). As seen in Appendices 1.3–1.6, this large value is due to a sample bias resulting from the lack of teeth that could be removed from the 12^th^ position on any female specimens smaller than STL 940 mm. If such teeth were included in our analysis, the mean value for the 12^th^ position would likely be similar to that seen for males, where the mean width of the teeth gradually decreases after position 8. A similar outlier can be seen in Fig. 5D on the palatoquadrate and is the result of the same sample bias.

On the palatoquadrate, our mean values show that the shortest teeth in the row generally occur in positions 9 to 12 and gradually decrease in height towards the commissure (mean = 1.39 to 2.49 mm; Fig. 5D, Table 1). The next shortest teeth occur in the symphyseal file (mean = 2.53 to 2.76 mm), and the teeth rapidly increase in height from the symphyseal file to positions 3 and 4. The teeth in these latter files are the tallest in the tooth row, with the tallest tooth (4.82 mm) measured on a large female specimen (MSC 42670) with a length of STL 1,001 mm (Appendix 1.7).

**Figure 6:**
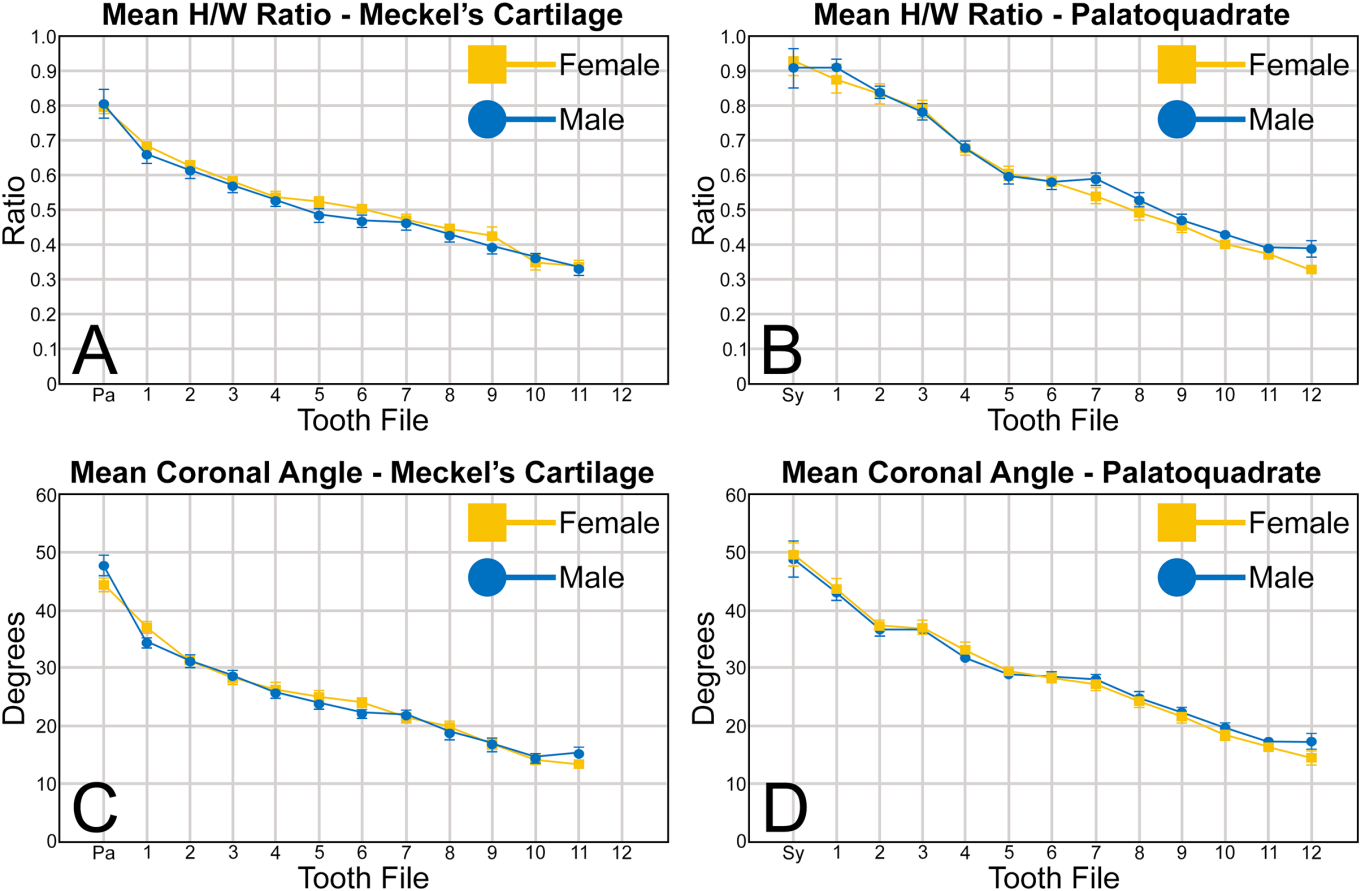
(A–D) Mean height/width (H/W) ratio and coronal angle of female and male *Rhizoprionodon terraenovae* teeth through ontogeny on the Meckel’s cartilage and palatoquadrate. Abbreviations: Pa, parasymphyseal file; Sy, symphyseal file. Lines above and below plotted values indicate the standard error.

When examining the mean H/W ratios for the teeth in both jaws (Figs. 6A, 6B and Table 1), the highest mean ratios occur in the parasymphyseal files of the Meckel’s cartilage (0.80 to 0.81) and the symphyseal file of the palatoquadrate (0.91 to 0.92), indicating that these teeth are almost as tall as they are wide. In all succeeding tooth files, the mean H/W ratios gradually decrease towards the commissure in both the Meckel’s cartilage and palatoquadrate. However, when the mean values are compared between both jaws, the H/W ratios within the palatoquadrate are almost always greater than those in the corresponding files of the Meckel’s cartilage (Figs. 6A, 6B, Table 1). This corroborates the conclusions of Schwartz & Hurst (1996), who determined that the upper teeth in the dentitions of *R. terraenovae* have a greater surface area than those in the lower files.

A similar trend can be seen regarding the mean coronal angle for teeth in both the Meckel’s cartilage and palatoquadrate (Figs. 6C, 6D, Table 1). Across both jaws, the highest coronal angles were measured on teeth in the parasymphyseal files of the Meckel’s cartilage (44.2° to 47.6°) and the symphyseal files in the palatoquadrate (48.8° to 49.6°), and the coronal angle gradually decreases the closer a tooth is positioned to the commissure. When comparing the coronal angle between the upper and lower jaws, the angle is almost always greater in the palatoquadrate when compared to the corresponding files in the Meckel’s cartilage, meaning the main cusp is more distally inclined in the lower files than in the corresponding upper teeth.

When comparing the mean for all values for both the Meckel’s cartilage and palatoquadrate across the sexes, the female values are nearly identical to those of the males through all the size classes and across all the variables (see Figs. 5, 6, Table 1). This indicates that there are little or no quantifiable differences (at least among the various factors evaluated) between the teeth of *R. terraenovae* females and males, corroborating the observations of both Springer (1964) and Compagno (1984, 1988), who both noted the lack of gynandric heterodonty in this taxon. In addition to the lack of quantifiable differences across female and male dentitions, the mean height, width, H/W ratios, and coronal angles appear to remain consistent through ontogeny. Although future studies that utilize techniques like geometric morphometrics may be able to quantify subtle morphological differences between female and male teeth or across growth stages, our set of measurements indicates that the female and male dentitions are nearly identical, and in terms of H/W ratios and coronal angles, immature teeth are essentially the same as those in the corresponding files of mature adults (*i.e*., the mature teeth are simply larger versions of the corresponding immature teeth). These latter statements are corroborated by the small standard errors calculated for the H/W ratios and coronal angles in this study (Fig. 6, Table 1). It should also be noted that the slightly larger standard errors calculated for the mean width and height of the teeth is more of a reflection of the large size range of specimens measured (STL 600 to 1,033 mm) rather than morphological differences between the sexes or growth stages.

### Tooth groups within the *R. terraenovae* dentition

The suite of measurements taken as part of our quantitative analysis allowed us to group the teeth within the *R. terraenovae* dentition into symphyseal/parasymphyseal, anterior, lateral, and posterior tooth groups. Although no single metric (H/W ratio, coronal angle, *etc*.) alone can be used to make these delineations, a combination of the four metrics (*i.e*., mean width, height, H/W ratio, and coronal angle) showed subtle but quantifiable differences between the various tooth groups. It should be noted that the number of teeth described for each tooth group below reflects those with standard dental formulae (*i.e*., 12-1-12/11-2-11), and the number of teeth within each tooth group will vary in those with non-standard dental formulae or asymmetrical dentitions, with additional or fewer teeth potentially occurring within each tooth group. Descriptions of the standardized tooth groups, and metrics that were used herein to delineate them are as follows:

#### Parasymphyseal and symphyseal teeth

Arguably the most distinctive teeth within the *R. terraenovae* dentition are those in the parasymphyseal and symphyseal files. These tooth files are unique because they contain the only teeth in the dentition that do not form within the left or right dental hollows in either the Meckel’s cartilage or palatoquadrate. Rather, two files of parasymphyseal teeth generally occur on the Meckel’s cartilages, one each on the left and right sides of the symphysis. In contrast, on the palatoquadrates a single symphyseal file occurs directly on the symphysis. Teeth in this group are marked by having higher mean H/W ratios (0.80 to 0.81 for parasymphyseal teeth and 0.91 to 0.92 for symphyseal teeth) and coronal angles (44.4° to 47.7° for parasymphyseal teeth and 48.8° to 49.6° for symphyseal teeth) than any of the other teeth in the dentition (Table 1). These teeth are also narrower than all other teeth in the dentition, and with the exception of the last two-to-three tooth positions located closest to the commissure, the parasymphyseal and symphyseal teeth are amongst the shortest in the dentition (see Table 1).

The symphyseal teeth have an erect and triangular main cusp with a mesial cutting edge that is slightly convex and a distal edge that is slightly sinuous. The teeth have both a mesial and distal heel, with the latter being slightly taller and more prominent. The intersection of the mesial cutting edge of the main cusp and mesial heel forms an oblique angle with a continuous cutting edge. The cutting edge on the mesial heel is evenly convex, and it slopes to the mesial edge of the tooth. Distally, the cutting edge on the main cusp is generally separated from the distal heel by a shallow notch. The shape of the distal heel is variable and can be convex, triangular, or subdivided to appear bifurcated. The cutting edges on the mesial and distal sides of the main cusp and the mesial heel are generally smooth, whereas the distal heel can have a smooth, irregular, or serrated cutting edge. Both labial and lingual faces of the tooth crown are convex, but more so lingually. The root extends higher on the lingual face of the crown than labially, and is tallest medially. Labially, the height of the root is rather consistent across the width of the tooth. The lingual face of the root is incised by a deep nutritive groove that forms a conspicuous basal notch. The root lobes are rounded. Although symphyseal teeth appear roughly symmetrical, the mesial and distal sides can be delineated by the shape of the mesial and distal cutting edges, the slight distal inclination of the main cusp, and more prominent distal heel that is separated from the distal cutting edge by a shallow distal notch. Interestingly, the distal edge/heel on symphyseal teeth can occur on either the right or left side, with all replacement teeth within the symphyseal file having the distal edge occurring on the same side.

When *in situ* in the Meckel’s cartilage, the parasymphyseal files (Figs. 4A, 4D) are imbricated, where the mesial or distal edge of the tooth in the first row overlaps the distal or mesial edge of the succeeding tooth (Fig. 3B). This indicates that the parasymphyseal teeth shed at different times, with those in the first row being shed before those in the second. The parasymphyseal teeth have a narrow and triangular main cusp that is slightly distally inclined. The mesial cutting edge is strongly concave and extends onto a convex mesial shoulder. The distal cutting edge is straight and intersects the distal heel at almost a 90° angle. The cutting edge on the distal heel is convex on most parasymphyseal teeth but is angular on some. The cutting edges are generally smooth, but the distal heel is serrated on some teeth. The root is higher on the lingual face, is tallest medially, and is bisected by a deep nutritive groove that forms a basal notch on most teeth. The root lobes are generally rounded but may appear angular on some specimens.

#### Upper and lower anterior teeth

**Figure 7:**
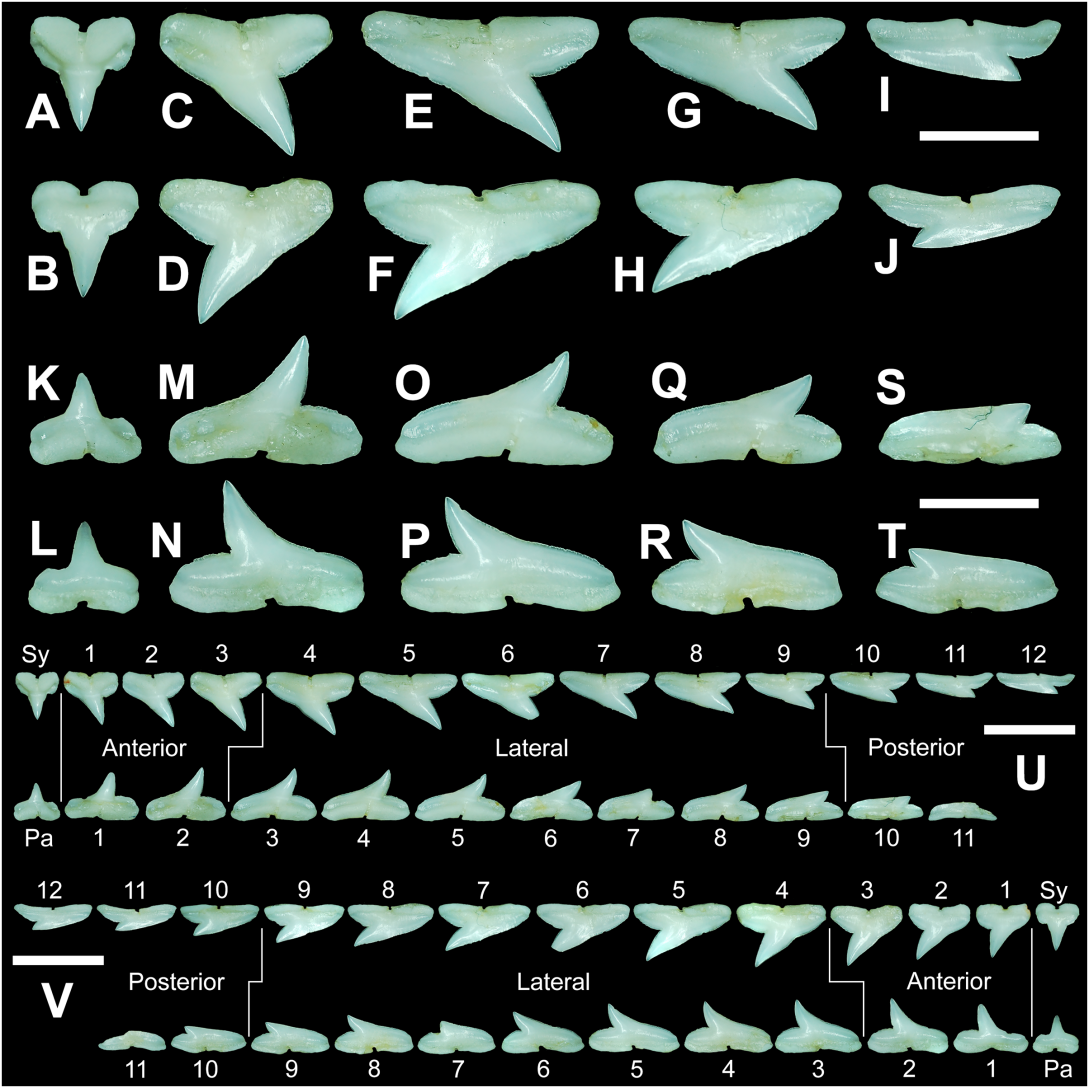
Right dentition of a mature female *Rhizoprionodon terraenovae*. MSC 44461.1, mature female, STL 991 mm. (A and B) Upper symphyseal tooth in (A) lingual and (B) labial views. (C and D) Upper anterior tooth in (C) lingual and (D) labial views. (E and F) Upper lateral tooth in (E) lingual and (F) labial views. (G and H) Upper lateral tooth in (G) lingual and (H) labial views. (I and J) Upper posterior tooth in (I) lingual and (J) labial views. (K and L) Lower parasymphyseal tooth in (K) lingual and (L) labial views. (M and N) Lower anterior tooth in (M) lingual and (N) labial views. (O and P) Lower lateral tooth in (O) lingual and (P) labial views. (Q and R) Lower lateral tooth in (Q) lingual and (R) labial views. (S and T) Lower posterior tooth in (S) lingual and (T) labial views. (U) Right upper and lower dentition in lingual view. (V) Right upper and lower dentition in labial view. Numbers refer to specific tooth files that are numbered consecutively from the symphysis to the commissure. Abbreviations: Pa, parasymphyseal tooth; Sy, symphyseal tooth. Scale bars for A–T = 5 mm. Scale bars for U–V = 1 cm.

The anterior files contain the most anteriorly positioned teeth that form within the left and right dental hollows on the palatoquadrate and Meckel’s cartilage. Within this study, three upper and two lower anterior files are recognized. These teeth generally increase in mean width and height from positions 1 to 3 in the palatoquadrate and 1 to 2 in the Meckel’s cartilage (Table 1, Fig. 7).

The three anterior files in the palatoquadrate (Figs. 7C, 7D, positions 1–3) are herein delineated from those in the lateral files by having much higher mean H/W ratios (0.78 to 0.91, compared to 0.46 to 0.68 for lateral teeth) and coronal angles (36.3° to 43.6°, compared to 21.5° to 33.2° for lateral teeth; Table 1), and these values are lower than those in the parasymphyseal/symphyseal files (see above). Interestingly, upper anterior positions 2 and 3 tend to have nearly identical mean coronal angles (between 36.6° and 37.3°) despite having significantly different mean H/W ratios (0.83 to 0.84 for position 1 and 0.78 to 0.79 for position 2). After position 3, both the mean H/W ratio and coronal angle drop significantly, herein marking the divide with the lateral files (see Table 1). In summary, the upper anterior files are herein defined as those with a combination of mean H/W ratios of between 0.77 and 0.91 and mean coronal angles of between 36° and 44°.

The upper anterior teeth have a well-defined and elongated main cusp that is clearly differentiated from the distal heel. The main cusp becomes gradually more distally inclined from positions 1 to 3 (see Table 1). The lingual crown face of each tooth is strongly convex, whereas the labial face is slightly convex. In mesial or distal views, the teeth have a slight lingual bend. The mesial edge of the crown ranges from straight to slightly concave, and the apex is slightly upturned. The distal cutting edge is evenly convex and is separated from the distal heel by a distinct notch. The shape of the cutting edge on the distal heel is variable and can be evenly convex, triangular, and/or bifurcated. The cutting edges can be serrated, irregular, or smooth. On serrated teeth, the largest serrations occur on the distal heel and the lower half of the mesial cutting edge, but serrations become finer toward the apex on the mesial and distal cutting edges. Short enameloid plications occur along the lingual crown base on a small number of teeth, but these are not considered to be taxonomically significant. The labial face of the root is low and flat, whereas it is higher and more pronounced on the lingual face. A deep nutritive groove divides the root on the lingual face and forms a distinct basal notch. The root lobes are rounded, and the teeth either have a flat basal edge or a shallow and V-shaped interlobe area. Upper anterior teeth can be differentiated from the lower anterior teeth by having a slightly taller and wider main cusp and a less concave mesial cutting edge.

The teeth in the anterior and lateral files of the Meckel’s cartilage have a gradient morphology (*i.e*., gradient monognathic heterodonty), and the divide between the anterior and lateral tooth groups is much more subtle than that in the palatoquadrate. The two lower anterior files are defined herein as those with mean H/W ratios between 0.61 and 0.70 in combination with mean coronal angles between 30° and 38° (Table 1, Figs. 7M, 7N, positions 1–2). In contrast, those in the lower lateral files have mean H/W ratios of between 0.39 and 0.59 and mean coronal angles of between 16° and 28° (see below).

Teeth in the lower anterior files have a triangular main cusp that is clearly separated from the distal heel. The cusp becomes slightly more distally inclined from positions 1 to 2 (mean of 34.3° to 37.1° for position 1 and 31.17° to 31.18° for position 2), which separates them from those in the lateral group, which have a mean coronal angle that does not exceed 29°. The main cusp appears more erect and narrower compared to those in the corresponding upper files. This is due to the mesial edge of the crown being strongly convex medially, straight apically, and convex at the crown base. The distal cutting edge of the main cusp is evenly convex and is separated from the distal heel by a conspicuous notch. The shape of the cutting edge on the distal heel is variable and can be rounded, triangular, or bifurcated. The cutting edges can be serrated, irregular, or smooth. On serrated teeth, the largest serrations occur on the distal heel and lower half of the mesial edge. The serrations on the mesial and distal cutting edges of the main cusp fine towards the apex. Short enameloid plications occur at the lingual crown base on a small number of teeth. The lingual face of the crown is strongly convex, whereas the labial face is slightly convex. In mesial or distal views, the teeth have a slight lingual bend. The root lobes are rounded, and the root face is higher lingually than labially. Lingually, the root is bisected by a deep nutritive groove that forms a distinct basal notch. The basal edge can be sinuous or have a shallow V-shaped interlobe area.

#### Upper and lower lateral teeth

The gross morphology of upper and lower lateral teeth is similar to those in the anterior positions but differs by having lower mean H/W ratios and coronal angles (Table 1). In the palatoquadrate, the divide between the anterior and lateral tooth groups is much easier to delineate compared to the Meckel’s cartilage. In the palatoquadrate, the divide between the anterior and lateral tooth groups generally occurs between positions 3 and 4, and six lateral files are generally present (Figs. 7U, 7V, positions 4–9). The lateral teeth are herein defined as those having a combination of mean H/W ratios between 0.45 and 0.68 and coronal angles between 21° and 33° (Table 1). In contrast, the upper anterior teeth have a combination of mean H/W ratios of between 0.77 and 0.91 and mean coronal angles of between 36° and 44° (see Table 1). The upper lateral teeth differ from those described herein as posterior teeth by having higher mean H/W ratios (0.45 to 0.68 for laterals compared to 0.32 to 0.42 for posteriors) and coronal angles (between 21° and 33° for laterals compared to 14° to 20° for posteriors). Seven lateral files are generally present within the Meckel’s cartilage and are defined herein as those with mean H/W ratios of between 0.39 and 0.58 in combination with mean coronal angles of between 16° to 29°. In contrast, those in the lower anterior files have mean H/W ratios between 0.61 and 0.70 in combination with mean coronal angles between 30° and 38°, and those in the lower posterior files have mean H/W ratios of between 0.33 and 0.36 and mean coronal angles between 13° and 15°. The separation of the lower lateral and anterior tooth groups can be best delineated by the lateral teeth having H/W ratios below 0.6 in combination with coronal angles below 30°.

In both the upper and lower lateral files, the morphology is gradient between the first and last lateral teeth, as they generally become narrower and shorter the closer they are located to the commissure (*i.e*., lower H/W ratios, Fig. 5), and the coronal angle generally decreases. The upper lateral teeth (Figs. 7E–7H, 7U, 7V, positions 4–9) have a main cusp that is well-delineated from the distal heel. Depending on the tooth position, the mesial edge can be slightly concave (Figs. 7U, 7V, positions 4 to 6) or slightly convex (Figs. 7U, 7V, positions 7–9). The main cusp is tall and triangular and has a slightly upturned apex. The distal cutting edge is convex and is separated from the distal heel by a distinct notch. The labial face of the crown is generally flat, whereas the lingual face is strongly convex. The shape of the cutting edge on the distal heel is variable and can be rounded, triangular, or bifurcated. The nature of the various cutting edges is also variable and can be smooth, irregular, or serrated. When present, the largest serrations occur on the distal heel and at the base of the mesial cutting edge. The serrations on the mesial and distal cutting edges fine towards the apex. The root is higher on the lingual face and is incised by a deep nutritive groove that forms a distinct basal notch. The root lobes are generally rounded, but they may appear more angular on some of the more posteriorly positioned lateral teeth (*i.e*., those in positions 8 and 9).

The lower lateral teeth (Figs. 7O–7R, 7U, 7V, positions 3–9) are similar in overall morphology to those in the upper lateral files but differ by having a slightly shorter and narrower main cusp, and the main cusp is more distally inclined than those in the corresponding upper positions. In addition, the root lobes on the lower teeth are also more evenly rounded than those in the upper lateral positions.

As noted above, the upper and lower lateral teeth are morphologically similar to those in the anterior positions, but those in the upper lateral files (Figs. 7E–7H) differ by having a straighter mesial cutting edge, less upturned apex, and a more distally inclined main cusp. Also, as shown by the gradual decrease in H/W ratio across the upper lateral row (Fig. 6B), the teeth are noticeably wider than tall (see Table 1). The lower lateral teeth (Figs. 7O–7R) differ from those in the lower anterior files by having a less upturned apex, a less convex mesial edge, and a more distally inclined main cusp.

#### Upper and lower posterior teeth

The last three positions in the palatoquadrate (Figs. 7U, 7V, positions 10–12) and last two positions in the Meckel’s cartilage (Figs. 7U, 7V, positions 10–11) are herein defined as posterior teeth. These teeth differ from those in the lateral positions by having much lower mean H/W ratios and coronal angles (Table 1). The three files of teeth defined herein as upper posterior have a mean H/W ratio of between 0.32 to 0.42, in contrast to the much taller upper lateral teeth that have a mean H/W ratio of between 0.45 to 0.69 (Table 1). In addition, the mean coronal angle on the upper posterior teeth ranges from 14° to 19.6°, whereas those on the upper lateral teeth range from 21° to 33°. On the Meckel’s cartilage, the posterior teeth have a mean H/W ratio of 0.33 to 0.36 and mean coronal angles of between 13° and 15°. In contrast, the lower lateral teeth have a H/W ratio of between 0.39 to 0.59 and a mean coronal angle of between 16° and 29°.

Within both the palatoquadrate and Meckel’s cartilage, the divide between the lateral and posterior tooth positions occurs between positions 9 and 10, where a significant decrease in greatest height can be observed (see Table 1). The teeth in the posterior positions are unique by being, on average, the shortest in either tooth row (Figs. 5B, 5D) and having the lowest H/W ratios (Table 1) because they are often more than three times wider than they are tall. The posterior teeth are similar in overall morphology to those in the lateral positions but differ by having a rather diminutive main cusp. On some posterior teeth, the main cusp is not developed, and the crown is represented only by a continuous convex cutting edge. As reflected by the low coronal angles shown in Table 1, the posterior teeth have a much more distally inclined main cusp than those in the lateral files. The upper posterior teeth (Figs. 7I, 7J, 7U, 7V, positions 10–12) can be differentiated from those in the lower posterior files (Figs. 7S, 7T, 7U, 7V, position 10–11) by having a slightly taller and wider main cusp and a noticeably longer distal heel.

### Monognathic and dignathic heterodonty

Our analysis shows that monognathic (including gradual and disjunct) and dignathic heterodonty occur within the dentition of *R. terraenovae*. Monognathic heterodonty, or morphological differences between the teeth within a single tooth row, is evident in both the palatoquadrate and Meckel’s cartilage (Figs. 7U, 7V). The teeth occurring in the left and right dental hollows of the palatoquadrate are similar in morphology but differ by gradually becoming narrower after position 5 (Fig. 5C), progressively shorter after position 4 (Fig. 5D), gradually smaller H/W ratios across the entire tooth row (Fig. 6B), and the main cusp becomes more distally inclined (*i.e*., smaller coronal angle) the closer a tooth is located to the commissure (Fig. 6D). Furthermore, beginning with position 7 (Figs. 7U, 7V) the mesial cutting edge becomes progressively more convex, the main cusp becomes noticeably and progressively shorter beginning at position 5, and the distal heel becomes noticeably more elongated in posterior positions 11 and 12. Similar monognathic heterodonty is observed on the Meckel’s cartilage, where the width gradually increases after position 5 (Fig. 5A), the height gradually decreases after position 2 (Fig. 5B), and the H/W ratio (Fig. 6A) and coronal angle (Fig. 6B) both progressively decrease across the tooth row. In addition, the main cusp becomes shorter and more distally inclined the closer a tooth is positioned to the commissure (Figs. 7U, 7V), and the mesial shoulder is more elongated on the teeth in the posterior positions (positions 10 and 11).

Disjunct heterodonty, meaning a dramatic change in tooth morphology across a tooth row, can be observed in both the palatoquadrate and Meckel’s cartilage by the unique morphology of the symphyseal and parasymphyseal teeth, respectively (Figs. 7A, 7B, 7K, 7L). These teeth differ morphologically from those that form within the dental hollows (see above) and have higher mean H/W ratios and coronal angles (Fig. 6, Table 1) than any of the other teeth in the dentition.

Dignathic heterodonty, meaning differences between the teeth in the upper and lower jaws, is also apparent in *R. terraenovae* (Figs. 7U, 7V). The teeth in the palatoquadrate have a higher mean H/W ratio than those in the corresponding positions in the Meckel’s cartilage (Figs. 6A, 6B), and the mean height on the teeth in positions 2–6 are taller than any of those in the Meckel’s cartilage (Figs. 5B–5D, Table 1). In addition, the base of the main cusp is wider on teeth in the upper files, a phenomenon that was observed by Schwartz & Hurst (1996), whose morphometric analysis showed that the labial face of upper teeth has more surface area compared to those in the lower files. Moreover, the labial face of the crown is more convex on teeth in the lower files, especially on those in positions 1 to 5 (Figs. 7U, 7V). Additionally, the mesial cutting edge is comparatively straighter on upper teeth and more conspicuously concave on those in lower files. Furthermore, the apex is more upturned on the lower more anteriorly positioned lateral teeth compared to teeth in the corresponding upper files, and the lower posterior teeth (Figs. 7U, 7V, positions 10 and 11) have an elongated mesial heel, whereas the upper posterior teeth have an elongated distal heel (Figs. 7U, 7V, positions 11 and 12). Lastly, the symphyseal and parasymphyseal tooth morphologies differ greatly (see above descriptions; Figs. 7A, 7B, 7K, 7L).

### Ontogenetic heterodonty

Our analysis also documented ontogenetic and gynandric heterodonty within the dentitions of *R. terraenovae*. Although Herman, Hovestadt-Euler & Hovestadt (1991) did not observe ontogenetic heterodonty within their sample of *Rhizoprionodon* teeth, Springer (1964) and Compagno (1984, 1988) noted that serrations were present on larger/mature *R. terraenovae* teeth, indicating a degree of ontogenetic heterodonty within the species. Our observations corroborate these latter observations, but our study also revealed that the ontogenetic/morphological change within the *R. terraenovae* dentition is much more drastic than previously documented. Our study concentrated on recording the ontogenetic/morphological changes observed on three regions of each tooth within the functional rows of the Meckel’s cartilage and palatoquadrate. The tooth regions we examined include the mesial and distal edges of the main cusp and the distal heel (Figs. 2D–2E), and the ontogenetic/morphological changes we observed for each are described in detail below.

#### Development of individual teeth, tooth rows, and tooth files

**Figure 8:**
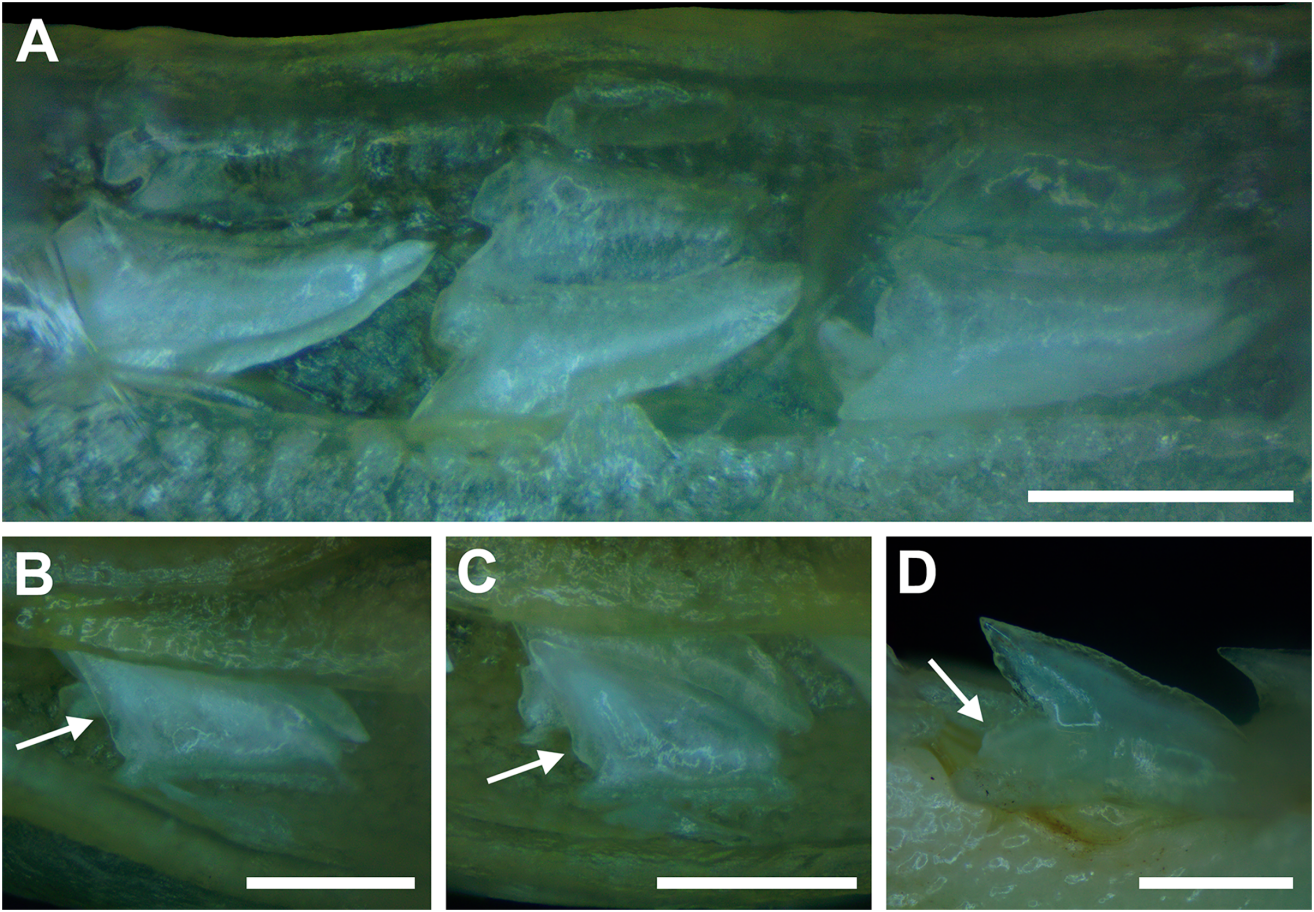
Development of *Rhizoprionodon terraenovae* tooth rows, files, and distal heel. (A–C) MSC 42685.2, *in utero* male pup, STL 190 mm. (A) Lingual view of left Meckel’s cartilage showing *in situ* lateral teeth (in labial view) arranged into two rows. (B) *In situ* upper lateral tooth in labial view showing a straight distal edge (denoted by the arrow). (C) *In situ* upper lateral tooth in labial view showing a distal protrusion (denoted by the arrow). (D) MSC 42685.5, *in utero* male pup, STL 330 mm; *in situ* upper lateral tooth in labial view showing a developed distal heel (denoted by the arrow). Scale bars = 1 mm.

The dentition of *R. terraenovae* is generally comprised of five tooth rows in both the Meckel’s cartilage and palatoquadrate. However, four *in utero* pups in our sample, including two females (MSC 42685.4 and MSC 42685.6) and two males (MSC 42685.2 and MSC 42685.3), exhibit dentitions that were still developing. These specimens provided us with unique insights into the *in utero* development of *R. terraenovae* dentitions, as well as information on the development of the individual teeth. The pups ranged between STL 130 to 190 mm and are the smallest specimens in our sample. All four jaws have dentitions consisting of only two tooth rows that are loosely arranged into files (Fig. 8A). Of the teeth in these rows, a majority are in the replacement position (*i.e*., apices turned away from the opening of the mouth), with only a few occupying the functional position (*i.e*., apices pointed toward the mouth opening). Furthermore, small gaps occur between the teeth because all five tooth rows have yet to develop. This indicates that the alternate-imbricate organization of *R. terraenovae* dentitions does not form until more than two tooth rows are developed. Of the two rows of teeth occurring in these jaws, the more labially positioned teeth are significantly smaller than those in the preceding row (Fig. 8A). This suggests a period of rapid growth for the pups that is in turn reflected in the individual rows and teeth.

Of the pups in the STL 130 to 190 mm size class, the teeth in the jaws were in various developmental stages, indicating that the initial development of teeth does not happen uniformly across the tooth row. Partially formed enameloid crowns are present on all the teeth, but nearly all lack a dentine root (Fig. 8A). Rather, the individual tooth crowns appear to be anchored to the jaw by a thin layer of transparent tissue. As the tooth develops, this connective tissue appears to mineralize into a less transparent and shallow bar-like root located just below the crown base (Figs. 8B and 8C). As reported in previous studies on galeomorph tooth development (*i.e*., Welton & Farish, 1993; Cappetta, 2012; Smith et al., 2012), the enameloid tooth crown forms before it is filled with dentine, and the formation of the dentine root often continues to develop until the tooth reaches the functional position. These phenomena are corroborated by our observations on the dentitions of these *in utero* pup specimens.

Of the individual teeth within the dentitions of the STL 130 to 190 mm size class, the distal heel was in various stages of development, indicating that the main cusp develops before the distal heel. In the earliest stage of tooth development, the distal heel is absent, and the distal edge of the tooth is represented only by the relatively straight distal edge of the main cusp (Fig. 8B). Formation of the distal heel begins with a small protrusion that develops at the base of the distal edge of the crown (Fig. 8C). As the root begins to mineralize along the crown base, the distal edge of both the root and crown base begin to project distally, and these features eventually combine to form a low distal shoulder. As the tooth develops, the enameloid in the medial portion of distal shoulder increases in height until it forms a convex distal heel (Fig. 8D). On all the teeth observed in this size class, the mesial and distal cutting edges are smooth, and the distal heel always has a convex (rounded) and smooth cutting edge.

Although there is a small gap in our dataset of *in utero* specimens between STL 190 and 250 mm, all pups measuring STL 250 mm or longer had complete dentitions (*i.e*., five tooth rows on both the Meckel’s cartilage and palatoquadrate) and nearly all the individual teeth in the functional rows had a fully formed crown and root. This indicates that the development of all five tooth rows within the Meckel’s cartilage and palatoquadrate, and the nearly complete development of the individual teeth, occurs between STL 190 and 250 mm. Interestingly, the distal heel on a small number of teeth was still developing in some pup individuals up to STL 310 mm, and additional ontogenetic development of the teeth could be observed on specimens beginning at STL 250 mm.

#### Ontogenetic development of the distal heel

**Figure 9:**
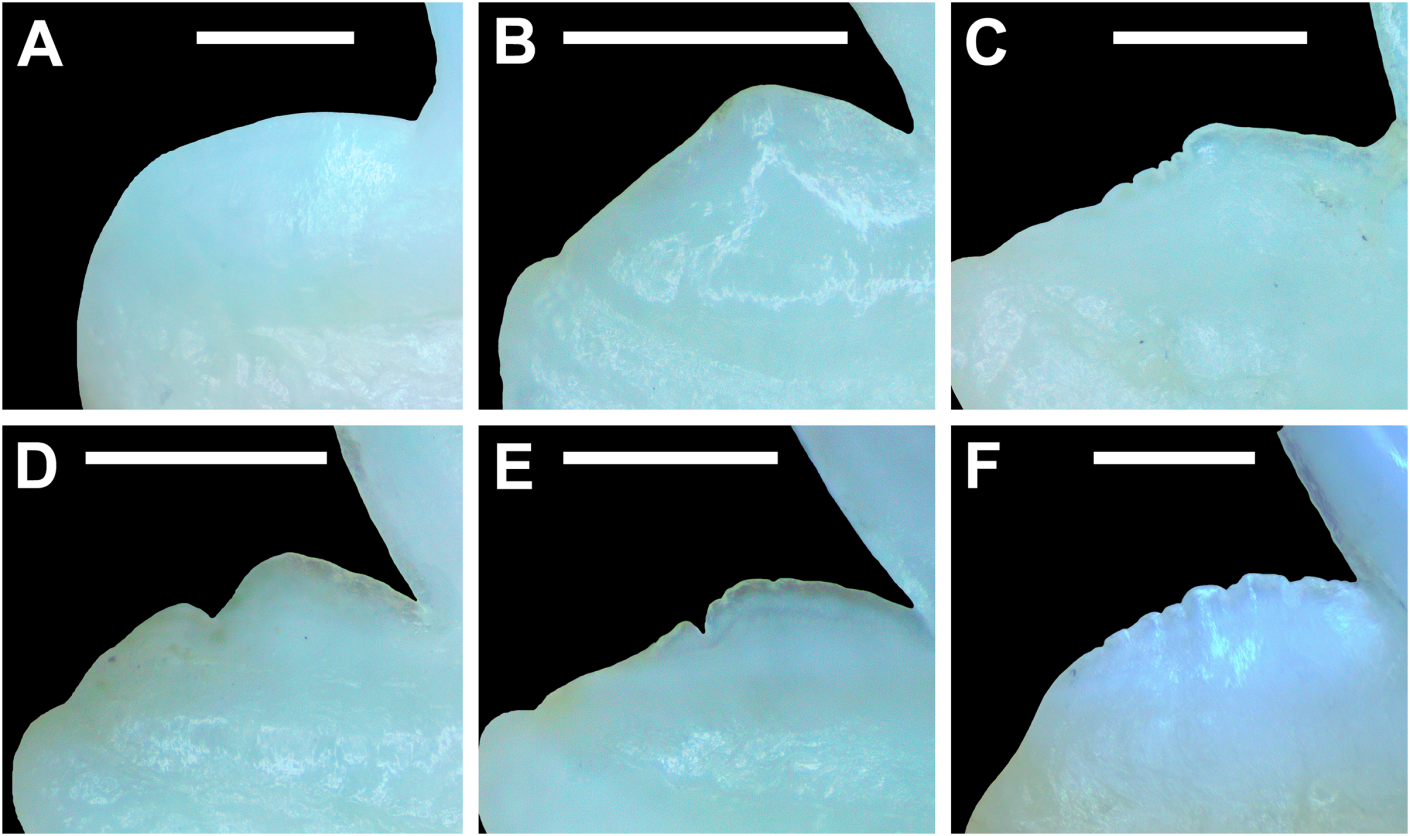
Distal heel morphologies on *Rhizoprionodon terraenovae* teeth. (A) Rounded distal heel with smooth cutting edge, MSC 44479, mature male, STL 870 mm. (B) Triangular distal heel with smooth cutting edge, MSC 44464, immature male, STL 665 mm. (C) Triangular distal heel with partially serrated cutting edge, MSC 42650, immature male, STL 645 mm. (D) Bifurcated distal heel with smooth cutting edge, MSC 42638, female, STL 675 mm. (E) Bifurcated distal heel with irregular cutting edge, MSC 42664, immature male, STL 640 mm. (F) Rounded distal heel with serrated cutting edge, MSC 44479, mature male, STL 870 mm. Scale bars = 1 mm.

Of the three regions of the tooth crown that we evaluated, the distal heel undergoes the most morphological change through ontogeny. As seen in Appendices 1.19–1.22, the morphology of the distal heel varies greatly across the tooth row, with rounded (Figs. 9A and 9F), triangular (Figs. 9B and 9C), and bifurcated (Figs. 9D and 9E) distal heel morphologies often occurring within the same jaw. Although our raw dataset shows a tremendous amount of morphological variation across our sample, when the number of rounded, triangular, and bifurcated teeth are tabulated per row and plotted with a polynomial curve, a generalized morphological trend shows that the shape of the distal heel transitions from rounded to triangular to bifurcated, then back to rounded as the shark matures.

**Figure 10:**
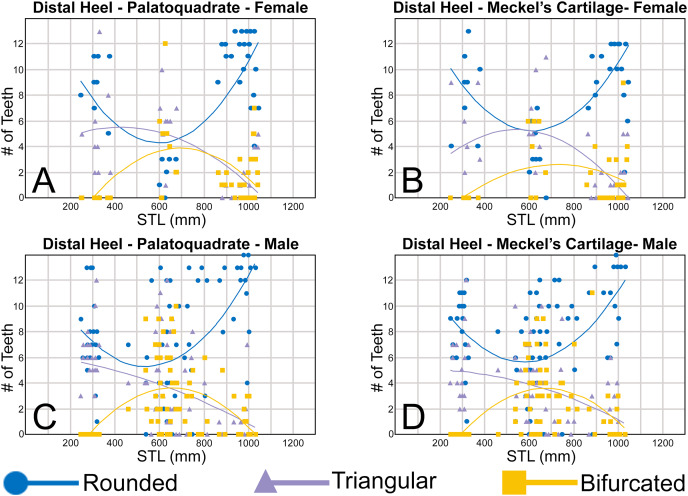
(A–D) Plots and polynomial trends of the morphology of the distal heel on female and male *Rhizoprionodon terraenovae* teeth through ontogeny on the Meckel’s cartilage and palatoquadrate. Tooth numbers represent the number of teeth with a particular characteristic (*i.e*., rounded, triangular, or bifurcated distal heel) that occur within the functional row (left or right) of the Meckel’s cartilage or palatoquadrate. STL, stretched total length.

Of the four pup specimens observed in the STL 130 to 190 mm size class (MSC 42685.2, MSC 42685.3, MSC 42685.4, and MSC 42685.6), all had incompletely developed dentitions in that only two tooth rows had developed. Although not all the teeth in these jaws were fully developed, the shape was rounded on every tooth with a developed distal heel, indicating the initial morphology of this structure is rounded (Fig. 8D). Of specimens in the STL 250 to 300 mm size class, most of the teeth in the row have a rounded distal heel, but many with a triangular distal heel also occur in each row (Fig. 10). Our polynomial trends show that across ontogeny in both females and males, the number of teeth with a rounded distal heel steadily decreases in specimens between STL 250 to 600 mm, only to later increase in number between STL 600 and 1,040 mm (with the most mature specimens on average having the most teeth per row with a rounded distal heel). In female jaws, the number of teeth with a triangular distal heel increases in individuals between STL 250 and 600 mm before steadily declining in number in larger, more mature specimens (Figs. 10A, 10B). In contrast, male specimens show a steady decline in the number of teeth with a triangular distal heel in all size classes after STL 250 mm (Figs. 10C, 10D).

In our sample, the first indication of a bifurcated distal heel was observed on a neonate male specimen of STL 462 mm (see Appendices 1.19–1.22). Although there is a small gap in our dataset of male specimens between STL 330 and 462 mm, this characteristic is absent in all male pup jaws we observed, indicating that the development of a bifurcated distal heel occurs shortly after birth in males, but not while *in utero*. In females, the first occurrence of a bifurcated distal heel on a tooth was observed on a specimen of STL 600 mm. Similar to the males, this characteristic was absent in all female pup jaws we observed (STL 250 to 378 mm) but was also absent within two female neonate specimens measuring STL 371 and 378 mm, respectively. This further corroborates our observation that bifurcation of the distal heel occurs shortly after birth, but not while *in utero*. Overall, our data indicates that males acquire teeth with bifurcated distal heels before females, likely accounting for tooth rows with a smaller number of teeth with a triangular distal heel in the STL 400 to 800 mm size class when compared to that of females (Fig. 10). However, in both females and males, the number of teeth with bifurcated and triangular distal heels steadily declines in specimens between STL 700 and 1,040 mm, coinciding with a large increase in teeth with a rounded distal heel within the same size range. This indicates that the shape of distal heel tends to transition back to its initial rounded morphology in most teeth as the shark reaches full maturity.

**Figure 11:**
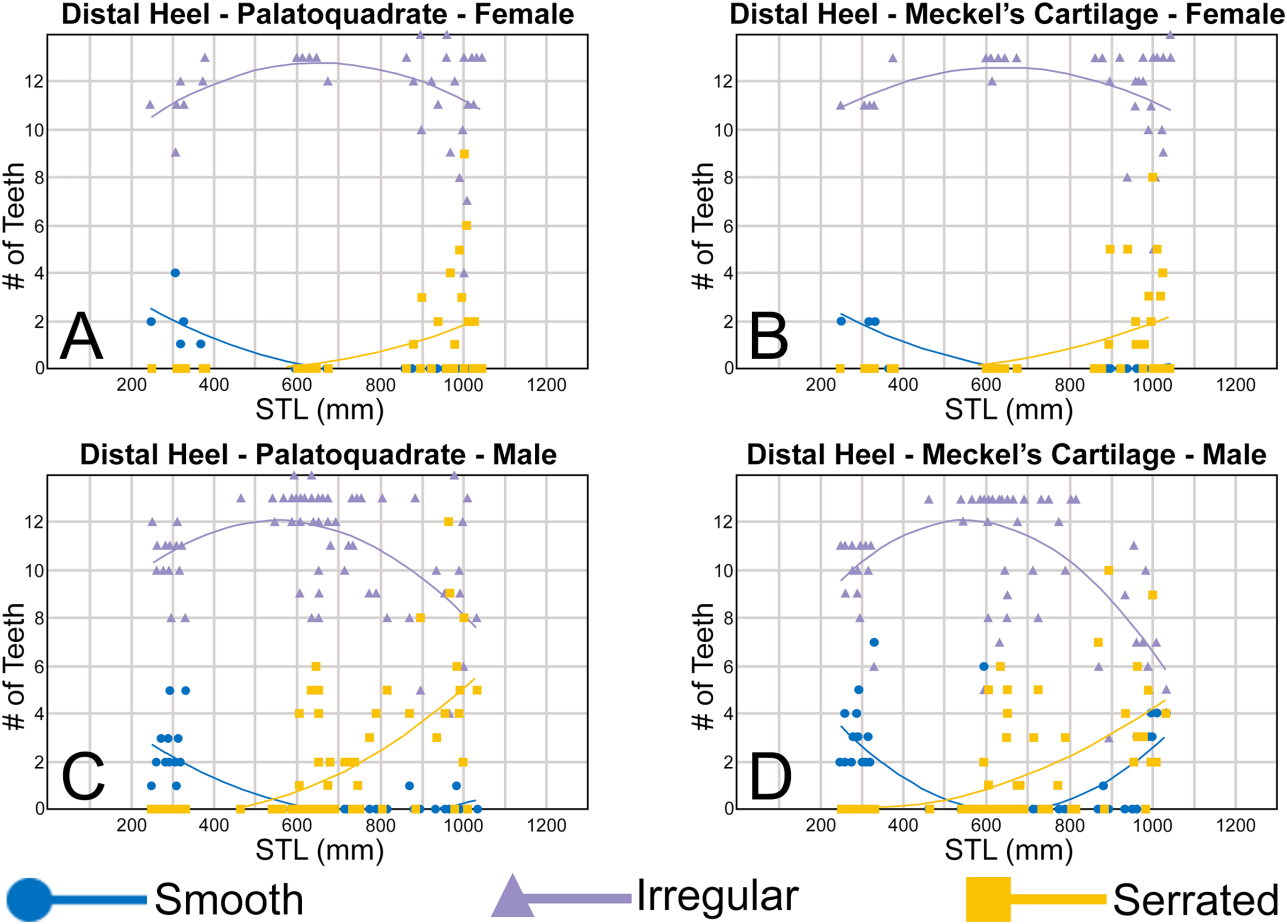
(A–D) Plots and polynomial trends of the nature of the cutting edge on the distal heel on female and male *Rhizoprionodon terraenovae* teeth through ontogeny on the Meckel’s cartilage and palatoquadrate. Tooth numbers represent the number of teeth with a particular characteristic (*i.e*., smooth, irregular, or serrated distal heel) that occur within the functional row (left or right) of the Meckel’s cartilage or palatoquadrate. STL, stretched total length.

On the four smallest pups in our sample (STL 130 to 190 mm), all teeth with a developed distal heel had a smooth cutting edge (thus indicating the edge starts as smooth). However, our data shows that of female and male specimens in the STL 250 to 400 mm size class, a larger proportion of teeth in each row has an irregular cutting edge on the distal heel as opposed to smooth (Fig. 11), indicating that the development of irregular cutting edges develops *in utero*. In both females and males, the number of teeth with an irregular distal heel continues to increase in specimens up to the STL 600 to 650 mm size class, before steadily decreasing in larger specimens. The increase in the number of irregular distal heels up to the STL 600 to 650 mm size class corresponds with a decrease in the number of teeth with a smooth distal heel in specimens between STL 250 and 600 mm, indicating that teeth with a smooth distal heel are being replaced with those with irregular cutting edges (Fig. 11).

**Figure 12:**
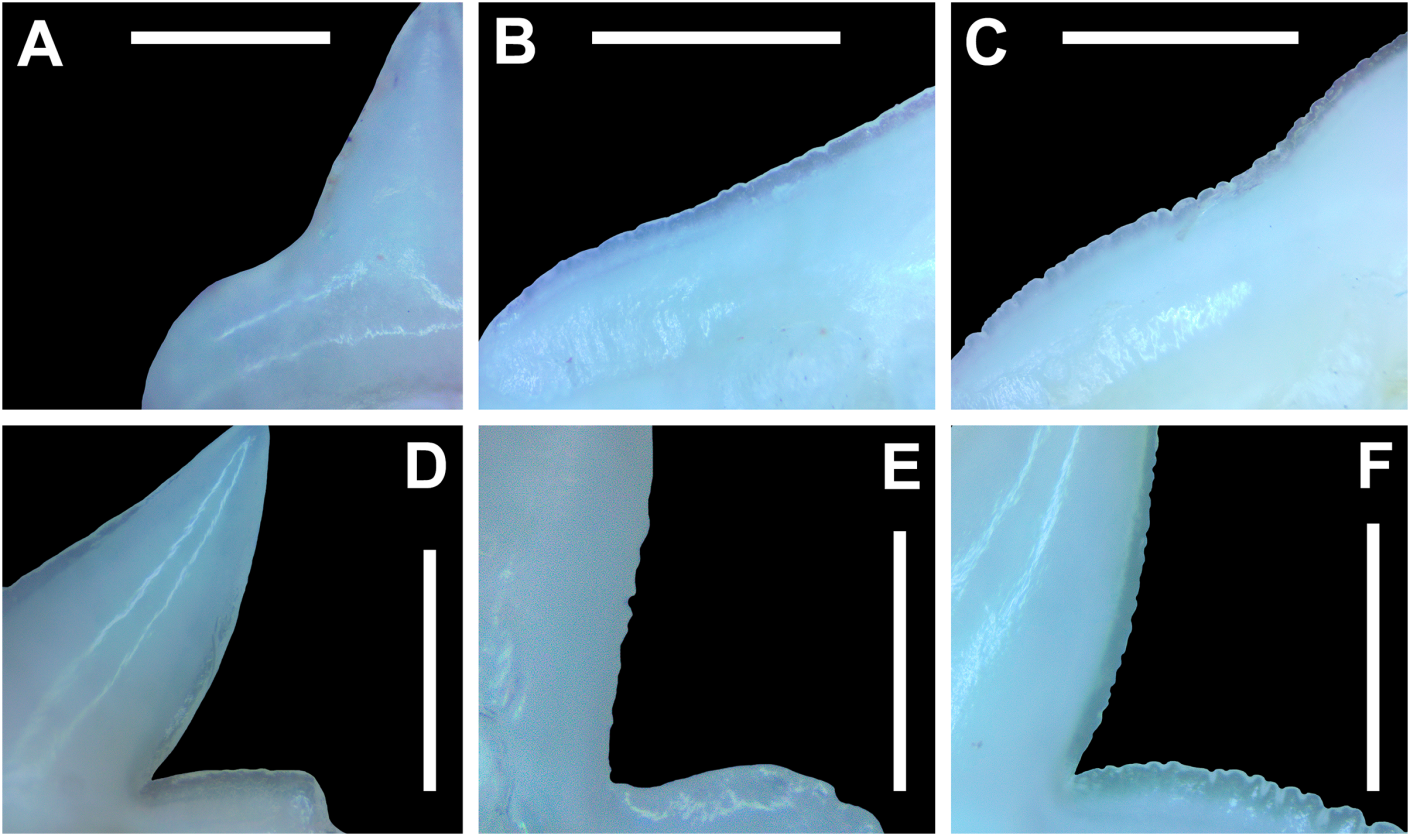
Morphology of the mesial and distal cutting edges on *Rhizoprionodon terraenovae* teeth. (A) Smooth mesial cutting edge, MSC 42650, immature male, STL 695 mm. (B) Irregular mesial cutting edge, MSC 42664, immature male, STL 640 mm. (C) Serrated mesial cutting edge, MSC 44461.1, female, STL 991 mm. (D) Smooth distal cutting edge, 42664, immature male, STL 640 mm. (E) Irregular distal cutting edge, MSC 42650, immature male, STL 695 mm. (F) Serrated distal cutting edge, MSC 44461.1, female, STL 991 mm. Scale bars = 0.5 mm.

Across both sexes, there is a noticeable trend where the number of teeth having a combination of a bifurcated distal heel and irregular cutting edge gradually increases until approximately the STL 550 to 600 mm size class (Figs. 11, 12). This combination of a bifurcated distal heel and irregular cutting edge can be interpreted as a precursor to the development of serrations, as the first serrated distal heel appears on a female specimen of STL 879 mm (MSC 44462) and male specimen of STL 595 mm (MSC 44460) (see Fig. 11, Appendices 1.23–1.26). This accounts for the disparity seen between the female and male trends in Fig. 11, where the number of teeth with an irregular distal heel decreases at a more rapid rate in males than females and coincides with the increase in the number of teeth with a serrated distal heel, which is seen in larger numbers, and in a smaller size class, in males than in females. This supports our observations that teeth with an irregular distal heel are replaced through ontogeny by those that are serrated. On female teeth, most of the distal heels change from triangular or bifurcated to rounded starting between STL 600 and 800 mm, just prior to the first occurrence of serrations on the distal heel (STL 879 mm). On male specimens between STL 600 and 800 mm, the shape of the distal heel is more varied and can be rounded, bifurcated, or triangular, despite the occurrence of serrations on some teeth (Figs. 11, 12). Interestingly, on male specimens between STL 800 and 1,050 mm, the number of teeth with a smooth distal heel increases in the Meckel’s cartilage, whereas there is little or no increase in the number of smooth distal heels in this size class in either female jaw, and only a minute increase can be seen around the STL 1,000 mm size class on the palatoquadrate in male specimens (Fig. 11).

#### Ontogenetic development of the mesial and distal cutting edges

**Figure 13:**
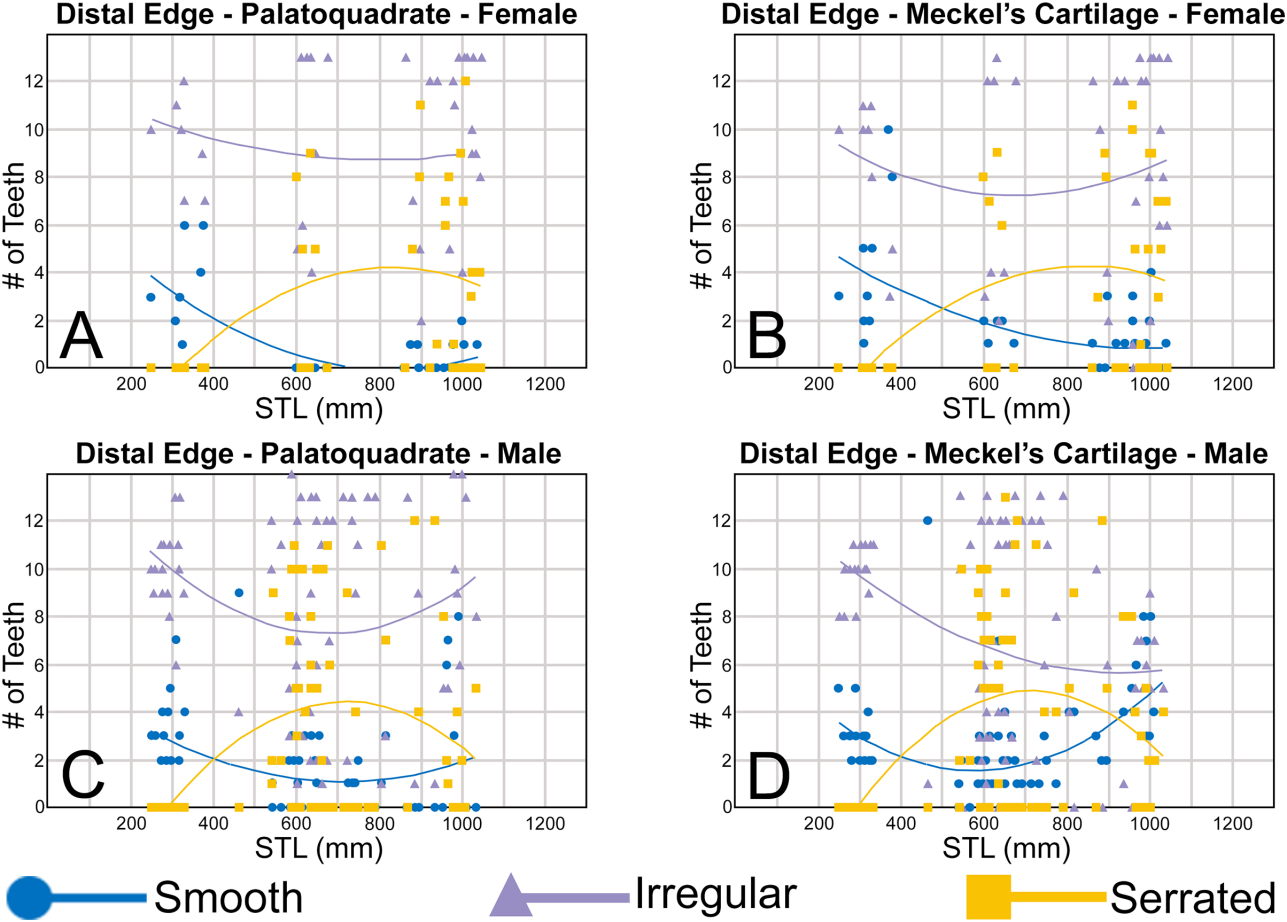
(A–D) Plots and polynomial trends of the nature of the distal cutting edge on female and male *Rhizoprionodon terraenovae* teeth through ontogeny on the Meckel’s cartilage and palatoquadrate. Tooth numbers represent the number of teeth with a particular characteristic (*i.e*., smooth, irregular, or serrated distal edge) that occur within the functional row (left or right) of the Meckel’s cartilage or palatoquadrate. STL, stretched total length.

**Figure 14:**
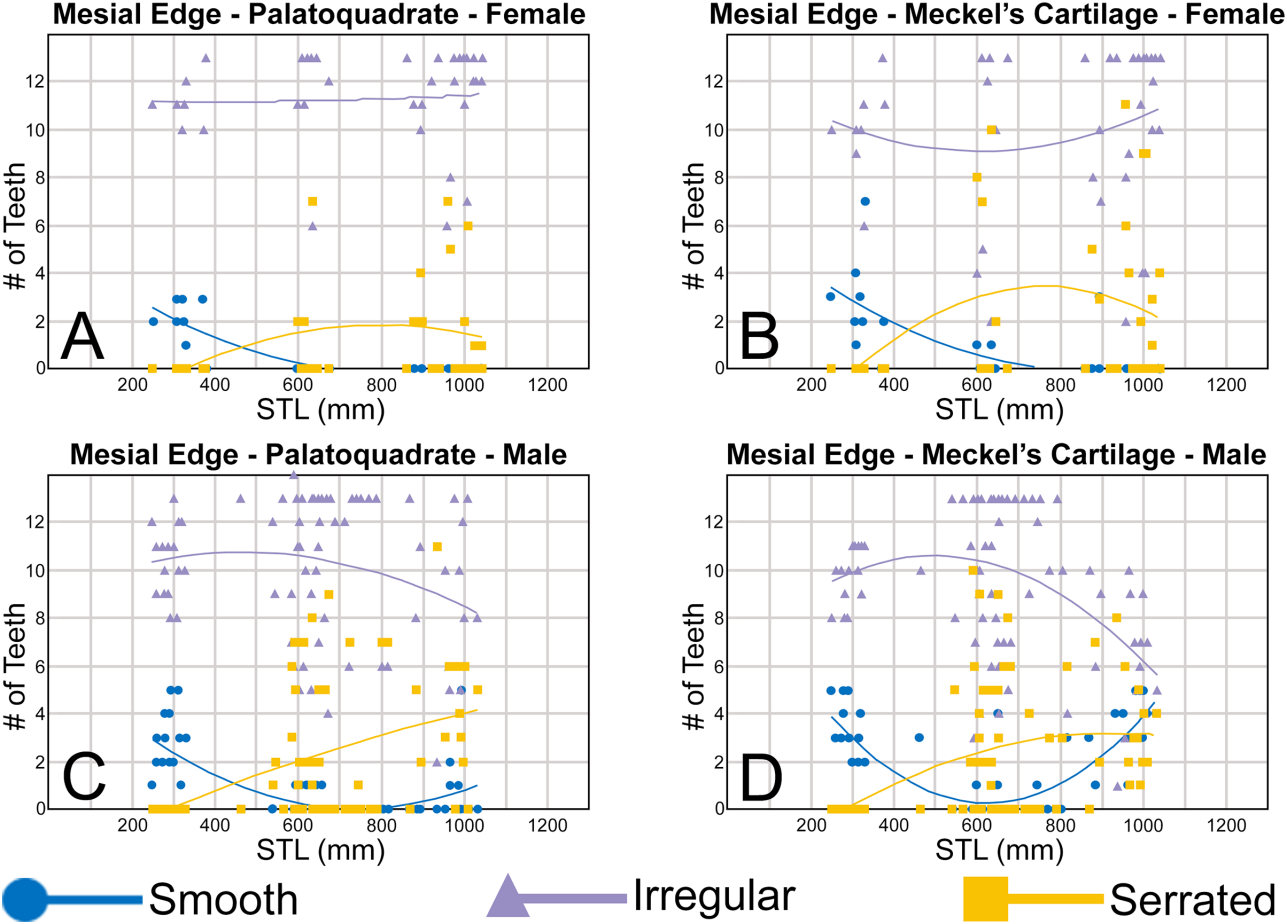
(A–D) Plots and polynomial trends of the nature of the mesial cutting edge on female and male *Rhizoprionodon terraenovae* teeth through ontogeny on the Meckel’s cartilage and palatoquadrate. Tooth numbers represent the number of teeth with a particular characteristic (*i.e*., smooth, irregular, or serrated mesial edge) that occur within the functional row (left or right) of the Meckel’s cartilage or palatoquadrate. STL, stretched total length.

Our data shows that the ontogenetic development of the mesial and distal cutting edges is similar to that of the distal heel and the cutting edges generally transition from smooth, to irregular, to serrated (Fig. 12) as the shark matures. The four smallest pups in our sample (STL 130 to 190 mm) show that all teeth initially have smooth mesial and distal cutting edges (Fig. 8). In females, the number of teeth with smooth mesial and distal cutting edges is highest in pups, but steadily declines as the shark matures, with very few smooth-edged teeth being present in specimens exceeding STL 700 mm (Figs. 13A, 13B, 14A, 14B). A similar trend can be seen on the palatoquadrate of male specimens (Figs. 13C, 14C), but on the Meckel’s cartilage the number of teeth with smooth mesial and distal cutting edges increases, with specimens in the STL 900 to 1,040 mm size classes often having higher numbers of such teeth compared to those in the STL 250 to 300 mm size class (Figs. 13D, 14D).

Teeth with irregular mesial and distal cutting edges are the most numerous in both the Meckel’s cartilage and palatoquadrate in both sexes and across ontogeny. As observed on the distal heel, ontogenetic/morphological development of the mesial and distal cutting edges begins *in utero*, with irregular cutting edges (Fig. 13) occurring on a majority of teeth in specimens as small as STL 250 mm in both female and male specimens (Figs. 13, 14). Overall, our trends show that the ontogenetic development of the mesial and distal cutting edges on female specimens appears to be relatively constant across ontogeny in both jaws. In females, the number of teeth with irregular mesial and distal cutting edges remains relatively consistent across ontogeny, with the decrease in the number of teeth with smooth edges in specimens between STL 250 to 600 mm corresponding with higher occurrence of teeth with serrated mesial and distal edges within the same size range (Figs. 13A, 13B, 14A, 14B). Very few teeth with smooth cutting edges are present in specimens exceeding STL 700 mm, and the slight decline in the number of serrated teeth in specimens exceeding STL 800 mm corresponds with a slight increase in the number of teeth with irregular cutting edges within the same range.

The trends on male teeth are more complicated, as the number of teeth with smooth, irregular, and serrated cutting edges appears to differ more drastically between the Meckel’s cartilage and palatoquadrate and between the two cutting edges. Variability can be seen in trends plotted for the distal and mesial edges (Figs. 13, 14) in males, indicating that cutting edges develop differently. For example, on the palatoquadrate the number of teeth with irregular distal cutting edges is highest in the STL 250 mm size class and lowest between STL 600 to 700 mm, before increasing in number in specimens exceeding STL 700 mm (Fig. 13C). In contrast, the number of teeth with irregular mesial edges in the palatoquadrate increases slightly in the STL 400 to 500 mm size class before incrementally decreasing in number in specimens exceeding STL 500 mm (Fig. 14C). On the Meckel’s cartilage in males, the number of teeth with irregular mesial and distal cutting edges decreases through ontogeny (Figs. 13D, 14D). Although this decline is relatively constant on the distal edge (Fig. 13D), the mesial edge exhibits an increase in the occurrence of irregular cutting edges on specimens in the STL 500 mm size class, before steadily declining in number in larger specimens (Fig. 14D).

On the distal edge of the teeth, the trends plotted for both irregular and smooth cutting edges across both male jaws appear to be strongly influenced by the occurrence of those with serrated distal edges (Figs. 13C, 13D). When the number of teeth with serrated distal edges increases, the number of teeth with irregular and smooth distal edges decreases, indicating that serrated edges begin to replace those of the other two. The opposite also holds true, as declines in the number of teeth with a serrated distal edge results in increases in the number of teeth with irregular or smooth distal edges. One notable difference between the two jaws is the larger number of smooth distal edges on teeth in the largest size classes (STL 800 to 1,050 mm) on the Meckel’s cartilage. In specimens exceeding STL 900 mm, teeth with smooth and irregular distal edges are nearly equal in number and far exceed the number of teeth with serrated distal edges (Fig. 13D). In contrast, on the palatoquadrate the number of teeth with irregular distal edges are the most prevalent in males of all size classes, but in specimens exceeding STL 900 mm they far outnumber those with serrated or smooth distal edges, of which the latter two are almost equal in number (Fig. 13C).

On the mesial edge of the teeth, the trends plotted for the number of irregular and smooth edges appear to have an inverse relationship (Figs. 14C, 14D). On both the Meckel’s cartilage and palatoquadrate, as the number of teeth with an irregular mesial edge increases, those with a smooth distal edge declines, and vice versa. In contrast, the number of teeth with serrated mesial edges gradually increase in number across ontogeny on both the Meckel’s cartilage and palatoquadrate, although the number of teeth with serrated mesial edges is greater in the palatoquadrate than it is in the Meckel’s cartilage. Notably in the largest size classes (>STL 900 mm), the teeth in the Meckel’s cartilage have a higher percentage with a smooth mesial edge than serrated, and there is a substantial decline in the number of teeth with irregular mesial cutting edges. This suggests that smooth cutting edges are replacing those with irregular/serrated edges in the males in this size class.

The disparity we observed between the trends plotted for the mesial and distal cutting edges and between the sexes appears to be related to the acquisition and/or loss of teeth with serrated edges through ontogeny. In our sample, no serrations were observed on the mesial and distal cutting edges of any *in utero* pup specimens (STL 190 to 330 mm), and serrations were absent from teeth on the jaws of neonate male (MSC 46739, STL 462 mm) and female (MSC 46740, STL 371 mm; MSC 46741, STL 378 mm) specimens we examined. The first mesial or distal serrations we observed occurred on a female of STL 600 mm (MSC 42649) and a male of STL 540 mm (MSC 42652), indicating that mesial/distal serrations develop after birth and not *in utero*. Our dataset also shows that mesial/distal serrations develop on male teeth before females, and serrations develop on the mesial and distal cutting edges before they form on the distal heel (the earliest observed occurrence of distal heel serrations is on a female specimen of STL 879 mm and a male of STL 595 mm). Finally, our data shows that in the largest size classes (>STL 900 mm) females have a larger number of serrated teeth than males, and in males a large number of teeth with serrated cutting edges on the Meckel’s cartilage are replaced with those with smooth cutting edges.

### Gynandric heterodonty

Gynandric heterodonty, or dental sexual dimorphism, refers to morphological differences between male and female dentitions of the same species, and this phenomenon may be year-round or seasonal. Year-round gynandric heterodonty can be observed between mature male and female dentitions throughout the year, as is the case in the Smallspotted Catshark, *Scyliorhinus canicula* (Linnaeus, 1758) (see Ellis & Shackley, 1995; Filiz & Taskavak, 2006). Seasonal gynandric heterodonty refers to a specific period of time during the year, generally breeding season, when the male and female dentitions differ, otherwise the dentitions are generally indistinguishable during the remainder of the year. Seasonal gynandric heterodonty is not well documented in galeomorph sharks but has been reported in batoids like the Atlantic Stingray, *Hypanus sabinus* (Lesueur, 1824) (see Kajiura & Tricas, 1996).

Of the seven extant *Rhizoprionodon* species, Springer (1964) and Compagno (1984, 1988) noted the development of gynandric heterodonty in mature specimens of *R. lalandii* and *R. oligolinx*, and its absence in mature specimens of *R. acutus*, *R. longurio*, *R. porosus*, and *R. terraenovae*. Springer (1964) considered the presence of gynandric heterodonty to be one of several morphological criteria for his proposed subgenus *R*. (*Protozygaena*), and those lacking this phenomenon were assigned to the subgenus *R*. (*Rhizoprionodon*). Both Springer (1964) and Compagno (1984) lacked sufficient data to evaluate the presence of gynandric heterodonty in *R. taylori*, but Springer (1964) included this taxon within *R*. (*Protozygaena*) based on other criteria. Although Compagno (1988) rejected Springer’s (1964) use of these subgenera, the latter’s illustrated *Rhizoprionodon* dentitions support his observations regarding the presence or absence of gynandric heterodonty within the various species.

**Figure 15:**
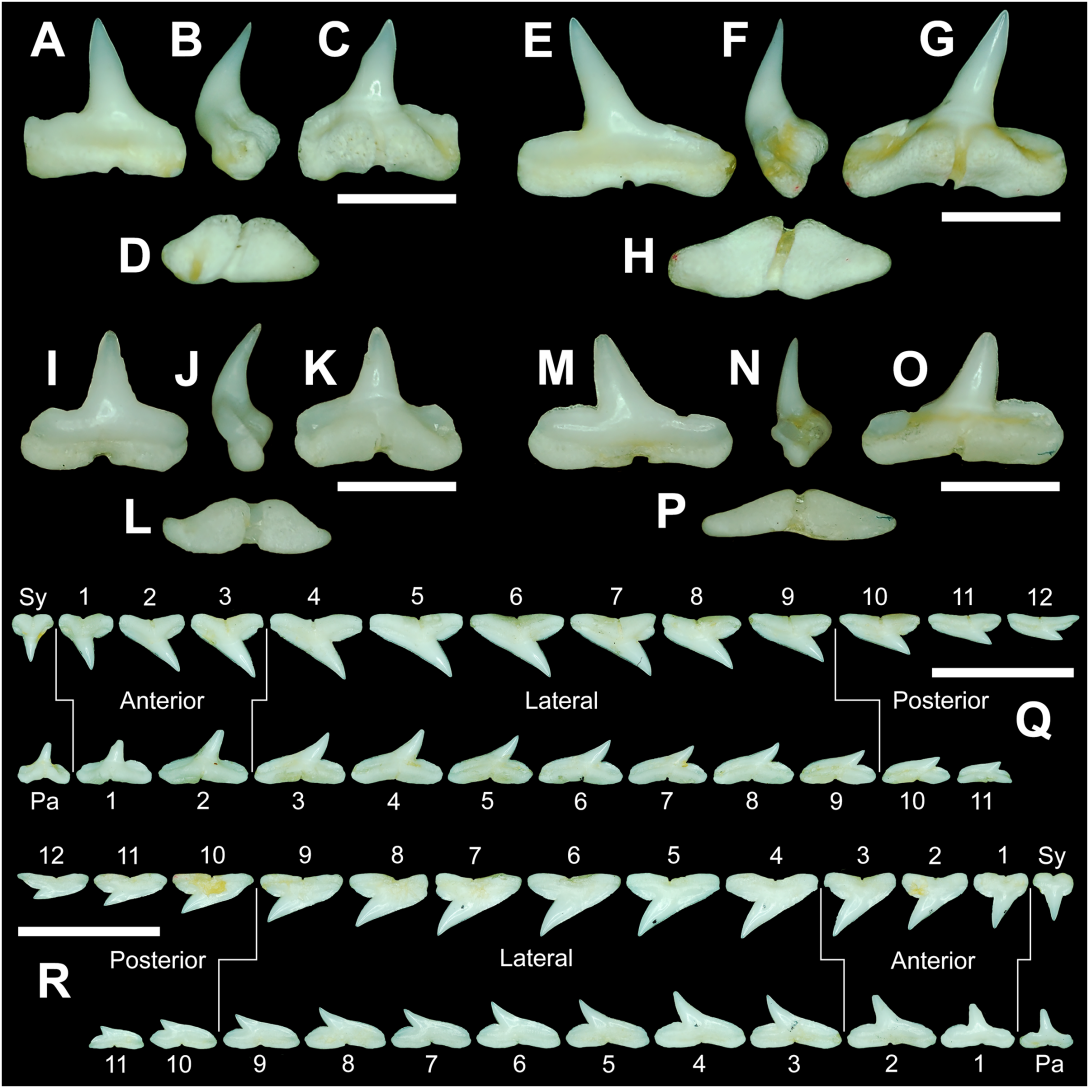
Dentition of a mature male *Rhizoprionodon terraenovae* and a comparison of gynandric (male) and non-gynandric (female) tooth morphologies. (A–H) MSC 44457, mature male, STL 990 mm. (A–D) Lower right gynandric parasymphyseal tooth in (A) labial, (B) mesial, (C) lingual, and (D) basal views. (E–H) lower right gynandric 1^st^ anterior tooth in (E) labial, (F) mesial, (G) lingual, and (H) basal views. (I–P) MSC 44461.1, mature female, STL 991 mm. (I–L) Lower right non-gynandric parasymphyseal tooth in (I) labial, (J) mesial, (K) lingual, and (L) basal views. (M–P) Lower right non-gynandric 1^st^ anterior tooth in (M) labial, (N) mesial, (O) lingual, and (P) basal views. (Q and R) MSC 42656, dentition of a mature gynandric male, STL 895 mm, in (Q) lingual view and (R) labial view. Numbers refer to specific tooth files that are numbered consecutively from the symphysis to the commissure. Pa, parasymphyseal tooth; Sy, symphyseal tooth. Scale bars for A–P = 3 mm. Scale bars for Q–R = 1 cm.

Our sample revealed marked gynandric heterodonty within all but one of the jaws of the mature male specimens that exceeded STL 815 mm, contradicting the conclusions of Springer (1964) and Compagno (1984, 1988) that gynandric heterodonty was absent in *R. terraenovae*. Our sample included 16 specimens that exceeded STL 815 mm, 10 of which were caught in the months of April and May and six in September and October (see Appendix 1.1). With the exception of specimen MSC 46703, the remaining 15 specimens exhibit conspicuous morphological differences between the lower parasymphyseal files and the first and second anterior files (*i.e*., lower positions 1 and 2) when compared to female and non-mature male specimens (Fig. 15). Within these tooth files, the individual teeth lack mesial and distal serrations and/or irregular cutting edges, and the labial and lingual faces of the crown are equally convex to the extent that the main cusp is circular in cross section (Figs. 15A–15H). Furthermore, gynandric heterodonty is expressed through teeth with a narrower, more needle-like main cusp that is more upturned in the mouth. Additionally, the distal heel is smooth and generally has a rounded cutting edge, and the teeth are labiolingually thicker than their non-gynandric counterparts (Figs. 15A–15P). Furthermore, some teeth completely lack cutting edges on the main cusp (Figs. 15A–15H).

In 10 of the 15 jaws that exhibit gynandric heterodonty, all the replacement teeth within the aforementioned files have the same morphology as those in the corresponding functional rows (*i.e*., all have the gynandric morphology). On the five remaining jaws (MSC 42671, MSC 46739, MSC 46735, MSC 46736, and MSC46737), gynandric teeth occur in the functional rows, but the teeth in all of the corresponding replacement rows display the non-gynandric morphology. On these jaws the replacement teeth have reverted to their original morphology, which includes having a wider and more triangular main cusp with distinct cutting edges and a more mesiodistally compressed crown. Although the mesial and distal edges of the replacement teeth in these files are not serrated, the edges appear irregular, and the rounded distal heel often has coarse serrations. Because these five specimens exhibit a combination of gynandric and non-gynandric tooth morphologies within the lower parasymphyseal and anterior tooth files, it demonstrates that male dentitions on specimens greater than STL 815 mm undergo seasonal changes, rather than maintaining gynandric teeth year-around. These specimens also show that non-gynandric and gynandric teeth are two distinct tooth morphologies, and one does not grade morphologically into the other. Instead, the difference between the morphology of the gynandric and non-gynandric teeth is drastic and one simply replaces the other, with no intermediate morphologies developing. Of the 16 male specimens in our sample that exceeded STL 815 mm, only one MSC 46703 (STL 964 mm) did not exhibit any gynandric teeth. However, due to the late calendar date when this specimen was caught (September 6), it is likely that all the seasonal gynandric teeth had already been replaced by non-gynandric ones.

The development of gynandric teeth in the parasymphyseal and anterior positions in the Meckel’s cartilage of mature males exceeding STL 815 mm is indicated by several trends in our polynomial data plots. Most notably, the development of gynandric teeth in mature males is reflected by the large increase in the number of teeth with smooth mesial and distal cutting edges (Figs. 13D, 14D) when compared to those in the palatoquadrate (Figs. 13C, 14C) or female specimens within the same size class (Figs. 13B, 14B). Coinciding with this increase is the relative decrease in the number of teeth with serrated or irregular cutting edges within the Meckel’s cartilage (Figs. 13D, 14D).

In addition to documenting the gynandric tooth morphologies described above, we also tested for the presence of more subtle signs of gynandric heterodonty in this species. To do so, several male and female dentitions were compared under magnification for morphological differences, and to account for morphological differences resulting from ontogeny (see above), a total of 10 female and 10 male jaws (*n* = 20) with nearly identical STLs were compared across various size classes (see Material and Methods). These ten size classes included female and male pairs with STLs of approximately 600 mm (MSC 42649 and MSC 42662), 610 mm (MSC 42645 and MSC 42663), 615 mm (MSC 43583 and MSC 44474), 635 mm (MSC 43585 and MSC 42657), 675 mm (MSC 42638 and MSC 42651), 895 mm (MSC 42685.1 and MSC 42656), 935/940 mm (MSC 44482.1 and MSC 44454), 980 mm (MSC 44481.1 and MSC 44480), 1000/1001 mm (MSC 42670 and MSC 42671), and 1030/1033 mm (MSC 42676 and MSC 44456).

**Figure 16:**
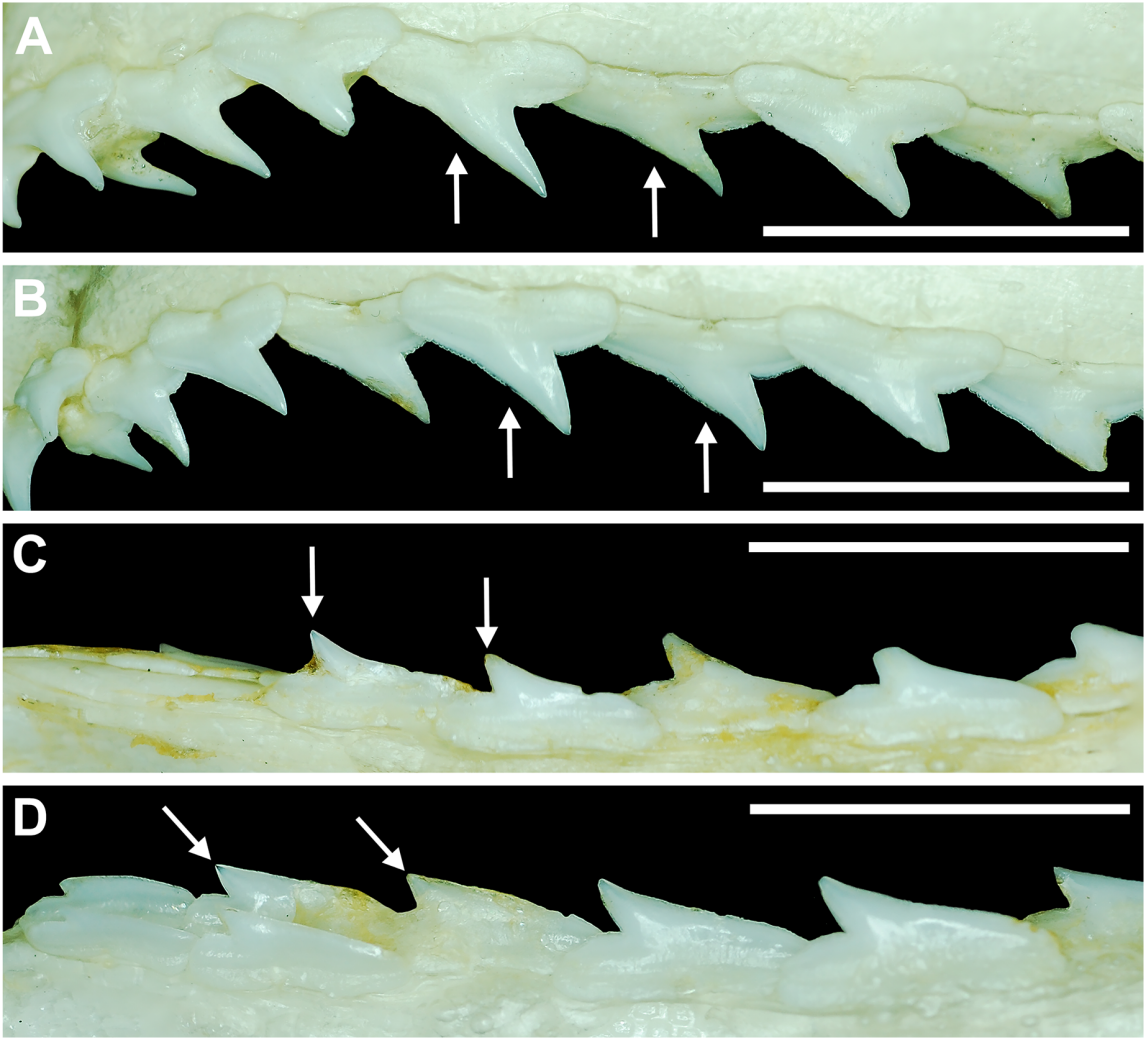
Gynandric heterodonty in mature male *vs* female *Rhizoprionodon terraenovae*. (A) MSC 44454, mature male, STL 935 mm, labial view of left upper dentition showing narrow main cusp on teeth (denoted by arrows). (B) MSC 44482.1, mature female, STL 940 mm, labial view of left upper dentition showing wide main cusp on teeth (denoted by arrows). (C) MSC 44480, mature male, STL 980 mm, labial view of left lower dentition showing the upturned apex on posterior teeth (denoted by arrows). (D) MSC 44481.1, mature female, STL 980 mm, labial view of left lower dentition showing the distally directed apex on posterior teeth (denoted by arrows). Scale bars = 1 cm.

As stated earlier, both Springer (1964) and Compagno (1984, 1988) noted the absence of gynandric heterodonty in *R. terraenovae*. Based on our observations of the jaw sets in our sample, this determination is only partially accurate, as no morphological differences could be observed between the female and male jaw sets for specimens between STL 600–815 mm. However, of the specimens exceeding STL 815 mm, slight morphological differences could be observed between the corresponding male and female jaw sets, in addition to the gynandric heterodonty documented above. For example, on the male specimen MSC 44454 (STL = 935 mm), the upper teeth have a mesiodistally narrower main cusp (Fig. 16A) with mesial and distal cutting edges that are more sinuous, and they have a more convex labial face compared to those in the corresponding files on the female specimen MSC 44482.1 (STL = 940 mm; Fig. 16B). In addition, specimen MSC 44454 (male) exhibits teeth with a more convex labial crown face than does MSC 44482.1 (female), a characteristic that is much more apparent on the files located closer to the jaw symphysis. A similar condition was observed on the teeth of the male specimen MSC 44480 (STL = 980 mm), which also has lower lateral teeth with a noticeably more upturned apex and narrower main cusp (Fig. 16C) compared to those in the corresponding files of the female specimen MSC 44481.1 (STL = 980 mm; Fig. 16D). On the two specimens with an STL of approximately 1,000 mm, the female jaw (MSC 42670, STL = 1,001 mm) has upper teeth with a noticeably wider main cusp than the corresponding teeth on the male specimen (MSC 42671, STL = 1,000 mm). In contrast, the upper teeth on the male specimen (MSC 42671) have a narrower main cusp, slightly more upturned apex, and a slightly more sinuous mesial and distal cutting edges. Moreover, the lower posterior-most teeth on this male specimen (MSC 42671) have a narrower and more upturned main cusp than teeth in the corresponding files on the female specimen (MSC 42670). Of the female and male specimens with an STL between 1,030 and 1,033 mm, the male jaw (MSC 44456, STL = 1,033 mm) has upper teeth with a narrower main cusp and more sinuous mesial and distal cutting edges compared to the files in the corresponding files of the female specimen (MSC 42676, STL = 1,030 mm). Lastly, the posterior-most teeth on the male specimen have a narrower main cusp and more upturned apex, and the labial face of the crown on the anterior files is more convex than those on the female specimen.

To determine whether these subtle characteristics are attributable to seasonal or year-round gynandric heterodonty, specimens MSC 44482.1 (mature female, STL 940 mm) and MSC 44454 (mature gynandric male, STL 935 mm) were compared to MSC 46703 (STL 964 mm), the only mature male in our sample that did not exhibit gynandric teeth. This comparison showed that the dentition of the non-gynandric male (MSC 46703) was identical to that of the mature female (MSC 44482.1) and that it has the same morphological tooth differences as the female specimen when compared to the mature gynandric male (MSC 44454). This indicates that the morphological characteristics noted above (Fig. 16) are also seasonal but less conspicuous compared to the lower gynandric tooth morphologies (Figs. 15A–15P). In fact, it must be stressed that these morphological differences are slight and not readily apparent on isolated teeth. Rather, jaw sets of mature males and females of the same STL need to be placed side-by-side, under magnification, to observe many of these differences. The subtlety of these tooth variations is likely why seasonal gynandric heterodonty has not been previously reported for *R. terraenovae*.

#### Ontogenetic tooth stages

It should be emphasized that the ontogenetic/morphological changes documented in this study are based on direct observations and trends we observed in our dataset (see Figs. 11–14, Appendices 1.19–1.34). Amongst the jaws in our dataset, we found that there is a significant amount of variability regarding the ontogenetic development of the individual teeth. For example, the ontogenetic development of teeth occurs at different rates within an individual tooth row, as well as between the palatoquadrate and Meckel’s cartilage, among growth stages, and between sexes (*i.e*., gynandric heterodonty). Nevertheless, the patterns of morphological change observed in our sample indicate that the isolated teeth and dentitions generally progress through five ontogenetic stages, which we identify as:

*Stage 1*: This stage is denoted by the development of the individual teeth, files, and tooth rows. The initial development of the dentition occurs *in utero*, as demonstrated by specimens between STL 100 and 200 mm. Less than five tooth rows (generally two) are present on specimens in this size class, and the distal heel is still forming on most teeth (Fig. 8). Regarding individual teeth, the main cusp forms before the root, and the initial formation of the root occurs before the formation of the distal heel. The mesial and distal cutting edges of the main cusp and distal heel are smooth on all teeth. By STL 250 mm, all five tooth rows are formed in the Meckel’s cartilage and palatoquadrate, and nearly all the teeth in the functional row are fully developed.

*Stage 2*: This stage occurs *in utero* and is marked by the transition from a rounded to triangular distal heel (Fig. 9) on the teeth, and irregular cutting edges develop on the distal heel and mesial/distal cutting edges of some teeth (Fig. 10). The distal heel is still developing on some teeth of specimens between STL 200 to 310 mm, but most of the distal heels are rounded (although a few are triangular). Across the tooth row, the cutting edge on the distal heel varies from smooth to irregular. As the specimens increase in STL, a greater number of teeth develop a triangular distal heel with an irregular cutting edge, and a larger number of teeth exhibit irregular mesial and distal cutting edges.

*Stage 3*: Individuals are neonatal and older during this stage, which is marked by the first occurrence of distal heel bifurcation (Fig. 9) and serrations on the mesial and distal cutting edges (Fig. 10). During this stage, some teeth of male specimens also exhibit the first signs of serrations on the distal heel. However, the cutting edges on the distal heel and mesial and distal edges on teeth of female specimens are generally irregular.

*Stage 4*: This stage is marked by the development of coarse serrations on both the mesial and distal cutting edges (of most teeth), and the first occurrence of serrations on the distal heel of female teeth. During this stage, the distal heel on a majority of female teeth transitions from triangular or bifurcated to rounded. In male dentitions, the distal heel shape is more varied and can be triangular, bifurcated, or rounded.

*Stage 5*: During this stage, the mesial and distal cutting edges and the distal heel on most teeth exhibit irregular and/or serrated edges (Figs. 9, 10). The distal heel on most teeth changes from triangular or bifurcated to rounded (Fig. 9). Gynandric heterodonty can be observed in male jaws and is particularly evident in the lower anterior and parasymphyseal files.

## Discussion

### Ontogenetic development of serrated teeth

The dental morphology of chondrichthyans varies greatly across the numerous fossil and extant taxa (see Naylor & Marcus, 1994; Cappetta, 2012; Ebert & Dando, 2021). Several previous studies attempted to group the various chondrichthyan dentitions into certain dental types that are largely reflective of function and adaptations to specific feeding habits (see Moss, 1972; Cappetta, 1986; Cappetta, 2012). Cappetta (2012) identified nine different chondrichthyan dental types, including clutching, tearing, cutting (which includes two sub-types, *senso stricto* cutting, and cutting-clutching), crushing, grinding, clutching-grinding, cutting-grinding, and crushing-grinding.

The *R. terraenovae* dentition is classified as a *sensu stricto* cutting subtype (*sensu* Cappetta, 2012), typified by closely spaced teeth with sharp cutting edges that essentially form a single continuous, sharp cutting surface along the functional rows. Monognathic and dignathic heterodonty is evident, with the main cusp on the teeth varying in width between the upper and lower jaws (dignathic) and generally becoming more distally inclined the closer a tooth is located to the commissure (monognathic). Teeth within this dental subtype can have smooth or serrated cutting edges, but the cutting efficiency is greatly increased by the development of serrations (Cappetta, 2012; Moyer & Bemis, 2017).

Our analysis of *R. terraenovae* jaws showed that the initial cutting edges on the teeth are smooth, but they develop irregular and eventually serrated cutting edges through ontogeny (Figs. 11–14, Appendices 1.23–1.34). Our data revealed that irregular cutting edges develop *in utero* (on pups as small as STL 250 mm) on the mesial and distal cutting edges of the main cusp and on the distal heel of the teeth. Conversely, serrated teeth do not develop *in utero* and instead develop shortly after birth. The same can be said for the development of a bifurcated distal heel on the teeth (which is a precursor to the development of serrations on the heel) because this feature does not occur in any of the *in utero* pup or neonate jaws in our sample. Our data also points to a possible relationship between the development of serrated distal heels and sexual maturity in female *R. terraenovae* because all but one (MSC 44471.1, STL 862 mm) of the pup-bearing individuals in our sample exhibited this characteristic (19 of 20, 95%). Conversely, serrated distal heels on teeth were observed on male specimens across various growth stages, including non-neonate immature, transitional, and mature individuals.

Our data demonstrates that serrations first appear on the mesial and distal cutting edges on the teeth of both male and female specimens between STL 500–600 mm. Additionally, serrations develop on the mesial and distal cutting edges before they occur on the distal heel, and these latter serrations occur in male specimens (first occurrence STL 605 mm) before they do in females (first occurrence STL 879 mm). Our interpretation of this data is that the development of irregular and serrated cutting edges on *R. terraenovae* teeth is ontogenetic (as alluded to by Springer (1964) and Compagno (1984, 1988)) and reflects ontogenetic dietary shifts within the species.

Various studies have shown that the diets of *R. terraenovae* populations in the northern Gulf of Mexico differ slightly by region as well as ontogenetic stage (Bethea, Buckel & Carlson, 2004; Bethea et al., 2006; Drymon, Powers & Carmichael, 2012; Harrington et al., 2016; Plumlee & Wells, 2016; Seubert et al., 2019). In the northern Gulf of Mexico, young-of-the-year individuals primarily feed on shrimp, crustaceans, and cephalopods, but they become increasingly reliant on fishes (mainly sciaends and clupeids) through ontogeny. We compared our data to the datasets utilized by Drymon, Powers & Carmichael (2012) and Harrington et al. (2016) to determine if there was a relationship between the reported ontogenetic dietary shifts for *R. terraenovae* and the ontogenetic tooth stages we observed in our sample. Drymon, Powers & Carmichael (2012) and Harrington et al. (2016) used both nitrogen and carbon stable isotope ratios and stomach content analyses to examine the diets of 454 male and female *R. terraenovae* specimens across various growth stages caught from the northern Gulf of Mexico between Alabama and Texas, USA.

**Figure 17:**
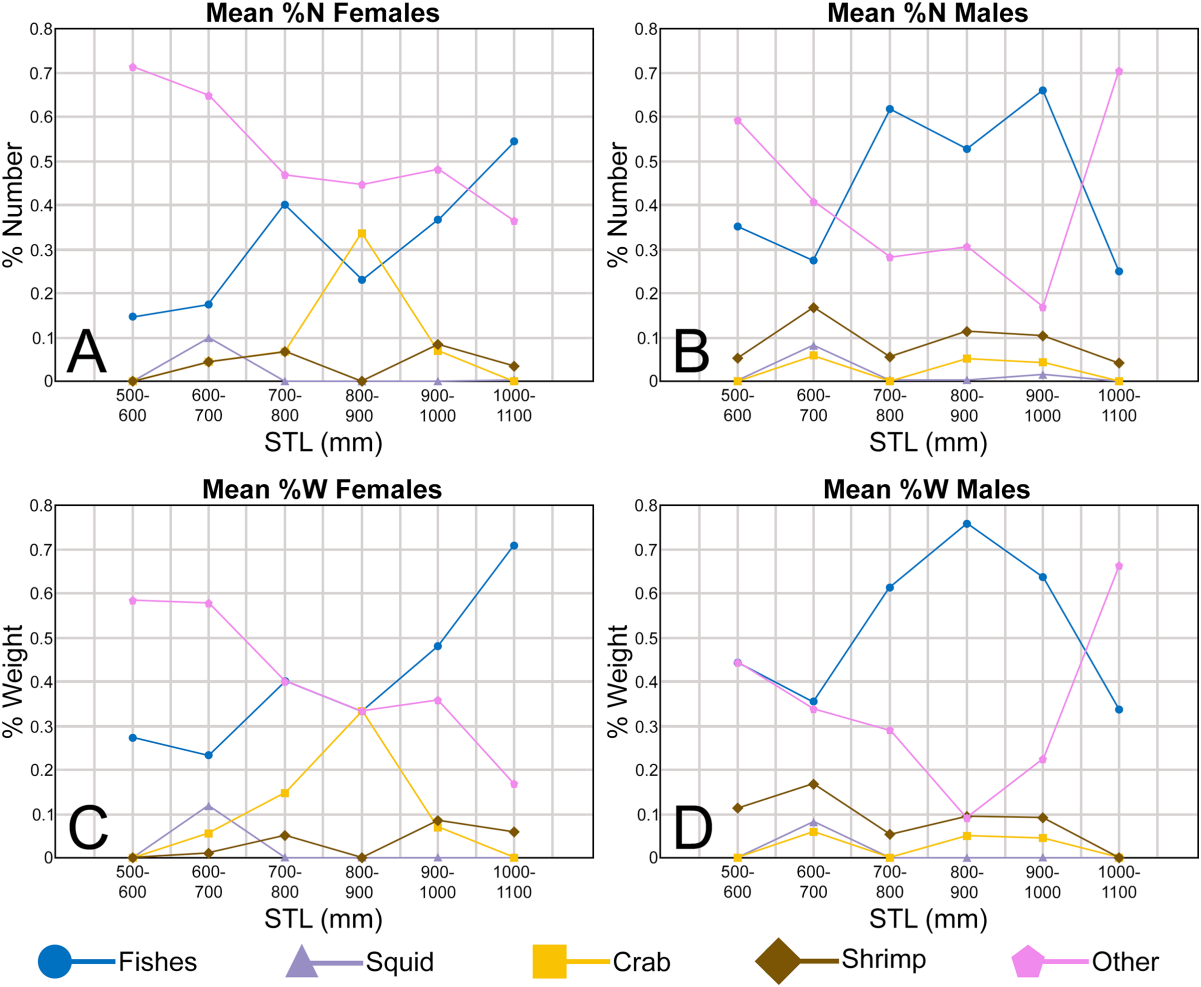
(A–D) Mean number (%N) and weight (%W) of prey items through ontogeny in the diets of male and female *Rhizoprionodon terraenovae* from the northern Gulf of Mexico, USA. STL, stretched total length. Data derived from Drymon, Powers & Carmichael (2012) and Harrington et al. (2016).

The data provided by Drymon, Powers & Carmichael (2012) and Harrington et al. (2016) showed a relationship between the percentage of fishes in the stomach and the length of a shark, with the mean weight (%W) and number (%N) of fishes generally increasing as the shark matured (Fig. 17, Appendices 1.35–1.36). Their data also showed that males consumed a higher percentage of fishes at a smaller size class than did females, with males in the STL 500–600 mm size class having 0.44 %W and 0.35 %N of fishes in their stomachs (Figs. 17B–17D, Appendices 1.35–1.36) compared to 0.27 %W and 0.14 %N in females of the same size class (Figs. 17A–17C, Appendices 1.35–1.36). In terms of %W, males had the highest percentage of fish consumption (76%) in the STL 800–900 mm size class, whereas female specimens peaked in the STL 1,000–1,100 mm size class (71%).

When this data is compared to our dataset, there appears to be a strong relationship between the ontogenetic development of irregular and serrated cutting edges on the teeth and the increase in fish consumption as the shark matures. Although male and female *R. terraenovae* individuals consume fishes throughout their lives, there is an initial dietary preference towards invertebrate prey before they become increasingly reliant on mostly larger teleost fish as they mature. Drymon, Powers & Carmichael (2012) and Harrington et al. (2016) observed that the diet of young-of-the-year *R. terraenovae* individuals (STL 330–590 mm) is comprised of relatively low percentages of small fishes but high percentages of invertebrates. This supports our observations of the development of irregular cutting edges on most of the teeth in the jaws of pups between STL 250–330 mm and neonate specimens between STL 371 and 462 mm (Figs. 12–14), as irregular cutting edges are likely more efficient at cutting flesh than are smooth ones (*sensu* Moyer & Bemis, 2017), and the *in utero* development of irregular cutting edges likely enables *R. terraenovae* specimens to consume small fishes shortly after birth. This also indicates that teeth with smooth or irregular cutting edges are sufficient for the consumption of softer bodied invertebrate prey and the occasional small fish.

Our data indicates that males develop serrated teeth before females, with the first mesial and distal serrations occurring on a tooth in a male specimen at STL 540 mm and in a female specimen at STL 600 mm (Appendices 1.23–1.34). In addition, the first serrated distal heel was observed on a male specimen at STL 605 mm, and on a female specimen at STL 879 mm (Appendices 1.23–1.34). This agrees with the datasets of Drymon, Powers & Carmichael (2012) and Harrington et al. (2016), which indicated that male *R. terraenovae* consume a larger percentage of fishes at the STL 500–600 mm size class (35.2% of the total diet) than do females of the same size (14.3%) and, overall, males generally consume a larger percentage of fishes than females of comparable size (Fig. 17, Appendices 1.35–1.36). This corresponds to the trends observable in our dataset, where males develop serrated teeth at shorter STLs than females, and immature and mature males generally have a larger number of fully serrated teeth within their dentitions than immature or mature females (Figs. 12–14). That the development of serrated cutting edges is concurrent with the increased consumption of fishes is not surprising, as serrated cutting edges are highly effective at cutting flesh (Moyer & Bemis, 2017).

Springer (1967) and Drymon et al. (2020) demonstrated that *R. terraenovae* populations in the northern Gulf of Mexico were highly segregated by sex, with males being abundant inshore during the spring and autumn, whereas females inhabited offshore areas during these periods. This sexual segregation may account for the more fish-heavy diets of males and for the development of serrated teeth in males before females, as males spend more time in the fish-rich inshore waters than females. Nevertheless, it is evident that the ontogenetic development of serrated teeth provides a distinct functional advantage for the concurrent increasing consumption of larger fishes.

The dataset of Drymon, Powers & Carmichael (2012) and Harrington et al. (2016) also showed a decrease in both the %W and %N of fishes in the largest size class of males within their sample (STL 1,000–1,100 mm; Figs. 17B, 17D), coupled with an increase in the consumption of other prey items. In contrast, female specimens of the same size class continued to show an increased reliance on fishes as part of their diets (Figs. 17A, 17C). This disparity may be related to seasonal gynandric heterodonty within the lower anterior files of mature male specimens (<STL 815 mm). The *R. terraenovae* dentition is a cutting adaptive dental type (*sensu* Cappetta, 2012), and it is possible that the unique male gynandric tooth morphology, specifically the narrowing of the main cusp and loss of cutting edges and serrations (Fig. 15), slightly alters the feeding mechanics of the shark, and therefore affects the dietary behavior of mature male individuals. When gynandric teeth develop, these tooth files transition from a *senso stricto* cutting subtype to a clutching dental type (*sensu* Cappetta, 2012), likely rendering them less efficient than serrated teeth when consuming large fishes. Assuming this interpretation is correct, the phenomenon could account for the slight alteration in the feeding behavior/diet of mature male sharks during the breeding season (see below), when they appear to be less reliant on larger fishes but more so on softer-bodied invertebrates and smaller fishes.

### Gynandric heterodonty

Fifteen of the 16 largest male specimens in our sample exhibit gynandric heterodonty, which is expressed as the development of unique needle-like teeth in the lower parasymphyseal and anterior tooth positions, and subtle morphological changes in other tooth positions. Ten of the 16 specimens were caught between April and May, whereas the remaining six were caught in September and October. Of the latter six jaw sets, all but one (MSC 46703) exhibits one-to-two rows of gynandric teeth in the functional position that are succeeded by three-to-four rows of non-gynandric replacement teeth. Because these specimens clearly illustrate that gynandric teeth in *R. terraenovae* are replaced, it indicates that these teeth develop seasonally, as opposed to being present year-round.

Of the 15 male specimens in our sample that exhibit seasonal gynandric teeth, all exceeded STL 815 mm, and 14 of 15 were fully mature based on extent of the calcification of their myxopterygia (see Materials and Methods). The only exception was specimen MSC 43590, which was a large transitional specimen measuring STL 885 mm (see Appendix 1.1). Overall, of our sample of male specimens measuring less than STL 815 mm, all were classified as transitional, immature, or were *in utero* pups. Without exception, seasonal gynandric teeth were absent in all specimens smaller than STL 815 mm as well as in all female specimens, regardless of size class. This indicates that the occurrence of gynandric teeth in *R. terraenovae* males is ontogenetic as well as seasonal. In addition, 14 of 15 specimens exhibiting gynandric teeth were fully mature, indicating that this phenomenon only occurs in mature males or large transitional specimens exceeding STL 815 mm. Specimen MSC 46703 is of interest because it is the only male specimen in our sample that exceeds STL 815 mm that does not have gynandric teeth. The lack of gynandric teeth in this specimen, which was caught on September 6, 2022, again indicates the seasonality of these teeth because it is likely that all had already been replaced by non-gynandric teeth subsequent to the end of the breeding season.

The unique morphology of the gynandric teeth in *R. terraenovae*, their seasonal development (~April to September), and almost exclusive occurrence in mature males strongly suggests their morphology is associated with reproduction as opposed to diet. Hoffmayer et al. (2013) indicated a variable, yet annual, reproductive cycle for *R. terraenovae* populations in the northern Gulf of Mexico, with the optimal water temperatures in the region likely allowing the species to breed throughout much of the year (March to October). This protracted breeding season contrasts with previous studies of *R. terraenovae* in the southern USA that indicated a much shorter, May to July, breeding season (*i.e*., Parsons, 1983; Loefer & Sedberry, 2003). We note here that Hoffmayer et al. (2013)’s study was based on a much larger sample size (*n* = 1,306) than that of Parsons (1983) (*n* = 66), and the latter study included specimens that were caught exclusively off the Alabama coast. In contrast, Hoffmayer et al. (2013)’s samples were obtained from a larger geographic area within the Gulf of Mexico, between Florida and Texas. Loefer & Sedberry (2003) had a sample size (*n* = 1,093) comparable to that of Hoffmayer et al. (2013), but their specimens were caught off the Atlantic Coast between northern Florida and Virginia, and their conclusions regarding a shortened breeding season are likely indicative of regional differences in reproductive cycles. Our observation of gynandric teeth within specimens caught between April and October appears to corroborate Hoffmayer et al. (2013)’s conclusions of reproductive plasticity within *R. terraenovae* populations in the northern Gulf of Mexico, with the species being capable of breeding any time in this region between March and October.

Sexual maturity in male sharks requires the complete development of the myxopterygia and rhipidion, and the myxopterygia must obtain a specific level of calcification so they can be rotated 180° in order to be inserted into the cloaca of the female to deliver sperm. In a study on the male reproductive biology of *R. terraenovae* in the northern Gulf of Mexico, Hoffmayer et al. (2010) correlated increases in reproductive hormones, like estradiol and testosterone, with specific morphological events like spermatogenesis and maturation of the testes. Klimley (2013) reported similar trends on captive and wild male Bonnetheads, *Sphyrna tiburo* (Linnaeus, 1758), where he observed a direct relationship between the calcification and length of the myxopterygia and increases in the concentration of testosterone and dishydrotestosterone. Within our sample of male specimens exhibiting gynandric teeth, 14 of 15 were classified as fully mature based on the extent of calcification of the myxopterygia. Although the exact endocrine process(es) that triggers the development of gynandric teeth within *R. terraenovae* is unknown, it is plausible their formation is also triggered by seasonal increases of these (or similar) reproductive hormones just prior to breeding season.

Specific breeding behaviors have not been documented for many elasmobranchs. However, mating scars observed by the current authors on female *R. terraenovae* specimens in the northern Gulf of Mexico indicate that males use their teeth to grasp females during copulation. The lower gynandric teeth function differently than non-gynandric teeth and, when present, these teeth form a clutching dental type rather than a cutting subtype (*sensu* Cappetta, 2012). The clutching function of these teeth likely helps to securely grasp a female during copulation (in a similar fashion documented in *H. sabinus* by Kajiura & Tricas (1996)), while at the same time reducing the potential for more significant injuries that could otherwise be caused by serrated teeth. It is our hypothesis that the mating scars observed on *R. terraenovae* females were caused by the serrated/irregular upper teeth (or lower lateral teeth) of the male, and we believe that a closer examination of fresh scars will reveal four to six subtle puncture marks formed by the clutching gynandric teeth.

### Implications on the fossil record

#### Intergeneric tooth comparisons

Our analysis of 126 *Rhizoprionodon terraenovae* jaw sets from the north-central Gulf of Mexico has shown there to be a significant amount of morphological variation within the dentitions of this taxon. These observations have direct implications on the fossil record of *Rhizoprionodon*, as there is tremendous ambiguity regarding the morphological characteristics used to identify fossil teeth of this genus. One of the primary issues regarding the study of fossil *Rhizoprionodon* is the morphological similarity of their teeth to those of extant *Loxodon*, *Scoliodon*, and some closely related sphyrnids. Prior studies have discussed these similarities (see Springer, 1964; Naylor, 1992; Li, 1997; Garry, 2003, 2004; Cappetta, 2012), and Cappetta (2012) noted that some fossil teeth assigned to *Rhizoprionodon* may in fact belong to one of the other genera (and *vice versa*). This lack of taxonomic clarity is largely due to the paucity of comprehensive studies on extant dentitions of these genera, which inhibits the determination of generic-level characteristics that would allow us to adequately differentiate isolated fossil teeth.

To improve our ability to accurately identify isolated *Rhizoprionodon* teeth in the fossil record, we compared our *R. terraenovae* sample to the dentitions of the extant species of *Eusphyra*, *Loxodon*, *Rhizoprionodon*, *Scoliodon*, and *Sphyrna* through jaw sets housed in the collections at McWane Science Center in Birmingham, USA (MSC) and the South Carolina State Museum in Columbia, USA (SC) (see Appendix 1.2 for a complete list of comparative jaw specimens), as well as to dentitions published in the literature (*i.e*., Springer, 1964; Gilbert, 1967; Compagno, 1984; Compagno, 1988; Ebert & Dando, 2021) The results of this interspecific and intergeneric analysis are provided below.

#### *Rhizoprionodon terraenovae vs* Rhizoprionodon spp.

Our examination of 126 *Rhizoprionodon terraenovae* dentitions shows that this taxon exhibits various types of heterodonty, including monognathic (gradational and disjunct) dignathic, ontogenetic, and seasonal gynandric. Tooth counts are also variable within this species, as 21% of our sample exhibit non-standard dental formulae and/or dental asymmetry. Our comparison of *R. terraenovae* jaws to those of the other six extant *Rhizoprionodon* species showed there to be no appreciable morphological tooth differences between the various taxa. Although some minor morphological differences were observed between the species, we could not determine if these characteristics are taxonomically significant because we cannot rule out the possibility that they reflect ontogeny and/or gynandric heterodonty. For example, the distal cutting edge of *R. acutus* (*i.e*., MSC 42591, SC96.77.9) is slightly more convex than on *R. terraenovae*, and the lower teeth of *R. longurio* have a slightly more upturned apex (*i.e*., MSC 46806). Our findings corroborate the work of Garry (2003, 2004) who, based on geometric morphometric analyses of the dentitions of five extant *Rhizoprionodon* species, could not identify significant morphological tooth differences among the taxa. Springer (1964) and Compagno (1984, 1988) noted some minor dental characteristics to differentiate the various species, like the presence of serrations on the teeth of mature *R. acutus* (MSC 42591), *R. longurio* (MSC 46806, SC2020.53.28), *R. porosus*, and *R. terraenovae*, and absence of serrated teeth in adult *R. lalandei*, *R. oligolinx*, and *R. taylori* (indicating teeth of the latter taxa have smooth cutting edges). However, as determined by our study, the presence or absence of serrated teeth in *R. terraenovae* is not taxonomically useful for identifying isolated teeth because its occurrence is ontogenetic and variable across the jaws, and it may be in other species as well. Springer (1964) and Compagno (1984, 1988) also used the presence or absence of gynandric heterodonty to differentiate the dentitions of the various species, with Compagno (1988:295) noting that this phenomenon was “hardly developed” in *R. acutus* and *R. longurio*, moderately developed in *R. lalandei*, and strong in *R. oligolinx*. Although Springer (1964) suggested that gynandric heterodonty was absent altogether in *R. longurio*, *R. porosus*, and *R. terraenovae*, our analysis of the latter species demonstrates the contrary and suggests this phenomenon also likely occurs in the other taxa, although it may be subtle in certain species.

Additional factors that inhibit accurate comparisons among the extant species are the ontogenetic and gynandric dental changes we observed in *R. terraenovae*, as these make determining useful interspecific tooth characteristics difficult without knowing the exact size class or sex of an individual (which is often not available with historically collected museum specimens). Despite these shortcomings, our analysis highlights the remarkable morphological similarity between the teeth of the extant *Rhizoprionodon* species. This in turn indicates that the dental morphology of *R. terraenovae* is largely reflective of the genus as a whole and can be utilized as a morphological baseline for intrageneric tooth comparisons.

#### Rhizoprionodon *vs* Loxodon and Scoliodon

Extant *Loxodon* and *Scoliodon* includes three species, *L. macrorhinus* Müller & Henle, 1839, *S. laticaudus* Müller & Henle, 1838, and *S. macrorhynchos* (Bleeker, 1852). Our analysis showed that extant *Rhizoprionodon* teeth can generally be separated from those of extant *Loxodon* and *Scoliodon* by a combination of the height and distal extent of the main cusp, and the nature of the cutting edges. As noted by Reinecke et al. (2011) and Ebersole, Cicimurri & Stringer (2019), *Rhizoprionodon* teeth differ from those of *Loxodon* and *Scoliodon* by having a relatively shorter main cusp, which does not extend to the distal edge of the crown base. In contrast, nearly all the teeth of *Loxodon* and *Scoliodon* (except those in the symphyseal and parasymphyseal files) have a main cusp that is more elongated than those in the corresponding files in *Rhizoprionodon*, and they have a main cusp apex that extends to, and sometimes beyond, the distal edge of the crown base. The nature of the cutting edges on the teeth is a less useful characteristic to separate these genera, as according to Springer (1964) and Compagno (1988), they are either serrated or irregular on *R. acutus*, *R. longurio*, *R. porosus*, and *R. terraenovae*, smooth on *R. lalandei*, *R. oligolinx*, and *R. taylori* teeth, irregular on mature *Loxodon*, and smooth on *Scoliodon*. However, we observed faint serrations on the mesial and distal edges of the teeth in the jaws of an extant *Scoliodon* jaw (MSC 46804), indicating this characteristic needs to be reevaluated for this taxon. The dentitions of mature male *Scoliodon* individuals also provide exceptions to the above observations.

Springer (1964) noted conspicuous gynandric heterodonty in the dentitions of *Scoliodon*, whereas it is generally more subtle in *Loxodon* and most of the species of *Rhizoprionodon*. In adult male *Scoliodon* dentitions, the teeth have a narrower, labiolingually thicker, and more sinuous main cusp compared to females and immature males (see Springer, 1964: fig. 3). Upper teeth of adult male *Scoliodon* can be differentiated from those in the corresponding files in *Rhizoprionodon* by having a taller and narrower main cusp that is distally extended beyond the distal edge of the crown base. Differentiating the lower mature male teeth of *Scoliodon* from those of *Rhizoprionodon* is more difficult because the main cusp on only the anterior and anterolateral teeth extends to or beyond the distal edge of the crown base. Nevertheless, *Scoliodon* teeth have a main cusp that is taller and narrower in the lateral and posterior lateral files than they are in *Rhizoprionodon*. The thicker, more cylindrical, and narrower main cusp on adult male *Scoliodon* teeth differentiate them from those of *Loxodon*, which have a main cusp that is wider and more labiolingually compressed. Female and non-mature male *Scoliodon* teeth are extremely difficult to differentiate from those of *Loxodon*, as are the posterior-most teeth of all three genera.

#### Rhizoprionodon *vs* Sphyrnidae

Extant Sphyrnidae includes two genera, *Eusphyra* and *Sphyrna*, and ten extant species—*E. blochii* (Cuvier, 1816), *S. corona* Springer, 1940, *S. gilberti* Quattro et al., 2013, *S. lewini* (Griffith & Smith, 1834), *S. media* Springer, 1940, *S. mokarran* (Rüppell, 1837), *S. tiburo* (Linnaeus, 1758), *S. tudes* (Valenciennes, 1822), *S. vespertina* Springer, 1940, and *S. zygaena* (Linnaeus, 1758). Others have noted the difficulty of separating isolated Sphyrnidae teeth from those of *Rhizoprionodon* (Springer, 1964; Naylor, 1992; Garry, 2003), and compounding the issue are the extremely varied dentitions of extant Sphyrnidae (see Gilbert, 1967; Compagno, 1984; Compagno, 1988; Ebert & Dando, 2021), which fall into several adaptive dental types (*sensu* Cappetta, 2012). Although it was not possible for us to determine a specific list of generic dental differences from *Rhizoprionodon* without undertaking similar studies on the various Sphyrnidae taxa, we do provide generalized characteristics that can be used to separate the various taxa on the species-level. Nevertheless, it must be noted that the posterior-most teeth of the various sphyrnids are morphologically very similar to those of *Rhizoprionodon*, and it is possible that isolated posterior teeth cannot be differentiated unless directly associated with other, more diagnostic, teeth.

Of the various species of Sphyrnidae, teeth of *S. mokarran* (*i.e*., SC2000.120.2) are unlike those of *Rhizoprionodon* and more comparable to certain species of *Carcharhinus*, like *C. leucas* (Valenciennes in Müller & Henle (1839)) (*i.e*., MSC 42586) and *C. obscurus* (Lesueur, 1818) (*i.e*., MSC 42614, SC86.186.1), in that they are rather broadly triangular and coarsely serrated. The upper and lower teeth of *S. zygaena* (*i.e*., MSC 42600) differ from *Rhizoprionodon* by being very robust in all jaw positions, and by having a mesiodistally wider main cusp and a more delineated mesial heel. The dentition of the recently described *S. gilberti* has yet to be published, but it is assumed the teeth are morphologically similar to those of the closely related *S. lewini*.

Several sphyrnids have teeth that are similar to those of *Rhizoprionodon*, including *E. blochii*, *S. corona*, *S. lewini*, *S. media*, *S. tiburo*, and *S. vespertina*. The greatest mesiodistal width of *Rhizoprionodon* teeth is generally less than 7 mm (see Appendices 1.3–1.6), which is smaller than adult teeth of most of the aforementioned sphyrnid species. In addition, when we compared the teeth of *S. media* and *S. lewini* (*i.e*., MSC 42805, MSC 46805, SC2000.120.4) to *Rhizoprionodon* we found that they have a comparatively larger and more erect cusp (particularly in the lower files) that is more conspicuously differentiated from the remainder of the crown. However, a juvenile *S. lewini* specimen that we examined (MSC 46805) has lower lateral teeth that are morphologically very similar to those of *Rhizoprionodon*, making the identification of isolated teeth within this age class difficult. This phenomenon compounds the difficulty of separating the two genera, as juvenile teeth of at least some *Sphyrna* species could be misidentified as adult teeth of *Rhizoprionodon*. *Rhizoprionodon* upper teeth have a taller main cusp and a taller and more variable distal heel that can be rounded, triangular, or bifurcated, as opposed to low and rounded on all *E. blochii* and *S. tiburo* (*i.e*., MSC 42603, SC96.77.3) teeth observed. On *S. corona*, the main cusp on the upper teeth extends to, or beyond, the distal edge of the crown, whereas it falls short of the distal edge on *Rhizoprionodon*. The main cusp on *S. tiburo* lower teeth is narrower and more erect than on *Rhizoprionodon*, and a main cusp is absent on several of the posterior-most teeth. Unfortunately, no jaw sets or illustrations of unequivocal *S. vespertina* were available for study. However, because this taxon was formerly viewed as a subspecies of *S. tiburo* (see Compagno, 1988), the two are presumed to have very similar dentitions. The lower teeth of *S. corona* have a narrower main cusp compared to *Rhizoprionodon*, and the distal heel is low with an almost flat occlusal surface (as opposed to being taller and variable in shape on *Rhizoprionodon*). Many tooth positions within the lower jaws of *E. blochii* are morphologically indistinguishable from those of *Rhizoprionodon*, but whereas all *E. blochii* teeth have smooth cutting edges, the teeth of most of the extant species of *Rhizoprionodon* have either irregular or serrated cutting edges. Regarding the cutting edges on the teeth of the various sphyrnid taxa, they appear strongly serrated on *S. mokarran* teeth (SC2000.120.2), weakly serrated on *S. zygaena* teeth (*i.e*., MSC 42600), smooth on most *S. lewini* teeth (with weak serrations occurring on teeth of large individuals, *i.e*., MSC 42605, MSC 46805), and smooth on all the teeth of *E. blochii, S. media, S. tudes, S. corona*, and *S. tiburo* (*i.e*., MSC 42603, SC96.77.3) (Gilbert, 1967).

All of these morphological comparisons considered, the various genera of extant Sphyrnidae and *Rhizoprionodon* are identified by their body shapes, and we have the benefit of examining the associated dentition to help identify isolated teeth of the extant taxa. The utility of these features is less obvious in the fossil record, particularly for taxa that are currently unknown from fossils. We could not identify a singular or combination of features that would allow us to confidently separate teeth of *Rhizoprionodon* from Sphyrnidae without considering our knowledge of these extant taxa. Small size alone should not be the morphological criterion used to identify teeth, as it cannot be known with certainty if a small tooth represents *Rhizoprionodon*, or a juvenile individual (*i.e*., *S. lewini*) or diminutive species (*i.e*., *S. tiburo*) of Sphyrnidae. Additionally, the morphological features we identified on *Rhizoprionodon* teeth, like the shape of cutting edges, shape of the distal heel, smooth/irregular/serrated cutting edges, cusp width and inclination, are variable within and among species. These features overlap with those of *E. blochii* (Sphyrnidae), and we consider it highly unlikely that one could accurately identify an early Pleistocene tooth as *E. blochii*, rather than a species of *Rhizoprionodon*. Although tooth morphology alone cannot clarify the taxonomy of all fossil teeth, when other data is considered, like extant range and molecular and morphological phylogenies, the taxonomy for many fossil *Rhizoprionodon*-like teeth becomes clearer.

#### The phylogenetic position and origin of Rhizoprionodon

The phylogenetic position of *Rhizoprionodon* remains unresolved. Compagno (1988) suggested two phylogenetic hypotheses for the genus based on morphological characteristics, the first being that *Rhizoprionodon* and *Loxodon* belong to a group, the Rhizoprionodontini, that is a sister group to the higher carcharhinids. The second hypothesis suggested that *Loxodon* was the most basal carcharhinid, and *Rhizoprionodon* represented a sister group to the higher taxa within the Carcharhiniformes. Molecular analyses by Naylor (1989, 1992) also pointed to a basal position for *Rhizoprionodon*, indicating that it, along with *Galeocerdo*, are the most basal genera in the clade. Musick, Harbin & Compagno (2004) suggested a hybrid phylogeny for the Carcharhinidae that combined the morphological hypotheses of Compagno (1988) with refinements indicated by the molecular analyses of Naylor (1989, 1992). This hybrid phylogeny places *Rhizoprionodon*, *Galeocerdo*, and *Scoliodon* as basal to the rest of the Carcharhinidae, followed by a group comprised of *Negaprion*, *Loxodon*, and *Triaenodon*, with *Carcharhinus* and *Prionace* being the most derived taxa. Herman, Hovestadt-Euler & Hovestadt (1991) proposed a similar phylogeny based on tooth vascularization, morphology, and fossil occurrences. However, these latter authors suggested that *Galeocerdo* was the most basal carcharhinid, *Rhizoprionodon* and *Scoliodon* split from the *Galeocerdo*-lineage during the Eocene, and *Sphyrna*, *Eusphyra*, *Negaprion*, *Isogomphodon*, *Triaenodon*, *Loxodon* and *Leptocharias* were derived from *Rhizoprionodon* and *Scoliodon*, with *Sphyrna* and *Eusphyra* diverging as late as the Pliocene. This particular phylogeny could explain the close similarity in tooth morphology between *Rhizoprionodon* and Sphyrnidae.

**Figure 18:**
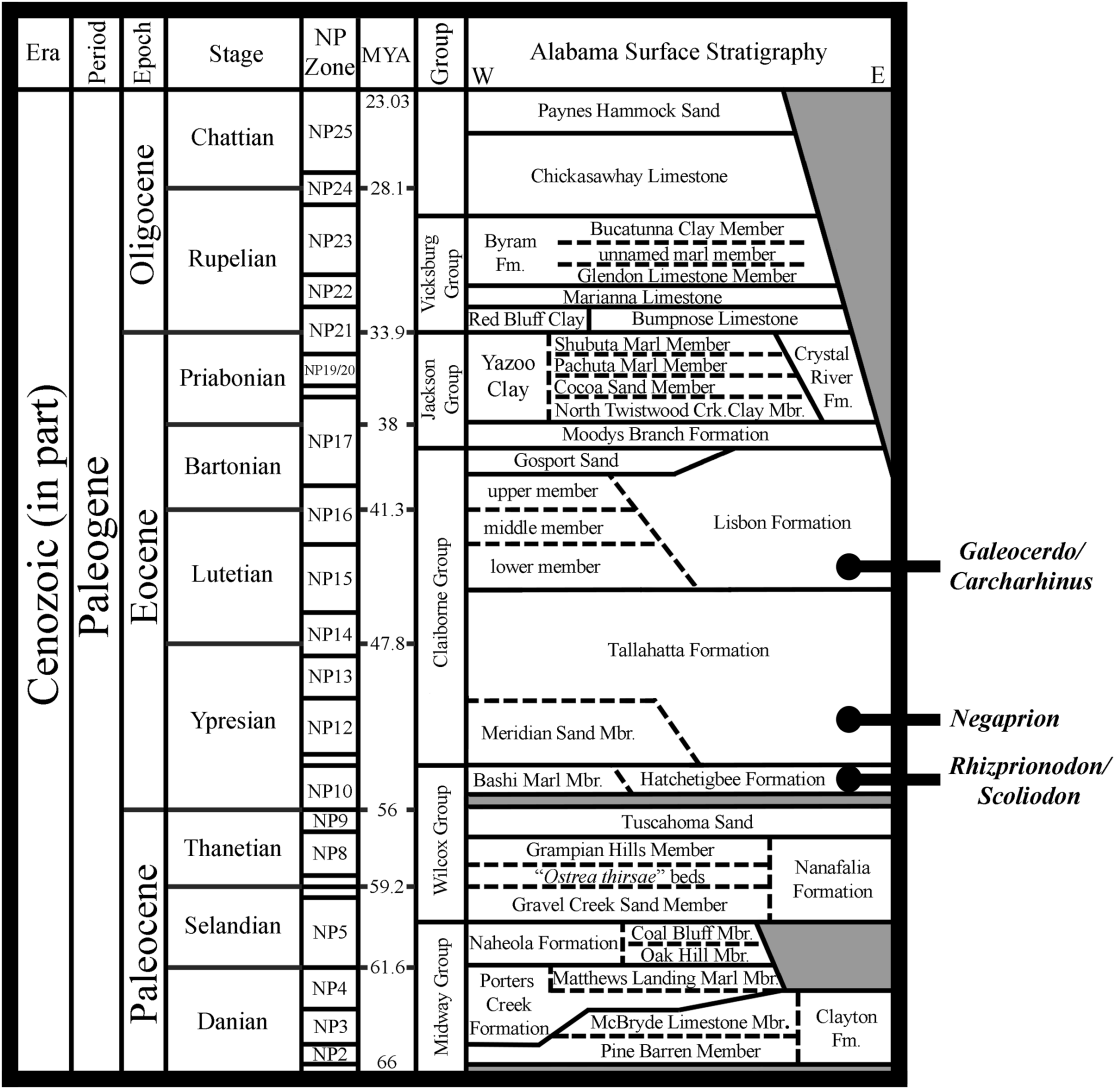
First occurrences of unequivocal fossil *Rhizoprionodon*, *Scoliodon*, *Negaprion*, *Galeocerdo*, and *Carcharhinus* in Paleogene strata in Alabama, USA. Acronyms: MYA, millions of years ago; E, east; W, west; NP Zone, Nannoplankton Zone. Grey shaded areas represent unconformities.

The hypothesized basal position of *Rhizoprionodon*, as inferred by these molecular and morphological analyses, is supported by first occurrences of specific genera in the fossil record (see Cappetta, 2012), specifically in Alabama, a state with a nearly complete Paleogene marine sequence. Based on specimens in the MSC collection, unequivocal *Rhizoprionodon* and *Scoliodon* teeth first appear in Alabama in the lower Ypresian Hatchetigbee Formation (~55 mya), *Negaprion* appears shortly after in the middle Ypresian component of the Tallahatta Formation (~50 mya), and *Galeocerdo* and *Carcharhinus* first appear in the middle Lutetian component of the Lisbon Formation (~45 mya; Fig. 18).

**Figure 19:**
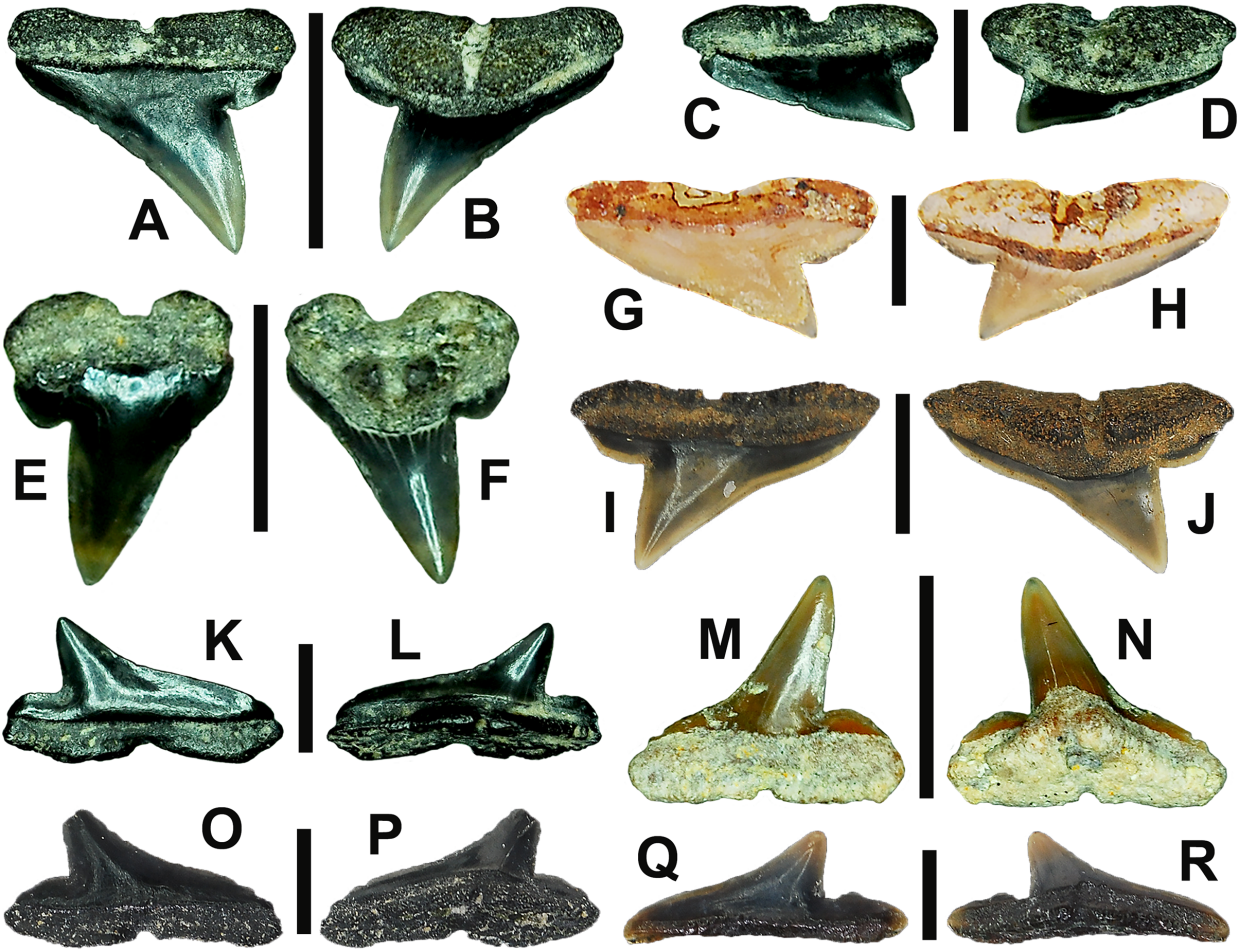
Paleogene †*R hizoprionodon ganntourensis* teeth from Alabama, USA. (A and B) MSC 2173.6, Gosport Sand, Clarke County, AL, USA, left upper anterior tooth in (A) labial and (B) lingual views, scale bar = 5 mm. (C and D) MSC 38504.9, Gosport Sand, Clarke County, AL, USA, left upper posterior tooth in (C) labial and (D) lingual views, scale bar = 3 mm. (E and F) MSC 37956.2, Hatchetigbee Formation, Butler County, AL, USA, right upper 1st anterior tooth in (E) labial and (F) lingual views, scale bar = 2 mm. (G and H) MSC 37907.1, Tallahatta Formation, Dale County, AL, USA, left upper lateral tooth in (G) labial and (H) lingual views, scale bar = 2 mm. (I and J) MSC 37098, contact of the Tallahatta and Lisbon Formations, Covington County, AL, USA, right upper lateral tooth in (I) labial and (J) lingual views, scale bar = 3 mm. (K and L) MSC 38504.7, Gosport Sand, Clarke County, AL, USA, right lower posterior tooth in (K) labial and (L) lingual views, scale bar = 2 mm. (M and N) MSC 35753.1, Tallahatta Formation, Dale County, AL, USA, lower left gynandric anterior tooth in (M) labial and (N) lingual views, scale bar = 4 mm. (O and P) MSC 2173.4, Gosport Sand, Clarke County, AL, USA, right lower lateral tooth in (O) labial and (P) lingual views, scale bar = 2 mm. (Q and R) MSC 37674.1, Lisbon Formation, Covington County, AL, USA, left lower posterior tooth in (Q) labial and (R) lingual views, scale bar = 2 mm.

Using the morphological criteria outlined above for identifying *Rhizoprionodon*-like taxa, the temporally oldest unequivocal *Rhizoprionodon* teeth are those of †*R. ganntourensis*, a taxon originally described from early Eocene (Ypresian) deposits in Morocco (Arambourg, 1952). This species has since been reported from lower and middle Eocene units in Belgium (Iserbyt & De Schutter, 2012), China (Li, 1997), France (Cappetta & Nolf, 1981; Dutheil, 1991), Jordan (Mustafa et al., 2005), Madagascar (Samonds et al., 2019), Morocco (Noubhani & Cappetta, 1997), Romania (Dica, 2005; Trif, Codrea & Arghius, 2019), Togo (Cappetta & Traverse, 1988), and Uzbekistan (Case et al., 1996), as well as Alabama (Ebersole, Cicimurri & Stringer, 2019) and South Carolina (Cicimurri & Knight, 2019) in the USA. Although the *Rhizoprionodon* teeth figured in these various studies show them to be morphologically similar to one another, some variation can be observed with respect to the morphology of the distal heel, as it is rounded on some specimens but triangular and/or bifurcated on others. Ebersole, Cicimurri & Stringer (2019) noted similar morphological variation on the illustrated †*R. ganntourensis* syntypes from Morrocco (Arambourg, 1952: pl. 24, figs. 49–63, text fig. 33), and utilized this characteristic to assign their middle Eocene teeth from Alabama to this taxon (Fig. 19). Because we observed this same variation in the *R. terraenovae* jaws in the present study, the varied distal heel morphology on †*R. ganntourensis* teeth can likely be attributed to intraspecific variation (*i.e*., ontogenetic heterodonty) and is therefore not necessarily an indication of multiple coeval species. Gynandric †*R. ganntourensis* teeth have also been identified in Alabama (see Figs. 19M, 19N). These teeth, along with the variable distal heel morphology observed in this taxon, indicate a degree of uniformitarianism between †*R. ganntourensis* and extant *R. terraenovae*, as their teeth appear to share similar patterns of ontogenetic and gynandric development.

The overall morphology of †*R. ganntourensis* teeth is very similar to those of extant *R. terraenovae*, so much so that isolated fossil teeth of the former can be confidently assigned to the upper and lower tooth groups defined herein for the latter (Table 1). The primary morphological difference between these two taxa is the apparent absence of serrated or irregular cutting edges on all †*R. ganntourensis* teeth (Fig. 19). Both Springer (1964) and Compagno (1984, 1988) noted serrated and/or irregular cutting edges on adult representatives of several species of extant *Rhizoprionodon*, and the current study indicates that the acquisition of serrated and/or irregular cutting edges on *R. terraenovae* teeth is ontogenetic and influenced by ontogenetic dietary shifts. However, because serrations and/or irregular cutting edges appear absent on †*R. ganntourensis* teeth regardless of size, its absence cannot be attributed to ontogeny. This suggests that the ontogenetic development of serrations and/or irregular cutting edges on fossil *Rhizoprionodon* teeth happens later in its evolutionary lineage, a phenomenon documented in other elasmobranch taxa like †*Otodus* (see Cappetta, 2012; Ebersole, Cicimurri & Stringer, 2019). Our data on *R. terraenovae* dentitions shows a strong relationship between the acquisition of serrated teeth with an increased dietary reliance on fishes as the shark matures. This could suggest a change in dietary preference for *Rhizoprionodon* through time, with the evolutionary development of serrations being driven by an increase in fish consumption in the more derived (likely Neogene) species. In other words, the apparent lack of serrations on †*R. ganntourensis* teeth could indicate a diet heavy in soft-bodied prey rather than bony fishes.

To our knowledge, the only other recognized Eocene *Rhizoprionodon* species is †*R. bisulcatus* Li, 1997, a taxon based on a single incomplete tooth derived from late Eocene deposits in the Western Tarim Basin in China. Li (1997) differentiated this taxon based on the occurrence of two nutritive grooves on the lingual root face. However, it is impossible to determine from a single tooth if this characteristic is apomorphic or a result of intraspecific variation. As part of his diagnosis, Li (1997:120) noted the similarity of extant *Rhizoprionodon*, *Scoliodon*, and *Loxodon* teeth, but differentiated the teeth of these genera based on the “straight basal line of the root,” which he observed as being straighter on *Rhizoprionodon*. However, our examination of extant dentitions revealed this characteristic to be ambiguous at best, as the basal concavity on the teeth of all three genera is variable and can be straight or shallow and V-shaped or U-shaped. Our comparison of dentitions indicates that the width and distal extent of the main cusp is a much more reliable characteristic to differentiate *Rhizoprionodon* from *Loxodon* and *Scoliodon* (as well as certain sphyrnid taxa). An examination of the line drawing provided by Li (1997, figs. 6f–6h) of the †*R. bisulcatus* holotype shows that the tooth is likely from a lower lateral file, and the occurrence of a tall and narrow main cusp that extends to the distal edge of the tooth indicates it is better aligned with *Loxodon* and *Scoliodon* than to *Rhizoprionodon*. Additional specimens are needed from the type locality to help elucidate the true affinity of the †*R. bisulcatus* morphology.

Because the presence of a second Eocene *Rhizoprionodon* species cannot be substantiated, †*R. ganntourensis* remains the only valid Eocene member of the genus. We consider two hypotheses regarding the singular nature of this taxon during the Eocene. The first is that several *Rhizoprionodon* species were present during the Paleogene, but these additional taxa have yet to be discovered, or the dentitions are so generalized that multiple species cannot be readily differentiated from one another based on isolated teeth (as is the case with the teeth of the extant *Rhizoprionodon* species). It is also possible that additional Eocene species have been recovered but have been assigned to other morphologically similar genera, like *Scoliodon* or even Sphyrnidae. Our second hypothesis is that †*R. ganntourensis* represents the most basal member of the genus, and is the only Eocene representative of *Rhizoprionodon*, and this taxon obtained a nearly circumglobal distribution by the middle Eocene. This second hypothesis is supported by Musick, Harbin & Compagno (2004) and Gallo et al. (2010), who suggested that the present distribution of the extant *Rhizoprionodon* species was the result of a widespread Tethyan taxon that was influenced by successive vicariant events resulting from the collision of Africa and Asia and the formation of the Suez land barrier during the Oligocene through Miocene. This is also supported by the widespread distribution of †*R. ganntourensis*-like teeth during the Eocene (see above occurrences).

The †*R. ganntourensis* syntypes were derived from middle Eocene (Lutetian) deposits in the Ganntour Basin of Morocco (Arambourg, 1952). Noubhani & Cappetta (1997) investigated the elasmobranchs at the same locality and subsequently confirmed the occurrence of †*R. ganntourensis* in slightly older upper Ypresian deposits at the site (likely Nannoplankton (NP) zones 12/13). Iserbyt & De Schutter (2012) later confirmed this taxon within the Roubaix Clay Member of the Kortrijk Clay Formation (just below the base of Aalbeke Clay Member) at the Marke clay pit in western Belgium. This latter occurrence is of interest because the stratigraphic position of the teeth indicates they were collected from the base of Zone NP12 (see Steurbaut & King, 2017: fig. 2), representing the stratigraphically oldest †*R. ganntourensis* teeth in the published record. Collectively, these records show that †*R. ganntourensis* was well established in the Tethys region by the early Eocene.

Ebersole, Cicimurri & Stringer (2019) reported the occurrence of †*R. ganntourensis* from several middle Eocene units in Alabama, and recently several unpublished †*R. ganntourensis* teeth were confirmed by one of the authors (JAE) in the MSC collection that were derived from the base of the Ypresian Hatchetigbee Formation in Lauderdale County, Mississippi and Butler County, Alabama, USA (Figs. 19E, 19F), a stratigraphic interval that resides almost entirely within Zone NP10 (Dockery & Thompson, 2016: fig. 343; Fig. 18). These Alabama and Mississippi specimens represent the stratigraphically oldest known records of *Rhizoprionodon* and demonstrate that this genus was present in the ancient Gulf of Mexico of the USA during the earliest Ypresian.

#### Taxonomy of fossil Rhizoprionodon

We conclude that the extinct †*R. ganntourensis*-type morphology is the only Eocene morphotype for the genus. We state this with the understanding that multiple coeval *Rhizoprionodon* species may have been present during the Eocene, but their generalized tooth morphology does not allow them to be identified at this time based on teeth alone. Nevertheless, the estimated early Eocene divergence of *Rhizoprionodon* from a carcharhinid ancestor (Martin, 1992) is corroborated by the occurrence of Ypresian †*R. ganntourensis* teeth in the fossil record, and the morphological similarity of these fossil teeth to those of extant *Rhizoprionodon* suggests their placement within this genus is appropriate. Although a second Eocene species, †*R. bisulcatus*, has been reported (Li, 1997), this taxon is based on a single incomplete tooth that may be more appropriately assigned to *Loxodon* or *Scoliodon*. Additional specimens are needed to validate this taxon and to elucidate its generic affinities. Additional Eocene *Rhizoprionodon* taxa may have been described, but mistakenly assigned to *Scoliodon*, *Loxodon*, *Sphyrna*, or even the extinct genus *Physogaleus*. Evaluating all of these records is outside of the scope of the present work, but we suggest that all Paleogene occurrences of the aforementioned genera be reevaluated using the morphological criteria outlined in this study.

A stratigraphically younger fossil species, †*Rhizoprionodon ficheuri* (Joleaud, 1912), has been reported from Oligocene to Pliocene deposits in Africa, Europe, and North America. Oligocene occurrences include Germany (Freess & Möller, 1993) and Virginia and North Carolina (Müller, 1999) in the USA. Miocene records include Austria (Hiden, 1995; Schultz, 2013), Costa Rica (Laurito, 1999), France (Joleaud, 1912; Cappetta, 1970; Brisswalter, 2009; Vialle, Adnet & Cappetta, 2011), Germany (Barthelt et al., 1991; Bracher, 2000; Bracher & Unger, 2007; Pollerspöck & Straube, 2017; Höltke et al., 2022), Madagascar (Andrianavalona et al., 2015), Spain (Mendiola & López, 2005), and Portugal (Balbino, 1996; Fialho et al., 2020), as well as Maryland (Visaggi & Godfrey, 2010) and Florida (Boyd, 2016) in the USA. Pliocene occurrences include Costa Rica (Laurito, 1999), Italy (Marsili, 2008), and Spain (Mora Morote, 1996; Mas, 2010). Based on these reports, it would seem that the species was not only long-lived, but nearly circumglobally distributed.

Joleaud (1912) originally named this taxon †*Carcharias* (*Physodon*) *ficheuri* based on four teeth recovered from middle Miocene deposits in Hérault, France. However, an examination of his illustrated syntypes (Joleaud, 1912: pl. 6.1–11) shows that they appear to represent a heterogeneous mix of at least three different taxa. The tooth illustrated in Joleaud (1912: figs. 1–3) is of a morphology that is not unlike certain scyliorhinids, and has a morphology that falls outside the *Rhizoprionodon*, *Scoliodon*, and *Loxodon* specimens we examined. The tooth illustrated in Joleaud (1912: figs. 4–6) exhibits characteristics that are consistent with *Loxodon* and *Scoliodon*, but as drawn the tooth also bears some similarity to *Physogaleus*, and we therefore exclude this morphology from the †*R. ficheuri* hypodigm. The remaining two teeth illustrated in Joleaud (1912: figs. 7–11) are consistent with *Rhizoprionodon* and could be identified as lower lateral (figs. 7, 8) and posterior teeth (figs. 10, 11).

Due to the morphological ambiguity surrounding †*R. ficheuri*, we examined figures of additional specimens assigned to the species by Cappetta (1970, 2012) that were derived from the type locality. The †*R. ficheuri* teeth figured by Cappetta (1970: pl. 15, figs. 18–27; pl. 16, figs. 1–4) all have a tall and narrow main cusp that is very similar to those of extant mature male *Scoliodon* and likely belong to this latter genus. Interestingly, Cappetta (1970: pl. 16, figs. 5–22) also figured a number of teeth from the same site as †*Scoliodon taxandriae* that have a morphology consistent with extant *Rhizoprionodon* teeth. Cappetta (2012: fig. 283) later illustrated three teeth from the site consistent in morphology with the †*Scoliodon taxandriae* teeth he figured in 1970 but referred these teeth to †*R. ficheuri*. This leads us to believe that a middle Miocene species of both *Rhizoprionodon* and *Scoliodon* are present at the †*R. ficheuri* type locality in Hérault, France, and their similar morphologies have resulted in them being described as a single taxon. In addition, the varied tooth morphologies figured as †*R. ficheuri* by Joleaud (1912) and Cappetta (1970, 2012), combined with the long stratigraphic occurrence of reported teeth of this taxon (Oligocene to Pliocene, see references above), suggests that †*R. ficheuri* has become a “waste-basket” taxon. We therefore recommend that any Oligo-Miocene †*R. ficheuri* or *Rhizoprionodon*-like teeth be reevaluated based on the morphological criteria presented herein because they may belong to *Scoliodon*, *Loxodon*, or even multiple species of *Rhizoprionodon*. We also recommend that a large sample of *Rhizoprionodon* teeth from the †*R. ficheuri* type locality be reexamined and described to provide a better understanding of the †*R. ficheuri* morphology.

The taxonomy of Neogene *Rhizoprionodon* is less clear. As far as the present authors are aware, molecular divergence times have not been estimated for the extant *Rhizoprionodon* species, and few reliable reports of these species can be found in the fossil record. Lim et al. (2022) suggested a relatively recent divergence for the closely related extant *Scoliodon* species, and Lim et al. (2010) estimated an early to middle Miocene divergence for the extant sphyrnids. The morphological dental similarities between *Rhizoprionodon*, *Scoliodon*, and some of the sphyrnids (especially *Eusphyra*) suggest that the extant *Rhizoprionodon* species also diverged during the Miocene (as was suggested by Herman, Hovestadt-Euler & Hovestadt (1991)). This late divergence could offer a plausible explanation for the morphological similarity between the dentitions of the extant *Rhizoprionodon* species and our inability to adequately differentiate them based on tooth morphology alone. Perhaps ca. 8 Ma was enough time for the genus to become geographically isolated and local populations to evolve into the various species (that can be identified by body characteristics), but the tooth morphology remained consistent among the species. If a Miocene divergence of the seven extant species from a common *Rhizoprionodon* ancestor is correct, Paleogene fossils must then belong to a basal member of the genus, and the usage of extant species names should be reserved for fossil teeth from the Neogene to Pleistocene. However, due to the morphological similarity of extant *Rhizoprionodon* teeth and our lack of knowledge of the paleobiogeographic ranges of the extant species, it may be best to refrain from assigning a species name to Neogene *Rhizoprionodon* teeth. A less desirable option is to tentatively (*i.e*., cf.) identify species based on the geographic range of the closest occurring living representative of the genus (*i.e*., *R*. cf. *R. terraenovae* in southern Alabama, rather than *R*. cf. *R. acutus*).

The same rationale can be applied to the usage of *Scoliodon* in the fossil record. Based on specimens in the MSC collection, the earliest occurrence of an unequivocal member of the genus are teeth of †*Scoliodon conecuhensis* Cappetta & Case, 2016 that first appear in Ypresian deposits in Alabama (Fig. 18). The remarkable similarity of these teeth to those of extant *Scoliodon* suggests they represent a stem member genus that split off from a carcharhinid ancestor at roughly the same time as *Rhizoprionodon*. Although the diversity of Paleogene *Scoliodon* is unknown, it should be noted that some fossil teeth assigned to *Scoliodon* may belong to *Loxodon* or *Rhizoprionodon*. Nevertheless, Herman, Hovestadt-Euler & Hovestadt (1991) and Lim et al. (2022) suggested a late divergence for the extant *Scoliodon* species, indicating that Neogene occurrences may belong to one of the extant taxa. Finally, the usage of *Sphyrna* in the fossil record should be used with caution. If Lim et al. (2010) is to be followed, extant *Sphyrna* and *Eusphyra* split from a carcharhinid ancestor at some time in the early to late Miocene. This then suggests that any Paleogene taxa assigned to one of these genera instead belong to an unknown stem-sphyrnid or to a morphologically similar taxon like *Rhizoprionodon*, *Loxodon*, or *Scoliodon*. Usage of the extant genera *Sphyrna* and *Eusphyra* should then be reserved for post-middle Neogene occurrences, after the time of divergence.

## Conclusions

The current analysis of *R. terraenovae* jaws has increased our knowledge of the dental variability of this species and the genus as a whole. Understanding this morphological variability, along with the establishment of generic-level differential characteristics for *Rhizoprionodon*, *Loxodon*, *Scoliodon*, and, to a lesser degree, sphyrnids, helps provide a level of clarity regarding the taxonomy and origins of fossil *Rhizoprionodon*. It is our recommendation that all fossil species previously assigned to *Rhizoprionodon*, *Loxodon*, *Scoliodon*, and *Sphyrna* be reexamined utilizing the data presented herein, which hopefully will further clarify the fossil diversity of all four genera. However, due to the morphological similarity of the teeth of these various taxa, we recommend that other lines of evidence, like extant range, molecular divergence estimates, and morphological phylogenies based on external characteristics, be consulted to provide guidance as to the usage of extant generic names in the fossil record. We also recommend that similar dental studies be carried out on the remaining extant *Rhizoprionodon* species to better understand the true range of dental morphologies within different regions and across the genus as a whole. It is our hope that such studies will allow us to establish species-level tooth characteristics to help accurately identify isolated teeth of fossil and extant *Rhizoprionodon*, and to help us better understand the paleobiodiversity, origins, and evolutionary history of the genus.

## Supplemental Information

10.7717/peerj.15142/supp-1 Supplemental Information 1 Raw data.

## Additional Information and Declarations

### Data Availability

The following information was supplied regarding data availability: The raw data is available in the Supplemental Files.
