# Supplementary material for: Observations on heterodonty within the dentition of the Atlantic Sharpnose Shark, Rhizoprionodon terraenovae (Richardson, 1836), from the north-central Gulf of Mexico, USA, with implications on the fossil record
Source: PeerJ. 2023 Apr 12;11:e15142. doi: 10.7717/peerj.15142 (PMC10105564; doi:10.7717/peerj.15142)
Supplement: Supplemental Information 1 [file peerj-11-15142-s001.docx]

**Supplemental data for:**

**Observations on heterodonty within the dentitions of the Atlantic Sharpnose Shark, *Rhizoprionodon terraenovae* (Richardson, 1836), from the north-central Gulf of Mexico, USA, with implications on the fossil record**

Jun A Ebersole^1^, Abigail T Kelosky^1^, Bryan L Huerta-Beltrán ^2^, David J Cicimurri^3^, J Marcus Drymon^4,5^

^1^ Collections Department, McWane Science Center, Birmingham, AL, USA

^2^ School of Biological, Environmental, and Earth Sciences, University of Southern Mississippi, Hattiesburg, MS, USA

^3^ Natural History Department, South Carolina State Museum, Columbia, SC, USA

^4^ Coastal Research and Extension Center, Mississippi State University, Biloxi, MS, USA

^5^ Mississippi Alabama Sea Grant Consortium, Ocean Springs, MS, USA

**Appendix 1.1: Specimen Data (n=126)**

|  | **MSC Number** | **Fish ID** | **Sex** | **Date Collected** | **Lifestage** | **STL (mm)** | **Pal DF** | **Mec DF** | **Notes** |
| --- | --- | --- | --- | --- | --- | --- | --- | --- | --- |
| **1** | MSC 42637 | PS051719BL01-014 | Male | 5/17/2019 | Immature | 750 | 12-1-12 | 11-2-11 |  |
| **2** | MSC 42638 | PS051719BL01-011 | Female | 5/17/2019 | N/A | 675 | 12-?-12 | 11-2-11 |  |
| **3** | MSC 42639 | PS051719BL01-008 | Male | 5/17/2019 | Immature | 650 | 12-?-12 | 12-2-11 |  |
| **4** | MSC 42640 | PS051719BL01-005 | Male | 5/17/2019 | Immature | 605 | 12-1-12 | 11-2-11 |  |
| **5** | MSC 42641 | PS051719BL01-009 | Male | 5/17/2019 | Immature | 635 | 12-1-12 | 11-2-11 |  |
| **6** | MSC 42643 | PS051719BL01-004 | Male | 5/17/2019 | Immature | 565 | 12-1-12 | 11-2-11 |  |
| **7** | MSC 42644 | PS052119BL01-001 | Male | 5/21/2019 | Mature | 955 | 12-1-12 | 11-2-11 | Sperm present |
| **8** | MSC 42645 | PS051719BL01-003 | Female | 5/17/2019 | N/A | 610 | 12-1-12 | 11-2-11 |  |
| **9** | MSC 42646 | PS051719BL01-006 | Male | 5/17/2019 | Immature | 775 | 12-1-12 | 11-2-11 |  |
| **10** | MSC 42647 | PS051719BL01-007 | Male | 5/17/2019 | Immature | 665 | 12-1-12 | 11-2-11 |  |
| **11** | MSC 42648 | PS051719BL01-019 | Male | 5/17/2019 | Immature | 680 | 12-1-12 | 11-2-11 |  |
| **12** | MSC 42649 | PS051719BL01-010 | Female | 5/17/2019 | N/A | 600 | 12-1-12 | 11-2-11 |  |
| **13** | MSC 42650 | PS051719BL01-015 | Male | 5/17/2019 | Immature | 645 | 12-2-12 | 11-2-11 |  |
| **14** | MSC 42651 | PS051719BL01-018 | Male | 5/17/2019 | Immature | 675 | 12-1-12 | 11-2-11 |  |
| **15** | MSC 42652 | PS051719BL01-013 | Male | 5/17/2019 | Immature | 540 | 12-1-12 | 11-2-11 |  |
| **16** | MSC 42653 | PS051719BL01-020 | Male | 5/17/2019 | Immature | 620 | 12-1-12 | 11-2-11 |  |
| **17** | MSC 42654 | PS051719BL01-016 | Male | 5/17/2019 | Immature | 650 | 12-1-12 | 11-1-11 |  |
| **18** | MSC 42655 | PS051719BL01-012 | Female | 5/17/2019 | N/A | 625 | 12-1-12 | 11-2-11 |  |
| **19** | MSC 42656 | PS052119BL01-002 | Male | 5/21/2019 | Mature | 895 | 12-1-12 | 11-2-11 | Sperm present |
| **20** | MSC 42657 | PS051719BL01-002 | Male | 5/17/2019 | Immature | 635 | 12-1-12 | 11-2-11 |  |
| **21** | MSC 42658 | PS051719BL01-017 | Male | 5/17/2019 | Transitional | 790 | 12-1-13 | 11-2-11 |  |
| **22** | MSC 42659 | PS051719BL01-021 | Male | 5/17/2019 | Immature | 650 | 12-?-12 | 11-2-11 |  |
| **23** | MSC 42660 | PS051719BL01-022 | Male | 5/17/2019 | Immature | 735 | 12-1-12 | 11-2-11 |  |
| **24** | MSC 42661 | PS052219BL03-004 | Male | 5/22/2019 | Immature | 590 | 13-1-13 | 12-1?-12 |  |
| **25** | MSC 42662 | PS052219BL03-005 | Male | 5/22/2019 | Immature | 605 | 12-1-12 | 11-2-11 |  |
| **26** | MSC 42663 | PS052219BL03-008 | Male | 5/22/2019 | Immature | 610 | 12-1-12 | 11-2-11 |  |
| **27** | MSC 42664 | PS052219BL03-003 | Male | 5/22/2019 | Immature | 640 | 12-1-12 | 11-2-11 |  |
| **28** | MSC 42665 | PS052219BL03-007 | Male | 5/22/2019 | Immature | 590 | 12-1-12 | 11-2-11 |  |
| **29** | MSC 42666 | AD052119BL01-023 | Female | 5/21/2019 | Mature | 960 | 12-1-12 | 11-2-11 | Pregnant with pups |
| **30** | MSC 42667 | PS052219BL03-001 | Male | 5/21/2019 | Immature | 745 | 13-1-13 | 11-2-11 |  |
| **31** | MSC 42668 | PS052219BL03-002 | Male | 5/22/2019 | Immature | 585 | 12-1-12 | 11-2-11 |  |
| **32** | MSC 42669 | WI090919BL01-001 | Female | 9/9/2019 | Mature | 1011 | 12-1-12 | 11-2-11 | Early stage pups present - 3 total |
| **33** | MSC 42670 | WI090919BL01-002 | Female | 9/9/2019 | Mature | 1001 | 12-1-12 | 11-2-11 | Early stage pups present - 7 total |
| **34** | MSC 42671 | WI090919BL01-003 | Male | 9/9/2019 | Mature | 1000 | 13-1-13 | 11-2-11 | Sperm present |
| **35** | MSC 42672 | WI090919BL01-004 | Female | 9/9/2019 | Mature | 1002 | 12-1-12 | 11-2-11 | Early stage pups present - 7 total |
| **36** | MSC 42673 | WI090919BL01-005 | Female | 9/9/2019 | Mature | 977 | 12-?-12 | 11-2-11 | Early stage pups present - 4 total |
| **37** | MSC 42674 | WI090919BL01-006 | Female | 9/9/2019 | Mature | 1040 | 12-1-12 | 12-2-12 | Early stage pups present - 6 total |
| **38** | MSC 42675 | WI090919BL01-008 | Female | 9/9/2019 | Mature | 1043 | 12-1-12 | 11-2-12 | Early stage pups present - 5 total |
| **39** | MSC 42676 | WI090919BL01-009 | Female | 9/9/2019 | Mature | 1030 | 12-1-12 | 11-2-11 | Early stage pups present - 8 total |
| **40** | MSC 42677 | WI090919BL01-010 | Female | 9/9/2019 | Mature | 1025 | 12-1-12 | 11-2-11 | Early stage pups present - 8 total |
| **41** | MSC 42678 | WI090919BL01-011 | Female | 9/9/2019 | Mature | 966 | 12-1-12 | 11-2-11 | Early stage pups present - 6 total |
| **42** | MSC 42679 | WI090919BL01-012 | Female | 5/7/2021 | Mature | 1007 | 12-1-13 | 11-2-11 | Early stage pups present - 8 total |
| **43** | MSC 42680 | WI090919BL01-013 | Female | 9/9/2019 | Mature | 1025 | 12-1-12 | 11-2-11 | Early stage pups present - 6 total |
| **44** | MSC 42681 | WI090919BL01-014 | Female | 9/9/2019 | Mature | 997 | 12-1-12 | 11-2-11 | Early stage pups present - 4 total |
| **45** | MSC 42682 | WI091019BL03-026 | Male | 9/10/2019 | Immature | 715 | 12-?-12 | 11-2-11 |  |
| **46** | MSC 42683 | WI091619BL01-005 | Female | 9/16/2019 | Mature | 960 | 12-1-12 | 11-2-12 |  |
| **47** | MSC 42684 | AD092119BL02-021 | Male | 9/21/2019 | Immature | 735 | 12-1-12 | 11-2-11 |  |
| **48** | MSC 42685.1 | WIII0818BL02-007 | Female | 11/8/2018 | Mature | 895 | 13-1-13 | 11-2-11 | 6 pups present |
| **49** | MSC 42685.2 | WIII0818BL02-007 | Male | 11/8/2018 | Pup | 190 | N/A | N/A |  |
| **50** | MSC 42685.3 | WIII0818BL02-007 | Male | 11/8/2018 | Pup | 190 | N/A | N/A |  |
| **51** | MSC 42685.4 | WIII0818BL02-007 | Female | 11/8/2018 | Pup | 180 | N/A | N/A |  |
| **52** | MSC 42685.5 | WIII0818BL02-007 | Male | 11/8/2018 | Pup | 330 | 12-1-12 | 11-2-11 |  |
| **53** | MSC 42685.6 | WIII0818BL02-007 | Female | 11/8/2018 | Pup | 130 | N/A | N/A |  |
| **54** | MSC 42685.7 | WIII0818BL02-007 | Female | 11/8/2018 | Pup | 330 | 12-1-12 | 11-2-11 |  |
| **55** | MSC 42686 | PS051719BL01-001 | Male | 5/17/2019 | Immature | 690 | 12-?-12 | 11-2-11 |  |
| **56** | MSC 42687 | PS052219BL03-006 | Male | 5/22/2019 | Immature | 605 | 12-1-12 | 11-2-11 |  |
| **57** | MSC 43583 | WI052120BL01-005 | Female | 5/21/2020 | N/A | 615 | 12-1-12 | 10-2-10 | Healed mating scars |
| **58** | MSC 43584 | WI052120BL03-040 | Female | 5/21/2020 | N/A | 630 | 12-1-12 | 11-2-11 |  |
| **59** | MSC 43585 | WI052120BL01-007 | Female | 5/21/2020 | N/A | 635 | 12-1-12 | 11-2-11 |  |
| **60** | MSC 43586 | WI051520BL03-040 | Female | 5/15/2020 | N/A | 646 | 13-1-12 | 11-2-10 |  |
| **61** | MSC 43587 | WI051520BL03-029 | Male | 5/15/2020 | Immature | 725 | 12-1-12 | 11-2-11 |  |
| **62** | MSC 43588 | WI051520BL03-037 | Male | 5/15/2020 | transitional | 805 | 12-1-12 | 11-2-11 | Bite marks on body |
| **63** | MSC 43589 | WI051520BL03-022 | Male | 5/15/2020 | Mature | 815 | 12-1-12 | 11-2-11 |  |
| **64** | MSC 43590 | WI051520BL03-017 | Male | 5/15/2020 | Transitional | 885 | 13-?-13 | 12-2-12 |  |
| **65** | MSC 44454 | AD051421BL01-001 | Male | 5/14/2021 | Mature | 935 | 12-1-12 | 11-2-11 |  |
| **66** | MSC 44455 | Not assigned | Male | 5/13/2021 | Immature | 650 | 12-1-12 | 11-2-11 |  |
| **67** | MSC 44456 | WI050721BL01-002 | Male | 5/7/2021 | Mature | 1033 | 12-1-12 | 11-2-11 |  |
| **68** | MSC 44457 | WI042021BL03-017 | Male | 4/20/2021 | Mature | 990 | 13-1-13 | 12-2-12 |  |
| **69** | MSC 44458 | WI050721BL03-010 | Male | 5/7/2021 | Mature | 1010 | 12-1-12 | 11-2-11 |  |
| **70** | MSC 44459 | WI043021BL02-006 | Female | 4/30/2021 | N/A | 899 | 12-1-12 | 11-2-11 |  |
| **71** | MSC 44460 | Not assigned | Male | 5/13/2021 | Immature | 595 | 12-1-12 | 11-2-11 |  |
| **72** | MSC 44461.1 | Not assigned | Female | Apr-21 | Mature | 991 | 12-1-12 | 11-2-11 | 5 pups present |
| **73** | MSC 44461.2 | Not assigned | Female | Apr-21 | Pup | 310 | 13-1-13 | 11-?-11 |  |
| **74** | MSC 44461.3 | Not assigned | Male | Apr-21 | Pup | 300 | 12-1-12 | 11-2-11 |  |
| **75** | MSC 44461.4 | Not assigned | Female | Apr-21 | Pup | 310 | 12-1-12 | 11-2-11 |  |
| **76** | MSC 44461.5 | Not assigned | Male | Apr-21 | Pup | 310 | 12-1-12 | 11-1?-11 |  |
| **77** | MSC 44461.6 | Not assigned | Male | Apr-21 | Pup | 295 | 12-1-12 | 11-2-11 |  |
| **78** | MSC 44462 | Not assigned | Female | Apr-21 | N/A | 879 | 12-1-12 | 11-2-11 |  |
| **79** | MSC 44463 | Not assigned | Male | 5/13/2021 | Immature | 675 | 12-1-12 | 11-2-11 |  |
| **80** | MSC 44464 | Not assigned | Male | 5/13/2021 | Immature | 655 | 12-1-12 | 11-2-11 |  |
| **81** | MSC 44465 | Not assigned | Male | 5/13/2021 | Immature | 540 | 12-1-12 | 11-2-11 |  |
| **82** | MSC 44466 | Not assigned | Male | 5/13/2021 | Immature | 605 | 12-1-12 | 11-2-11 |  |
| **83** | MSC 44467 | Not assigned | Male | 5/13/2021 | Immature | 635 | 13-1-13 | 11-2-11 |  |
| **84** | MSC 44468 | Not assigned | Male | 5/13/2021 | Immature | 650 | 12-1-12 | 11-2-11 |  |
| **85** | MSC 44469 | Not assigned | Male | 5/13/2021 | Immature | 600 | 12-1-12 | 11-2-11 |  |
| **86** | MSC 44470.1 | Not assigned | Female | Apr-21 | Mature | 922 | 12-?-12 | 11-2-11 | 1 atretic embryo present |
| **87** | MSC 44470.2 | Not assigned | Male | Apr-21 | Pup | 250 | 12-1-12 | 11-2-11 |  |
| **88** | MSC 44471.1 | Not assigned | Female | Apr-21 | Mature | 862 | 12-1-12 | 11-2-11 | 3 pups present |
| **89** | MSC 44471.2 | Not assigned | Male | Apr-21 | Pup | 260 | 12-1-12 | 8?-2?-11 |  |
| **90** | MSC 44471.3 | Not assigned | Male | Apr-21 | Pup | 260 | 12-1-12 | 11-2-11 |  |
| **91** | MSC 44471.4 | Not assigned | Female | Apr-21 | Pup | 250 | 12-1-12 | 11-2-11 |  |
| **92** | MSC 44472.1 | Not assigned | Female | Apr-21 | Mature | 1021 | 12-1-12 | 11-2-11 | 6 pups present, close to birth size |
| **93** | MSC 44472.2 | Not assigned | Male | Apr-21 | Pup | 300 | 12-1-12 | 11-2-11 |  |
| **94** | MSC 44472.3 | Not assigned | Male | Apr-21 | Pup | 320 | 12-1-12 | 11-2-11 |  |
| **95** | MSC 44472.4 | Not assigned | Female | Apr-21 | Pup | 325 | 12-1-12 | 11-2-11 |  |
| **96** | MSC 44472.5 | Not assigned | Male | Apr-21 | Pup | 315 | 12-1-12 | 11-2-11 |  |
| **97** | MSC 44472.6 | Not assigned | Male | Apr-21 | Pup | 315 | 12-1-12 | 11-2-11 |  |
| **98** | MSC 44472.7 | Not assigned | Male | Apr-21 | Pup | 300 | 12-1-12 | 11-2-11 |  |
| **99** | MSC 44473 | Not assigned | Male | 5/13/2021 | Immature | 585 | 12-1-12 | 11-2-11 |  |
| **100** | MSC 44474 | Not assigned | Male | 5/13/2021 | Immature | 615 | 12-1-12 | 11-2-11 |  |
| **101** | MSC 44475 | Not assigned | Male | 5/13/2021 | Immature | 635 | 12-1-12 | 11-2-11 |  |
| **102** | MSC 44476 | Not assigned | Male | 5/13/2021 | Immature | 600 | 12-1-12 | 11-2-11 |  |
| **103** | MSC 44477 | Not assigned | Male | 5/13/2021 | Immature | 545 | 11-1-12 | 11-2-10 |  |
| **104** | MSC 44478 | Not assigned | Male | 5/13/2021 | Mature | 660 | 12-1-12 | 11-2-11 |  |
| **105** | MSC 44479 | W1042021BL03-016 | Male | 4/20/2021 | Mature | 870 | 12-1-12 | 11-2-11 |  |
| **106** | MSC 44480 | WI040621BL02-110 | Male | 4/6/2021 | Mature | 980 | 13-1-13 | 11-2-11 |  |
| **107** | MSC 44481.1 | Not assigned | Female | Apr-21 | Mature | 980 | 12-1-12 | 11-2-11 | 4 pups present |
| **108** | MSC 44481.2 | Not assigned | Female | Apr-21 | Pup | 320 | 12-1-12 | 11-2-11 |  |
| **109** | MSC 44481.3 | Not assigned | Male | Apr-21 | Pup | 310 | 12-1-12 | 11-2-11 |  |
| **110** | MSC 44481.4 | Not assigned | Male | Apr-21 | Pup | 320 | 12-1-12 | 11-2-11 |  |
| **111** | MSC 44481.5 | Not assigned | Female | Apr-21 | Pup | 310 | 12-1-12 | 11-2-11 |  |
| **112** | MSC 44482.1 | Not assigned | Female | Apr-21 | Mature | 940 | 12-1-12 | 11-2-11 | 6 pups present |
| **113** | MSC 44482.2 | Not assigned | Male | Apr-21 | Pup | 275 | 12-1-12 | 11-2-10 |  |
| **114** | MSC 44482.3 | Not assigned | Male | Apr-21 | Pup | 280 | 12-1-12 | 11-2-11 |  |
| **115** | MSC 44482.4 | Not assigned | Male | Apr-21 | Pup | 290 | 12-1-12 | 11-2-11 |  |
| **116** | MSC 44482.5 | Not assigned | Male | Apr-21 | Pup | 290 | 12-1-12 | 11-1-11 |  |
| **117** | MSC 44482.6 | Not assigned | Male | Apr-21 | Pup | 280 | 12-1-12 | 11-2-11 |  |
| **118** | MSC 44482.7 | Not assigned | Male | Apr-21 | Pup | 280 | 12-1-12 | 11-2-11 |  |
| **119** | MSC 46703 | Not assigned | Male | 9/6/2022 | Mature | 964 | 12-1-12 | 11-2-11 |  |
| **120** | MSC 46735 | WI100622BL02-006 | Male | 10/6/2022 | Mature | 994 | 13-1-13 | 11-2-11 |  |
| **121** | MSC 46736 | WI100722BL04-027 | Male | 10/7/2022 | Mature | 966 | 12-1-12 | 11-2-11 |  |
| **122** | MSC 46737 | WI100722BL01-012 | Male | 10/7/2022 | Mature | 984 | 12-1-12 | 11-2-11 |  |
| **123** | MSC 46738 | WI100722BL01-007 | Male | 10/7/2022 | Mature | 1001 | 13-1-12 | 11-2-11 |  |
| **124** | MSC 46739 | Not assigned | Male | 2022 | Neonate | 462 | 12-1-12 | 11-2-11 |  |
| **125** | MSC 46740 | Not assigned | Female | 2022 | Neonate | 378 | 12-1-12 | 11-2-11 |  |
| **126** | MSC 46741 | Not assigned | Female | 2022 | Neonate | 371 | 12-1-12 | 11-2-11 |  |

**Appendix 11. Specimen Data.** Acronyms: **Mec DF** = Meckel’s cartilage dental formula; **Pal DF** = Palatoquadrate dental formula; **STL** = stretched total length; **?** = indicates missing tooth/data. Sexual maturity for females based on the presence of pups. Male life stage based on extent of calcification of the myxopterygia (see Materials & Methods).

**Appendix 1.2: Comparative Specimens**

| **Number** | **Taxon** | **Maturity Stage** | **Sex** |
| --- | --- | --- | --- |
| MSC 42586 | *Carcharhinus leucas* | Unknown | Unknown |
| SC86.186.1 | *Carcharhinus obscurus* | Unknown | Unknown |
| MSC 42614 | *Carcharhinus obscurus* | Unknown | Unknown |
| MSC 42591 | *Rhizoprionodon acutus* | Unknown | Unknown |
| SC2020.53.28 | *Rhizoprionodon longorio* | Unknown | Unknown |
| SC96.77.9 | *Rhizoprionodon acutus* | Unknown | Unknown |
| MSC 46806 | *Rhizoprionodon longorio* | Unknown | Unknown |
| MSC 46804 | *Scoliodon* sp. | Unknown | Unknown |
| MSC 42605 | *Sphyrna lewini* | Unknown | Unknown |
| MSC 46805 | *Sphyrna lewini* | Juvenile | Unknown |
| SC2000.120.4 | *Sphyrna lewini* | Unknown | Unknown |
| SC2000.120.2 | *Sphyrna mokarran* | Unknown | Unknown |
| MSC 42603 | *Sphyrna tiburo* | Unknown | Unknown |
| SC96.77.3 | *Sphyrna tiburo* | Unknown | Unknown |
| MSC 42600 | *Sphyrna zygaena* | Unknown | Unknown |

**Appendix 1.2. Comparative jaw specimens examined.** Acronyms: **MSC** = McWane Science Center, 200 19^th^ Street North, Birmingham, Alabama, 35023, USA. **SC** = South Carolina State Museum, Columbia, South Carolina, 29201, USA.

**Appendix 1.3: Greatest Tooth Width – Female – Palatoquadrate**

|  | **MSC 42649** | **MSC 42645** | **MSC 43583** | **MSC 43585** | **MSC 42638** | **MSC 42685** | **MSC 44482.1** | **MSC 44481.1** | **MSC 42670** | **MSC 42676** |
| --- | --- | --- | --- | --- | --- | --- | --- | --- | --- | --- |
|  | **STL 600** | **STL 610** | **STL 615** | **STL 635** | **STL 675** | **STL 895** | **STL 940** | **STL 980** | **STL 1001** | **STL 1030** |
| **Sy** | 2.09 | 2.88 | 2.1 | 2.84 | NA | 3.12 | 3.09 | 3.53 | 3.58 | 3.41 |
| **1** | 2.83 | 2.83 | 2.61 | 2.71 | 2.63 | 4.27 | 3.9 | 4.25 | 4.47 | 4.23 |
| **2** | 2.98 | 3.2 | 3.08 | 3.75 | 3.06 | 4.97 | 4.84 | 5.23 | 4.88 | 4.46 |
| **3** | 3.39 | 3.53 | 3.58 | 4.35 | 3.42 | 5.71 | 5.36 | 5.4 | 5.67 | 5.05 |
| **4** | 4.54 | 2.88 | 4.29 | 4.43 | 4.11 | 5.9 | 6.64 | 6.71 | 7.07 | 6.23 |
| **5** | 4.07 | 4.11 | 4.62 | 4.67 | 4.59 | 5.98 | 7.29 | 7.03 | 7.15 | 6.88 |
| **6** | 3.69 | 4.32 | 4.65 | 4.35 | 4.69 | 6 | 6.76 | 7.15 | 7.32 | 6.92 |
| **7** | 4.28 | 4.44 | 4.52 | 4.24 | 4.38 | 6.02 | 6.63 | 6.79 | 6.91 | 6.63 |
| **8** | 3.87 | 4.3 | 4.31 | 4.06 | 4.43 | 5.73 | 6.56 | 6.72 | 7.04 | 7.16 |
| **9** | 2.1 | 4.29 | 3.91 | 3.72 | 4.04 | 4.56 | 6.15 | 6.38 | 6.66 | 6.84 |
| **10** | 3.23 | NA | 2.44 | 3.29 | 3.37 | 4.13 | 5.87 | 5.92 | 5.89 | 6.83 |
| **11** | 3.38 | 3.31 | NA | 3.45 | 3.38 | NA | 5.51 | 5.71 | 5.84 | 6.04 |
| **12** | NA | NA | NA | NA | NA | NA | NA | 5.1 | 5.67 | NA |

**Appendix 1.3. Greatest mesiodistal tooth width for female *Rhizoprionodon terraenovae* teeth across ontogeny on the palatoquadrate.** Numbers refer to specific tooth files that are numbered consecutively from the symphysis to the commissure. NA = no data available for this particular tooth. STL = stretched total length. Sy = symphyseal tooth.

**Appendix 1.4: Greatest Tooth Width – Female – Meckel’s Cartilage**

|  | **MSC 42649** | **MSC 42645** | **MSC 43583** | **MSC 43585** | **MSC 42638** | **MSC 42685** | **MSC 44482.1** | **MSC 44481.1** | **MSC 42670** | **MSC 42676** |
| --- | --- | --- | --- | --- | --- | --- | --- | --- | --- | --- |
|  | **STL 600** | **STL 610** | **STL 615** | **STL 635** | **STL 675** | **STL 895** | **STL 940** | **STL 980** | **STL 1001** | **STL 1030** |
| **Pa** | 2.48 | 2.33 | 2.42 | 2.29 | 2.6 | 3.5 | 3.36 | 4.01 | 4.07 | 3.81 |
| **1** | 3.46 | 3.51 | 3.66 | 3.6 | 3.7 | 4.95 | 5.23 | 4.03 | 6.07 | 5.85 |
| **2** | 3.79 | 4.05 | 4.12 | 4.19 | 4.12 | 5.34 | 5.98 | 5.36 | 6.6 | 6.22 |
| **3** | 3.95 | 4.2 | 4.27 | 4.3 | 4.16 | 5.48 | 6.48 | 6.09 | 6.84 | 7.09 |
| **4** | 4.05 | 4.36 | 4.43 | 4.43 | 4.35 | 5.91 | 6.68 | 6.24 | 6.97 | 7.2 |
| **5** | 3.47 | 4.41 | 4.28 | 4.35 | 4.35 | 6.23 | 6.88 | 6.17 | 7.03 | 6.98 |
| **6** | 3.99 | 4.16 | 4.21 | NA | 4.2 | 5.97 | 6.4 | 6.87 | 6.84 | 6.62 |
| **7** | NA | 3.92 | 3.98 | 3.93 | 4.06 | 5.81 | 6.27 | 6.61 | 6 | 6.44 |
| **8** | 3.57 | 3.83 | 3.67 | 3.71 | 3.74 | 5.74 | 6.09 | 6.32 | 6.29 | 6.14 |
| **9** | 2.12 | 3.71 | 3.42 | 3.52 | 3.48 | 5.21 | NA | 5.92 | 5.79 | 5.57 |
| **10** | NA | 3.37 | 3.16 | 3.19 | 3.11 | 4.75 | 5.2 | 5.02 | 5.45 | NA |
| **11** | NA | 3.39 | NA | NA | 2.59 | NA | 4.72 | 4.5 | 4.91 | NA |

**Appendix 1.4. Greatest mesiodistal tooth width for female *Rhizoprionodon terraenovae* teeth across ontogeny on the Meckel’s cartilage.** Numbers refer to specific tooth files that are numbered consecutively from the symphysis to the commissure. NA = no data available for this particular tooth. Pa = parasymphyseal tooth. STL = stretched total length.

**Appendix 1.5: Greatest Tooth Width – Male – Palatoquadrate**

|  | **MSC 42662** | **MSC 42663** | **MSC 44474** | **MSC 42657** | **MSC 42651** | **MSC 42656** | **MSC 44454** | **MSC 44480** | **MSC 42671** | **MSC 44456** |
| --- | --- | --- | --- | --- | --- | --- | --- | --- | --- | --- |
|  | **STL 600** | **STL 610** | **STL 615** | **STL 635** | **STL 675** | **STL 895** | **STL 935** | **STL 980** | **STL 1000** | **STL 1033** |
| **Sy** | 2.62 | 2.2 | 1.99 | 2.08 | 2.81 | 2.77 | 3.75 | 3.61 | 3.42 | 3.62 |
| **1** | 3.01 | 2.63 | 2.74 | 2.66 | 3.27 | 3.68 | 4.28 | 4.09 | 4.22 | 4.27 |
| **2** | 3.37 | 2.7 | 3.13 | 3.28 | 3.03 | 4.38 | 4.62 | 4.71 | 4.88 | 4.96 |
| **3** | 4.13 | 3.18 | 3.47 | 3.54 | 3.89 | 4.82 | 5.49 | 5.29 | 5.51 | 5.74 |
| **4** | 4.44 | 3.5 | 4.17 | 4.01 | 4.8 | 6.12 | 6.77 | 6.36 | 6.68 | 6.75 |
| **5** | 4.32 | 4.14 | 4.45 | 4.3 | 4.93 | 6.24 | 6.72 | 7.26 | 7.32 | 7 |
| **6** | 4.37 | 4.29 | 4.39 | 4.44 | NA | 6.11 | 6.95 | 7.22 | 6.5 | 6.48 |
| **7** | 4.09 | 4.29 | 4.35 | 4.36 | 4.6 | 5.76 | 6.8 | 7.05 | 6.29 | NA |
| **8** | 4.03 | 4.25 | 4.21 | 4.32 | 4.56 | 5.34 | 6.48 | 6.77 | 6.79 | 6.36 |
| **9** | 3.71 | NA | 3.86 | 4.15 | 4.25 | 5.6 | 6.25 | 6.57 | 6.44 | 6.22 |
| **10** | 3.31 | 3.58 | 3.76 | 4.21 | 3.84 | 5.42 | 5.85 | 6.15 | 5.65 | 5.69 |
| **11** | 3.35 | 3.22 | 3.7 | 3.99 | 3.54 | 4.96 | 5.46 | 5.69 | 5.2 | 5.67 |
| **12** | NA | NA | 3.31 | 3.32 | 4 | 4.77 | NA | 3.21 | 4.73 | NA |

**Appendix 1.5. Greatest mesiodistal tooth width for male *Rhizoprionodon terraenovae* teeth across ontogeny on the palatoquadrate.** Numbers refer to specific tooth files that are numbered consecutively from the symphysis to the commissure. NA = no data available for this particular tooth. STL = stretched total length. Sy = symphyseal tooth.

**Appendix 1.6: Greatest Tooth Width – Male – Meckel’s Cartilage**

|  | **MSC 42662** | **MSC 42663** | **MSC 44474** | **MSC 42657** | **MSC 42651** | **MSC 42656** | **MSC 44454** | **MSC 44480** | **MSC 42671** | **MSC 44456** |
| --- | --- | --- | --- | --- | --- | --- | --- | --- | --- | --- |
|  | **STL 600** | **STL 610** | **STL 615** | **STL 635** | **STL 675** | **STL 895** | **STL 935** | **STL 980** | **STL 1000** | **STL 1033** |
| **Pa** | 2.24 | 2.36 | 2.53 | 2.51 | 2.71 | 3.47 | 3.78 | 3.36 | 3.68 | 3.02 |
| **1** | 3.61 | 3.59 | 3.52 | 3.59 | 3.93 | 4.97 | 5.6 | 5.12 | 5.38 | 4.67 |
| **2** | 3.98 | 3.93 | 4.09 | 4.11 | 4.38 | 5.84 | 6.08 | 6.11 | 5.87 | 5.4 |
| **3** | 3.95 | 3.92 | 4.07 | 4.08 | 4.33 | 6.02 | 6.67 | 6.47 | 6.58 | 6.51 |
| **4** | 4.06 | 4.31 | 4.19 | 4.11 | 4.52 | 6.06 | 6.63 | 5.71 | 6.93 | 6.58 |
| **5** | 4.05 | 4.16 | 4.16 | 4.27 | 4.51 | 5.67 | 6.57 | 6.87 | 7.13 | 6.39 |
| **6** | 3.98 | 4.06 | 4.06 | 4.27 | 4.5 | 5.62 | 6.41 | 6.75 | 6.78 | 6.33 |
| **7** | 3.8 | 3.95 | 3.87 | 4.12 | 4.24 | 5.27 | 6.1 | 6.16 | 6.28 | 6.1 |
| **8** | 3.65 | 3.76 | 3.61 | 3.88 | 4.08 | 5.4 | 6.05 | 5.99 | 6.09 | 5.77 |
| **9** | 3.39 | 3.06 | 3.35 | 3.23 | 3.64 | 5.14 | 5.53 | 5.99 | 5.74 | 5.65 |
| **10** | 2.99 | 2.66 | 2.98 | 2.81 | 3.61 | 4.48 | 5.12 | 5.5 | 5.09 | NA |
| **11** | 2.89 | NA | NA | NA | 3.21 | 3.67 | 4.25 | NA | 5.05 | NA |

**Appendix 1.6. Greatest mesiodistal tooth width for male *Rhizoprionodon terraenovae* teeth across ontogeny on the Meckel’s cartilage.** Numbers refer to specific tooth files that are numbered consecutively from the symphysis to the commissure. NA = no data available for this particular tooth. Pa = parasymphyseal tooth. STL = stretched total length.

**Appendix 1.7: Greatest Tooth Height – Female – Palatoquadrate**

|  | **MSC 42649** | **MSC 42645** | **MSC 43583** | **MSC 43585** | **MSC 42638** | **MSC 42685** | **MSC 44482.1** | **MSC 44481.1** | **MSC 42670** | **MSC 42676** |
| --- | --- | --- | --- | --- | --- | --- | --- | --- | --- | --- |
|  | **STL 600** | **STL 610** | **STL 615** | **STL 635** | **STL 675** | **STL 895** | **STL 940** | **STL 980** | **STL 1001** | **STL 1030** |
| **Sy** | 2.34 | 2.01 | 2.06 | NA | NA | 2.88 | 2.86 | 3.38 | 3.52 | 2.99 |
| **1** | 2.57 | 2.4 | 2.45 | 1.51 | 2.56 | 3.66 | 3.98 | 3.72 | 4.28 | 4.03 |
| **2** | 2.7 | 2.39 | 2.62 | 2.62 | 2.62 | 3.95 | 4.06 | 3.73 | 4.45 | 3.98 |
| **3** | 3.06 | 2.74 | 2.84 | 2.7 | 2.95 | 3.98 | 4.43 | 4.1 | 4.75 | 4.36 |
| **4** | 2.92 | 2.38 | 2.83 | 2.6 | 3.04 | 3.91 | 4.48 | 4.4 | 4.82 | 4.61 |
| **5** | 2.61 | 2.7 | 2.75 | 2.39 | 2.81 | 2.77 | 4.42 | 4.27 | 4.67 | 4.45 |
| **6** | 2.09 | 2.7 | 2.67 | 2.1 | 2.69 | 3.62 | 3.75 | 4.2 | 4.48 | 4.26 |
| **7** | 2.22 | 2.36 | 2.38 | 1.66 | 2.49 | 3.33 | 3.45 | 4.11 | 4.15 | 4.35 |
| **8** | 1.66 | 1.87 | 1.86 | 1.62 | 2.5 | 2.84 | 3.25 | 3.65 | 3.74 | 4.1 |
| **9** | 0.78 | 2.1 | 1.89 | 1.32 | 1.81 | 1.97 | 3.05 | 3.26 | 3.23 | 3.63 |
| **10** | 1.29 | NA | 0.79 | 1.22 | 1.37 | 1.73 | 2.33 | 2.82 | 2.54 | 3.07 |
| **11** | 1.18 | 1.23 | NA | 1.14 | 1.12 | NA | 1.78 | 2.33 | 2.02 | 2.67 |
| **12** | NA | NA | NA | NA | NA | NA | NA | 1.82 | 1.8 | NA |

**Appendix 1.7. Greatest apicobasal tooth height for female *Rhizoprionodon terraenovae* teeth across ontogeny on the palatoquadrate.** Numbers refer to specific tooth files that are numbered consecutively from the symphysis to the commissure. NA = no data available for this particular tooth. STL = stretched total length. Sy = symphyseal tooth.

**Appendix 1.8: Greatest Tooth Height – Female – Meckel’s Cartilage**

|  | **MSC 42649** | **MSC 42645** | **MSC 43583** | **MSC 43585** | **MSC 42638** | **MSC 42685** | **MSC 44482.1** | **MSC 44481.1** | **MSC 42670** | **MSC 42676** |
| --- | --- | --- | --- | --- | --- | --- | --- | --- | --- | --- |
|  | **STL 600** | **STL 610** | **STL 615** | **STL 635** | **STL 675** | **STL 895** | **STL 940** | **STL 980** | **STL 1001** | **STL 1030** |
| **Pa** | 2.08 | 1.99 | 1.79 | 1.88 | 1.58 | 2.62 | 2.69 | 2.84 | 3.37 | 3.09 |
| **1** | 2.47 | 2.42 | 2.54 | 2.38 | 2.27 | 3.35 | 3.81 | 2.72 | 4.13 | 3.88 |
| **2** | 2.53 | 2.44 | 2.21 | 2.48 | 2.45 | 3.64 | 3.72 | 3.63 | 4.29 | 4.09 |
| **3** | 2.45 | 2.35 | 2.45 | 2.33 | 2.06 | 3.54 | 3.76 | NA | 4.18 | 3.72 |
| **4** | 2.33 | 2.2 | 2.17 | 2.14 | 2.27 | 3.35 | 3.76 | 3.58 | 4.04 | 3.76 |
| **5** | 2.1 | 2.08 | 2.17 | 2.02 | 1.74 | 3.2 | 3.63 | 3.64 | 3.75 | 3.47 |
| **6** | 2.06 | 1.92 | 2.01 | NA | 1.77 | 2.99 | 3.47 | 3.4 | 3.78 | 3.39 |
| **7** | 1.58 | 1.71 | 1.8 | 1.78 | 1.35 | 2.75 | 3.07 | 2.78 | 3.23 | 3.29 |
| **8** | 1.45 | 1.78 | 1.43 | 1.63 | 1.57 | 2.8 | 2.74 | 2.84 | 2.89 | 2.83 |
| **9** | 1.07 | 1.42 | 1.15 | 1.24 | 1.24 | 2.59 | NA | 2.68 | 2.13 | 2.57 |
| **10** | NA | 1.2 | 0.75 | 0.89 | 1.04 | 2.14 | 1.97 | 1.69 | 1.85 | NA |
| **11** | NA | 1.16 | NA | NA | 0.85 | NA | 1.53 | 1.38 | 1.58 | NA |

**Appendix 1.8. Greatest apicobasal tooth height for female *Rhizoprionodon terraenovae* teeth across ontogeny on the Meckel’s cartilage.** Numbers refer to specific tooth files that are numbered consecutively from the symphysis to the commissure. NA = no data available for this particular tooth. Pa = parasymphyseal tooth. STL = stretched total length.

**Appendix 1.9: Greatest Tooth Height – Male – Palatoquadrate**

|  | **MSC 42662** | **MSC 42663** | **MSC 44474** | **MSC 42657** | **MSC 42651** | **MSC 42656** | **MSC 44454** | **MSC 44480** | **MSC 42671** | **MSC 44456** |
| --- | --- | --- | --- | --- | --- | --- | --- | --- | --- | --- |
|  | **STL 600** | **STL 610** | **STL 615** | **STL 635** | **STL 675** | **STL 895** | **STL 935** | **STL 980** | **STL 1000** | **STL 1033** |
| **Sy** | NA | 2.28 | 1.94 | 2.15 | 2.39 | 3.22 | 2.87 | 2.13 | 2.66 | 3.2 |
| **1** | 2.46 | 2.62 | 2.28 | 2.32 | 2.73 | 3.74 | 3.81 | 3.52 | 3.59 | 4.09 |
| **2** | 2.5 | 2.55 | 2.37 | 2.48 | 2.93 | 3.89 | 3.99 | 3.86 | 4 | 4.12 |
| **3** | 2.77 | 2.54 | 2.64 | 2.88 | 3.11 | 4.32 | 3.68 | 4.03 | 4.23 | 4.41 |
| **4** | 2.87 | 2.69 | 2.72 | 2.83 | 3.08 | 4.38 | 4.39 | 4.24 | 4.28 | 4.5 |
| **5** | 2.76 | 2 | 2.68 | 2.56 | 3.28 | 4.33 | 4.33 | 3.87 | 4.11 | 4.14 |
| **6** | 2.64 | 2.73 | 2.59 | 2.42 | NA | 4.24 | 4.02 | 4.06 | 3.96 | 3.49 |
| **7** | 2.01 | 2.57 | 2.5 | 2.37 | 2.24 | 4.23 | 4.01 | 3.62 | 3.68 | 3.48 |
| **8** | 2.32 | 2.14 | 2.22 | 2.26 | 2.57 | 3.67 | 3.02 | 3.55 | 3.46 | 2.85 |
| **9** | 2.03 | NA | 1.9 | 1.96 | 1.74 | 3.27 | 2.99 | 3.12 | 2.83 | 2.59 |
| **10** | 1.61 | 1.52 | 1.42 | 1.7 | 1.5 | 2.8 | 2.31 | 2.66 | 2.7 | 2.13 |
| **11** | 1.4 | 1.26 | 1.47 | 1.71 | 1.5 | 2.18 | 2.08 | 2.28 | 2.05 | 1.78 |
| **12** | 1.15 | NA | 1.11 | 1.4 | 1.24 | 1.79 | NA | 1.56 | 1.51 | NA |

**Appendix 1.9. Greatest apicobasal tooth height for male *Rhizoprionodon terraenovae* teeth across ontogeny on the palatoquadrate.** Numbers refer to specific tooth files that are numbered consecutively from the symphysis to the commissure. NA = no data available for this particular tooth. STL = stretched total length. Sy = symphyseal tooth.

**Appendix 1.10: Greatest Tooth Height – Male – Meckel’s Cartilage**

|  | **MSC 42662** | **MSC 42663** | **MSC 44474** | **MSC 42657** | **MSC 42651** | **MSC 42656** | **MSC 44454** | **MSC 44480** | **MSC 42671** | **MSC 44456** |
| --- | --- | --- | --- | --- | --- | --- | --- | --- | --- | --- |
|  | **STL 600** | **STL 610** | **STL 615** | **STL 635** | **STL 675** | **STL 895** | **STL 935** | **STL 980** | **STL 1000** | **STL 1033** |
| **Pa** | 2.02 | 2.1 | 2.02 | 1.77 | 2.18 | 2.7 | 2.88 | 2.69 | 2.75 | 3.29 |
| **1** | 2.35 | 2.43 | 2.5 | 2.45 | 2.55 | 2.84 | 3.19 | 3.22 | 3.53 | 4.05 |
| **2** | 2.49 | 2.61 | 2.43 | 2.28 | 2.65 | 3.53 | 3.38 | 3.28 | 3.73 | 4.23 |
| **3** | 2.52 | 2.32 | 2.4 | 2.35 | 2.63 | 3.49 | 2.87 | 3.69 | 3.78 | 4.31 |
| **4** | 2.32 | 2.09 | 2.26 | 1.95 | 2.58 | 3.63 | 3.39 | 2.9 | 3.4 | 3.67 |
| **5** | 2.18 | 2.05 | 2.16 | 2.07 | 2.38 | 3.27 | 2.86 | 2.88 | 3.54 | NA |
| **6** | 2.08 | 1.94 | 2.03 | 1.76 | 2.16 | 3.12 | 2.58 | 2.79 | 3.4 | 3.03 |
| **7** | 1.89 | 1.78 | 1.95 | 1.75 | 2.2 | 2.67 | 3.06 | 2.74 | 3.31 | 2.57 |
| **8** | 1.49 | 1.43 | 1.67 | 1.47 | 1.84 | 2.86 | 2.23 | 2.69 | 2.97 | 2.16 |
| **9** | 1.23 | 1.03 | 1.38 | 1.14 | 1.84 | 2.37 | 2.59 | 2.66 | 2.34 | 1.81 |
| **10** | 1.1 | 0.86 | 1.09 | 0.9 | 1.51 | 2 | 1.8 | 1.88 | 2.08 | NA |
| **11** | 0.86 | NA | NA | NA | 1.34 | 1.56 | 1.21 | NA | 1.6 | NA |

**Appendix 1.10. Greatest apicobasal tooth height for male *Rhizoprionodon terraenovae* teeth across ontogeny on the Meckel’s cartilage.** Numbers refer to specific tooth files that are numbered consecutively from the symphysis to the commissure. NA = no data available for this particular tooth. Pa = parasymphyseal tooth. STL = stretched total length.

**Appendix 1.11: Height/Width Ratio – Female – Palatoquadrate**

|  | **MSC 42649** | **MSC 42645** | **MSC 43583** | **MSC 43585** | **MSC 42638** | **MSC 42685** | **MSC 44482.1** | **MSC 44481.1** | **MSC 42670** | **MSC 42676** |
| --- | --- | --- | --- | --- | --- | --- | --- | --- | --- | --- |
|  | **STL 600** | **STL 610** | **STL 615** | **STL 635** | **STL 675** | **STL 895** | **STL 940** | **STL 980** | **STL 1001** | **STL 1030** |
| **Sy** | 1.12 | 0.698 | 0.981 | NA | 0.851 | 0.923 | 0.926 | 0.958 | 0.983 | 0.877 |
| **1** | 0.908 | 0.848 | 0.939 | 0.557 | 0.835 | 0.857 | 1.02 | 0.875 | 0.957 | 0.953 |
| **2** | 0.906 | 0.747 | 0.851 | 0.699 | 0.967 | 0.794 | 0.839 | 0.713 | 0.912 | 0.892 |
| **3** | 0.902 | 0.776 | 0.793 | 0.621 | 0.799 | 0.697 | 0.826 | 0.759 | 0.838 | 0.863 |
| **4** | 0.643 | 0.826 | 0.66 | 0.587 | 0.642 | 0.663 | 0.675 | 0.656 | 0.682 | 0.74 |
| **5** | 0.641 | 0.657 | 0.595 | 0.512 | 0.665 | 0.463 | 0.606 | 0.607 | 0.653 | 0.647 |
| **6** | 0.566 | 0.625 | 0.574 | 0.483 | NA | 0.603 | 0.555 | 0.587 | 0.612 | 0.616 |
| **7** | 0.519 | 0.532 | 0.527 | 0.392 | 0.487 | 0.553 | 0.52 | 0.605 | 0.601 | 0.656 |
| **8** | 0.429 | 0.435 | 0.432 | 0.399 | 0.564 | 0.496 | 0.495 | 0.543 | 0.531 | 0.573 |
| **9** | 0.371 | 0.49 | 0.483 | 0.355 | 0.409 | 0.432 | 0.496 | 0.511 | 0.485 | 0.531 |
| **10** | 0.399 | NA | 0.324 | 0.371 | 0.391 | 0.419 | 0.397 | 0.476 | 0.431 | 0.449 |
| **11** | 0.349 | 0.372 | NA | 0.33 | 0.424 | NA | 0.323 | 0.408 | 0.346 | 0.442 |
| **12** | NA | NA | NA | NA | NA | NA | NA | 0.357 | 0.317 | NA |

**Appendix 1.11. Height/weight (H/W) ratios for female *Rhizoprionodon terraenovae* teeth across ontogeny on the palatoquadrate.** Numbers refer to specific tooth files that are numbered consecutively from the symphysis to the commissure. NA = no data available for this particular tooth. STL = stretched total length. Sy = symphyseal tooth.

**Appendix 1.12: Height/Width Ratio – Female – Meckel’s Cartilage**

|  | **MSC 42649** | **MSC 42645** | **MSC 43583** | **MSC 43585** | **MSC 42638** | **MSC 42685** | **MSC 44482.1** | **MSC 44481.1** | **MSC 42670** | **MSC 42676** |
| --- | --- | --- | --- | --- | --- | --- | --- | --- | --- | --- |
|  | **STL 600** | **STL 610** | **STL 615** | **STL 635** | **STL 675** | **STL 895** | **STL 940** | **STL 980** | **STL 1001** | **STL 1030** |
| **Pa** | 0.838 | 0.854 | 0.74 | 0.821 | 0.804 | 0.749 | 0.801 | 0.708 | 0.828 | 0.811 |
| **1** | 0.714 | 0.689 | 0.694 | 0.661 | 0.649 | 0.677 | 0.728 | 0.675 | 0.68 | 0.663 |
| **2** | 0.668 | 0.602 | 0.536 | 0.592 | 0.605 | 0.682 | 0.622 | 0.677 | 0.65 | 0.658 |
| **3** | 0.62 | 0.56 | 0.574 | 0.542 | 0.607 | 0.646 | 0.58 | NA | 0.611 | 0.525 |
| **4** | 0.575 | 0.505 | 0.49 | 0.483 | 0.571 | 0.567 | 0.563 | 0.574 | 0.58 | 0.522 |
| **5** | 0.605 | 0.472 | 0.507 | 0.464 | 0.528 | 0.514 | 0.528 | 0.59 | 0.533 | 0.497 |
| **6** | 0.516 | 0.462 | 0.477 | NA | 0.48 | 0.501 | 0.542 | 0.494 | 0.553 | 0.512 |
| **7** | NA | 0.436 | 0.452 | 0.453 | 0.519 | 0.473 | 0.49 | 0.421 | 0.538 | 0.511 |
| **8** | 0.406 | 0.465 | 0.39 | 0.439 | 0.451 | 0.488 | 0.45 | 0.449 | 0.459 | 0.461 |
| **9** | 0.505 | 0.383 | 0.336 | 0.352 | 0.505 | 0.497 | NA | 0.453 | 0.368 | 0.461 |
| **10** | NA | 0.356 | 0.237 | 0.279 | 0.418 | 0.451 | 0.379 | 0.337 | 0.339 | NA |
| **11** | NA | 0.342 | NA | NA | 0.417 | NA | 0.324 | 0.307 | 0.322 | NA |

**Appendix 1.12. Height/weight (H/W) ratios for female *Rhizoprionodon terraenovae* teeth across ontogeny on the Meckel’s cartilage.** Numbers refer to specific tooth files that are numbered consecutively from the symphysis to the commissure. NA = no data available for this particular tooth. Pa = parasymphyseal tooth. STL = stretched total length.

**Appendix 1.13: Height/Width Ratio – Male – Palatoquadrate**

|  | **MSC 42662** | **MSC 42663** | **MSC 44474** | **MSC 42657** | **MSC 42651** | **MSC 42656** | **MSC 44454** | **MSC 44480** | **MSC 42671** | **MSC 44456** |
| --- | --- | --- | --- | --- | --- | --- | --- | --- | --- | --- |
|  | **STL 600** | **STL 610** | **STL 615** | **STL 635** | **STL 675** | **STL 895** | **STL 935** | **STL 980** | **STL 1000** | **STL 1033** |
| **Sy** | 0.939 | 1.036 | 0.975 | 1.034 | NA | 1.162 | 0.765 | 0.59 | 0.778 | 0.884 |
| **1** | 0.831 | 0.996 | 0.832 | 0.872 | 0.973 | 1.016 | 0.89 | 0.861 | 0.851 | 0.958 |
| **2** | 0.822 | 0.944 | 0.757 | 0.756 | 0.856 | 0.888 | 0.864 | 0.82 | 0.82 | 0.831 |
| **3** | 0.695 | 0.799 | 0.761 | 0.814 | 0.863 | 0.896 | 0.67 | 0.762 | 0.768 | 0.768 |
| **4** | 0.622 | 0.769 | 0.652 | 0.706 | 0.74 | 0.716 | 0.648 | 0.667 | 0.641 | 0.667 |
| **5** | 0.611 | 0.483 | 0.602 | 0.595 | 0.612 | 0.694 | 0.644 | 0.533 | 0.561 | 0.591 |
| **6** | 0.46 | 0.636 | 0.59 | 0.545 | 0.574 | 0.694 | 0.578 | 0.562 | 0.609 | 0.539 |
| **7** | 0.567 | 0.599 | 0.575 | 0.544 | 0.568 | 0.734 | 0.59 | 0.513 | 0.585 | NA |
| **8** | 0.504 | 0.504 | 0.527 | 0.523 | 0.564 | 0.687 | 0.466 | 0.524 | 0.51 | 0.448 |
| **9** | 0.434 | NA | 0.492 | 0.472 | 0.448 | 0.584 | 0.478 | 0.475 | 0.439 | 0.416 |
| **10** | 0.423 | 0.425 | 0.378 | 0.404 | 0.407 | 0.517 | 0.395 | 0.433 | 0.478 | 0.374 |
| **11** | 0.343 | 0.391 | 0.397 | 0.429 | 0.331 | 0.44 | 0.381 | 0.401 | 0.394 | 0.314 |
| **12** | NA | NA | 0.335 | 0.422 | NA | 0.375 | NA | 0.486 | 0.319 | NA |

**Appendix 1.13. Height/weight (H/W) ratios for male *Rhizoprionodon terraenovae* teeth across ontogeny on the palatoquadrate.** Numbers refer to specific tooth files that are numbered consecutively from the symphysis to the commissure. NA = no data available for this particular tooth. STL = stretched total length. Sy = symphyseal tooth.

**Appendix 1.14: Height/Width Ratio – Male – Meckel’s Cartilage**

|  | **MSC 42662** | **MSC 42663** | **MSC 44474** | **MSC 42657** | **MSC 42651** | **MSC 42656** | **MSC 44454** | **MSC 44480** | **MSC 42671** | **MSC 44456** |
| --- | --- | --- | --- | --- | --- | --- | --- | --- | --- | --- |
|  | **STL 600** | **STL 610** | **STL 615** | **STL 635** | **STL 675** | **STL 895** | **STL 935** | **STL 980** | **STL 1000** | **STL 1033** |
| **Pa** | 0.902 | 0.89 | 0.798 | 0.705 | 0.608 | 0.778 | 0.762 | 0.801 | 0.747 | 1.089 |
| **1** | 0.651 | 0.677 | 0.71 | 0.682 | 0.614 | 0.571 | 0.57 | 0.629 | 0.656 | 0.867 |
| **2** | 0.626 | 0.664 | 0.594 | 0.555 | 0.595 | 0.604 | 0.556 | 0.537 | 0.635 | 0.783 |
| **3** | 0.638 | 0.591 | 0.59 | 0.576 | 0.495 | 0.58 | 0.43 | 0.57 | 0.574 | 0.662 |
| **4** | 0.571 | 0.485 | 0.539 | 0.474 | 0.522 | 0.599 | 0.511 | 0.508 | 0.491 | 0.558 |
| **5** | 0.538 | 0.493 | 0.519 | 0.485 | 0.4 | 0.577 | 0.435 | 0.419 | 0.496 | NA |
| **6** | 0.523 | 0.478 | 0.5 | 0.412 | 0.421 | 0.555 | 0.402 | 0.413 | 0.501 | 0.479 |
| **7** | 0.497 | 0.451 | 0.504 | 0.425 | 0.333 | 0.507 | 0.502 | 0.444 | 0.527 | 0.421 |
| **8** | 0.408 | 0.38 | 0.463 | 0.379 | 0.42 | 0.529 | 0.369 | 0.449 | 0.488 | 0.374 |
| **9** | 0.363 | 0.337 | 0.412 | 0.353 | 0.356 | 0.461 | 0.468 | 0.444 | 0.408 | 0.32 |
| **10** | 0.368 | 0.323 | 0.366 | 0.32 | 0.334 | 0.446 | 0.352 | 0.341 | 0.409 | NA |
| **11** | 0.298 | NA | NA | NA | 0.328 | 0.425 | 0.285 | NA | 0.317 | NA |

**Appendix 1.14. Height/weight (H/W) ratios for male *Rhizoprionodon terraenovae* teeth across ontogeny on the Meckel’s cartilage.** Numbers refer to specific tooth files that are numbered consecutively from the symphysis to the commissure. NA = no data available for this particular tooth. Pa = parasymphyseal tooth. STL = stretched total length.

**Appendix 1.15: Coronal Angle – Female – Palatoquadrate**

|  | **MSC 42649** | **MSC 42645** | **MSC 43583** | **MSC 43585** | **MSC 42638** | **MSC 42685** | **MSC 44482.1** | **MSC 44481.1** | **MSC 42670** | **MSC 42676** |
| --- | --- | --- | --- | --- | --- | --- | --- | --- | --- | --- |
|  | **STL 600** | **STL 610** | **STL 615** | **STL 635** | **STL 675** | **STL 895** | **STL 940** | **STL 980** | **STL 1001** | **STL 1030** |
| **Sy** | 53.51 | 34 | 52.84 | NA | NA | 52.63 | 48.45 | 51.16 | 53.71 | 50.77 |
| **1** | 41.1 | 41.31 | 48.12 | 29.9 | 42.79 | 38.96 | 49.1 | 46.79 | 50.53 | 47.31 |
| **2** | 36.53 | 32.35 | 40.94 | 33.65 | 37.9 | 38.82 | 37.85 | 36.12 | 41.55 | 37.68 |
| **3** | 41.42 | 33.18 | 41.08 | 28.66 | 38.71 | 35.1 | 39.36 | 34.85 | 39.54 | 37.61 |
| **4** | 28.58 | 41.89 | 33.16 | 27.1 | 35.1 | 32.28 | 35.07 | 31.65 | 32.12 | 34.59 |
| **5** | 28.09 | 30.35 | 29.33 | 24.28 | 29.3 | 29.77 | 30.98 | 28.04 | 30.92 | 31.73 |
| **6** | 26.92 | 28.05 | 28.95 | 22.29 | 28.41 | 31.56 | 27.48 | 28.88 | 30.57 | 29.37 |
| **7** | 23.67 | 25.63 | 26.69 | 22.65 | 28.07 | 28.15 | 26.94 | 28.11 | 28.47 | 32.57 |
| **8** | 20.55 | 22.23 | 25.93 | 18.86 | 27.87 | 23.95 | 22.52 | 24.78 | 26.38 | 28.35 |
| **9** | 15.89 | 20.47 | 22.52 | NA | 19.87 | 20.32 | 23.56 | 21.85 | 22.22 | 27.07 |
| **10** | 13.66 | NA | 16.72 | 17.57 | 18.54 | 21.4 | 16.03 | 20.01 | 18.66 | 21.38 |
| **11** | 14.29 | 14.65 | NA | 16.59 | 16.07 | NA | 17.67 | 17.65 | 14.24 | 20.74 |
| **12** | NA | NA | NA | NA | NA | NA | NA | 17.32 | 11.47 | NA |

**Appendix 1.15. Coronal angles for female *Rhizoprionodon terraenovae* teeth across ontogeny on the palatoquadrate.** Numbers refer to specific tooth files that are numbered consecutively from the symphysis to the commissure. NA = no data available for this particular tooth. STL = stretched total length. Sy = symphyseal tooth.

**Appendix 1.16: Coronal Angle – Female – Meckel’s Cartilage**

|  | **MSC 42649** | **MSC 42645** | **MSC 43583** | **MSC 43585** | **MSC 42638** | **MSC 42685** | **MSC 44482.1** | **MSC 44481.1** | **MSC 42670** | **MSC 42676** |
| --- | --- | --- | --- | --- | --- | --- | --- | --- | --- | --- |
|  | **STL 600** | **STL 610** | **STL 615** | **STL 635** | **STL 675** | **STL 895** | **STL 940** | **STL 980** | **STL 1001** | **STL 1030** |
| **Pa** | 53.51 | 34 | 52.84 | NA | NA | 52.63 | 48.45 | 51.16 | 53.71 | 50.77 |
| **1** | 41.1 | 41.31 | 48.12 | 29.9 | 42.79 | 38.96 | 49.1 | 46.79 | 50.53 | 47.31 |
| **2** | 36.53 | 32.35 | 40.94 | 33.65 | 37.9 | 38.82 | 37.85 | 36.12 | 41.55 | 37.68 |
| **3** | 41.42 | 33.18 | 41.08 | 28.66 | 38.71 | 35.1 | 39.36 | 34.85 | 39.54 | 37.61 |
| **4** | 28.58 | 41.89 | 33.16 | 27.1 | 35.1 | 32.28 | 35.07 | 31.65 | 32.12 | 34.59 |
| **5** | 28.09 | 30.35 | 29.33 | 24.28 | 29.3 | 29.77 | 30.98 | 28.04 | 30.92 | 31.73 |
| **6** | 26.92 | 28.05 | 28.95 | 22.29 | 28.41 | 31.56 | 27.48 | 28.88 | 30.57 | 29.37 |
| **7** | 23.67 | 25.63 | 26.69 | 22.65 | 28.07 | 28.15 | 26.94 | 28.11 | 28.47 | 32.57 |
| **8** | 20.55 | 22.23 | 25.93 | 18.86 | 27.87 | 23.95 | 22.52 | 24.78 | 26.38 | 28.35 |
| **9** | 15.89 | 20.47 | 22.52 | NA | 19.87 | 20.32 | 23.56 | 21.85 | 22.22 | 27.07 |
| **10** | 13.66 | NA | 16.72 | 17.57 | 18.54 | 21.4 | 16.03 | 20.01 | 18.66 | 21.38 |
| **11** | 14.29 | 14.65 | NA | 16.59 | 16.07 | NA | 17.67 | 17.65 | 14.24 | 20.74 |

**Appendix 1.16. Coronal angles for female *Rhizoprionodon terraenovae* teeth across ontogeny on the Meckel’s cartilage.** Numbers refer to specific tooth files that are numbered consecutively from the symphysis to the commissure. NA = no data available for this particular tooth. Pa = parasymphyseal tooth. STL = stretched total length.

**Appendix 1.17: Coronal Angle – Male – Palatoquadrate**

|  | **MSC 42662** | **MSC 42663** | **MSC 44474** | **MSC 42657** | **MSC 42651** | **MSC 42656** | **MSC 44454** | **MSC 44480** | **MSC 42671** | **MSC 44456** |
| --- | --- | --- | --- | --- | --- | --- | --- | --- | --- | --- |
|  | **STL 600** | **STL 610** | **STL 615** | **STL 635** | **STL 675** | **STL 895** | **STL 935** | **STL 980** | **STL 1000** | **STL 1033** |
| **Sy** | NA | 54.91 | 57.35 | 57.03 | 38.84 | 57.86 | 44.77 | 30.67 | 42.65 | 54.81 |
| **1** | 41.91 | 45.28 | 40.71 | 42.4 | 36.59 | 51.05 | 41.55 | 40.99 | 44.42 | 45.14 |
| **2** | 34.87 | 44.71 | 33.77 | 33.29 | 40.04 | 36.11 | 36.86 | 34.73 | 36.25 | 35.56 |
| **3** | 37.08 | 35.65 | 35.34 | 36.98 | 39.31 | 38.5 | 33.19 | 36.02 | 36.96 | 37.16 |
| **4** | 32.42 | 35.31 | 30.92 | 31.37 | 31.73 | 32.32 | 27.49 | 30.63 | 32.88 | 32.74 |
| **5** | 28.8 | 27.7 | 29.94 | 25.78 | 32.41 | 31.21 | 29.3 | 25.29 | 29.05 | 29.01 |
| **6** | 29.49 | 29.96 | 28.3 | 26.11 | NA | 31.96 | 27.89 | 27.47 | 31.6 | 23.69 |
| **7** | 24.49 | 27.23 | 28.17 | 25.35 | 28.85 | 33.25 | 28.57 | 23.8 | 30.94 | NA |
| **8** | 24.71 | 23.26 | 24.77 | 24.63 | 26.79 | 31.31 | 23.68 | 25.32 | 24.15 | 20.02 |
| **9** | 23.08 | NA | 21.01 | 21.28 | 24.36 | 26.7 | 21.8 | 23.09 | 21.74 | 18.73 |
| **10** | 18.32 | 18.34 | 16.66 | 19.72 | 23.71 | 24.46 | 17.44 | 19.27 | 22.13 | 15.38 |
| **11** | 15.78 | 14.94 | 15.48 | 19.7 | 17.94 | 19.47 | 16.69 | 18.23 | 18.12 | 15.93 |
| **12** | 15.88 | NA | 16.2 | 17.92 | 15.44 | 19.37 | NA | 24.92 | 10.32 | NA |

**Appendix 1.17. Coronal angles for male *Rhizoprionodon terraenovae* teeth across ontogeny on the palatoquadrate.** Numbers refer to specific tooth files that are numbered consecutively from the symphysis to the commissure. NA = no data available for this particular tooth. STL = stretched total length. Sy = symphyseal tooth.

**Appendix 1.18: Coronal Angle – Male – Meckel’s Cartilage**

|  | **MSC 42662** | **MSC 42663** | **MSC 44474** | **MSC 42657** | **MSC 42651** | **MSC 42656** | **MSC 44454** | **MSC 44480** | **MSC 42671** | **MSC 44456** |
| --- | --- | --- | --- | --- | --- | --- | --- | --- | --- | --- |
|  | **STL 600** | **STL 610** | **STL 615** | **STL 635** | **STL 675** | **STL 895** | **STL 935** | **STL 980** | **STL 1000** | **STL 1033** |
| **Pa** | 47.7 | 43.89 | 43.5 | 41.61 | 45.3 | 42.38 | 45.35 | 55.17 | 52.46 | 59.13 |
| **1** | 33.8 | 34.55 | 37.09 | 32.27 | 33.69 | 34.99 | 29.33 | 34 | 38.34 | 35.36 |
| **2** | 29.81 | 31.93 | 29.19 | 26.92 | 30.47 | 31.95 | 28.33 | 29.47 | 37.09 | 36.57 |
| **3** | 29.59 | 29.6 | 27.45 | 27.35 | 29.74 | 28.59 | 23.54 | 28.47 | 32.56 | 32.06 |
| **4** | 27.07 | 22.84 | 25.4 | 22.61 | 27.59 | 29.37 | 25.51 | 21.35 | 27.13 | 25.99 |
| **5** | 24.55 | 23.48 | 23.54 | 22.97 | 25.49 | 26.67 | 21.58 | 19.42 | 25.63 | NA |
| **6** | 23.28 | 21.62 | 23.22 | 19.34 | 22.29 | 25.18 | 20.5 | 17.36 | 26.23 | 21.47 |
| **7** | 22.29 | 19.9 | 22.91 | 18.07 | 22.67 | 22.43 | 23.21 | 20.22 | 27.09 | 18.75 |
| **8** | 16.32 | 15.79 | 19.99 | 14.58 | 23.12 | 23.52 | 16.27 | 19.16 | 23.55 | 15.35 |
| **9** | 11.2 | 11.54 | 16.19 | 12.73 | 21.76 | 19.54 | 20.56 | 19.91 | 17.79 | 15.55 |
| **10** | 16.32 | 10.15 | 11.57 | 13.11 | 16.78 | 17.35 | 13.44 | 13.44 | 17.37 | NA |
| **11** | 11.69 | NA | NA | NA | 15.21 | 21.07 | 11.68 | NA | 15.8 | NA |

**Appendix 1.18. Coronal angles for male *Rhizoprionodon terraenovae* teeth across ontogeny on the Meckel’s cartilage.** Numbers refer to specific tooth files that are numbered consecutively from the symphysis to the commissure. NA = no data available for this particular tooth. Pa = parasymphyseal tooth. STL = stretched total length.

**Appendix 1.19: Distal Heel Morphology – Females – Palatoquadrate**

| **Specimen Data** | | | **Palatoquadrate Tooth Position** | | | | | | | | | | | | | |
| --- | --- | --- | --- | --- | --- | --- | --- | --- | --- | --- | --- | --- | --- | --- | --- | --- |
| **MSC Number** | **Lifestage** | **STL (mm)** | **13** | **12** | **11** | **10** | **9** | **8** | **7** | **6** | **5** | **4** | **3** | **2** | **1** | **Sy** |
| MSC 44471.4 | Pup | 250 |  | RN | RN | RN | RN | RN | RN | TR | TR | TR | RN | RN | TR | TR |
| MSC 44461.2 | Pup | 310 | RN | RN | RN | RN | RN |  | RN | TR | TR | RN | RN | RN | TR | TR |
| MSC 44461.4 | Pup | 310 |  | RN | RN | RN | RN | TR | TR | RN | RN | RN | RN | RN | RN | RN |
| MSC 44481.5 | Pup | 310 |  | RN | RN | RN | RN | RN | RN | TR | TR | TR | TR | TR | TR | RN |
| MSC 44481.2 | Pup | 320 |  | RN | TR | RN | RN | TR | TR | RN | TR | RN | RN | RN | RN | RN |
| MSC 44472.4 | Pup | 325 |  | TR | TR | TR | TR | TR | TR | TR | RN | TR | RN | TR | TR | TR |
| MSC 42685.7 | Pup | 330 |  | TR | TR | TR | TR | TR | TR | TR | TR | TR | TR | TR | TR | TR |
| MSC 46740 | N/A | 371 |  | RN | TR | TR | TR | TR | TR | RN | TR | RN | RN | RN | TR | TR |
| MSC 46741 | N/A | 378 |  | RN | RN | TR | RN | RN | RN | RN | RN | RN | RN | RN | RN | TR |
| MSC 42649 | N/A | 600 |  | RN | TR | TR | BI | TR | TR | BI | BI | BI | BI | TR | TR | TR |
| MSC 42645 | N/A | 610 |  | RN | RN | RN | TR | TR | TR | TR | TR | TR | TR | TR | TR | TR |
| MSC 43583 | N/A | 615 |  | RN | RN | BI | BI | TR | BI | TR | TR | TR | BI | BI | TR | RN |
| MSC 42655 | N/A | 625 |  | TR | BI | BI | BI | BI | BI | BI | BI | BI | BI | BI | BI | BI |
| MSC 43584 | N/A | 630 |  | TR | TR | BI | BI | BI | BI | BI | TR | TR | TR | TR | RN | RN |
| MSC 43585 | N/A | 635 |  | BI | BI | RN | TR | TR | TR | RN | TR | BI | TR | TR | BI | BI |
| MSC 43586 | N/A | 646 | RN | RN | TR | TR | RN | TR | TR | BI | TR | BI | BI | TR | BI |  |
| MSC 42638 | N/A | 675 |  | RN | RN | RN | BI | TR | BI | TR | TR | TR | TR | TR | TR |  |
| MSC 44471.1 | Mature | 862 |  | RN | RN | TR | TR | RN | RN | RN | RN | RN | RN | BI | BI | RN |
| MSC 44462 | N/A | 879 |  | RN | RN | RN | RN | RN | BI | RN | RN | RN | RN | RN | RN | RN |
| MSC 42685.1 | Mature | 895 | RN | TR | RN | RN | RN | RN | RN | RN | RN | RN | RN | RN | RN | BI |
| MSC 44459 | N/A | 899 |  | RN | RN | RN | RN | RN | BI | RN | RN | RN | RN | RN | RN | BI |
| MSC 44470.1 | Mature | 922 |  | RN | BI | RN | RN | RN | RN | RN | RN | RN | RN | RN | RN |  |
| MSC 44482.1 | Mature | 940 |  | RN | RN | RN | RN | RN | RN | RN | RN | RN | RN | RN | RN | RN |
| MSC 42666 | Mature | 960 |  | BI | BI | RN | RN | RN | RN | RN | RN | RN | RN | RN | TR | BI |
| MSC 42683 | Mature | 960 | RN | RN | TR | RN | RN | RN | RN | RN | RN | RN | RN | RN | BI | RN |
| MSC 42678 | Mature | 966 |  | RN | RN | RN | RN | RN | RN | RN | RN | RN | RN | RN | RN | RN |
| MSC 42673 | Mature | 977 |  | RN | RN | RN | RN | RN | RN | RN | RN | RN | RN | TR | TR |  |
| MSC 44481.1 | Mature | 980 |  | RN | RN | RN | RN | RN | RN | RN | RN | RN | RN | RN | BI | RN |
| MSC 44461.1 | Mature | 991 |  | RN | RN | RN | RN | RN | RN | RN | RN | RN | RN | RN | RN | RN |
| MSC 42681 | Mature | 997 |  | RN | RN | BI | RN | RN | RN | BI | RN | RN | RN | RN | RN | RN |
| MSC 42670 | Mature | 1001 |  | RN | RN | RN | RN | RN | RN | RN | RN | RN | RN | RN | RN | RN |
| MSC 42672 | Mature | 1002 |  | RN | RN | RN | RN | TR | RN | RN | RN | RN | RN | RN | RN | RN |
| MSC 42679 | Mature | 1007 |  | RN | RN | RN | RN | RN | RN | RN | RN | RN | RN | RN | RN | RN |
| MSC 42669 | Mature | 1011 |  | TR | RN | RN | RN | BI | BI | BI | TR | TR | RN | RN | RN | RN |
| MSC 44472.1 | Mature | 1021 |  | RN | RN | RN | RN | BI | BI | BI | RN | BI | RN | RN | RN | BI |
| MSC 42677 | Mature | 1025 |  | BI | RN | BI | TR | BI | BI | TR | BI | RN | BI | RN | BI | RN |
| MSC 42680 | Mature | 1025 |  | RN | RN | RN | RN | RN | RN | RN | RN | RN | RN | RN | RN | RN |
| MSC 42676 | Mature | 1030 |  | RN | RN | RN | RN | BI | BI | RN | BI | RN | RN | RN | RN | RN |
| MSC 42674 | Mature | 1040 |  | TR | TR | TR | RN | RN | RN | RN | RN | TR | BI | BI | RN | RN |
| MSC 42675 | Mature | 1043 |  | TR | TR | TR | TR | BI | TR | RN | RN | RN | RN | RN | RN | RN |

**Appendix 1.19. Female dataset for the morphology of the distal heel on the palatoquadrate.** Column header acronyms: **STL** = stretched total length; **Sy** = symphyseal tooth. Descriptive acronyms: **BI** = bifurcated distal heel; **RN** = rounded distal heel; **TR** = triangular distal heel. Color codes: **Black** = no data; **Dark grey** = bifurcated distal heel; **Light grey** = triangular distal heel; **White** = rounded distal heel. All pups were *in utero*. Sexual maturity for females based on the presence of pups.

**Appendix 1.20: Distal Heel Morphology – Females – Meckel’s Cartilage**

| **Specimen Data** | | | **Meckel's Cartilage Tooth Position** | | | | | | | | | | | | | |
| --- | --- | --- | --- | --- | --- | --- | --- | --- | --- | --- | --- | --- | --- | --- | --- | --- |
| **MSC Number** | **Lifestage** | **STL (mm)** | **12** | **11** | **10** | **9** | **8** | **7** | **6** | **5** | **4** | **3** | **2** | **1** | **LPa** | **RPa** |
| MSC 44471.4 | Pup | 250 |  | RN | RN | RN | RN | TR | TR | TR | TR | TR | TR | TR | TR | TR |
| MSC 44461.2 | Pup | 310 |  | RN | RN | RN | RN | RN | RN | TR | RN | RN | TR | RN |  |  |
| MSC 44461.4 | Pup | 310 |  | RN | RN | RN | RN | RN | TR | RN | TR | RN | RN | RN | RN | RN |
| MSC 44481.5 | Pup | 310 |  | RN | RN | RN | RN | RN | TR | RN | TR | TR | TR | TR | RN | TR |
| MSC 44481.2 | Pup | 320 |  | RN | TR | RN | RN | RN | TR | RN | RN | TR | RN | RN | RN | TR |
| MSC 44472.4 | Pup | 325 |  | TR | TR | TR | TR | TR | TR | TR | TR | TR | TR | TR | TR | TR |
| MSC 42685.7 | Pup | 330 |  | TR | TR | TR | TR | TR | TR | TR | TR | TR | TR | TR | TR | TR |
| MSC 46740 | N/A | 371 |  | TR | RN | TR | TR | TR | TR | TR | RN | RN | RN | TR | TR | TR |
| MSC 46741 | N/A | 378 |  | RN | RN | RN | RN | RN | RN | RN | RN | TR | TR | TR | RN | RN |
| MSC 42649 | N/A | 600 |  | TR | BI | BI | TR | TR | TR | BI | BI | BI | BI | RN | TR | RN |
| MSC 42645 | N/A | 610 |  | RN | RN | RN | TR | TR | TR | TR | TR | TR | TR | RN | RN | RN |
| MSC 43583 | N/A | 615 |  |  | RN | RN | TR | BI | TR | TR | TR | BI | BI | BI | RN | TR |
| MSC 42655 | N/A | 625 |  | TR | TR | RN | RN | BI | BI | RN | RN | RN | BI | BI | BI | BI |
| MSC 43584 | N/A | 630 |  | TR | TR | TR | TR | TR | TR | TR | TR | TR | TR | RN | RN | RN |
| MSC 43585 | N/A | 635 |  | TR | TR | TR | RN | TR | RN | RN | RN | RN | RN | RN | TR | TR |
| MSC 43586 | N/A | 646 |  | RN | RN | BI | RN | TR | BI | TR | BI | BI | BI | BI | TR | TR |
| MSC 42638 | N/A | 675 |  | TR | TR | TR | TR | TR | TR | BI | TR | TR | TR | TR | BI | TR |
| MSC 44471.1 | Mature | 862 |  | RN | TR | TR | TR | RN | RN | RN | RN | RN | TR | TR | BI | RN |
| MSC 44462 | N/A | 879 |  | RN | RN | RN | BI | RN | RN | BI | RN | RN | RN | RN | RN | RN |
| MSC 42685.1 | Mature | 895 |  | RN | BI | RN | RN | RN | BI | RN | TR | BI | RN | RN | RN | BI |
| MSC 44459 | N/A | 899 |  | RN | RN | RN | RN | BI | BI | RN | BI | BI | RN | RN | RN | RN |
| MSC 44470.1 | Mature | 922 |  | RN | TR | TR | RN | RN | RN | RN | RN | RN | RN | RN | RN | RN |
| MSC 44482.1 | Mature | 940 |  | RN | RN | RN | RN | RN | RN | RN | RN | RN | RN | RN | RN | RN |
| MSC 42666 | Mature | 960 |  | RN | RN | BI | RN | BI | RN | RN | RN | RN | RN | RN | BI | RN |
| MSC 42683 | Mature | 960 |  | RN | RN | RN | RN | RN | RN | RN | RN | RN | RN | RN | RN | RN |
| MSC 42678 | Mature | 966 |  | RN | TR | RN | RN | RN | RN | RN | RN | RN | RN | RN | RN | RN |
| MSC 42673 | Mature | 977 |  | RN | RN | RN | RN | RN | RN | RN | RN | RN | RN | RN | RN | RN |
| MSC 44481.1 | Mature | 980 |  | TR | RN | RN | RN | RN | RN | RN | RN | RN | RN | RN | RN | RN |
| MSC 44461.1 | Mature | 991 |  | RN | RN | RN | BI | BI | BI | RN | RN | RN | RN | RN | RN | RN |
| MSC 42681 | Mature | 997 |  | RN | RN | RN | RN | RN | RN | RN | RN | RN | RN | RN | RN | BI |
| MSC 42670 | Mature | 1001 |  | RN | RN | RN | RN | RN | RN | RN | RN | RN | RN | RN | RN | RN |
| MSC 42672 | Mature | 1002 |  | RN | RN | RN | RN | RN | RN | BI | RN | RN | RN | RN | RN | RN |
| MSC 42679 | Mature | 1007 |  | RN | RN | RN | RN | RN | RN | RN | RN | RN | RN | RN | RN | RN |
| MSC 42669 | Mature | 1011 |  | RN | RN | RN | RN | RN | RN | RN | BI | TR | TR | RN | RN | RN |
| MSC 44472.1 | Mature | 1021 |  | RN | RN | RN | RN | TR | TR | TR | TR | TR | RN | RN | RN | RN |
| MSC 42677 | Mature | 1025 |  | BI | TR | TR | BI | BI | BI | BI | BI | BI | BI | BI | RN | RN |
| MSC 42680 | Mature | 1025 |  | RN | RN | RN | RN | RN | RN | RN | RN | RN | RN | RN | RN | RN |
| MSC 42676 | Mature | 1030 |  | RN | RN | RN | RN | BI | RN | RN | RN | RN | RN | RN | RN | RN |
| MSC 42674 | Mature | 1040 | TR | TR | TR | RN | TR | RN | RN | RN | BI | TR | BI | RN | BI | RN |
| MSC 42675 | Mature | 1043 |  | RN | RN | RN | RN | RN | RN | RN | BI | BI | BI | RN | RN | BI |

**Appendix 1.20. Female dataset for the morphology of the distal heel on the Meckel’s cartilage.** Column header acronyms: **LPa** = left parasymphyseal tooth; **RPa** = right parasymphyseal tooth; **STL** = stretched total length. Descriptive acronyms: **BI** = bifurcated distal heel; **RN** = rounded distal heel; **TR** = triangular distal heel. Color codes: **Black** = no data; **Dark grey** = bifurcated distal heel; **Light grey** = triangular distal heel; **White** = rounded distal heel. All pups were *in utero*. Sexual maturity for females based on the presence of pups.

**Appendix 1.21: Distal Heel Morphology – Males – Palatoquadrate**

| **Specimen Data** | | | **Palatoquadrate Tooth Position** | | | | | | | | | | | | | |
| --- | --- | --- | --- | --- | --- | --- | --- | --- | --- | --- | --- | --- | --- | --- | --- | --- |
| **MSC Number** | **Lifestage** | **STL (mm)** | **13** | **12** | **11** | **10** | **9** | **8** | **7** | **6** | **5** | **4** | **3** | **2** | **1** | **Sy** |
| MSC 44470.2 | Pup | 250 |  | RN | RN | RN | RN | TR | TR | TR | RN | RN | RN | RN | RN | RN |
| MSC 44471.2 | Pup | 260 |  | RN | RN | TR | RN | TR | TR | TR | TR | TR | RN | RN | RN | **N/A** |
| MSC 44471.3 | Pup | 260 |  | RN | RN | RN | RN | TR | TR | TR | TR | TR | TR | RN | RN | TR |
| MSC 44482.2 | Pup | 275 |  | RN | RN | RN | RN | RN | RN | RN | RN | RN | RN | RN | RN | RN |
| MSC 44482.3 | Pup | 280 |  | RN | RN | TR | TR | RN | RN | TR | TR | TR | TR | TR | RN | TR |
| MSC 44482.6 | Pup | 280 |  | RN | RN | TR | RN | TR | RN | TR | TR | TR | RN | RN | RN | TR |
| MSC 44482.7 | Pup | 280 |  | RN | RN | RN | RN | TR | TR | TR | TR | TR | RN | TR | TR | RN |
| MSC 44482.4 | Pup | 290 |  | TR | TR | TR | RN | TR | RN | RN | RN | RN | RN | RN | RN | TR |
| MSC 44482.5 | Pup | 290 |  | RN | RN | TR | TR | TR | RN | RN | TR | TR | TR | TR | RN | RN |
| MSC 44461.6 | Pup | 295 |  | RN | RN | RN | RN | RN | RN | RN | RN | RN | RN | RN | RN | RN |
| MSC 44461.3 | Pup | 300 |  | RN | RN | RN | RN | RN | RN | RN | RN | RN | RN | RN | RN | RN |
| MSC 44472.2 | Pup | 300 |  | RN | RN | RN | RN | RN | RN | TR | TR | TR | TR | RN | TR | TR |
| MSC 44472.7 | Pup | 300 |  | RN | TR | TR | RN | TR | RN | RN | RN | RN | RN | TR | RN | TR |
| MSC 44461.5 | Pup | 310 |  | RN | RN | TR | RN | RN | TR | TR | TR | TR | RN | RN | RN | RN |
| MSC 44481.3 | Pup | 310 |  | RN | RN | RN | TR | RN | TR | TR | RN | RN | RN | RN | RN | RN |
| MSC 44472.5 | Pup | 315 |  | RN | RN | TR | TR | TR | TR | TR | TR | RN | RN | RN | RN | RN |
| MSC 44472.6 | Pup | 315 |  | RN | TR | TR | TR | TR | TR | TR | RN | TR | TR | RN | TR | TR |
| MSC 44472.3 | Pup | 320 |  | RN | TR | TR | TR | TR | TR | TR | TR | TR | TR | TR | TR | TR |
| MSC 44481.4 | Pup | 320 |  | RN | RN | RN | TR | TR | TR | TR | TR | RN | RN | RN | RN | RN |
| MSC 42685.5 | Pup | 330 |  | RN | RN | TR | RN | TR | TR | TR | TR | RN | TR | RN | TR | RN |
| MSC 46739 | Immature | 462 |  | RN | RN | RN | RN | BI | BI | TR | RN | RN | TR | RN | TR | TR |
| MSC 42652 | Immature | 540 |  | BI | BI | BI | BI | TR | BI | BI | TR | BI | BI | TR | BI | TR |
| MSC 44465 | Immature | 540 |  | BI | BI | RN | RN | TR | TR | RN | RN | TR | BI | TR | BI | BI |
| MSC 44477 | Immature | 545 |  |  | RN | RN | BI | RN | TR | TR | TR | BI | TR | RN | RN | RN |
| MSC 42643 | Immature | 565 |  | RN | RN | RN | RN | RN | RN | RN | RN | RN | RN | RN | BI | RN |
| MSC 42668 | Immature | 585 |  | BI | RN | RN | TR | TR | BI | BI | TR | TR | TR | TR | TR | TR |
| MSC 44473 | Immature | 585 |  | RN | RN | RN | TR | BI | BI | TR | BI | BI | BI | BI | BI |  |
| MSC 42661 | Immature | 590 | TR | TR | TR | TR | TR | TR | TR | TR | TR | TR | BI | RN | RN | BI |
| MSC 42665 | Immature | 590 |  | RN | TR | RN | TR | TR | TR | BI | BI | BI | BI | TR | BI | BI |
| MSC 44460 | Immature | 595 |  | RN | RN | RN | TR | TR | TR | RN | TR | RN | TR | RN | BI | RN |
| MSC 44469 | Immature | 600 |  | TR | TR | TR | BI | BI | BI | TR | BI | BI | BI | TR | BI | TR |
| MSC 44476 | Immature | 600 |  | BI | BI | RN | RN | BI | RN | BI | BI | BI | BI | BI | RN | BI |
| MSC 42640 | Immature | 605 |  | RN | RN | TR | RN | RN | BI | TR | BI | BI | BI | RN | RN | RN |
| MSC 42662 | Immature | 605 |  | TR | TR | TR | TR | BI | BI | TR | TR | TR | TR | TR | TR | TR |
| MSC 42687 | Immature | 605 |  | TR | TR | TR | BI | BI | TR | BI | BI | BI | TR | TR | BI | TR |
| MSC 44466 | Immature | 605 |  | RN | RN | RN | RN | RN | RN | RN | RN | RN | RN | RN | RN | RN |
| MSC 42663 | Immature | 610 |  | RN | RN | TR | TR | TR | TR | TR | TR | TR | TR | RN | RN | TR |
| MSC 44474 | Immature | 615 |  | RN | RN | RN | RN | RN | RN | TR | BI | BI | TR | TR | RN | BI |
| MSC 42653 | Immature | 620 |  | BI | TR | BI | RN | BI | BI | BI | TR | BI | BI | BI | RN | TR |
| MSC 42641 | Immature | 635 |  | RN | RN | TR | RN | TR | TR | BI | TR | BI | TR | TR | TR | RN |
| MSC 42657 | Immature | 635 |  | TR | RN | TR | TR | TR | TR | TR | TR | TR | TR | TR | TR | TR |
| MSC 44467 | Immature | 635 | RN | RN | BI | RN | RN | BI | BI | BI | BI | BI | RN | RN | RN | RN |
| MSC 44475 | Immature | 635 |  | TR | RN | RN | RN | RN | RN | RN | RN | RN | RN | RN | RN | RN |
| MSC 42664 | Immature | 640 |  | RN | RN | RN | BI | TR | BI | BI | BI | BI | BI | RN | TR |  |
| MSC 42650 | Immature | 645 |  | RN | RN | RN | RN | RN |  | TR | RN | RN | RN | RN | RN | TR |
| MSC 42639 | Immature | 650 |  | RN | RN | BI | RN | TR | BI | RN | BI | BI | BI | RN | RN |  |
| MSC 42654 | Immature | 650 |  | RN | RN | RN | RN | RN | RN | RN | RN | RN | BI | RN | RN | RN |
| MSC 42659 | Immature | 650 |  | RN | RN | RN | RN | BI | BI | RN | RN | BI | BI | BI | BI |  |
| MSC 44455 | Immature | 650 |  | RN | TR | RN | TR | TR | TR | TR | RN | TR | TR | TR | RN | RN |
| MSC 44468 | Immature | 650 |  | TR | RN | RN | RN | RN | RN | RN | RN | RN | RN | RN | RN | RN |
| MSC 44464 | Immature | 655 |  | TR | BI | TR | BI | BI | BI | BI | BI | BI | BI | BI | TR | TR |
| MSC 44478 | Mature | 660 |  | TR | TR | TR | TR | TR | BI | TR | BI | TR | TR | BI | BI | TR |
| MSC 42647 | Immature | 665 |  | BI | BI | RN | BI | BI | TR | BI | BI | BI | TR | RN | RN | BI |
| MSC 42651 | Immature | 675 |  | BI | RN | RN | RN | BI | BI | BI | BI | BI | BI | BI | BI | BI |
| MSC 44463 | Immature | 675 |  | RN | RN | RN | TR | TR | RN | TR | TR | RN | BI | RN | RN | BI |
| MSC 42648 | Immature | 680 |  | RN | RN | RN | RN | TR | BI | BI | BI | TR | BI | RN | RN | RN |
| MSC 42686 | Immature | 690 |  | RN | RN | RN | RN | RN | RN | RN | BI | BI | RN | RN | RN |  |
| MSC 42682 | Immature | 715 |  | RN | RN | TR | RN | BI | TR | RN | TR | RN | TR | TR | RN |  |
| MSC 43587 | Immature | 725 |  | RN | RN | RN | RN | RN | RN | BI | BI | RN | BI | RN | RN | RN |
| MSC 42660 | Immature | 735 |  | RN | RN | RN | RN | RN | RN | RN | RN | RN | RN | RN | RN | RN |
| MSC 42684 | Immature | 735 |  | RN | RN | BI | RN | RN | RN | RN | TR | BI | TR | RN | BI | BI |
| MSC 42667 | Immature | 745 | RN | TR | RN | TR | TR | BI | TR | TR | TR | BI | BI | RN | RN | RN |
| MSC 42637 | Immature | 750 |  | TR | TR | TR | TR | TR | TR | BI | TR | BI | BI | BI | BI | TR |
| MSC 42646 | Immature | 775 |  | RN | RN | RN | RN | RN | RN | RN | RN | RN | RN | RN | RN |  |
| MSC 42658 | Transitional | 790 |  | RN | RN | RN | RN | RN | RN | RN | RN | RN | RN | RN | RN | RN |
| MSC 43588 | Transitional | 805 |  | BI | TR | RN | RN | RN | BI | TR | TR | TR | BI | BI | BI | BI |
| MSC 43589 | Mature | 815 |  | RN | RN | RN | RN | RN | RN | RN | RN | RN | RN | RN | RN | BI |
| MSC 44479 | Mature | 870 |  | TR | RN | RN | RN | RN | RN | RN | RN | RN | RN | RN | RN | RN |
| MSC 43590 | Transitional | 885 | BI | BI | BI | RN | RN | BI | RN | BI | RN | RN | RN | RN | RN |  |
| MSC 42656 | Mature | 895 |  | RN | RN | RN | RN | RN | RN | RN | RN | RN | RN | RN | RN | RN |
| MSC 44454 | Mature | 935 |  | TR | RN | RN | RN | RN | RN | RN | BI | RN | RN | BI | BI | RN |
| MSC 42644 | Mature | 955 |  | RN | RN | RN | RN | RN | RN | RN | RN | RN | RN | RN | RN | RN |
| MSC 46703 | Mature | 964 |  | RN | RN | RN | RN | RN | RN | RN | RN | RN | RN | RN | RN | RN |
| MSC 46736 | Mature | 966 |  | RN | RN | RN | TR | RN | RN | RN | RN | RN | RN | RN | RN | RN |
| MSC 44480 | Mature | 980 | RN | RN | RN | RN | RN | RN | RN | RN | RN | RN | RN | RN | RN | RN |
| MSC 46737 | Mature | 984 |  | RN | BI | RN | RN | RN | RN | RN | RN | RN | RN | RN | RN | RN |
| MSC 44457 | Mature | 990 | RN | RN | RN | RN | RN | RN | RN | BI | TR | TR | RN | RN | RN | RN |
| MSC 46735 | Mature | 994 | RN | RN | TR | RN | TR | BI | TR | TR | TR | TR | BI | BI | TR | RN |
| MSC 42671 | Mature | 1000 | RN | RN | RN | RN | RN | RN | RN | RN | RN | RN | RN | RN | RN | RN |
| MSC 46739 | Mature | 1001 | RN | RN | RN | RN | TR | RN | BI | RN | RN | BI | BI | RN | TR | TR |
| MSC 44458 | Mature | 1010 |  | RN | RN | RN | RN | RN | RN | RN | RN | RN | RN | RN | RN | RN |
| MSC 44456 | Mature | 1033 |  | RN | RN | RN | RN | RN | RN | RN | RN | RN | RN | RN | RN | RN |

**Appendix 1.21. Male dataset for the morphology of the distal heel on the palatoquadrate.** Column header acronyms: **STL** = stretched total length; **Sy** = symphyseal tooth. Descriptive acronyms: **BI** = bifurcated distal heel; **RN** = rounded distal heel; **TR** = triangular distal heel. Color codes: **Black** = no data; **Dark grey** = bifurcated distal heel; **Light grey** = triangular distal heel; **White** = rounded distal heel. Life stage for males based on extent of calcification of the myxopterygia (see Material & Methods).

**Appendix 1.22: Distal Heel Morphology – Males – Meckel’s Cartilage**

| **Specimen Data** | | | **Meckel's Cartilage Tooth Position** | | | | | | | | | | | | | |
| --- | --- | --- | --- | --- | --- | --- | --- | --- | --- | --- | --- | --- | --- | --- | --- | --- |
| **MSC Number** | **Lifestage** | **STL (mm)** | **12** | **11** | **10** | **9** | **8** | **7** | **6** | **5** | **4** | **3** | **2** | **1** | **LPa** | **RPa** |
| MSC 44470.2 | Pup | 250 |  | RN | RN | RN | RN | RN | RN | RN | RN | RN | TR | TR | TR | TR |
| MSC 44471.2 | Pup | 260 |  | RN | RN | TR | RN | RN | TR | TR | RN | RN | TR | TR | TR | TR |
| MSC 44471.3 | Pup | 260 |  | RN | RN | RN | RN | TR | TR | TR | TR | RN | TR | RN | RN | TR |
| MSC 44482.2 | Pup | 275 |  | RN | RN | RN | TR | TR | TR | RN | TR | RN | TR | TR | RN | TR |
| MSC 44482.3 | Pup | 280 |  | RN | RN | RN | RN | RN | TR | RN | RN | TR | RN | RN | TR | TR |
| MSC 44482.6 | Pup | 280 |  | RN | RN | RN | RN | TR | TR | RN | TR | RN | RN | RN | RN | TR |
| MSC 44482.7 | Pup | 280 |  | RN | RN | RN | TR | RN | RN | RN | RN | RN | TR | RN | TR | TR |
| MSC 44482.4 | Pup | 290 |  | RN | RN | RN | RN | RN | RN | TR | TR | RN | RN | RN | RN | RN |
| MSC 44482.5 | Pup | 290 |  | RN | RN | RN | RN | TR | TR | RN | RN | RN | RN | TR | RN | RN |
| MSC 44461.6 | Pup | 295 |  | RN | RN | RN | RN | RN | RN | RN | RN | TR | RN | TR | RN | RN |
| MSC 44461.3 | Pup | 300 |  | RN | TR | RN | RN | RN | RN | RN | RN | TR | TR | RN | RN | RN |
| MSC 44472.2 | Pup | 300 |  | RN | RN | RN | TR | TR | RN | TR | TR | RN | TR | RN | RN | RN |
| MSC 44472.7 | Pup | 300 |  | RN | RN | RN | TR | TR | TR | RN | RN | RN | RN | TR | RN | RN |
| MSC 44461.5 | Pup | 310 |  | RN | RN | TR | RN | RN | RN | RN | TR | RN | RN | TR | RN | RN |
| MSC 44481.3 | Pup | 310 |  | RN | RN | TR | TR | RN | RN | RN | RN | RN | RN | RN | RN | RN |
| MSC 44472.5 | Pup | 315 |  | RN | RN | RN | TR | TR | TR | RN | TR | RN | RN | RN | TR | TR |
| MSC 44472.6 | Pup | 315 |  | RN | TR | TR | TR | TR | TR | TR | TR | RN | RN | RN | TR | TR |
| MSC 44472.3 | Pup | 320 |  | TR | TR | RN | TR | TR | TR | TR | TR | TR | TR | TR | TR | TR |
| MSC 44481.4 | Pup | 320 |  | RN | RN | RN | RN | RN | RN | RN | RN | RN | RN | RN |  | RN |
| MSC 42685.5 | Pup | 330 |  | RN | TR | RN | TR | RN | RN | RN | TR | RN | TR | TR | TR | TR |
| MSC 46739 | Immature | 462 |  | RN | RN | RN | RN | RN | TR | TR | TR | TR | TR | RN | RN | RN |
| MSC 42652 | Immature | 540 |  | TR | TR | TR | TR | TR | TR | TR | TR | TR | RN | BI | TR | BI |
| MSC 44465 | Immature | 540 |  | TR | TR | TR | RN | RN | RN | RN | RN | TR | BI | RN | TR | TR |
| MSC 44477 | Immature | 545 |  | RN | RN | RN | BI | BI | RN | BI | TR | BI | TR | RN | TR |  |
| MSC 42643 | Immature | 565 |  | TR | TR | RN | RN | RN | TR | TR | RN | RN | TR | RN | RN | RN |
| MSC 42668 | Immature | 585 |  | TR | TR | RN | TR | BI | BI | RN | RN | BI | BI | BI | BI | BI |
| MSC 44473 | Immature | 585 |  | RN | TR | RN | BI | TR | BI | BI | BI | TR | BI | RN | TR | TR |
| MSC 42661 | Immature | 590 | RN | RN | RN | RN | RN | RN | TR | RN | BI | BI | RN | BI | RN |  |
| MSC 42665 | Immature | 590 |  | TR | TR | TR | TR | RN | RN | TR | BI | TR | TR | RN | BI | BI |
| MSC 44460 | Immature | 595 |  | RN | RN | BI | TR | BI | TR | BI | TR | TR | BI | TR | BI | BI |
| MSC 44469 | Immature | 600 |  | TR | TR | TR | BI | BI | TR | BI | BI | TR | TR | BI | TR | BI |
| MSC 44476 | Immature | 600 |  | TR | RN | RN | RN | RN | BI | BI | RN | BI | BI | BI | BI | BI |
| MSC 42640 | Immature | 605 |  | TR | TR | RN | TR | BI | RN | BI | BI | RN | BI | RN | RN | RN |
| MSC 42662 | Immature | 605 |  | TR | TR | TR | TR | TR | BI | TR | BI | TR | TR | RN | BI | BI |
| MSC 42687 | Immature | 605 |  | RN | BI | RN | TR | TR | TR | BI | BI | TR | TR | BI | TR | TR |
| MSC 44466 | Immature | 605 |  | RN | RN | RN | RN | RN | RN | RN | RN | RN | RN | RN | BI | BI |
| MSC 42663 | Immature | 610 |  | RN | RN | RN | TR | TR | RN | TR | TR | RN | TR | TR | TR | TR |
| MSC 44474 | Immature | 615 |  | RN | RN | RN | BI | RN | RN | BI | RN | BI | TR | BI | TR | TR |
| MSC 42653 | Immature | 620 |  | TR | TR | TR | TR | TR | TR | BI | BI | BI | BI | BI | TR | TR |
| MSC 42641 | Immature | 635 |  | RN | TR | TR | BI | BI | TR | BI | BI | BI | TR | TR | BI | BI |
| MSC 42657 | Immature | 635 |  | TR | RN | RN | RN | RN | RN | RN | RN | TR | TR | TR | RN | RN |
| MSC 44467 | Immature | 635 |  | BI | BI | RN | BI | BI | BI | BI | RN | RN | BI | RN | BI | BI |
| MSC 44475 | Immature | 635 |  | TR | RN | RN | RN | RN | RN | RN | RN | RN | RN | BI | RN | RN |
| MSC 42664 | Immature | 640 |  | TR | TR | TR | BI | BI | TR | TR | BI | TR | TR | TR | TR |  |
| MSC 42650 | Immature | 645 |  | RN | RN | RN | RN | RN | RN | RN | RN | RN | RN | BI | BI | BI |
| MSC 42639 | Immature | 650 | TR | TR | TR | RN | RN | RN | BI | BI | BI | BI | RN | RN | RN |  |
| MSC 42654 | Immature | 650 |  | RN | RN | RN | RN | RN | RN | TR | RN | TR | TR | TR | RN |  |
| MSC 42659 | Immature | 650 |  | RN | RN | RN | BI | RN | RN | RN | RN | RN | RN | RN |  | TR |
| MSC 44455 | Immature | 650 |  | RN | TR | RN | RN | RN | RN | RN | RN | RN | RN | RN | RN | RN |
| MSC 44468 | Immature | 650 |  | TR | BI | RN | RN | RN | RN | RN | BI | RN | BI | RN | BI | BI |
| MSC 44464 | Immature | 655 |  | TR | TR | TR | BI | TR | TR | BI | BI | TR | TR | TR | TR | TR |
| MSC 44478 | Mature | 660 |  | TR | TR | TR | TR | TR | BI | BI | TR | TR | BI | TR | TR | TR |
| MSC 42647 | Immature | 665 |  | BI | TR | BI | BI | TR | TR | BI | BI | TR | BI | BI | BI | BI |
| MSC 42651 | Immature | 675 |  | RN | BI | BI | RN | BI | RN | RN | BI | BI | RN | RN | BI | BI |
| MSC 44463 | Immature | 675 |  | TR | RN | RN | RN | RN | BI | TR | BI | BI | RN | BI | RN |  |
| MSC 42648 | Immature | 680 |  | BI | BI | BI | RN | RN | RN | RN | RN | RN | RN | RN | BI | BI |
| MSC 42686 | Immature | 690 |  | RN | RN | RN | RN | RN | RN | BI | RN | RN | BI | BI | RN | RN |
| MSC 42682 | Immature | 715 |  | RN | RN | RN | RN | RN | RN | RN | RN | TR | RN | RN | RN | RN |
| MSC 43587 | Immature | 725 |  | RN | RN | RN | RN | BI | BI | RN | RN | RN | RN | RN | RN | BI |
| MSC 42660 | Immature | 735 |  | RN | RN | RN | RN | RN | RN | BI | RN | RN | RN | RN | RN | RN |
| MSC 42684 | Immature | 735 |  | TR | RN | RN | BI | RN | RN | BI | BI | TR | RN | TR | RN | RN |
| MSC 42667 | Immature | 745 |  | TR | TR | TR | RN | TR | RN | BI | RN | TR | RN | BI | BI | BI |
| MSC 42637 | Immature | 750 |  | TR | TR | TR | TR | TR | TR | TR | TR | BI | TR | BI | BI | BI |
| MSC 42646 | Immature | 775 |  | RN | RN | RN | RN | RN | RN | RN | RN | RN | TR | RN | BI | BI |
| MSC 42658 | Transitional | 790 |  | BI | BI | RN | RN | RN | RN | RN | RN | RN | RN | RN | BI | RN |
| MSC 43588 | Transitional | 805 |  | RN | RN | RN | RN | BI | RN | BI | BI | BI | BI | TR | BI | BI |
| MSC 43589 | Mature | 815 |  | TR | TR | RN | RN | RN | RN | RN | RN | RN | RN | RN | BI | BI |
| MSC 44479 | Mature | 870 |  | RN | RN | RN | RN | RN | RN | RN | RN | RN | RN | RN | BI | BI |
| MSC 43590 | Transitional | 885 | TR | TR | BI | BI | BI | BI | BI | BI | BI | BI | BI | TR | BI | BI |
| MSC 42656 | Mature | 895 |  | RN | RN | RN | RN | RN | RN | RN | RN | RN | RN | RN | RN | RN |
| MSC 44454 | Mature | 935 |  | RN | RN | RN | TR | RN | RN | RN | RN | RN | RN | BI | RN | RN |
| MSC 42644 | Mature | 955 |  | RN | TR | TR | TR | RN | TR | RN | RN | RN | RN | BI | BI | BI |
| MSC 46703 | Mature | 964 |  | RN | RN | RN | RN | RN | RN | RN | RN | RN | RN | TR | TR | RN |
| MSC 46736 | Mature | 966 |  | TR | RN | TR | TR | TR | TR | TR | BI | RN | RN | RN | RN | RN |
| MSC 44480 | Mature | 980 |  | RN | RN | RN | RN | RN | RN | RN | RN | RN | RN | RN | RN | RN |
| MSC 46737 | Mature | 984 |  | TR | TR | TR | RN | RN | RN | RN | RN | RN | RN | RN | RN | RN |
| MSC 44457 | Mature | 990 | RN | RN | RN | RN | RN | RN | RN | RN | RN | RN | RN | RN | RN | RN |
| MSC 46735 | Mature | 994 |  | RN | RN | TR | RN | RN | TR | BI | BI | TR | TR | RN | RN | RN |
| MSC 42671 | Mature | 1000 |  | RN | RN | RN | RN | RN | RN | RN | RN | RN | RN | RN | RN | RN |
| MSC 46739 | Mature | 1001 |  | RN | RN | RN | TR | BI | BI | BI | RN | RN | RN | RN | RN | RN |
| MSC 44458 | Mature | 1010 |  | RN | RN | RN | RN | RN | RN | RN | RN | RN | RN | RN | RN | RN |
| MSC 44456 | Mature | 1033 |  | RN | RN | RN | RN | RN | RN | RN | RN | RN | RN | RN | RN | RN |

**Appendix 1.22. Male datasets for the morphology of the distal heel on the Meckel’s cartilage.** Column header acronyms: **LPa** = left parasymphyseal tooth; **RPa** = right parasymphyseal tooth; **STL** = stretched total length. Descriptive acronyms: **BI** = bifurcated distal heel; **RN** = rounded distal heel; **TR** = triangular distal heel. Color codes: **Black** = no data; **Dark grey** = bifurcated distal heel; **Light grey** = triangular distal heel; **White** = rounded distal heel. All pups were *in utero*. Life stage for males based on extent of calcification of the myxopterygia (see Materials & Methods).

**Appendix 1.23: Distal Heel Cutting Edge – Females – Palatoquadrate**

| **Specimen Data** | | | **Palatoquadrate Tooth Position** | | | | | | | | | | | | | |
| --- | --- | --- | --- | --- | --- | --- | --- | --- | --- | --- | --- | --- | --- | --- | --- | --- |
| **MSC Number** | **Lifestage** | **STL (mm)** | **13** | **12** | **11** | **10** | **9** | **8** | **7** | **6** | **5** | **4** | **3** | **2** | **1** | **Sy** |
| MSC 44471.4 | Pup | 250 |  | SM | SM | IR | IR | IR | IR | IR | IR | IR | IR | IR | IR | IR |
| MSC 44461.2 | Pup | 310 | SM | SM | SM | IR | IR |  | IR | IR | IR | IR | IR | SM | IR | IR |
| MSC 44461.4 | Pup | 310 |  | SM | SM | IR | IR | IR | IR | IR | IR | IR | IR | IR | IR | IR |
| MSC 44481.5 | Pup | 310 |  | SM | SM | IR | IR | IR | IR | IR | IR | IR | IR | IR | IR | IR |
| MSC 44481.2 | Pup | 320 |  | SM | IR | IR | IR | IR | IR | IR | IR | IR | IR | IR | IR | IR |
| MSC 44472.4 | Pup | 325 |  | SM | SM | IR | IR | IR | IR | IR | IR | IR | IR | IR | IR | IR |
| MSC 42685.7 | Pup | 330 |  | SM | SM | IR | IR | IR | IR | IR | IR | IR | IR | IR | IR | IR |
| MSC 46740 | N/A | 371 |  | IR | IR | IR | IR | IR | IR | IR | IR | IR | IR | IR | IR | SM |
| MSC 46741 | N/A | 378 |  | IR | IR | IR | IR | IR | IR | IR | IR | IR | IR | IR | IR | IR |
| MSC 42649 | N/A | 600 |  | IR | IR | IR | IR | IR | IR | IR | IR | IR | IR | IR | IR | IR |
| MSC 42645 | N/A | 610 |  | IR | IR | IR | IR | IR | IR | IR | IR | IR | IR | IR | IR | IR |
| MSC 43583 | N/A | 615 |  | IR | IR | IR | IR | IR | IR | IR | IR | IR | IR | IR | IR | IR |
| MSC 42655 | N/A | 625 |  | IR | IR | IR | IR | IR | IR | IR | IR | IR | IR | IR | IR | IR |
| MSC 43584 | N/A | 630 |  | IR | IR | IR | IR | IR | IR | IR | IR | IR | IR | IR | IR | IR |
| MSC 43585 | N/A | 635 |  | IR | IR | IR | IR | IR | IR | IR | IR | IR | IR | IR | IR | IR |
| MSC 43586 | N/A | 646 | IR | IR | IR | IR | IR | IR | IR | IR | IR | IR | IR | IR | IR |  |
| MSC 42638 | N/A | 675 |  | IR | IR | IR | IR | IR | IR | IR | IR | IR | IR | IR | IR |  |
| MSC 44471.1 | Mature | 862 |  | IR | IR | IR | IR | IR | IR | IR | IR | IR | IR | IR | IR | IR |
| MSC 44462 | N/A | 879 |  | SE | IR | IR | IR | IR | IR | IR | IR | IR | IR | IR | IR | IR |
| MSC 42685.1 | Mature | 895 | IR | IR | IR | IR | IR | IR | IR | IR | IR | IR | IR | IR | IR | IR |
| MSC 44459 | N/A | 899 |  | IR | IR | SE | IR | IR | IR | IR | IR | IR | SE | IR | SE | IR |
| MSC 44470.1 | Mature | 922 |  | IR | IR | IR | IR | IR | IR | IR | IR | IR | IR | IR | IR |  |
| MSC 44482.1 | Mature | 940 |  | IR | IR | SE | IR | IR | IR | IR | IR | IR | IR | IR | IR | SE |
| MSC 42666 | Mature | 960 |  | IR | IR | IR | IR | IR | IR | IR | IR | IR | IR | IR | IR | IR |
| MSC 42683 | Mature | 960 | IR | IR | IR | IR | IR | IR | IR | IR | IR | IR | IR | IR | IR | IR |
| MSC 42678 | Mature | 966 |  | SE | SE | IR | IR | IR | IR | IR | IR | IR | IR | IR | SE | SE |
| MSC 42673 | Mature | 977 |  | IR | IR | IR | IR | IR | IR | IR | IR | IR | IR | IR | IR |  |
| MSC 44481.1 | Mature | 980 |  | IR | IR | IR | IR | IR | SE | IR | IR | IR | IR | IR | IR | IR |
| MSC 44461.1 | Mature | 991 |  | SE | IR | IR | SE | IR | SE | IR | SE | SE | IR | IR | IR | IR |
| MSC 42681 | Mature | 997 |  | SE | SE | IR | IR | IR | IR | IR | IR | IR | IR | SE | IR | IR |
| MSC 42670 | Mature | 1001 |  | SE | SE | SE | SE | IR | SE | IR | IR | SE | SE | IR | SE | SE |
| MSC 42672 | Mature | 1002 |  | IR | IR | IR | IR | IR | IR | IR | IR | IR | IR | IR | IR | IR |
| MSC 42679 | Mature | 1007 |  | SE | SE | SE | IR | IR | SE | IR | SE | IR | SE | IR | IR | IR |
| MSC 42669 | Mature | 1011 |  | IR | IR | IR | IR | IR | IR | IR | IR | IR | IR | SE | IR | SE |
| MSC 44472.1 | Mature | 1021 |  | IR | IR | IR | IR | IR | IR | IR | IR | IR | IR | IR | IR | IR |
| MSC 42677 | Mature | 1025 |  | IR | IR | IR | IR | IR | IR | IR | IR | IR | IR | IR | IR | IR |
| MSC 42680 | Mature | 1025 |  | IR | IR | IR | IR | IR | SE | IR | IR | SE | IR | IR | IR | IR |
| MSC 42676 | Mature | 1030 |  | IR | IR | IR | IR | IR | IR | IR | IR | IR | IR | IR | IR | IR |
| MSC 42674 | Mature | 1040 |  | IR | IR | IR | IR | IR | IR | IR | IR | IR | IR | IR | IR | IR |
| MSC 42675 | Mature | 1043 |  | IR | IR | IR | IR | IR | IR | IR | IR | IR | IR | IR | IR | IR |

**Appendix 1.23. Female dataset for the nature of the cutting edge on the distal heel on the palatoquadrate.** Column header acronyms: **STL** = stretched total length; **Sy** = symphyseal tooth. Descriptive acronyms: **IR** = irregular cutting edge; **SE** = serrated cutting edge; **SM** = smooth cutting edge. Color codes: **Black** = no data; **Dark grey** = serrated cutting edge; **Light grey** = irregular cutting edge; **White** = smooth cutting edge. All pups were *in utero*. Sexual maturity for females based on the presence of pups.

**Appendix 1.24: Distal Heel Cutting Edge – Females – Meckel’s Cartilage**

| **Specimen Data** | | | **Meckel's Cartilage Tooth Position** | | | | | | | | | | | | | |
| --- | --- | --- | --- | --- | --- | --- | --- | --- | --- | --- | --- | --- | --- | --- | --- | --- |
| **MSC Number** | **Lifestage** | **STL (mm)** | **12** | **11** | **10** | **9** | **8** | **7** | **6** | **5** | **4** | **3** | **2** | **1** | **LPa** | **RPa** |
| MSC 44471.4 | Pup | 250 |  | SM | SM | IR | IR | IR | IR | IR | IR | IR | IR | IR | IR | IR |
| MSC 44461.2 | Pup | 310 |  | SM | SM | IR | IR | IR | IR | IR | IR | IR | IR | IR |  |  |
| MSC 44461.4 | Pup | 310 |  | SM | SM | IR | IR | IR | IR | IR | IR | IR | IR | IR | IR | IR |
| MSC 44481.5 | Pup | 310 |  | SM | SM | IR | IR | IR | IR | IR | IR | IR | IR | IR | IR | IR |
| MSC 44481.2 | Pup | 320 |  | SM | SM | IR | IR | IR | IR | IR | IR | IR | IR | IR | IR | IR |
| MSC 44472.4 | Pup | 325 |  | SM | SM | IR | IR | IR | IR | IR | IR | IR | IR | IR | IR | IR |
| MSC 42685.7 | Pup | 330 |  | SM | SM | IR | IR | IR | IR | IR | IR | IR | IR | IR | IR | IR |
| MSC 46740 | N/A | 371 |  | IR | IR | IR | IR | IR | IR | IR | IR | IR | IR | IR | IR | IR |
| MSC 46741 | N/A | 378 |  | IR | IR | IR | IR | IR | IR | IR | IR | IR | IR | IR | IR | IR |
| MSC 42649 | N/A | 600 |  | IR | IR | IR | IR | IR | IR | IR | IR | IR | IR | IR | IR | IR |
| MSC 42645 | N/A | 610 |  | IR | IR | IR | IR | IR | IR | IR | IR | IR | IR | IR | IR | IR |
| MSC 43583 | N/A | 615 |  |  | IR | IR | IR | IR | IR | IR | IR | IR | IR | IR | IR | IR |
| MSC 42655 | N/A | 625 |  | IR | IR | IR | IR | IR | IR | IR | IR | IR | IR | IR | IR | IR |
| MSC 43584 | N/A | 630 |  | IR | IR | IR | IR | IR | IR | IR | IR | IR | IR | IR | IR | IR |
| MSC 43585 | N/A | 635 |  | IR | IR | IR | IR | IR | IR | IR | IR | IR | IR | IR | IR | IR |
| MSC 43586 | N/A | 646 |  | IR | IR | IR | IR | IR | IR | IR | IR | IR | IR | IR | IR | IR |
| MSC 42638 | N/A | 675 |  | IR | IR | IR | IR | IR | IR | IR | IR | IR | IR | IR | IR | IR |
| MSC 44471.1 | Mature | 862 |  | IR | IR | IR | IR | IR | IR | IR | IR | IR | IR | IR | IR | IR |
| MSC 44462 | N/A | 879 |  | IR | IR | IR | IR | IR | IR | IR | IR | IR | IR | IR | IR | IR |
| MSC 42685.1 | Mature | 895 |  | SE | IR | IR | IR | IR | IR | IR | IR | IR | IR | IR | IR | IR |
| MSC 44459 | N/A | 899 |  | IR | SE | SE | SE | IR | IR | IR | IR | IR | SE | SE | IR | IR |
| MSC 44470.1 | Mature | 922 |  | IR | IR | IR | IR | IR | IR | IR | IR | IR | IR | IR | IR | IR |
| MSC 44482.1 | Mature | 940 |  | SE | SE | SE | IR | IR | IR | IR | IR | SE | IR | SE | IR | IR |
| MSC 42666 | Mature | 960 |  | SE | IR | IR | IR | IR | IR | IR | IR | IR | IR | SE | IR | IR |
| MSC 42683 | Mature | 960 |  | IR | IR | IR | IR | IR | IR | IR | IR | IR | IR | SE | IR | IR |
| MSC 42678 | Mature | 966 |  | IR | IR | IR | IR | IR | IR | IR | IR | IR | IR | IR | SE | IR |
| MSC 42673 | Mature | 977 |  | IR | IR | IR | IR | IR | IR | IR | IR | IR | IR | IR | IR | IR |
| MSC 44481.1 | Mature | 980 |  | IR | IR | IR | SE | IR | IR | IR | IR | IR | IR | IR | IR | IR |
| MSC 44461.1 | Mature | 991 |  | SE | SE | SE | IR | IR | IR | IR | IR | IR | IR | IR | IR | IR |
| MSC 42681 | Mature | 997 |  | IR | SE | IR | SE | IR | IR | IR | IR | IR | IR | IR | IR | IR |
| MSC 42670 | Mature | 1001 |  | SE | SE | SE | SE | SE | IR | IR | SE | IR | IR | IR | SE | SE |
| MSC 42672 | Mature | 1002 |  | IR | IR | IR | IR | IR | IR | IR | IR | IR | IR | IR | IR | IR |
| MSC 42679 | Mature | 1007 |  | SE | IR | IR | SE | SE | IR | IR | IR | SE | SE | IR | IR | IR |
| MSC 42669 | Mature | 1011 |  | IR | IR | IR | IR | IR | IR | IR | IR | IR | IR | IR | IR | IR |
| MSC 44472.1 | Mature | 1021 |  | SE | SE | IR | IR | IR | IR | IR | IR | IR | IR | SE | IR | IR |
| MSC 42677 | Mature | 1025 |  | IR | IR | IR | IR | IR | IR | IR | IR | IR | IR | IR | IR | IR |
| MSC 42680 | Mature | 1025 |  | IR | IR | SE | IR | SE | SE | SE | IR | IR | IR | IR | IR | IR |
| MSC 42676 | Mature | 1030 |  | IR | IR | IR | IR | IR | IR | IR | IR | IR | IR | IR | IR | IR |
| MSC 42674 | Mature | 1040 | IR | IR | IR | IR | IR | IR | IR | IR | IR | IR | IR | IR | IR | IR |
| MSC 42675 | Mature | 1043 |  | IR | IR | IR | IR | IR | IR | IR | IR | IR | IR | IR | IR | IR |

**Appendix 1.24. Female dataset for the nature of the cutting edge on the distal heel on the Meckel’s cartilage.** Column header acronyms: **LPa** = left parasymphyseal tooth; **RPa** = right parasymphyseal tooth; **STL** = stretched total length. Descriptive acronyms: **IR** = irregular cutting edge; **SE** = serrated cutting edge; **SM** = smooth cutting edge. Color codes: **Black** = no data; **Dark grey** = serrated cutting edge; **Light grey** = irregular cutting edge; **White** = smooth cutting edge. * = gynandric tooth morphology. All pups were *in utero*. Sexual maturity for females based on the presence of pups.

**Appendix 1.25: Distal Heel Cutting Edge – Males – Palatoquadrate**

| **Specimen Data** | | | **Palatoquadrate Tooth Position** | | | | | | | | | | | | | |
| --- | --- | --- | --- | --- | --- | --- | --- | --- | --- | --- | --- | --- | --- | --- | --- | --- |
| **MSC Number** | **Lifestage** | **STL (mm)** | **13** | **12** | **11** | **10** | **9** | **8** | **7** | **6** | **5** | **4** | **3** | **2** | **1** | **Sy** |
| MSC 44470.2 | Pup | 250 |  | SM | IR | IR | IR | IR | IR | IR | IR | IR | IR | IR | IR | IR |
| MSC 44471.2 | Pup | 260 |  | SM | SM | IR | IR | IR | IR | IR | IR | IR | IR | IR | IR |  |
| MSC 44471.3 | Pup | 260 |  | SM | SM | IR | IR | IR | IR | IR | IR | IR | IR | IR | IR | IR |
| MSC 44482.2 | Pup | 275 |  | SM | SM | SM | IR | IR | IR | IR | IR | IR | IR | IR | IR | IR |
| MSC 44482.3 | Pup | 280 |  | SM | SM | IR | IR | IR | IR | IR | IR | IR | IR | IR | IR | IR |
| MSC 44482.6 | Pup | 280 |  | SM | SM | IR | IR | IR | IR | IR | IR | IR | IR | IR | IR | IR |
| MSC 44482.7 | Pup | 280 |  | SM | SM | IR | IR | IR | IR | IR | IR | IR | IR | IR | IR | IR |
| MSC 44482.4 | Pup | 290 |  | SM | SM | IR | IR | IR | IR | IR | IR | IR | IR | IR | IR | SM |
| MSC 44482.5 | Pup | 290 |  | SM | SM | IR | IR | IR | IR | IR | IR | IR | IR | IR | IR | IR |
| MSC 44461.6 | Pup | 295 |  | SM | SM | IR | IR | IR | IR | IR | IR | IR | IR | SM | SM | SM |
| MSC 44461.3 | Pup | 300 |  | SM | SM | IR | IR | IR | IR | IR | IR | IR | IR | IR | IR | IR |
| MSC 44472.2 | Pup | 300 |  | SM | SM | IR | IR | IR | IR | IR | IR | IR | IR | IR | IR | IR |
| MSC 44472.7 | Pup | 300 |  | SM | SM | IR | IR | IR | IR | IR | IR | IR | IR | IR | IR | IR |
| MSC 44461.5 | Pup | 310 |  | IR | IR | IR | IR | IR | IR | IR | IR | IR | IR | IR | IR | SM |
| MSC 44481.3 | Pup | 310 |  | SM | SM | IR | IR | IR | IR | IR | IR | IR | IR | IR | IR | IR |
| MSC 44472.5 | Pup | 315 |  | SM | SM | IR | IR | IR | IR | IR | IR | IR | IR | IR | IR | SM |
| MSC 44472.6 | Pup | 315 |  | SM | SM | IR | IR | IR | IR | IR | IR | IR | IR | SM | IR | IR |
| MSC 44472.3 | Pup | 320 |  | SM | SM | IR | IR | IR | IR | IR | IR | IR | IR | IR | IR | IR |
| MSC 44481.4 | Pup | 320 |  | SM | SM | IR | IR | IR | IR | IR | IR | IR | IR | IR | IR | IR |
| MSC 42685.5 | Pup | 330 |  | SM | SM | SM | SM | IR | IR | IR | IR | IR | IR | IR | IR | SM |
| MSC 46739 | Immature | 462 |  | IR | IR | IR | IR | IR | IR | IR | IR | IR | IR | IR | IR | IR |
| MSC 42652 | Immature | 540 |  | IR | IR | IR | IR | IR | IR | IR | IR | IR | IR | IR | IR | IR |
| MSC 44465 | Immature | 540 |  | IR | IR | IR | IR | IR | IR | IR | IR | IR | IR | IR | IR | IR |
| MSC 44477 | Immature | 545 |  |  | IR | IR | IR | IR | IR | IR | IR | IR | IR | IR | IR | IR |
| MSC 42643 | Immature | 565 |  | IR | IR | IR | IR | IR | IR | IR | IR | IR | IR | IR | IR | IR |
| MSC 42668 | Immature | 585 |  | IR | IR | IR | IR | IR | IR | IR | IR | IR | IR | IR | IR | IR |
| MSC 44473 | Immature | 585 |  | IR | IR | IR | IR | IR | IR | IR | IR | IR | IR | IR | IR |  |
| MSC 42661 | Immature | 590 | IR | IR | IR | IR | IR | IR | IR | IR | IR | IR | IR | IR | IR | IR |
| MSC 42665 | Immature | 590 |  | IR | IR | IR | IR | IR | IR | IR | IR | IR | IR | IR | IR | IR |
| MSC 44460 | Immature | 595 |  | IR | IR | IR | IR | IR | IR | IR | IR | IR | IR | IR | IR | IR |
| MSC 44469 | Immature | 600 |  | IR | IR | IR | IR | IR | IR | IR | IR | IR | IR | IR | IR | IR |
| MSC 44476 | Immature | 600 |  | IR | IR | IR | IR | IR | IR | IR | IR | IR | IR | IR | IR | IR |
| MSC 42640 | Immature | 605 |  | IR | IR | IR | IR | IR | IR | IR | IR | IR | IR | IR | SE | IR |
| MSC 42662 | Immature | 605 |  | IR | IR | IR | IR | IR | IR | IR | IR | IR | IR | IR | IR | IR |
| MSC 42687 | Immature | 605 |  | IR | IR | IR | IR | IR | IR | IR | IR | IR | IR | IR | IR | IR |
| MSC 44466 | Immature | 605 |  | IR | SE | SE | IR | IR | IR | IR | IR | IR | IR | SE | SE | IR |
| MSC 42663 | Immature | 610 |  | IR | IR | IR | IR | IR | IR | IR | IR | IR | IR | IR | IR | IR |
| MSC 44474 | Immature | 615 |  | IR | IR | IR | IR | IR | IR | IR | IR | IR | IR | IR | IR | IR |
| MSC 42653 | Immature | 620 |  | IR | IR | IR | IR | IR | IR | IR | IR | IR | IR | IR | IR | IR |
| MSC 42641 | Immature | 635 |  | IR | IR | IR | IR | IR | IR | IR | IR | IR | IR | IR | IR | IR |
| MSC 42657 | Immature | 635 |  | IR | IR | IR | IR | IR | IR | IR | IR | IR | IR | IR | IR | IR |
| MSC 44467 | Immature | 635 | IR | IR | IR | IR | IR | IR | IR | IR | IR | IR | IR | IR | IR | IR |
| MSC 44475 | Immature | 635 |  | IR | IR | IR | IR | IR | SE | IR | SE | IR | SE | SE | SE | IR |
| MSC 42664 | Immature | 640 |  | IR | IR | IR | IR | IR | IR | IR | IR | IR | IR | IR | IR |  |
| MSC 42650 | Immature | 645 |  | SE | SE | IR | IR | SE |  | IR | SE | IR | IR | SE | SE | IR |
| MSC 42639 | Immature | 650 |  | IR | IR | IR | IR | IR | IR | SE | IR | IR | IR | SE | IR |  |
| MSC 42654 | Immature | 650 |  | IR | IR | IR | SE | IR | IR | SE | IR | IR | IR | SE | IR | SE |
| MSC 42659 | Immature | 650 |  | IR | IR | IR | IR | IR | IR | IR | IR | IR | IR | IR | IR |  |
| MSC 44455 | Immature | 650 |  | IR | IR | IR | IR | IR | IR | IR | IR | IR | IR | IR | IR | IR |
| MSC 44468 | Immature | 650 |  | IR | IR | IR | SE | SE | IR | SE | IR | SE | SE | IR | IR | IR |
| MSC 44464 | Immature | 655 |  | IR | IR | IR | IR | IR | IR | IR | IR | IR | IR | IR | IR | IR |
| MSC 44478 | Mature | 660 |  | IR | IR | IR | IR | IR | IR | IR | IR | IR | IR | IR | IR | IR |
| MSC 42647 | Immature | 665 |  | IR | IR | IR | IR | IR | IR | IR | IR | IR | IR | IR | IR | IR |
| MSC 42651 | Immature | 675 |  | IR | IR | IR | SE | IR | IR | IR | IR | IR | IR | IR | IR | IR |
| MSC 44463 | Immature | 675 |  | IR | IR | IR | IR | IR | IR | IR | IR | IR | IR | IR | IR | IR |
| MSC 42648 | Immature | 680 |  | IR | SE | IR | IR | IR | IR | IR | IR | IR | IR | IR | SE | IR |
| MSC 42686 | Immature | 690 |  | IR | IR | IR | IR | IR | IR | IR | IR | IR | IR | IR | IR |  |
| MSC 42682 | Immature | 715 |  | SE | IR | IR | IR | IR | IR | IR | IR | IR | IR | IR | SE |  |
| MSC 43587 | Immature | 725 |  | SE | IR | SE | IR | IR | IR | IR | IR | IR | IR | IR | IR | IR |
| MSC 42660 | Immature | 735 |  | IR | IR | IR | IR | IR | IR | IR | IR | IR | IR | SE | SE | IR |
| MSC 42684 | Immature | 735 |  | IR | IR | IR | IR | IR | IR | IR | IR | IR | IR | IR | IR | IR |
| MSC 42667 | Immature | 745 | IR | IR | IR | IR | IR | IR | IR | IR | IR | IR | IR | SE | IR | IR |
| MSC 42637 | Immature | 750 |  | IR | IR | IR | IR | IR | IR | IR | IR | IR | IR | IR | IR | IR |
| MSC 42646 | Immature | 775 |  | IR | SE | SE | SE | IR | IR | IR | IR | IR | IR | IR | IR |  |
| MSC 42658 | Transitional | 790 |  | SE | SE | SE | IR | IR | IR | IR | IR | IR | SE | IR | IR | IR |
| MSC 43588 | Transitional | 805 |  | IR | IR | IR | IR | IR | IR | IR | IR | IR | IR | IR | IR | IR |
| MSC 43589 | Mature | 815 |  | IR | IR | IR | IR | SE | IR | SE | IR | IR | SE | SE | SE | IR |
| MSC 44479 | Mature | 870 |  | IR | SE | SE | IR | SE | IR | SE | IR | IR | IR | IR | IR | SM |
| MSC 43590 | Transitional | 885 | IR | IR | IR | IR | IR | IR | IR | IR | IR | IR | IR | IR | IR |  |
| MSC 42656 | Mature | 895 |  | SE | SE | IR | IR | IR | SE | SE | IR | IR | SE | SE | SE | SE |
| MSC 44454 | Mature | 935 |  | IR | IR | IR | SE | IR | IR | IR | IR | SE | IR | IR | IR | SE |
| MSC 42644 | Mature | 955 |  | IR | IR | IR | IR | SE | IR | IR | SE | IR | SE | SE | IR | IR |
| MSC 46703 | Mature | 964 |  | SE | SE | SE | SE | SE | SE | SE | SE | SE | SE | SE | IR | SE |
| MSC 46736 | Mature | 966 |  | IR | SE | IR | SE | SE | SE | SE | SE | SE | SE | IR | IR | SE |
| MSC 44480 | Mature | 980 | IR | IR | IR | IR | IR | IR | IR | IR | IR | IR | IR | IR | IR | IR |
| MSC 46737 | Mature | 984 |  | SM | IR | IR | IR | IR | SE | SE | SE | SE | SE | SE | IR | IR |
| MSC 44457 | Mature | 990 | IR | IR | IR | IR | IR | IR | IR | IR | IR | IR | SE | SE | SE | SE |
| MSC 46735 | Mature | 994 | IR | IR | IR | IR | SE | SE | IR | SE | SE | IR | IR | IR | SE | IR |
| MSC 42671 | Mature | 1000 | IR | IR | IR | IR | IR | IR | IR | IR | IR | IR | IR | IR | SE | SE |
| MSC 46739 | Mature | 1001 | IR | IR | IR | SE | IR | SE | SE | SE | SE | SE | SE | SE | IR | IR |
| MSC 44458 | Mature | 1010 |  | IR | IR | IR | IR | IR | IR | IR | IR | IR | IR | IR | IR | IR |
| MSC 44456 | Mature | 1033 |  | IR | IR | IR | IR | IR | IR | SE | IR | IR | SE | SE | SE | SE |

**Appendix 1.25. Male dataset for the nature of the cutting edge on the distal heel on the palatoquadrate.** Column header acronyms: **STL** = stretched total length; **Sy** = symphyseal tooth. Descriptive acronyms: **IR** = irregular cutting edge; **SE** = serrated cutting edge; **SM** = smooth cutting edge. Color codes: **Black** = no data; **Dark grey** = serrated cutting edge; **Light grey** = irregular cutting edge; **White** = smooth cutting edge. All pups were *in utero*. Life stage for males based on extent of calcification of the myxopterygia (see Materials & Methods).

**Appendix 1.26: Distal Heel Cutting Edge – Males – Meckel’s Cartilage**

| **Specimen Data** | | | **Meckel's Cartilage Tooth Position** | | | | | | | | | | | | | |
| --- | --- | --- | --- | --- | --- | --- | --- | --- | --- | --- | --- | --- | --- | --- | --- | --- |
| **MSC Number** | **Lifestage** | **STL (mm)** | **12** | **11** | **10** | **9** | **8** | **7** | **6** | **5** | **4** | **3** | **2** | **1** | **LPa** | **RPa** |
| MSC 44470.2 | Pup | 250 |  | SM | SM | IR | IR | IR | IR | IR | IR | IR | IR | IR | IR | IR |
| MSC 44471.2 | Pup | 260 |  | SM | SM | IR | SM | IR | IR | IR | IR | IR | IR | IR | SM | IR |
| MSC 44471.3 | Pup | 260 |  | SM | SM | IR | IR | IR | IR | IR | IR | IR | IR | IR | IR | IR |
| MSC 44482.2 | Pup | 275 |  | SM | SM | IR | IR | IR | IR | IR | IR | IR | IR | IR | IR | IR |
| MSC 44482.3 | Pup | 280 |  | SM | SM | IR | IR | IR | IR | IR | IR | IR | IR | IR | IR | SM |
| MSC 44482.6 | Pup | 280 |  | SM | SM | SM | IR | IR | IR | IR | IR | IR | IR | IR | IR | IR |
| MSC 44482.7 | Pup | 280 |  | SM | SM | IR | IR | IR | IR | IR | IR | IR | IR | IR | IR | IR |
| MSC 44482.4 | Pup | 290 |  | SM | SM | IR | IR | IR | IR | IR | IR | IR | IR | IR | SM | SM |
| MSC 44482.5 | Pup | 290 |  | SM | SM | IR | IR | IR | IR | SM | IR | IR | IR | IR | IR | IR |
| MSC 44461.6 | Pup | 295 |  | SM | SM | SM | IR | IR | IR | IR | IR | SM | IR | SM | IR | IR |
| MSC 44461.3 | Pup | 300 |  | SM | SM | IR | IR | IR | IR | IR | IR | IR | IR | IR | IR | IR |
| MSC 44472.2 | Pup | 300 |  | SM | SM | IR | IR | IR | IR | IR | IR | IR | IR | IR | IR | IR |
| MSC 44472.7 | Pup | 300 |  | SM | SM | IR | IR | IR | IR | IR | IR | IR | IR | IR | IR | IR |
| MSC 44461.5 | Pup | 310 |  | SM | SM | IR | IR | IR | IR | IR | IR | IR | IR | IR | IR |  |
| MSC 44481.3 | Pup | 310 |  | SM | SM | IR | IR | IR | IR | IR | IR | IR | IR | IR | IR | IR |
| MSC 44472.5 | Pup | 315 |  | SM | SM | IR | IR | IR | IR | IR | IR | IR | IR | IR | IR | IR |
| MSC 44472.6 | Pup | 315 |  | SM | SM | IR | SM | IR | IR | IR | IR | IR | IR | IR | IR | IR |
| MSC 44472.3 | Pup | 320 |  | SM | IR | SM | IR | IR | IR | IR | IR | IR | IR | IR | IR | IR |
| MSC 44481.4 | Pup | 320 |  | SM | SM | IR | IR | IR | IR | IR | IR | IR | IR | IR |  | IR |
| MSC 42685.5 | Pup | 330 |  | SM | SM | SM | IR | IR | IR | IR | IR | SM | SM | IR | SM | SM |
| MSC 46739 | Immature | 462 |  | IR | IR | IR | IR | IR | IR | IR | IR | IR | IR | IR | IR | IR |
| MSC 42652 | Immature | 540 |  | IR | IR | IR | IR | IR | IR | IR | IR | IR | IR | IR | IR | IR |
| MSC 44465 | Immature | 540 |  | IR | IR | IR | IR | IR | IR | IR | IR | IR | IR | IR | IR | IR |
| MSC 44477 | Immature | 545 |  | IR | IR | IR | IR | IR | IR | IR | IR | IR | IR | IR | IR |  |
| MSC 42643 | Immature | 565 |  | IR | IR | IR | IR | IR | IR | IR | IR | IR | IR | IR | IR | IR |
| MSC 42668 | Immature | 585 |  | IR | IR | IR | IR | IR | IR | IR | IR | IR | IR | IR | IR | IR |
| MSC 44473 | Immature | 585 |  | IR | IR | IR | IR | IR | IR | IR | IR | IR | IR | IR | IR | IR |
| MSC 42661 | Immature | 590 | IR | IR | IR | IR | IR | IR | IR | IR | IR | IR | IR | IR | IR |  |
| MSC 42665 | Immature | 590 |  | IR | IR | IR | IR | IR | IR | IR | IR | IR | IR | IR | IR | IR |
| MSC 44460 | Immature | 595 |  | SE | SE | SM | SM | IR | IR | IR | IR | IR | SM | SM | SM | SM |
| MSC 44469 | Immature | 600 |  | IR | IR | IR | IR | IR | IR | IR | IR | IR | IR | IR | IR | IR |
| MSC 44476 | Immature | 600 |  | IR | IR | IR | IR | IR | IR | IR | IR | IR | IR | IR | IR | IR |
| MSC 42640 | Immature | 605 |  | IR | IR | IR | IR | IR | IR | IR | IR | IR | IR | IR | IR | IR |
| MSC 42662 | Immature | 605 |  | IR | IR | IR | IR | IR | IR | IR | IR | IR | IR | IR | IR | IR |
| MSC 42687 | Immature | 605 |  | SE | IR | IR | IR | IR | IR | IR | IR | IR | IR | IR | IR | IR |
| MSC 44466 | Immature | 605 |  | SE | IR | IR | SE | IR | SE | IR | SE | IR | SE | IR | IR | IR |
| MSC 42663 | Immature | 610 |  | IR | IR | IR | IR | IR | IR | IR | IR | IR | IR | IR | IR | IR |
| MSC 44474 | Immature | 615 |  | IR | IR | IR | IR | IR | IR | IR | IR | IR | IR | IR | IR | IR |
| MSC 42653 | Immature | 620 |  | IR | IR | IR | IR | IR | IR | IR | IR | IR | IR | IR | IR | IR |
| MSC 42641 | Immature | 635 |  | IR | IR | IR | IR | IR | IR | IR | IR | IR | IR | IR | IR | IR |
| MSC 42657 | Immature | 635 |  | IR | IR | IR | IR | IR | IR | IR | IR | IR | IR | IR | IR | IR |
| MSC 44467 | Immature | 635 |  | IR | IR | IR | IR | IR | IR | IR | IR | IR | IR | IR | IR | IR |
| MSC 44475 | Immature | 635 |  | IR | SE | SE | SE | SE | IR | SE | IR | IR | IR | IR | SE | IR |
| MSC 42664 | Immature | 640 |  | IR | IR | IR | IR | IR | IR | IR | IR | IR | IR | IR | IR |  |
| MSC 42650 | Immature | 645 |  | IR | SE | SE | SE | IR | IR | IR | IR | IR | IR | IR | IR | IR |
| MSC 42639 | Immature | 650 | IR | IR | IR | IR | SE | SE | IR | IR | IR | IR | SE | SE | IR |  |
| MSC 42654 | Immature | 650 |  | IR | SE | IR | SE | IR | IR | IR | IR | IR | IR | IR | SE |  |
| MSC 42659 | Immature | 650 |  | IR | IR | SE | IR | IR | IR | IR | IR | SE | SE | SE |  | IR |
| MSC 44455 | Immature | 650 |  | IR | IR | IR | IR | IR | IR | IR | IR | IR | IR | IR | IR | IR |
| MSC 44468 | Immature | 650 |  | IR | IR | SE | SE | SE | SE | SE | IR | IR | IR | IR | IR | IR |
| MSC 44464 | Immature | 655 |  | IR | IR | IR | IR | IR | IR | IR | IR | IR | IR | IR | IR | IR |
| MSC 44478 | Mature | 660 |  | IR | IR | IR | IR | IR | IR | IR | IR | IR | IR | IR | IR | IR |
| MSC 42647 | Immature | 665 |  | IR | IR | IR | IR | IR | IR | IR | IR | IR | IR | IR | IR | IR |
| MSC 42651 | Immature | 675 |  | IR | IR | IR | SE | IR | IR | IR | IR | IR | IR | IR | IR | IR |
| MSC 44463 | Immature | 675 |  | IR | IR | IR | IR | IR | IR | IR | IR | IR | IR | IR | IR |  |
| MSC 42648 | Immature | 680 |  | IR | IR | IR | SE | IR | IR | IR | IR | IR | IR | IR | IR | IR |
| MSC 42686 | Immature | 690 |  | IR | IR | IR | IR | IR | IR | IR | IR | IR | IR | IR | IR | IR |
| MSC 42682 | Immature | 715 |  | SE | IR | IR | IR | SE | IR | IR | IR | IR | IR | IR | SE | IR |
| MSC 43587 | Immature | 725 |  | SE | SE | SE | SE | IR | IR | SE | IR | IR | IR | IR | IR | IR |
| MSC 42660 | Immature | 735 |  | IR | IR | IR | IR | IR | IR | IR | IR | IR | IR | IR | IR | IR |
| MSC 42684 | Immature | 735 |  | IR | IR | IR | IR | IR | IR | IR | IR | IR | IR | IR | IR | IR |
| MSC 42667 | Immature | 745 |  | IR | IR | IR | IR | IR | IR | IR | IR | IR | IR | IR | IR | IR |
| MSC 42637 | Immature | 750 |  | IR | IR | IR | IR | IR | IR | IR | IR | IR | IR | IR | IR | IR |
| MSC 42646 | Immature | 775 |  | SE | IR | IR | IR | IR | IR | IR | IR | IR | IR | IR | IR | IR |
| MSC 42658 | Transitional | 790 |  | IR | IR | SE | IR | SE | IR | IR | IR | IR | SE | IR | IR | IR |
| MSC 43588 | Transitional | 805 |  | IR | IR | IR | IR | IR | IR | IR | IR | IR | IR | IR | IR | IR |
| MSC 43589 | Mature | 815 |  | IR | IR | IR | IR | IR | IR | IR | IR | IR | IR | IR | IR | IR |
| MSC 44479 | Mature | 870 |  | SE | SE | SE | SE | SE | SE | IR | IR | IR | IR | SE | IR | IR |
| MSC 43590 | Transitional | 885 | SM | IR | IR | IR | IR | IR | IR | IR | IR | IR | IR | IR | IR | IR |
| MSC 42656 | Mature | 895 |  | SE | SE | SE | SE | SE | SE | SE | SE | IR | SE | IR | IR | SE |
| MSC 44454 | Mature | 935 |  | SE | SE | IR | IR | IR | IR | IR | IR | SE | IR | IR | SE | IR |
| MSC 42644 | Mature | 955 |  | IR | IR | IR | IR | IR | IR | SE | SE | IR | IR | IR | IR | IR |
| MSC 46703 | Mature | 964 |  | IR | IR | IR | IR | SE | SE | SE | IR | SE | SE | SE | IR | IR |
| MSC 46736 | Mature | 966 |  | IR | IR | IR | IR | IR | IR | SE | IR | SE | SE | SM* | SM* | SM* |
| MSC 44480 | Mature | 980 |  | SE | IR | IR | IR | IR | SE | IR | IR | IR | SE | SM* | SM* | SM* |
| MSC 46737 | Mature | 984 |  | IR | IR | IR | IR | IR | IR | IR | IR | IR | IR | SM* | SM* | SM* |
| MSC 44457 | Mature | 990 | SE | SE | IR | IR | IR | IR | SE | SE | IR | IR | SE | SM* | SM* | SM* |
| MSC 46735 | Mature | 994 |  | IR | IR | IR | IR | SE | SE | SE | IR | SE | SE | SM* | SM* | SM* |
| MSC 42671 | Mature | 1000 | SE | SE | IR | IR | IR | IR | IR | IR | IR | IR | IR | SM* | SM* | SM* |
| MSC 46739 | Mature | 1001 | IR | SE | SE | SE | SE | SE | SE | IR | SE | SE | SE | SM* | SM* | SM* |
| MSC 44458 | Mature | 1010 |  | IR | IR | IR | SE | SE | IR | IR | IR | IR | SM* | SM* | SM* | SM* |
| MSC 44456 | Mature | 1033 |  | SE | IR | SE | IR | IR | IR | IR | SE | SE | SM* | SM* | SM* | SM* |

**Appendix 1.26. Male dataset for the nature of the cutting edge on the distal heel of the Meckel’s cartilage.** Column header acronyms: **LPa** = left parasymphyseal tooth; **RPa** = right parasymphyseal tooth; **STL** = stretched total length. Descriptive acronyms: **IR** = irregular cutting edge; **SE** = serrated cutting edge; **SM** = smooth cutting edge. Color codes: **Black** = no data; **Dark grey** = serrated cutting edge; **Light grey** = irregular cutting edge; **White** = smooth cutting edge. * = gynandric tooth morphology. All pups were *in utero*. Life stage for males based on extent of calcification of the myxopterygia (see Materials & Methods).

**Appendix 1.27: Distal Cutting Edge – Females – Palatoquadrate**

| **Specimen Data** | | | **Palatoquadrate Tooth Position** | | | | | | | | | | | | | |
| --- | --- | --- | --- | --- | --- | --- | --- | --- | --- | --- | --- | --- | --- | --- | --- | --- |
| **MSC Number** | **Lifestage** | **STL (mm)** | **13** | **12** | **11** | **10** | **9** | **8** | **7** | **6** | **5** | **4** | **3** | **2** | **1** | **Sy** |
| MSC 44471.4 | Pup | 250 |  | SM | SM | IR | IR | IR | IR | IR | IR | IR | IR | IR | IR | SM |
| MSC 44461.2 | Pup | 310 | SM | SM | IR | IR | IR | IR | IR | IR | IR | IR | IR | IR | IR | IR |
| MSC 44461.4 | Pup | 310 |  | SM | SM | IR | IR | IR | IR | IR | IR | IR | IR | IR | IR | IR |
| MSC 44481.5 | Pup | 310 |  | SM | SM | IR | IR | IR | IR | IR | IR | IR | IR | IR | IR | IR |
| MSC 44481.2 | Pup | 320 |  | SM | SM | SM | IR | IR | IR | IR | IR | IR | IR | IR | IR | IR |
| MSC 44472.4 | Pup | 325 |  | SM | IR | IR | IR | IR | IR | IR | IR | IR | IR | IR | IR | IR |
| MSC 42685.7 | Pup | 330 |  | SM | SM | IR | SM | IR | IR | SM | SM | IR | SM | IR | IR | IR |
| MSC 46740 | N/A | 371 |  | SM | SM | SM | IR | IR | IR | IR | IR | IR | IR | IR | IR | SM |
| MSC 46741 | N/A | 378 |  | SM | SM | SM | SM | SM | IR | SM | IR | IR | IR | IR | IR | IR |
| MSC 42649 | N/A | 600 |  | SE | SE | SE | SE | SE | SE | SE | SE | IR | IR | IR | IR | IR |
| MSC 42645 | N/A | 610 |  | IR | IR | IR | IR | IR | IR | IR | IR | IR | IR | IR | IR | IR |
| MSC 43583 | N/A | 615 |  |  |  | SE | SE | SE | IR | SE | SE | IR | IR | IR | IR | IR |
| MSC 42655 | N/A | 625 |  | IR | IR | IR | IR | IR | IR | IR | IR | IR | IR | IR | IR | IR |
| MSC 43584 | N/A | 630 |  | IR | IR | IR | IR | IR | IR | IR | IR | IR | IR | IR | IR | IR |
| MSC 43585 | N/A | 635 |  | SE | SE | IR | IR | SE | SE | SE | SE | SE | SE | IR | SE | IR |
| MSC 43586 | N/A | 646 | IR | SE | SE | SE | IR | SE | SE | IR | IR | IR | IR | IR | IR | IR |
| MSC 42638 | N/A | 675 |  | IR | IR | IR | IR | IR | IR | IR | IR | IR | IR | IR | IR | IR |
| MSC 44471.1 | Mature | 862 |  | IR | IR | IR | IR | IR | IR | IR | IR | IR | IR | IR | IR | IR |
| MSC 44462 | N/A | 879 |  | SM | IR | IR | SE | IR | SE | IR | SE | IR | SE | IR | SE | IR |
| MSC 42685.1 | Mature | 895 | SM | SE | SE | IR | IR | SE | SE | SE | SE | IR | IR | IR | SE | SE |
| MSC 44459 | N/A | 899 |  | SE | SE | SE | SE | SE | SE | IR | SE | SE | SE | SE | IR | SE |
| MSC 44470.1 | Mature | 922 |  | IR | IR | IR | IR | IR | IR | IR | IR | IR | IR | IR | IR |  |
| MSC 44482.1 | Mature | 940 |  | IR | IR | IR | IR | IR | SE | IR | IR | IR | IR | IR | IR | IR |
| MSC 42666 | Mature | 960 |  | SE | SE | SE | SE | SE | IR | IR | IR | IR | IR | IR | SE | SE |
| MSC 42683 | Mature | 960 |  | SE | IR | IR | SE | SE | SE | SE | SE | IR | IR | IR | SE | IR |
| MSC 42678 | Mature | 966 |  | SE | SE | SE | IR | SE | IR | IR | IR | IR | IR | IR | SE | IR |
| MSC 42673 | Mature | 977 |  | IR | IR | IR | IR | IR | IR | IR | IR | IR | IR | IR | IR |  |
| MSC 44481.1 | Mature | 980 |  | SM | IR | SE | IR | IR | IR | IR | IR | IR | IR | IR | IR | IR |
| MSC 44461.1 | Mature | 991 |  | IR | IR | IR | IR | IR | IR | IR | IR | IR | IR | IR | IR | IR |
| MSC 42681 | Mature | 997 |  | SE | SE | IR | IR | IR | SE | SE | IR | IR | IR | IR | IR | IR |
| MSC 42670 | Mature | 1001 |  | SM | SM | SE | SE | SE | SE | SE | IR | IR | IR | IR | SE | SE |
| MSC 42672 | Mature | 1002 |  |  | IR | IR | IR | IR | IR | IR | IR | IR | IR | IR | IR | IR |
| MSC 42679 | Mature | 1007 |  | SM | SE | SE | SE | SE | SE | SE | SE | SE | SE | SE | SE | SE |
| MSC 42669 | Mature | 1011 |  | IR | IR | IR | IR | IR | IR | IR | IR | IR | IR | IR | IR | IR |
| MSC 44472.1 | Mature | 1021 |  | SE | SE | IR | SE | IR | IR | IR | IR | IR | IR | IR | IR | IR |
| MSC 42677 | Mature | 1025 |  | IR | IR | IR | IR | IR | IR | IR | IR | IR | IR | IR | IR | IR |
| MSC 42680 | Mature | 1025 |  | SE | SE | SE | SE | IR | IR | IR | IR | IR | IR | IR | IR | IR |
| MSC 42676 | Mature | 1030 |  | SE | SE | SE | IR | IR | IR | IR | SE | IR | IR | IR | IR | IR |
| MSC 42674 | Mature | 1040 |  | SM | SE | SE | SE | SE | IR | IR | IR | IR | IR | IR | IR | IR |
| MSC 42675 | Mature | 1043 |  | IR | IR | IR | IR | IR | IR | IR | IR | IR | IR | IR | IR | IR |

**Appendix 1.27. Female dataset for the nature of the distal cutting edge on the palatoquadrate.** Column header acronyms: **STL** = stretched total length; **Sy** = symphyseal tooth. Descriptive acronyms: **IR** = irregular cutting edge; **SE** = serrated cutting edge; **SM** = smooth cutting edge. * = gynandric tooth morphology. Color codes: **Black** = no data; **Dark grey** = serrated cutting edge; **Light grey** = irregular cutting edge; **White** = smooth cutting edge. All pups were *in utero*. Sexual maturity for females based on the presence of pups.

**Appendix 1.28: Distal Cutting Edge – Females – Meckel’s Cartilage**

| **Specimen Data** | | | **Meckel's Cartilage Tooth Position** | | | | | | | | | | | | | |
| --- | --- | --- | --- | --- | --- | --- | --- | --- | --- | --- | --- | --- | --- | --- | --- | --- |
| **MSC Number** | **Lifestage** | **STL (mm)** | **12** | **11** | **10** | **9** | **8** | **7** | **6** | **5** | **4** | **3** | **2** | **1** | **LPa** | **RPa** |
| MSC 44471.4 | Pup | 250 |  | SM | SM | SM | IR | IR | IR | IR | IR | IR | IR | IR | IR | IR |
| MSC 44461.2 | Pup | 310 |  | SM | IR | IR | IR | IR | IR | IR | IR | IR | IR | IR |  |  |
| MSC 44461.4 | Pup | 310 |  | SM | SM | IR | IR | IR | IR | IR | IR | IR | IR | IR | IR | IR |
| MSC 44481.5 | Pup | 310 |  | SM | SM | SM | IR | SM | IR | IR | IR | SM | IR | IR | IR | IR |
| MSC 44481.2 | Pup | 320 |  | SM | SM | IR | IR | IR | IR | SM | IR | IR | IR | IR | IR | IR |
| MSC 44472.4 | Pup | 325 |  | SM | SM | IR | IR | IR | IR | IR | IR | IR | IR | IR | IR | IR |
| MSC 42685.7 | Pup | 330 |  | SM | SM | SM | IR | IR | IR | SM | IR | SM | IR | IR | IR | IR |
| MSC 46740 | N/A | 371 |  | SM | SM | SM | SM | SM | SM | SM | SM | SM | IR | SM | IR | IR |
| MSC 46741 | N/A | 378 |  | SM | SM | SM | SM | SM | IR | IR | IR | SM | SM | SM | IR | IR |
| MSC 42649 | N/A | 600 |  | SM | SM | SE | SE | SE | SE | SE | SE | IR | IR | IR | SE | SE |
| MSC 42645 | N/A | 610 |  | IR | SM | IR | IR | IR | IR | IR | IR | IR | IR | IR | IR | IR |
| MSC 43583 | N/A | 615 |  |  |  | SE | SE | SE | SE | SE | SE | SE | IR | IR | IR | IR |
| MSC 42655 | N/A | 625 |  | IR | IR | IR | IR | IR | IR | IR | IR | IR | IR | IR |  | IR |
| MSC 43584 | N/A | 630 |  | IR | IR | IR | IR | IR | IR | IR | IR | IR | IR | IR | IR | IR |
| MSC 43585 | N/A | 635 |  | SM | SM | SE | SE | SE | SE | SE | SE | SE | SE | SE | IR | IR |
| MSC 43586 | N/A | 646 |  | SM | SM | SE | SE | SE | IR | IR | SE | SE | IR | IR | SE |  |
| MSC 42638 | N/A | 675 |  | SM | IR | IR | IR | IR | IR | IR | IR | IR | IR | IR | IR | IR |
| MSC 44471.1 | Mature | 862 |  | SM | IR | IR | IR | IR | IR | IR | IR | IR | IR | IR | IR | IR |
| MSC 44462 | N/A | 879 |  | SE | IR | IR | SE | SE | IR | IR | IR | IR | IR | IR | IR | IR |
| MSC 42685.1 | Mature | 895 |  | SE | SE | SE | IR | SE | SE | SE | SE | IR | IR | IR | SE | SE |
| MSC 44459 | N/A | 899 |  | SE | SE | SE | SE | SE | SE | IR | SE | IR | SE | SM | SM | SM |
| MSC 44470.1 | Mature | 922 |  | SM | IR | IR | IR | IR | IR | IR | IR | IR | IR | IR | IR | IR |
| MSC 44482.1 | Mature | 940 |  | SM | IR | IR | IR | IR | IR | IR | IR | IR | IR | IR | IR | IR |
| MSC 42666 | Mature | 960 |  | SM | SM | SE | SE | SE | SE | SE | SE | SE | SE | SE | SE | SE |
| MSC 42683 | Mature | 960 | SM | SM | SM | SE | SE | SE | SE | SE | SE | IR | SE | SE | SE | SE |
| MSC 42678 | Mature | 966 |  | SM | SE | SE | SE | IR | IR | IR | IR | IR | SE | SE | IR | IR |
| MSC 42673 | Mature | 977 |  | IR | IR | IR | IR | IR | IR | IR | IR | IR | IR | IR | IR | IR |
| MSC 44481.1 | Mature | 980 |  | IR | IR | IR | IR | IR | SE | IR | IR | IR | IR | IR | IR | IR |
| MSC 44461.1 | Mature | 991 |  | SM | IR | IR | IR | IR | IR | IR | IR | IR | IR | IR | IR | IR |
| MSC 42681 | Mature | 997 |  | SE | IR | SE | IR | SE | IR | IR | IR | IR | IR | SE | IR | SE |
| MSC 42670 | Mature | 1001 |  | SE | SM | SM | SE | SE | SE | SE | IR | SE | IR | SE | SE | SE |
| MSC 42672 | Mature | 1002 |  | IR | IR | IR | IR | IR | IR | IR | IR | IR | IR | IR | IR | IR |
| MSC 42679 | Mature | 1007 |  | SM | SM | SM | SE | SM | SE | SE | SE | SE | SE | SE | SE | SE |
| MSC 42669 | Mature | 1011 |  | IR | IR | IR | IR | IR | IR | IR | IR | IR | IR | IR | IR | IR |
| MSC 44472.1 | Mature | 1021 |  | IR | IR | IR | IR | IR | IR | IR | IR | IR | IR | IR | IR | IR |
| MSC 42677 | Mature | 1025 |  | SE | SE | IR | IR | SE | IR | IR | IR | IR | IR | IR | IR | IR |
| MSC 42680 | Mature | 1025 |  | SE | SE | SE | IR | SE | IR | SE | SE | SE | IR | IR | IR | IR |
| MSC 42676 | Mature | 1030 |  | SE | SE | IR | IR | IR | IR | IR | IR | IR | IR | SE | SE | SE |
| MSC 42674 | Mature | 1040 | SM | SE | SE | SE | SE | IR | SE | IR | IR | IR | IR | SE | IR | IR |
| MSC 42675 | Mature | 1043 |  | IR | IR | IR | IR | IR | IR | IR | IR | IR | IR | IR | IR | IR |

**Appendix 1.28. Female dataset for the nature of the distal cutting edge on the Meckel’s cartilage.** Column header acronyms: **LPa** = left parasymphyseal tooth; **RPa** = right parasymphyseal tooth; **STL** = stretched total length. Descriptive acronyms: **IR** = irregular cutting edge; **SE** = serrated cutting edge; **SM** = smooth cutting edge. Color codes: **Black** = no data; **Dark grey** = serrated cutting edge; **Light grey** = irregular cutting edge; **White** = smooth cutting edge. All pups were *in utero*. Sexual maturity for females based on the presence of pups.

**Appendix 1.29: Distal Cutting Edge – Males – Palatoquadrate**

| **Specimen Data** | | | **Palatoquadrate Tooth Position** | | | | | | | | | | | | | |
| --- | --- | --- | --- | --- | --- | --- | --- | --- | --- | --- | --- | --- | --- | --- | --- | --- |
| **MSC Number** | **Lifestage** | **STL (mm)** | **13** | **12** | **11** | **10** | **9** | **8** | **7** | **6** | **5** | **4** | **3** | **2** | **1** | **Sy** |
| MSC 44470.2 | Pup | 250 |  | SM | SM | IR | SM | IR | IR | IR | IR | IR | IR | IR | IR | IR |
| MSC 44471.2 | Pup | 260 |  | SM | SM | SM | IR | IR | IR | IR | IR | IR | IR | IR | IR |  |
| MSC 44471.3 | Pup | 260 |  | SM | SM | SM | IR | IR | IR | IR | IR | IR | IR | IR | IR | IR |
| MSC 44482.2 | Pup | 275 |  | SM | SM | IR | IR | IR | IR | IR | IR | IR | IR | IR | IR | IR |
| MSC 44482.3 | Pup | 280 |  | SM | SM | SM | IR | IR | IR | IR | IR | IR | IR | IR | IR | IR |
| MSC 44482.6 | Pup | 280 |  | SM | SM | IR | IR | IR | IR | IR | IR | IR | IR | IR | IR | IR |
| MSC 44482.7 | Pup | 280 |  | SM | SM | SM | IR | IR | IR | IR | IR | IR | IR | IR | IR | SM |
| MSC 44482.4 | Pup | 290 |  | SM | SM | SM | IR | IR | IR | IR | IR | IR | IR | IR | IR | SM |
| MSC 44482.5 | Pup | 290 |  | SM | SM | IR | IR | IR | IR | IR | IR | IR | IR | IR | IR | IR |
| MSC 44461.6 | Pup | 295 |  | SM | SM | IR | IR | IR | IR | IR | IR | IR | IR | SM | SM | SM |
| MSC 44461.3 | Pup | 300 |  | SM | SM | IR | IR | IR | IR | IR | IR | IR | IR | IR | IR | IR |
| MSC 44472.2 | Pup | 300 |  | SM | SM | IR | IR | IR | IR | IR | IR | IR | IR | IR | IR | IR |
| MSC 44472.7 | Pup | 300 |  | SM | SM | IR | IR | IR | IR | IR | IR | IR | IR | IR | IR | IR |
| MSC 44461.5 | Pup | 310 |  | IR | IR | IR | IR | IR | IR | IR | IR | IR | IR | IR | IR | IR |
| MSC 44481.3 | Pup | 310 |  | SM | SM | SM | SM | SM | IR | IR | IR | IR | IR | SM | IR | SM |
| MSC 44472.5 | Pup | 315 |  | SM | SM | IR | IR | IR | IR | IR | IR | IR | IR | IR | IR | SM |
| MSC 44472.6 | Pup | 315 |  | IR | IR | IR | IR | IR | IR | IR | IR | IR | IR | IR | IR | IR |
| MSC 44472.3 | Pup | 320 |  | IR | IR | IR | IR | IR | IR | IR | IR | IR | IR | IR | IR | IR |
| MSC 44481.4 | Pup | 320 |  | SM | SM | SM | IR | IR | IR | IR | IR | IR | IR | IR | IR | IR |
| MSC 42685.5 | Pup | 330 |  | SM | SM | IR | IR | IR | IR | IR | SM | IR | IR | IR | SM | IR |
| MSC 46739 | Immature | 462 |  | SM | SM | SM | SM | SM | SM | IR | IR | IR | IR | SM | SM | SM |
| MSC 42652 | Immature | 540 |  | SM | IR | SE | SE | IR | IR | IR | IR | IR | IR | IR | IR | IR |
| MSC 44465 | Immature | 540 |  | IR | IR | IR | IR | IR | IR | IR | IR | SE | IR | IR | IR | IR |
| MSC 44477 | Immature | 545 |  |  | SE | SE | SE | SE | SM | IR | SE | IR | SE | SE | SE | SE |
| MSC 42643 | Immature | 565 |  | SE | SE | IR | IR | IR | IR | IR | IR | IR | IR | IR | IR | IR |
| MSC 42668 | Immature | 585 |  | SM | SE | SE | SE | SE | SE | SM | IR | SE | IR | SE | SE | IR |
| MSC 44473 | Immature | 585 |  | SE | SE | SE | SE | SE | IR | IR | IR | SE | SE | IR | IR | **N** |
| MSC 42661 | Immature | 590 | IR | IR | IR | IR | IR | IR | IR | IR | IR | IR | IR | IR | IR | IR |
| MSC 42665 | Immature | 590 |  | SM | SM | SM | SE | SE | SE | SE | SE | SE | SE | SE | SE | SE |
| MSC 44460 | Immature | 595 |  | SM | SE | SE | SE | SM | SE | SE | SE | SE | SE | SE | SE | SE |
| MSC 44469 | Immature | 600 |  | SM | SE | SM | SE | IR | SE | IR | IR | SE | SE | IR | IR | IR |
| MSC 44476 | Immature | 600 |  | SM | SM | SE | SE | IR | IR | SE | IR | IR | IR | IR | IR | IR |
| MSC 42640 | Immature | 605 |  | SM | SM | SE | SE | SE | SE | SE | SE | SE | SE | SE | SE | IR |
| MSC 42662 | Immature | 605 |  | SE | SE | SE | IR | IR | SM | SM | IR | IR | IR | IR | IR | **N** |
| MSC 42687 | Immature | 605 |  | IR | IR | IR | IR | IR | IR | IR | IR | IR | IR | IR | IR | **N** |
| MSC 44466 | Immature | 605 |  | SM | SE | SE | SE | IR | SE | SE | IR | IR | IR | IR | IR | IR |
| MSC 42663 | Immature | 610 |  | IR | IR | IR | IR | IR | IR | IR | IR | IR | IR | IR | IR | IR |
| MSC 44474 | Immature | 615 |  | SE | SE | SE | SE | IR | SE | SE | SE | SE | SE | SE | IR | IR |
| MSC 42653 | Immature | 620 |  | SM | SM | SM | SE | SE | SE | SE | IR | IR | IR | SM | IR | **N** |
| MSC 42641 | Immature | 635 |  | SM | SE | SM | SE | SM | IR | SE | SE | SE | SE | SE | SE | IR |
| MSC 42657 | Immature | 635 |  | IR | IR | IR | IR | IR | IR | IR | IR | IR | IR | IR | IR | IR |
| MSC 44467 | Immature | 635 | IR | SE | SE | IR | IR | SE | IR | IR | IR | IR | SE | SE | IR | IR |
| MSC 44475 | Immature | 635 |  | SM | SM | SM | SE | SE | IR | SE | IR | SE | SE | IR | SE | IR |
| MSC 42664 | Immature | 640 |  | IR | IR | IR | IR | IR | IR | IR | IR | IR | IR | IR | IR | IR |
| MSC 42650 | Immature | 645 |  | SM | SM | SE | SE | SE | SE | SE | IR | IR | IR | IR | IR | IR |
| MSC 42639 | Immature | 650 |  | SM | SM | SE | SE | SE | SE | IR | IR | IR | IR | IR | SE | **N** |
| MSC 42654 | Immature | 650 |  | IR | IR | IR | IR | IR | IR | IR | IR | IR | IR | IR | IR | IR |
| MSC 42659 | Immature | 650 |  | SM | SE | SE | IR | SE | SE | SE | IR | IR | IR | IR | IR | **N** |
| MSC 44455 | Immature | 650 |  | SM | IR | IR | IR | IR | IR | IR | IR | IR | IR | IR | IR | IR |
| MSC 44468 | Immature | 650 |  | SM | SE | SE | SE | SE | SE | SE | SE | SE | SE | SE | IR | IR |
| MSC 44464 | Immature | 655 |  | SM | SM | SM | IR | IR | IR | IR | IR | IR | IR | IR | IR | IR |
| MSC 44478 | Mature | 660 |  | IR | IR | SE | SE | IR | IR | IR | IR | IR | IR | IR | IR | IR |
| MSC 42647 | Immature | 665 |  | SM | SM | SE | SE | SE | SE | SE | SE | SE | SE | SE | SE | IR |
| MSC 42651 | Immature | 675 |  | IR | IR | IR | IR | IR | IR | IR | IR | IR | IR | IR | IR | **N** |
| MSC 44463 | Immature | 675 |  | SE | SE | SE | SE | SE | SE | SE | SE | SE | SE | IR | IR | SE |
| MSC 42648 | Immature | 680 |  | SE | SE | SE | SE | IR | IR | IR | SE | IR | IR | IR | SE | IR |
| MSC 42686 | Immature | 690 |  | IR | IR | IR | IR | IR | IR | IR | IR | IR | IR | IR | IR | **N** |
| MSC 42682 | Immature | 715 |  | IR | IR | IR | IR | IR | IR | IR | IR | IR | IR | IR | IR | IR |
| MSC 43587 | Immature | 725 |  | SM | SE | SE | SE | SE | SE | SE | SE | SE | IR | IR | SE | **N** |
| MSC 42660 | Immature | 735 |  | IR | IR | IR | IR | IR | IR | IR | IR | IR | IR | IR | IR | IR |
| MSC 42684 | Immature | 735 |  | SM | IR | IR | IR | IR | IR | IR | IR | IR | IR | IR | IR | IR |
| MSC 42667 | Immature | 745 | SE | SM | SE | SE | SE | IR | IR | IR | IR | IR | IR | IR | IR | IR |
| MSC 42637 | Immature | 750 |  | SM | SM | IR | IR | IR | IR | IR | IR | IR | IR | IR | IR | IR |
| MSC 42646 | Immature | 775 |  | IR | IR | IR | IR | IR | IR | IR | IR | IR | IR | IR | IR | IR |
| MSC 42658 | Transitional | 790 |  | IR | IR | IR | IR | IR | IR | IR | IR | IR | IR | IR | IR | IR |
| MSC 43588 | Transitional | 805 |  | SM | SE | SE | SE | SE | SE | SE | SE | SE | SE | SE | SE | IR |
| MSC 43589 | Mature | 815 |  | SM | SM | SM | SE | SE | IR | SE | SE | IR | IR | SE | SE | SE |
| MSC 44479 | Mature | 870 |  | IR | IR | IR | IR | IR | IR | IR | IR | IR | IR | IR | IR | IR |
| MSC 43590 | Transitional | 885 | SE | SE | SE | SE | SE | SE | SE | IR | SE | SE | SE | SE | SE | **N** |
| MSC 42656 | Mature | 895 |  | SE | IR | IR | IR | SE | SE | SE | IR | IR | IR | IR | IR | IR |
| MSC 44454 | Mature | 935 |  | IR | SE | SE | SE | SE | SE | SE | SE | SE | SE | SE | SE | SE |
| MSC 42644 | Mature | 955 |  | SE | IR | IR | SE | SE | SE | SE | IR | SE | SE | SE | IR | IR |
| MSC 46703 | Mature | 964 |  | SM | SM | SM | SM | IR | IR | IR | IR | IR | SM | SE | SM | SE |
| MSC 46736 | Mature | 966 |  | SM | SM | SM | SM | SM | SE | IR | IR | IR | IR | SM | SM | IR |
| MSC 44480 | Mature | 980 | IR | IR | IR | IR | IR | IR | IR | IR | IR | IR | IR | IR | IR | IR |
| MSC 46737 | Mature | 984 |  | IR | SM | SM | IR | IR | IR | IR | IR | IR | IR | IR | IR | SM |
| MSC 44457 | Mature | 990 | IR | IR | IR | IR | IR | IR | IR | IR | IR | IR | SE | SE | SE | SE |
| MSC 46735 | Mature | 994 | SM | SM | SM | SM | SM | IR | IR | IR | IR | IR | IR | SM | SM | SM |
| MSC 42671 | Mature | 1000 | IR | IR | IR | IR | IR | IR | IR | IR | IR | IR | IR | IR | SE | SE |
| MSC 46739 | Mature | 1001 | IR | IR | IR | IR | IR | IR | IR | IR | IR | IR | IR | IR | SM | SM |
| MSC 44458 | Mature | 1010 |  | IR | IR | IR | IR | IR | IR | IR | IR | IR | IR | IR | IR | IR |
| MSC 44456 | Mature | 1033 |  | IR | IR | IR | IR | IR | IR | SE | IR | IR | SE | SE | SE | SE |

**Appendix 129. Male dataset for the nature of the distal cutting edge on the palatoquadrate.** Column header acronyms: **STL** = stretched total length; **Sy** = symphyseal tooth. Descriptive acronyms: **IR** = irregular cutting edge; **SE** = serrated cutting edge; **SM** = smooth cutting edge. Color codes: **Black** = no data; **Dark grey** = serrated cutting edge; **Light grey** = irregular cutting edge; **White** = smooth cutting edge. All pups were *in utero*. Life stage for males based on extent of calcification of the myxopterygia (see Materials & Methods).

**Appendix 1.30: Distal Cutting Edge – Males – Meckel’s Cartilage**

| **Specimen Data** | | | **Meckel's Cartilage Tooth Position** | | | | | | | | | | | | | |
| --- | --- | --- | --- | --- | --- | --- | --- | --- | --- | --- | --- | --- | --- | --- | --- | --- |
| **MSC Number** | **Lifestage** | **STL (mm)** | **12** | **11** | **10** | **9** | **8** | **7** | **6** | **5** | **4** | **3** | **2** | **1** | **LPa** | **RPa** |
| MSC 44470.2 | Pup | 250 |  | SM | SM | SM | SM | IR | SM | IR | IR | IR | IR | IR | IR | IR |
| MSC 44471.2 | Pup | 260 |  | SM | SM | SM | IR | IR | IR | IR | IR | IR | IR | IR |  |  |
| MSC 44471.3 | Pup | 260 |  | SM | SM | SM | IR | IR | IR | IR | IR | IR | IR | IR | IR | IR |
| MSC 44482.2 | Pup | 275 |  | SM | SM | SM | IR | IR | IR | IR | IR | IR | IR | IR | IR | IR |
| MSC 44482.3 | Pup | 280 |  | SM | SM | SM | IR | IR | IR | IR | IR | IR | IR | IR | IR | IR |
| MSC 44482.6 | Pup | 280 |  | SM | SM | IR | IR | IR | IR | IR | IR | IR | IR | IR | IR | IR |
| MSC 44482.7 | Pup | 280 |  | SM | SM | SM | IR | IR | IR | IR | IR | IR | IR | IR | IR | IR |
| MSC 44482.4 | Pup | 290 |  | SM | SM | SM | IR | IR | IR | IR | IR | IR | IR | IR | SM | SM |
| MSC 44482.5 | Pup | 290 |  | SM | SM | SM | IR | IR | IR | IR | IR | IR | IR | IR | IR | IR |
| MSC 44461.6 | Pup | 295 |  | SM | SM | SM | IR | IR | IR | IR | IR | IR | IR | IR | IR | IR |
| MSC 44461.3 | Pup | 300 |  | SM | SM | IR | IR | IR | IR | IR | IR | IR | IR | IR | IR | IR |
| MSC 44472.2 | Pup | 300 |  | SM | SM | IR | IR | IR | IR | IR | IR | IR | IR | IR | IR | IR |
| MSC 44472.7 | Pup | 300 |  | SM | SM | IR | IR | IR | IR | IR | IR | IR | IR | IR | IR |  |
| MSC 44461.5 | Pup | 310 |  | SM | SM | IR | IR | IR | IR | IR | IR | IR | IR | IR | IR | IR |
| MSC 44481.3 | Pup | 310 |  | SM | SM | IR | IR | SM | IR | IR | IR | IR | IR | IR | IR | IR |
| MSC 44472.5 | Pup | 315 |  | SM | SM | SM | IR | IR | IR | IR | IR | IR | IR | IR | IR | IR |
| MSC 44472.6 | Pup | 315 |  | SM | SM | IR | IR | IR | IR | IR | IR | IR | IR | IR | IR | IR |
| MSC 44472.3 | Pup | 320 |  | SM | SM | IR | IR | IR | IR | IR | IR | IR | IR | IR | IR | IR |
| MSC 44481.4 | Pup | 320 |  | SM | SM | SM | SM | IR | IR | IR | IR | IR | IR | IR |  |  |
| MSC 42685.5 | Pup | 330 |  | SM | SM | IR | IR | IR | IR | IR | IR | IR | IR | IR | IR | IR |
| MSC 46739 | Immature | 462 |  | SM | SM | SM | SM | SM | SM | IR | SM | SM | SM | SM | SM | SM |
| MSC 42652 | Immature | 540 |  | IR | IR | IR | IR | IR | IR | IR | IR | IR | IR | IR | IR | IR |
| MSC 44465 | Immature | 540 |  | SM | IR | IR | IR | IR | IR | SE | SE | IR | IR | IR | IR | IR |
| MSC 44477 | Immature | 545 |  | SM | SM | SE | SE | SE | SE | SE | SE | SE | SE | SE | SE |  |
| MSC 42643 | Immature | 565 |  | SE | IR | SE | IR | IR | IR | IR | IR | IR | IR | IR | IR | IR |
| MSC 42668 | Immature | 585 |  | SM | SE | IR | IR | SE | SE | SE | SE | SE | SE | SE | SE | IR |
| MSC 44473 | Immature | 585 |  | SM | SM | SE | SE | IR | SE | SE | SE | SE | IR | IR | IR | IR |
| MSC 42661 | Immature | 590 | SM | IR | IR | IR | IR | IR | IR | IR | IR | IR | IR | IR | IR |  |
| MSC 42665 | Immature | 590 |  | SM | SM | SE | SM | SE | SE | SE | SE | SE | SE | SE | SE | SE |
| MSC 44460 | Immature | 595 |  | SM | SM | SM | SE | SE | SE | SE | SE | SE | SE | SE | IR | IR |
| MSC 44469 | Immature | 600 |  | SM | SE | SE | SE | SE | SE | SE | SE | IR | IR | IR | IR | IR |
| MSC 44476 | Immature | 600 |  | SM | SM | SE | SE | IR | SE | SE | SE | IR | IR | IR | IR | IR |
| MSC 42640 | Immature | 605 |  | SM | SM | SE | SE | SE | SE | SE | SE | SE | SE | SE | IR | SE |
| MSC 42662 | Immature | 605 |  | SM | SE | SE | SE | SE | SE | SE | IR | IR | IR | IR | SE | SE |
| MSC 42687 | Immature | 605 |  | IR | IR | IR | IR | IR | IR | IR | IR | IR | IR | IR | IR | IR |
| MSC 44466 | Immature | 605 |  | SM | SM | SE | SE | SE | SE | SE | SE | SE | SE | IR | IR | IR |
| MSC 42663 | Immature | 610 |  | SM | IR | IR | IR | IR | IR | IR | IR | IR | IR | IR | IR | IR |
| MSC 44474 | Immature | 615 |  | SM | SM | SM | SE | SE | SE | SE | SE | SE | SE | IR | IR | IR |
| MSC 42653 | Immature | 620 |  | SE | SE | SE | SM | IR | SE | SE | IR | IR | IR | IR | IR | IR |
| MSC 42641 | Immature | 635 |  | SM | SM | SM | SE | SM | SM | SE | SE | SE | SE | SM | SM | SE |
| MSC 42657 | Immature | 635 |  | SM | IR | IR | IR | IR | IR | SE | IR | IR | IR | IR | IR | IR |
| MSC 44467 | Immature | 635 |  | SE | SM | SM | SE | SM | IR | IR | SE | SE | IR | SE | SE | IR |
| MSC 44475 | Immature | 635 |  | SM | SM | SE | SE | SE | SE | IR | SE | IR | IR | SM |  | IR |
| MSC 42664 | Immature | 640 |  | IR | IR | IR | IR | IR | IR | IR | IR | IR | IR | IR | IR |  |
| MSC 42650 | Immature | 645 |  | SM | SE | SE | SE | SE | SE | SM | IR | IR | SE | SE | IR | IR |
| MSC 42639 | Immature | 650 | SM | SM | SM | SE | SE | SE | SE | SE | SE | IR | SE | IR | SM |  |
| MSC 42654 | Immature | 650 |  | IR | IR | IR | IR | IR | IR | IR | IR | IR | IR | IR | IR |  |
| MSC 42659 | Immature | 650 |  | SE | SE | SE | SE | SE | SE | SE | SE | SE | SE | SE | SE | SE |
| MSC 44455 | Immature | 650 |  | SM | IR | IR | IR | IR | IR | IR | IR | IR | IR | IR | IR | IR |
| MSC 44468 | Immature | 650 |  | SE | SE | SE | SE | SE | SE | SE | SE | IR | IR | IR | IR | SE |
| MSC 44464 | Immature | 655 |  | SM | SM | IR | IR | IR | IR | IR | IR | IR | IR | IR | IR | IR |
| MSC 44478 | Mature | 660 |  | SM | SM | IR | IR | IR | IR | IR | IR | IR | IR | IR | IR | IR |
| MSC 42647 | Immature | 665 |  | SM | SM | SM | SE | SE | SE | SE | IR | SE | IR | SE | SE | IR |
| MSC 42651 | Immature | 675 |  | IR | IR | IR | IR | IR | IR | IR | IR | IR | IR | IR | IR | IR |
| MSC 44463 | Immature | 675 |  | SM | SM | SE | SE | SE | SE | SE | SE | SE | SE | SE | SE | SE |
| MSC 42648 | Immature | 680 |  | SM | SE | SE | SE | SE | SE | SE | SE | SE | SE | SE | SE | SE |
| MSC 42686 | Immature | 690 |  | SM | IR | IR | IR | IR | IR | IR | IR | IR | IR | IR | IR | IR |
| MSC 42682 | Immature | 715 |  | SM | IR | IR | IR | IR | IR | IR | IR | IR | IR | IR | IR | IR |
| MSC 43587 | Immature | 725 |  | SE | SE | SE | SE | SE | SE | SE | SE | SE | IR | IR | SE | SE |
| MSC 42660 | Immature | 735 |  | SM | IR | IR | IR | IR | IR | IR | IR | IR | IR | IR | IR | IR |
| MSC 42684 | Immature | 735 |  | IR | IR | IR | IR | IR | IR | IR | IR | IR | IR | IR | IR | IR |
| MSC 42667 | Immature | 745 |  | SM | SM | SE | IR | IR | IR | SE | IR | IR | SE | SE | IR | SM |
| MSC 42637 | Immature | 750 |  | SM | SM | IR | IR | IR | IR | IR | IR | IR | IR | IR | IR | IR |
| MSC 42646 | Immature | 775 |  | SE | SE | IR | IR | IR | IR | IR | IR | IR | IR | SM | SE | SE |
| MSC 42658 | Transitional | 790 |  | IR | IR | IR | IR | IR | IR | IR | IR | IR | IR | IR | IR | IR |
| MSC 43588 | Transitional | 805 |  | SM | SM | SM | SM | SE | SE | SE | SE | SE | IR | IR | IR | IR |
| MSC 43589 | Mature | 815 |  | SM | SE | SE | SE | SE | SE | SE | SE | SE | SE | SM* | SM* | SM* |
| MSC 44479 | Mature | 870 |  | IR | IR | IR | IR | IR | IR | IR | IR | IR | IR | SM* | SM* | SM* |
| MSC 43590 | Transitional | 885 | SM | SE | SE | SE | SE | SE | SE | SE | SE | SE | SE | SE | SE | SM* |
| MSC 42656 | Mature | 895 |  | SE | IR | IR | IR | SE | IR | SE | IR | IR | SE | SE | SM* | SM* |
| MSC 44454 | Mature | 935 |  | SE | IR | SE | SE | SE | SE | SE | SE | SE | SM* | SM* | SM* | SM* |
| MSC 42644 | Mature | 955 |  | SM | SE | SE | SE | SE | SE | SE | SE | SE | SM* | SM* | SM* | SM* |
| MSC 46703 | Mature | 964 |  | SM | SM | IR | IR | IR | SE | IR | IR | SE | SE | SE | SM | SM |
| MSC 46736 | Mature | 966 |  | IR | IR | IR | IR | SM | IR | IR | IR | SM | SM | SM* | SM* | SM* |
| MSC 44480 | Mature | 980 |  | SE | IR | IR | IR | IR | SE | IR | IR | IR | SE | SM* | SM* | SM* |
| MSC 46737 | Mature | 984 |  | SM | SM | SM | SM | IR | IR | IR | IR | IR | SM* | SM* | SM* | SM* |
| MSC 44457 | Mature | 990 | SE | SE | IR | IR | IR | IR | SE | SE | IR | IR | SE | SM* | SM* | SM* |
| MSC 46735 | Mature | 994 |  | SM | SM | IR | IR | IR | IR | IR | SM | IR | SM | SM* | SM* | SM* |
| MSC 42671 | Mature | 1000 | SE | SE | IR | IR | IR | IR | IR | IR | IR | IR | IR | SM* | SM* | SM* |
| MSC 46739 | Mature | 1001 |  | SM | SM | SM | SM | IR | IR | IR | IR | IR | SM | SM* | SM* | SM* |
| MSC 44458 | Mature | 1010 |  | IR | IR | IR | SE | SE | IR | IR | IR | IR | SM* | SM* | SM* | SM* |
| MSC 44456 | Mature | 1033 |  | SE | IR | SE | IR | IR | IR | IR | SE | SE | SM* | SM* | SM* | SM* |

**Appendix 130. Male dataset for the nature of the distal cutting edge on the Meckel’s cartilage.** Column header acronyms: **LPa** = Left parasymphyseal tooth. **RPa** = Right parasymphyseal tooth. **STL** = stretched total length. Descriptive acronyms: **IR** = irregular cutting edge; **SE** = serrated cutting edge; **SM** = smooth cutting edge. * = gynandric tooth morphology. Color codes: **Black** = no data; **Dark grey** = serrated cutting edge; **Light grey** = irregular cutting edge; **White** = smooth cutting edge. All pups were *in utero*. Life stage for males based on extent of calcification of the myxopterygia (see Materials & Methods).

**Appendix 1.31: Mesial Cutting Edge – Females – Palatoquadrate**

| **Specimen Data** | | | **Palatoquadrate Tooth Position** | | | | | | | | | | | | | |
| --- | --- | --- | --- | --- | --- | --- | --- | --- | --- | --- | --- | --- | --- | --- | --- | --- |
| **MSC Number** | **Lifestage** | **STL (mm)** | **13** | **12** | **11** | **10** | **9** | **8** | **7** | **6** | **5** | **4** | **3** | **2** | **1** | **Sy** |
| MSC 44471.4 | Pup | 250 |  | SM | IR | IR | IR | IR | IR | IR | IR | IR | IR | IR | IR | SM |
| MSC 44461.2 | Pup | 310 | SM | SM | IR | IR | IR | IR | IR | IR | IR | IR | IR | IR | IR | SM |
| MSC 44461.4 | Pup | 310 |  | SM | SM | IR | IR | IR | IR | IR | IR | IR | IR | IR | IR | IR |
| MSC 44481.5 | Pup | 310 |  | SM | SM | IR | IR | IR | IR | IR | IR | IR | IR | IR | IR | IR |
| MSC 44481.2 | Pup | 320 |  | SM | SM | SM | IR | IR | IR | IR | IR | IR | IR | IR | IR | IR |
| MSC 44472.4 | Pup | 325 |  | SM | SM | IR | IR | IR | IR | IR | IR | IR | IR | IR | IR | IR |
| MSC 42685.7 | Pup | 330 |  | SM | IR | IR | IR | IR | IR | IR | IR | IR | IR | IR | IR | IR |
| MSC 46740 | N/A | 371 |  | SM | SM | IR | IR | IR | IR | IR | IR | IR | IR | IR | IR | SM |
| MSC 46741 | N/A | 378 |  | IR | IR | IR | IR | IR | IR | IR | IR | IR | IR | IR | IR | IR |
| MSC 42649 | N/A | 600 |  | IR | SE | SE | IR | IR | IR | IR | IR | IR | IR | IR | IR | IR |
| MSC 42645 | N/A | 610 |  | IR | IR | IR | IR | IR | IR | IR | IR | IR | IR | IR | IR | IR |
| MSC 43583 | N/A | 615 |  | SE | IR | SE | IR | IR | IR | IR | IR | IR | IR | IR | IR | IR |
| MSC 42655 | N/A | 625 |  | IR | IR | IR | IR | IR | IR | IR | IR | IR | IR | IR | IR | IR |
| MSC 43584 | N/A | 630 |  | IR | IR | IR | IR | IR | IR | IR | IR | IR | IR | IR | IR | IR |
| MSC 43585 | N/A | 635 |  | IR | SE | SE | IR | IR | SE | SE | SE | SE | IR | IR | SE | IR |
| MSC 43586 | N/A | 646 | IR | IR | IR | IR | IR | IR | IR | IR | IR | IR | IR | IR | IR |  |
| MSC 42638 | N/A | 675 |  | IR | IR | IR | IR | IR | IR | IR | IR | IR | IR | IR | IR |  |
| MSC 44471.1 | Mature | 862 |  | IR | IR | IR | IR | IR | IR | IR | IR | IR | IR | IR | IR | IR |
| MSC 44462 | N/A | 879 |  | IR | IR | IR | IR | IR | SE | IR | IR | IR | SE | IR | IR | IR |
| MSC 42685.1 | Mature | 895 | IR | IR | SE | IR | IR | IR | IR | SE | IR | IR | IR | IR | SE | SE |
| MSC 44459 | N/A | 899 |  | IR | IR | IR | SE | IR | SE | IR | IR | IR | IR | IR | IR | IR |
| MSC 44470.1 | Mature | 922 |  | IR | IR | IR | IR | IR | IR | IR | IR | IR | IR | IR | IR |  |
| MSC 44482.1 | Mature | 940 |  | IR | IR | IR | IR | IR | IR | IR | IR | IR | IR | IR | IR | IR |
| MSC 42666 | Mature | 960 |  | SE | SE | SE | SE | SE | IR | IR | IR | IR | IR | IR | SE | SE |
| MSC 42683 | Mature | 960 |  | SE | IR | IR | IR | SE | SE | SE | SE | IR | IR | IR | SE | SE |
| MSC 42678 | Mature | 966 |  | SE | SE | SE | IR | IR | IR | IR | IR | IR | IR | IR | SE | SE |
| MSC 42673 | Mature | 977 |  | IR | IR | IR | IR | IR | IR | IR | IR | IR | IR | IR | IR |  |
| MSC 44481.1 | Mature | 980 |  | IR | IR | IR | IR | IR | IR | IR | IR | IR | IR | IR | IR | IR |
| MSC 44461.1 | Mature | 991 |  | IR | IR | IR | IR | IR | IR | IR | IR | IR | IR | IR | IR | IR |
| MSC 42681 | Mature | 997 |  | IR | IR | IR | IR | IR | IR | IR | IR | IR | IR | IR | IR | IR |
| MSC 42670 | Mature | 1001 |  | IR | IR | IR | IR | IR | IR | IR | IR | IR | IR | IR | SE | SE |
| MSC 42672 | Mature | 1002 |  | IR | IR | IR | IR | IR | IR | IR | IR | IR | IR | IR | IR | IR |
| MSC 42679 | Mature | 1007 |  | SE | SE | SE | SE | IR | IR | SE | IR | SE | IR | IR | IR | IR |
| MSC 42669 | Mature | 1011 |  | IR | IR | IR | IR | IR | IR | IR | IR | IR | IR | IR | IR | IR |
| MSC 44472.1 | Mature | 1021 |  | IR | IR | IR | IR | IR | IR | IR | IR | IR | IR | IR | IR | IR |
| MSC 42677 | Mature | 1025 |  | IR | IR | IR | IR | IR | IR | IR | IR | IR | IR | IR | SE | IR |
| MSC 42680 | Mature | 1025 |  | IR | IR | IR | IR | IR | IR | IR | IR | IR | IR | IR | IR | IR |
| MSC 42676 | Mature | 1030 |  | SE | IR | IR | IR | IR | IR | IR | IR | IR | IR | IR | IR | IR |
| MSC 42674 | Mature | 1040 |  | IR | IR | SE | IR | IR | IR | IR | IR | IR | IR | IR | IR | IR |
| MSC 42675 | Mature | 1043 |  | IR | IR | IR | IR | IR | IR | IR | IR | IR | IR | IR | IR | IR |

**Appendix 1.31. Female dataset for the nature of the mesial cutting edge on the palatoquadrate.** Column header acronyms: **STL** = stretched total length; **Sy** = symphyseal tooth. Descriptive acronyms: **IR** = irregular cutting edge; **SE** = serrated cutting edge; **SM** = smooth cutting edge. Color codes: **Black** = no data; **Dark grey** = serrated cutting edge; **Light grey** = irregular cutting edge; **White** = smooth cutting edge. All pups were *in utero*. Sexual maturity for females based on the presence of pups.

**Appendix 1.32: Mesial Cutting Edge – Females – Meckel’s Cartilage**

| **Specimen Data** | | | **Meckel's Cartilage Tooth Position** | | | | | | | | | | | | | |
| --- | --- | --- | --- | --- | --- | --- | --- | --- | --- | --- | --- | --- | --- | --- | --- | --- |
| **MSC Number** | **Lifestage** | **STL (mm)** | **12** | **11** | **10** | **9** | **8** | **7** | **6** | **5** | **4** | **3** | **2** | **1** | **LPa** | **RPa** |
| MSC 44471.4 | Pup | 250 |  | SM | SM | SM | IR | IR | IR | IR | IR | IR | IR | IR | IR | IR |
| MSC 44461.2 | Pup | 310 |  | SM | IR | IR | IR | IR | IR | IR | IR | IR | IR | IR |  |  |
| MSC 44461.4 | Pup | 310 |  | SM | SM | IR | IR | IR | IR | IR | IR | IR | IR | IR | IR | IR |
| MSC 44481.5 | Pup | 310 |  | SM | SM | SM | IR | IR | IR | IR | IR | SM | IR | IR | IR | IR |
| MSC 44481.2 | Pup | 320 |  | SM | SM | IR | IR | IR | IR | IR | IR | IR | IR | IR | IR | SM |
| MSC 44472.4 | Pup | 325 |  | SM | SM | IR | IR | IR | IR | IR | IR | IR | IR | IR | IR | IR |
| MSC 42685.7 | Pup | 330 |  | SM | SM | SM | IR | IR | IR | IR | IR | SM | SM | IR | SM | SM |
| MSC 46740 | N/A | 371 |  | IR | IR | IR | IR | IR | IR | IR | IR | IR | IR | IR | IR | IR |
| MSC 46741 | N/A | 378 |  | SM | SM | IR | IR | IR | IR | IR | IR | IR | IR | IR | IR | IR |
| MSC 42649 | N/A | 600 |  | SE | SE | IR | SM | SE | SE | SE | IR | IR | SE | IR | SE | SE |
| MSC 42645 | N/A | 610 |  | IR | IR | IR | IR | IR | IR | IR | IR | IR | IR | IR | IR | IR |
| MSC 43583 | N/A | 615 |  |  | SE | IR | SE | SE | SE | SE | SE | SE | IR | IR | IR | IR |
| MSC 42655 | N/A | 625 |  | IR | IR | IR | IR | IR | IR | IR | IR | IR | IR | IR |  | IR |
| MSC 43584 | N/A | 630 |  | IR | IR | IR | IR | IR | IR | IR | IR | IR | IR | IR | IR | IR |
| MSC 43585 | N/A | 635 |  | SM | SE | SE | SE | SE | SE | SE | SE | SE | SE | SE | IR | IR |
| MSC 43586 | N/A | 646 |  | IR | IR | IR | IR | IR | IR | IR | SE | IR | IR | IR | SE |  |
| MSC 42638 | N/A | 675 |  | IR | IR | IR | IR | IR | IR | IR | IR | IR | IR | IR | IR | IR |
| MSC 44471.1 | Mature | 862 |  | IR | IR | IR | IR | IR | IR | IR | IR | IR | IR | IR | IR | IR |
| MSC 44462 | N/A | 879 |  | SE | SE | SE | SE | IR | IR | IR | IR | IR | SE | IR | IR | IR |
| MSC 42685.1 | Mature | 895 |  | SE | IR | IR | IR | IR | IR | IR | IR | IR | IR | IR | SE | SE |
| MSC 44459 | N/A | 899 |  | SE | IR | SE | IR | SE | IR | IR | IR | IR | IR | SM | SM | SM |
| MSC 44470.1 | Mature | 922 |  | IR | IR | IR | IR | IR | IR | IR | IR | IR | IR | IR | IR | IR |
| MSC 44482.1 | Mature | 940 |  | IR | IR | IR | IR | IR | IR | IR | IR | IR | IR | IR | IR | IR |
| MSC 42666 | Mature | 960 |  | SE | SE | IR | SE | SE | SE | IR | SE | SE | SE | SE | SE | SE |
| MSC 42683 | Mature | 960 | SE | SE | IR | IR | IR | IR | IR | IR | IR | IR | SE | SE | SE | SE |
| MSC 42678 | Mature | 966 |  | IR | IR | SE | SE | IR | IR | IR | IR | IR | SE | SE | IR | IR |
| MSC 42673 | Mature | 977 |  | IR | IR | IR | IR | IR | IR | IR | IR | IR | IR | IR | IR | IR |
| MSC 44481.1 | Mature | 980 |  | IR | IR | IR | IR | IR | IR | IR | IR | IR | IR | IR | IR | IR |
| MSC 44461.1 | Mature | 991 |  | IR | IR | IR | IR | IR | IR | IR | IR | IR | IR | IR | IR | IR |
| MSC 42681 | Mature | 997 |  | IR | IR | IR | IR | IR | IR | IR | IR | IR | IR | SE | IR | SE |
| MSC 42670 | Mature | 1001 |  | SE | SE | SE | SE | SE | IR | IR | IR | SE | IR | SE | SE | SE |
| MSC 42672 | Mature | 1002 |  | IR | IR | IR | IR | IR | IR | IR | IR | IR | IR | IR | IR | IR |
| MSC 42679 | Mature | 1007 |  | SE | SE | IR | IR | IR | SE | SE | IR | SE | SE | SE | SE | SE |
| MSC 42669 | Mature | 1011 |  | IR | IR | IR | IR | IR | IR | IR | IR | IR | IR | IR | IR | IR |
| MSC 44472.1 | Mature | 1021 |  | IR | IR | IR | IR | IR | IR | IR | IR | IR | IR | IR | IR | IR |
| MSC 42677 | Mature | 1025 |  | IR | IR | IR | IR | IR | IR | IR | IR | IR | IR | IR | IR | SE |
| MSC 42680 | Mature | 1025 |  | SE | SE | SE | IR | IR | IR | IR | IR | IR | IR | IR | IR | IR |
| MSC 42676 | Mature | 1030 |  | IR | IR | IR | IR | IR | IR | IR | IR | IR | IR | IR | IR | IR |
| MSC 42674 | Mature | 1040 | SE | SE | IR | SE | IR | IR | IR | IR | IR | IR | IR | SE | IR | IR |
| MSC 42675 | Mature | 1043 |  | IR | IR | IR | IR | IR | IR | IR | IR | IR | IR | IR | IR | IR |

**Appendix 1.32. Female dataset for the nature of the mesial cutting edge on the Meckel’s cartilage.** Column header acronyms: **LPa** = left parasymphyseal tooth; **RPa** = right parasymphyseal tooth; **STL** = stretched total length. Descriptive acronyms: **IR** = irregular cutting edge; **SE** = serrated cutting edge; **SM** = smooth cutting edge. Color codes: **Black** = no data; **Dark grey** = serrated cutting edge; **Light grey** = irregular cutting edge; **White** = smooth cutting edge. All pups were *in utero*. Sexual maturity for females based on the presence of pups.

**Appendix 1.33: Mesial Cutting Edge – Males – Palatoquadrate**

| **Specimen Data** | | | **Palatoquadrate Tooth Position** | | | | | | | | | | | | | |
| --- | --- | --- | --- | --- | --- | --- | --- | --- | --- | --- | --- | --- | --- | --- | --- | --- |
| **MSC Number** | **Lifestage** | **STL (mm)** | **13** | **12** | **11** | **10** | **9** | **8** | **7** | **6** | **5** | **4** | **3** | **2** | **1** | **Sy** |
| MSC 44470.2 | Pup | 250 |  | SM | IR | IR | IR | IR | IR | IR | IR | IR | IR | IR | IR | IR |
| MSC 44471.2 | Pup | 260 |  | SM | SM | SM | IR | IR | IR | IR | IR | IR | IR | IR | IR |  |
| MSC 44471.3 | Pup | 260 |  | SM | SM | IR | IR | IR | IR | IR | IR | IR | IR | IR | IR | IR |
| MSC 44482.2 | Pup | 275 |  | SM | SM | IR | IR | IR | IR | IR | IR | IR | IR | IR | IR | IR |
| MSC 44482.3 | Pup | 280 |  | SM | SM | SM | IR | IR | IR | IR | IR | IR | IR | IR | IR | IR |
| MSC 44482.6 | Pup | 280 |  | SM | SM | SM | IR | IR | IR | IR | IR | IR | IR | IR | IR | IR |
| MSC 44482.7 | Pup | 280 |  | SM | SM | SM | IR | IR | IR | IR | IR | IR | IR | IR | IR | SM |
| MSC 44482.4 | Pup | 290 |  | SM | SM | SM | IR | IR | IR | IR | IR | IR | IR | IR | IR | SM |
| MSC 44482.5 | Pup | 290 |  | SM | SM | IR | IR | IR | IR | IR | IR | IR | IR | IR | IR | IR |
| MSC 44461.6 | Pup | 295 |  | SM | SM | IR | IR | IR | IR | IR | IR | IR | IR | SM | SM | SM |
| MSC 44461.3 | Pup | 300 |  | SM | SM | IR | IR | IR | IR | IR | IR | IR | IR | IR | IR | IR |
| MSC 44472.2 | Pup | 300 |  | SM | SM | IR | IR | IR | IR | IR | IR | IR | IR | IR | IR | IR |
| MSC 44472.7 | Pup | 300 |  | SM | SM | IR | IR | IR | IR | IR | IR | IR | IR | IR | IR | IR |
| MSC 44461.5 | Pup | 310 |  | IR | IR | IR | IR | IR | IR | IR | IR | IR | IR | IR | IR | IR |
| MSC 44481.3 | Pup | 310 |  | SM | SM | SM | IR | IR | IR | IR | IR | IR | IR | SM | IR | SM |
| MSC 44472.5 | Pup | 315 |  | SM | SM | IR | IR | IR | IR | IR | IR | IR | IR | IR | IR | SM |
| MSC 44472.6 | Pup | 315 |  | IR | SM | IR | IR | IR | IR | IR | IR | IR | IR | IR | IR | IR |
| MSC 44472.3 | Pup | 320 |  | SM | IR | IR | IR | IR | IR | IR | IR | IR | IR | IR | IR | IR |
| MSC 44481.4 | Pup | 320 |  | SM | IR | IR | IR | IR | IR | IR | IR | IR | IR | IR | IR | IR |
| MSC 42685.5 | Pup | 330 |  | SM | SM | IR | IR | IR | IR | IR | IR | IR | IR | IR | SM | IR |
| MSC 46739 | Immature | 462 |  | IR | IR | IR | IR | IR | IR | IR | IR | IR | IR | IR | IR | IR |
| MSC 42652 | Immature | 540 |  | IR | IR | SE | IR | IR | IR | IR | IR | IR | IR | IR | IR | IR |
| MSC 44465 | Immature | 540 |  | IR | SE | IR | IR | IR | IR | IR | IR | IR | IR | IR | IR | IR |
| MSC 44477 | Immature | 545 |  |  | IR | IR | IR | IR | IR | IR | IR | IR | IR | SE | SE |  |
| MSC 42643 | Immature | 565 |  | IR | IR | IR | IR | IR | IR | IR | IR | IR | IR | IR | IR | IR |
| MSC 42668 | Immature | 585 |  | SE | SE | SE | SE | IR | SE | IR | IR | IR | IR | IR | SE | IR |
| MSC 44473 | Immature | 585 |  | IR | SE | SE | SE | IR | IR | IR | IR | IR | IR | IR | IR |  |
| MSC 42661 | Immature | 590 | IR | IR | IR | IR | IR | IR | IR | IR | IR | IR | IR | IR | IR | IR |
| MSC 42665 | Immature | 590 |  | IR | SE | IR | SE | IR | SE | IR | IR | SE | IR | SE | SE | SE |
| MSC 44460 | Immature | 595 |  | SE | SM | IR | IR | SE | IR | IR | SE | IR | IR | IR | SE | SE |
| MSC 44469 | Immature | 600 |  | SM | SE | IR | IR | IR | IR | IR | IR | IR | IR | IR | IR | IR |
| MSC 44476 | Immature | 600 |  | IR | IR | IR | IR | IR | IR | IR | IR | IR | IR | IR | IR | IR |
| MSC 42640 | Immature | 605 |  | SE | SM | SE | SE | IR | IR | IR | IR | SE | SE | SE | SE | IR |
| MSC 42662 | Immature | 605 |  | IR | IR | IR | IR | IR | IR | IR | IR | IR | IR | IR | IR |  |
| MSC 42687 | Immature | 605 |  | IR | IR | IR | IR | IR | IR | IR | IR | IR | IR | IR | IR |  |
| MSC 44466 | Immature | 605 |  | IR | SE | SE | IR | IR | IR | IR | IR | IR | IR | IR | IR | IR |
| MSC 42663 | Immature | 610 |  | IR | IR | IR | IR | IR | IR | IR | IR | IR | IR | IR | IR | IR |
| MSC 44474 | Immature | 615 |  | SE | IR | IR | SE | IR | SE | SE | IR | SE | SE | SE | IR | IR |
| MSC 42653 | Immature | 620 |  | SM | IR | SE | SE | IR | IR | IR | IR | IR | IR | IR | IR | IR |
| MSC 42641 | Immature | 635 |  | SE | SE | SE | IR | SE | IR | IR | IR | SE | SE | SE | SE | IR |
| MSC 42657 | Immature | 635 |  | IR | IR | IR | IR | IR | IR | IR | IR | IR | IR | IR | IR | IR |
| MSC 44467 | Immature | 635 | IR | SE | IR | IR | IR | IR | IR | IR | IR | IR | IR | IR | IR | IR |
| MSC 44475 | Immature | 635 |  | SE | IR | IR | IR | SE | IR | SM | IR | SM | IR | IR | IR | IR |
| MSC 42664 | Immature | 640 |  | IR | IR | IR | IR | IR | IR | IR | IR | IR | IR | IR | IR | IR |
| MSC 42650 | Immature | 645 |  | IR | SM | IR | IR | SE | IR | SE | IR | IR | IR | IR | IR | IR |
| MSC 42639 | Immature | 650 |  | SE | IR | IR | SE | SE | SE | IR | IR | IR | IR | IR | SE |  |
| MSC 42654 | Immature | 650 |  | IR | IR | IR | IR | IR | IR | IR | IR | IR | IR | IR | IR | IR |
| MSC 42659 | Immature | 650 |  | SE | SE | SE | IR | SE | IR | SE | IR | IR | IR | IR | IR |  |
| MSC 44455 | Immature | 650 |  | IR | IR | IR | IR | IR | IR | IR | IR | IR | IR | IR | IR | IR |
| MSC 44468 | Immature | 650 |  | IR | SE | SE | IR | IR | IR | IR | IR | IR | IR | IR | IR | IR |
| MSC 44464 | Immature | 655 |  | SM | IR | IR | IR | IR | IR | IR | IR | IR | IR | IR | IR | IR |
| MSC 44478 | Mature | 660 |  | IR | IR | IR | IR | IR | IR | IR | IR | IR | IR | IR | IR | IR |
| MSC 42647 | Immature | 665 |  | SE | IR | SE | IR | SE | IR | SE | IR | SE | IR | IR | IR | IR |
| MSC 42651 | Immature | 675 |  | IR | IR | IR | IR | IR | IR | IR | IR | IR | IR | IR | IR | IR |
| MSC 44463 | Immature | 675 |  | SE | IR | SE | SE | SE | IR | SE | SE | SE | SE | IR | IR | SE |
| MSC 42648 | Immature | 680 |  | IR | IR | IR | IR | IR | IR | IR | IR | IR | IR | IR | IR | IR |
| MSC 42686 | Immature | 690 |  | IR | IR | IR | IR | IR | IR | IR | IR | IR | IR | IR | IR |  |
| MSC 42682 | Immature | 715 |  | IR | IR | IR | IR | IR | IR | IR | IR | IR | IR | IR | IR |  |
| MSC 43587 | Immature | 725 |  | SE | SE | IR | SE | SE | SE | IR | SE | IR | IR | IR | SE | IR |
| MSC 42660 | Immature | 735 |  | IR | IR | IR | IR | IR | IR | IR | IR | IR | IR | IR | IR | IR |
| MSC 42684 | Immature | 735 |  | IR | IR | IR | IR | IR | IR | IR | IR | IR | IR | IR | IR | IR |
| MSC 42667 | Immature | 745 | SE | IR | IR | IR | IR | IR | IR | IR | IR | IR | IR | IR | IR | IR |
| MSC 42637 | Immature | 750 |  | IR | IR | IR | IR | IR | IR | IR | IR | IR | IR | IR | IR | IR |
| MSC 42646 | Immature | 775 |  | IR | IR | IR | IR | IR | IR | IR | IR | IR | IR | IR | IR | IR |
| MSC 42658 | Transitional | 790 |  | IR | IR | IR | IR | IR | IR | IR | IR | IR | IR | IR | IR | IR |
| MSC 43588 | Transitional | 805 |  | SE | SE | SE | IR | IR | SE | IR | SE | IR | IR | SE | SE | IR |
| MSC 43589 | Mature | 815 |  | SE | SE | SE | IR | SE | IR | IR | IR | IR | IR | SE | SE | SE |
| MSC 44479 | Mature | 870 |  | IR | IR | IR | IR | IR | IR | IR | IR | IR | IR | IR | IR | IR |
| MSC 43590 | Transitional | 885 | IR | IR | IR | IR | IR | IR | SE | IR | IR | SE | SE | SE | SE |  |
| MSC 42656 | Mature | 895 |  | SE | IR | IR | IR | IR | SE | IR | IR | IR | IR | IR | IR | IR |
| MSC 44454 | Mature | 935 |  | SE | SE | SE | SE | IR | IR | SE | SE | SE | SE | SE | SE | SE |
| MSC 42644 | Mature | 955 |  | SE | IR | IR | IR | SE | IR | SE | IR | IR | IR | IR | IR | IR |
| MSC 46703 | Mature | 964 |  | SM | IR | IR | SE | IR | IR | SE | SE | SE | SE | IR | IR | SE |
| MSC 46736 | Mature | 966 |  | SM | IR | SE | IR | IR | SE | SE | SE | SE | SE | IR | IR | SM |
| MSC 44480 | Mature | 980 | IR | IR | IR | IR | IR | IR | IR | IR | IR | IR | IR | IR | IR | IR |
| MSC 46737 | Mature | 984 |  | IR | IR | IR | IR | SE | SE | SE | SE | SE | SE | IR | IR | SM |
| MSC 44457 | Mature | 990 | IR | IR | IR | IR | IR | IR | IR | IR | IR | IR | SE | SE | SE | SE |
| MSC 46735 | Mature | 994 | IR | IR | SM | SM | IR | IR | IR | SE | SE | SE | IR | SM | SM | SM |
| MSC 42671 | Mature | 1000 | IR | IR | IR | IR | IR | IR | IR | IR | IR | IR | IR | IR | SE | SE |
| MSC 46739 | Mature | 1001 | IR | IR | IR | IR | IR | SE | SE | SE | SE | SE | SE | IR | IR | IR |
| MSC 44458 | Mature | 1010 |  | IR | IR | IR | IR | IR | IR | IR | IR | IR | IR | IR | IR | IR |
| MSC 44456 | Mature | 1033 |  | IR | IR | IR | IR | IR | IR | SE | IR | IR | SE | SE | SE | SE |

**Appendix 1.33. Male dataset for the nature of the mesial cutting edge on the palatoquadrate.** Column header acronyms: **Sy** = symphyseal tooth; **STL** = stretched total length. Descriptive acronyms: **IR** = irregular cutting edge; **SE** = serrated cutting edge; **SM** = smooth cutting edge. Color codes: **Black** = no data; **Dark grey** = serrated cutting edge; **Light grey** = irregular cutting edge; **White** = smooth cutting edge. All pups were *in utero*. Life stage for males based on extent of calcification of the myxopterygia (see Materials & Methods).

**Appendix 1.34: Mesial Cutting Edge – Males – Meckel’s Cartilage**

| **Specimen Data** | | | **Meckel's Cartilage Tooth Position** | | | | | | | | | | | | | |
| --- | --- | --- | --- | --- | --- | --- | --- | --- | --- | --- | --- | --- | --- | --- | --- | --- |
| **MSC Number** | **Lifestage** | **STL (mm)** | **12** | **11** | **10** | **9** | **8** | **7** | **6** | **5** | **4** | **3** | **2** | **1** | **LPa** | **RPa** |
| MSC 44470.2 | Pup | 250 |  | SM | SM | SM | IR | IR | IR | IR | IR | IR | IR | IR | SM | SM |
| MSC 44471.2 | Pup | 260 |  | SM | SM | SM | IR | IR | IR | IR | IR | IR | IR | IR |  |  |
| MSC 44471.3 | Pup | 260 |  | SM | SM | SM | IR | IR | IR | IR | IR | IR | IR | IR | IR | IR |
| MSC 44482.2 | Pup | 275 |  | SM | SM | SM | IR | IR | IR | IR | IR | IR | IR | IR | IR | IR |
| MSC 44482.3 | Pup | 280 |  | SM | SM | SM | IR | IR | IR | IR | IR | IR | IR | IR | SM | IR |
| MSC 44482.6 | Pup | 280 |  | SM | SM | IR | IR | IR | IR | IR | IR | IR | IR | IR | SM | SM |
| MSC 44482.7 | Pup | 280 |  | SM | SM | SM | IR | IR | IR | IR | IR | IR | IR | IR | SM | SM |
| MSC 44482.4 | Pup | 290 |  | SM | SM | SM | IR | IR | IR | IR | IR | IR | IR | IR | SM | SM |
| MSC 44482.5 | Pup | 290 |  | SM | SM | SM | IR | IR | IR | IR | IR | IR | IR | IR | IR | IR |
| MSC 44461.6 | Pup | 295 |  | SM | SM | SM | IR | IR | IR | IR | IR | IR | IR | IR | IR | IR |
| MSC 44461.3 | Pup | 300 |  | SM | SM | IR | IR | IR | IR | IR | IR | IR | IR | IR | IR | IR |
| MSC 44472.2 | Pup | 300 |  | SM | SM | IR | IR | IR | IR | IR | IR | IR | IR | IR | IR | IR |
| MSC 44472.7 | Pup | 300 |  | SM | SM | IR | IR | IR | IR | IR | IR | IR | IR | IR | IR | IR |
| MSC 44461.5 | Pup | 310 |  | SM | SM | IR | IR | IR | IR | IR | IR | IR | IR | IR | IR |  |
| MSC 44481.3 | Pup | 310 |  | SM | SM | IR | IR | IR | IR | IR | IR | IR | IR | IR |  | IR |
| MSC 44472.5 | Pup | 315 |  | SM | SM | SM | IR | IR | IR | IR | IR | IR | IR | IR | IR | IR |
| MSC 44472.6 | Pup | 315 |  | SM | SM | IR | IR | IR | IR | IR | IR | IR | IR | IR | IR | IR |
| MSC 44472.3 | Pup | 320 |  | SM | SM | IR | IR | IR | IR | IR | IR | IR | IR | IR | IR | IR |
| MSC 44481.4 | Pup | 320 |  | SM | SM | SM | SM | IR | IR | IR | IR | IR | IR | IR |  |  |
| MSC 42685.5 | Pup | 330 |  | SM | SM | IR | IR | IR | IR | IR | IR | IR | IR | IR | IR | IR |
| MSC 46739 | Immature | 462 |  | IR | IR | IR | IR | IR | IR | IR | IR | IR | IR | SM | SM | SM |
| MSC 42652 | Immature | 540 |  | IR | IR | IR | IR | IR | IR | IR | IR | IR | IR | IR | IR | IR |
| MSC 44465 | Immature | 540 |  | IR | IR | IR | IR | IR | IR | IR | IR | IR | IR | IR | IR | IR |
| MSC 44477 | Immature | 545 |  | SE | SE | IR | IR | SE | IR | IR | IR | IR | IR | SE | SE |  |
| MSC 42643 | Immature | 565 |  | IR | IR | IR | IR | IR | IR | IR | IR | IR | IR | IR | IR | IR |
| MSC 42668 | Immature | 585 |  | IR | IR | IR | IR | IR | IR | IR | IR | IR | IR | SE | SE | IR |
| MSC 44473 | Immature | 585 |  | IR | IR | SE | SE | IR | IR | IR | IR | IR | IR | IR | IR | IR |
| MSC 42661 | Immature | 590 | IR | IR | IR | IR | IR | IR | IR | IR | IR | IR | IR | IR | IR |  |
| MSC 42665 | Immature | 590 |  | SE | SE | SE | SE | IR | IR | IR | SE | SE | SE | SE | SE | SE |
| MSC 44460 | Immature | 595 |  | SE | SE | SE | SE | SE | IR | IR | IR | IR | IR | SE | IR | IR |
| MSC 44469 | Immature | 600 |  | SM | SE | IR | IR | IR | IR | IR | SE | IR | IR | IR | IR | IR |
| MSC 44476 | Immature | 600 |  | IR | IR | IR | IR | IR | IR | IR | IR | IR | IR | IR | IR | IR |
| MSC 42640 | Immature | 605 |  | SE | SE | SE | SE | IR | IR | IR | SE | SE | SE | SE | SE | IR |
| MSC 42662 | Immature | 605 |  | IR | SE | SE | SE | IR | IR | IR | IR | IR | IR | IR | IR | IR |
| MSC 42687 | Immature | 605 |  | IR | IR | IR | IR | IR | IR | IR | IR | IR | IR | IR | IR | IR |
| MSC 44466 | Immature | 605 |  | SE | SE | SE | SE | IR | IR | IR | IR | IR | IR | IR | IR | IR |
| MSC 42663 | Immature | 610 |  | IR | IR | IR | IR | IR | IR | IR | IR | IR | IR | IR | IR | IR |
| MSC 44474 | Immature | 615 |  | SE | SE | SE | SE | SE | IR | IR | IR | IR | IR | IR | IR | IR |
| MSC 42653 | Immature | 620 |  | IR | IR | IR | IR | IR | SE | SE | IR | IR | IR | IR | IR | IR |
| MSC 42641 | Immature | 635 |  | IR | SE | IR | SE | IR | IR | SE | IR | SE | IR | SM | SM | SE |
| MSC 42657 | Immature | 635 |  | IR | IR | IR | IR | IR | IR | IR | IR | IR | IR | IR | IR | IR |
| MSC 44467 | Immature | 635 |  | IR | IR | IR | IR | IR | IR | IR | IR | IR | IR | SE | SE | IR |
| MSC 44475 | Immature | 635 |  | SM | SM | IR | SE | IR | IR | IR | IR | IR | IR | IR |  | IR |
| MSC 42664 | Immature | 640 |  | IR | IR | IR | IR | IR | IR | IR | IR | IR | IR | IR | IR | IR |
| MSC 42650 | Immature | 645 |  | SE | SE | SE | IR | IR | SE | IR | IR | IR | SE | IR | IR | IR |
| MSC 42639 | Immature | 650 | SM | SM | SM | IR | SE | IR | IR | SE | SE | IR | IR | IR | SM |  |
| MSC 42654 | Immature | 650 |  | IR | IR | IR | IR | IR | IR | IR | IR | IR | IR | IR | IR | IR |
| MSC 42659 | Immature | 650 |  | SE | IR | SE | IR | IR | IR | SE | SE | SE | SE | SE | SE | SE |
| MSC 44455 | Immature | 650 |  | SM | IR | IR | IR | IR | IR | IR | IR | IR | IR | IR | IR | IR |
| MSC 44468 | Immature | 650 |  | SE | SE | SE | SE | IR | SE | IR | IR | IR | IR | SM | IR | IR |
| MSC 44464 | Immature | 655 |  | IR | IR | IR | IR | IR | IR | IR | IR | IR | IR | IR | IR | IR |
| MSC 44478 | Mature | 660 |  | IR | IR | IR | IR | IR | IR | IR | IR | IR | IR | IR | IR | IR |
| MSC 42647 | Immature | 665 |  | SE | SE | SE | SE | SE | IR | IR | IR | IR | IR | SE | SE | IR |
| MSC 42651 | Immature | 675 |  | IR | IR | IR | IR | IR | IR | IR | IR | IR | IR | IR | IR | IR |
| MSC 44463 | Immature | 675 |  | SE | SE | SE | SE | SE | IR | IR | SE | IR | IR | IR | SE | SE |
| MSC 42648 | Immature | 680 |  | SE | SE | IR | SE | SE | IR | IR | IR | IR | SE | IR | SE | IR |
| MSC 42686 | Immature | 690 |  | IR | IR | IR | IR | IR | IR | IR | IR | IR | IR | IR | IR | IR |
| MSC 42682 | Immature | 715 |  | IR | IR | IR | IR | IR | IR | IR | IR | IR | IR | IR | IR | IR |
| MSC 43587 | Immature | 725 |  | IR | IR | SE | IR | IR | SE | IR | IR | IR | IR | IR | SE | SE |
| MSC 42660 | Immature | 735 |  | IR | IR | IR | IR | IR | IR | IR | IR | IR | IR | IR | IR | IR |
| MSC 42684 | Immature | 735 |  | IR | IR | IR | IR | IR | IR | IR | IR | IR | IR | IR | IR | IR |
| MSC 42667 | Immature | 745 |  | IR | IR | IR | IR | IR | IR | IR | IR | IR | IR | IR | IR | SM |
| MSC 42637 | Immature | 750 |  | IR | IR | IR | IR | IR | IR | IR | IR | IR | IR | IR | IR | IR |
| MSC 42646 | Immature | 775 |  | IR | IR | IR | IR | IR | IR | IR | IR | IR | IR | SE | SE | SE |
| MSC 42658 | Transitional | 790 |  | IR | IR | IR | IR | IR | IR | IR | IR | IR | IR | IR | IR | IR |
| MSC 43588 | Transitional | 805 |  | SE | SE | SE | IR | IR | IR | IR | IR | IR | IR | IR | IR | IR |
| MSC 43589 | Mature | 815 |  | SE | IR | IR | SE | SE | SE | IR | SE | IR | SE | SM* | SM* | SM* |
| MSC 44479 | Mature | 870 |  | IR | IR | IR | IR | IR | IR | IR | IR | IR | IR | SM* | SM* | SM* |
| MSC 43590 | Transitional | 885 | SE | SE | IR | SE | IR | IR | IR | IR | IR | SE | SE | SE | SE | SM* |
| MSC 42656 | Mature | 895 |  | IR | IR | IR | IR | IR | IR | IR | IR | IR | SE | SE | SM* | SM* |
| MSC 44454 | Mature | 935 |  | SE | SE | IR | SE | SE | SE | SE | SE | SE | SM* | SM* | SM* | SM* |
| MSC 42644 | Mature | 955 |  | SE | SE | IR | IR | IR | SE | SE | SE | SE | SM* | SM* | SM* | SM* |
| MSC 46703 | Mature | 964 |  | SM | IR | IR | IR | IR | SE | IR | IR | SE | IR | IR | IR | IR |
| MSC 46736 | Mature | 966 |  | IR | IR | IR | IR | IR | IR | IR | SE | IR | IR | SM* | SM* | SM* |
| MSC 44480 | Mature | 980 |  | SE | IR | IR | IR | IR | SE | IR | IR | IR | SE | SM* | SM* | SM* |
| MSC 46737 | Mature | 984 |  | SM | IR | IR | IR | SE | SE | SE | IR | IR | SM | SM* | SM* | SM* |
| MSC 44457 | Mature | 990 | SE | SE | IR | IR | IR | IR | SE | SE | IR | IR | SE | SM* | SM* | SM* |
| MSC 46735 | Mature | 994 |  | SM | IR | IR | IR | IR | SE | IR | IR | IR | SM* | SM* | SM* | SM* |
| MSC 42671 | Mature | 1000 | SE | SE | IR | IR | IR | IR | IR | IR | IR | IR | IR | SM* | SM* | SM* |
| MSC 46739 | Mature | 1001 |  | SM | SM | IR | IR | IR | SE | IR | SE | SE | SE | SM* | SM* | SM* |
| MSC 44458 | Mature | 1010 |  | IR | IR | IR | SE | SE | IR | IR | IR | IR | SM* | SM* | SM* | SM* |
| MSC 44456 | Mature | 1033 |  | SE | IR | SE | IR | IR | IR | IR | SE | SE | SM* | SM* | SM* | SM* |

**Appendix 1.34. Male dataset for the nature of the mesial cutting edge on the Meckel’s cartilage.** Column header acronyms: **LPa** = left parasymphyseal tooth; **RPa** = right parasymphyseal tooth; **STL** = stretched total length. Descriptive acronyms: **IR** = irregular cutting edge; **SE** = serrated cutting edge; **SM** = smooth cutting edge. * = gynandric tooth morphology. Color codes: **Black** = no data; **Dark grey** = serrated cutting edge; **Light grey** = irregular cutting edge; **White** = smooth cutting edge. All pups were *in utero*. Life stage for males based on extent of calcification of the myxopterygia (see Materials & Methods).

**Appendix 1.35: Mean number of different prey items within the diets of *Rhizoprionodon terraenovae* within the northern Gulf of Mexico**

| **Size Class (STL)** | **Sex** | **Mean %N Fish** | **Mean %N Squid** | **Mean %N Crab** | **Mean %N Shrimp** | **Mean %N Other** |
| --- | --- | --- | --- | --- | --- | --- |
| 500-600 mm | Female | 0.143 | 0.000 | 0.000 | 0.000 | 0.714 |
| 600-700 mm | Female | 0.173 | 0.095 | 0.042 | 0.042 | 0.649 |
| 700-800 mm | Female | 0.400 | 0.000 | 0.067 | 0.067 | 0.467 |
| 800-900 mm | Female | 0.222 | 0.000 | 0.333 | 0.000 | 0.444 |
| 900-1000 mm | Female | 0.365 | 0.000 | 0.071 | 0.082 | 0.482 |
| 1000-1100 mm | Female | 0.543 | 0.002 | 0.000 | 0.031 | 0.361 |
| 500-600 mm | Male | 0.352 | 0.000 | 0.000 | 0.056 | 0.593 |
| 600-700 mm | Male | 0.277 | 0.083 | 0.062 | 0.171 | 0.407 |
| 700-800 mm | Male | 0.621 | 0.000 | 0.000 | 0.056 | 0.282 |
| 800-900 mm | Male | 0.529 | 0.000 | 0.052 | 0.114 | 0.305 |
| 900-1000 mm | Male | 0.662 | 0.018 | 0.045 | 0.106 | 0.168 |
| 1000-1100 mm | Male | 0.250 | 0.000 | 0.000 | 0.042 | 0.708 |

**Appendix 1.35. Mean number of different prey items within the diets of northern Gulf of Mexico *Rhizoprionodon terraenovae*. STL** = stretched total length. Data derived from Drymon, Powers & Carmichael (2012) and Harrington et al. (2016).

**Appendix 1.36: Mean weight of different prey items within the diets of northern Gulf of Mexico *Rhizoprionodon terraenovae***

| **Size Class (STL)** | **Sex** | **Mean %W Fish** | **Mean %W Squid** | **Mean %W Crab** | **Mean %W Shrimp** | **Mean %W Other** |
| --- | --- | --- | --- | --- | --- | --- |
| 500-600 mm | Female | 0.272 | 0.000 | 0.000 | 0.000 | 0.585 |
| 600-700 mm | Female | 0.234 | 0.122 | 0.055 | 0.010 | 0.579 |
| 700-800 mm | Female | 0.400 | 0.000 | 0.147 | 0.053 | 0.400 |
| 800-900 mm | Female | 0.333 | 0.000 | 0.333 | 0.000 | 0.333 |
| 900-1000 mm | Female | 0.486 | 0.000 | 0.071 | 0.086 | 0.357 |
| 1000-1100 mm | Female | 0.710 | 0.000 | 0.000 | 0.062 | 0.165 |
| 500-600 mm | Male | 0.444 | 0.000 | 0.000 | 0.111 | 0.444 |
| 600-700 mm | Male | 0.353 | 0.083 | 0.060 | 0.168 | 0.335 |
| 700-800 mm | Male | 0.615 | 0.000 | 0.000 | 0.052 | 0.292 |
| 800-900 mm | Male | 0.760 | 0.000 | 0.049 | 0.097 | 0.094 |
| 900-1000 mm | Male | 0.636 | 0.000 | 0.045 | 0.091 | 0.227 |
| 1000-1100 mm | Male | 0.332 | 0.000 | 0.000 | 0.001 | 0.667 |

**Appendix 1.36. Mean weight of different prey items within the diets of northern Gulf of Mexico *Rhizoprionodon terraenovae*. STL** = stretched total length. Data derived from Drymon, Powers & Carmichael (2012) and Harrington et al. (2016).
